# Conspicuousness, phylogenetic structure, and origins of Müllerian mimicry in 4000 lycid beetles from all zoogeographic regions

**DOI:** 10.1038/s41598-021-85567-x

**Published:** 2021-03-16

**Authors:** Michal Motyka, Dominik Kusy, Michal Masek, Matej Bocek, Yun Li, R. Bilkova, Josef Kapitán, Takashi Yagi, Ladislav Bocak

**Affiliations:** 1 Laboratory of Diversity and Molecular Evolution, CATRIN-CRH, Palacky University, 17. listopadu 50, 771 46 Olomouc, Czech Republic; 2 Department of Optics, Faculty of Science, Palacky University, 17. listopadu 12, 771 46 Olomouc, Czech Republic; 3 Department of Biological Sciences, Graduate School of Science, Osaka Prefecture University, 1-2 Gakuen-cho, Naka-ku, Sakai, Osaka 599-8570 Japan

**Keywords:** Mullerian mimicry, Phylogenetics, Biogeography

## Abstract

Biologists have reported on the chemical defences and the phenetic similarity of net-winged beetles (Coleoptera: Lycidae) and their co-mimics. Nevertheless, our knowledge has remained fragmental, and the evolution of mimetic patterns has not been studied in the phylogenetic context. We illustrate the general appearance of ~ 600 lycid species and ~ 200 co-mimics and their distribution. Further, we assemble the phylogeny using the transcriptomic backbone and ~ 570 species. Using phylogenetic information, we closely scrutinise the relationships among aposematically coloured species, the worldwide diversity, and the distribution of aposematic patterns. The emitted visual signals differ in conspicuousness. The uniform coloured dorsum is ancestral and was followed by the evolution of bicoloured forms. The mottled patterns, i.e. fasciate, striate, punctate, and reticulate, originated later in the course of evolution. The highest number of sympatrically occurring patterns was recovered in New Guinea and the Andean mountain ecosystems (the areas of the highest abundance), and in continental South East Asia (an area of moderate abundance but high in phylogenetic diversity). Consequently, a large number of co-existing aposematic patterns in a single region and/or locality is the rule, in contrast with the theoretical prediction, and predators do not face a simple model-like choice but cope with complex mimetic communities. Lycids display an ancestral aposematic signal even though they sympatrically occur with differently coloured unprofitable relatives. We show that the highly conspicuous patterns evolve within communities predominantly formed by less conspicuous Müllerian mimics and, and often only a single species displays a novel pattern. Our work is a forerunner to the detailed research into the aposematic signalling of net-winged beetles.

## Introduction

Defence mechanisms are inextricably linked to biological fitness and among them Müllerian mimicry is one common anti-predatory strategy^1–3^. The evolution of mimicry has been studied from various perspectives, but the considered numbers of interacting species and patterns under consideration have usually been low^4–6^. In order to investigate the evolution of mimicry from a long-term perspective, while simultaneously studying a high number of interacting species, we need to focus on ancient and highly diversified lineages^7,8^. Unlike the well-studied *Heliconius* system, some lineages of aposematically coloured insects contain up to several hundred species in a single geographic region and up to a hundred species in a single locality^9,10^. This means that unpalatable (or more widely unprofitable) aposematically coloured species dynamically interact within complex communities containing multiple patterns, imperfect mimics, as well as Müllerian and Batesian co-mimics. Here, we study the world fauna of net-winged beetles (Coleoptera: Lycidae), an elateroid family with pronounced chemical protection, diverse aposematic colouration, and ~ 4200 species^9,11^.

**Figure 1:**
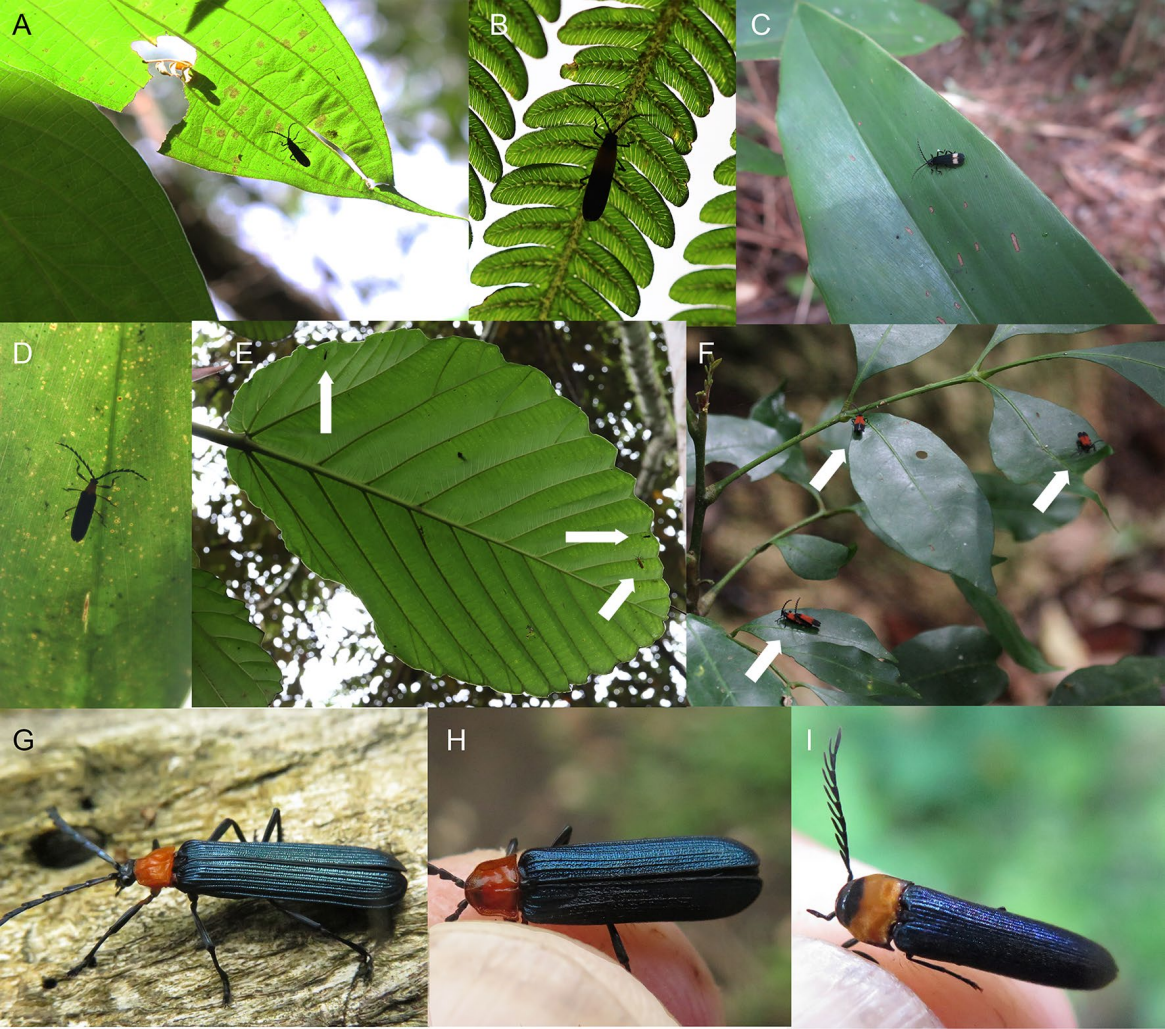
Aposematically coloured Lycidae in nature. (**A**) *Microtrichalus* sp. on a leaf observed against the clear sky. (**B**) *Metriorrhynchus* sp. on a fern leaf. (**C**) Metriorrhynchini on an upper side of a leaf. (**D**) *Eniclases* sp. on a leaf. (**E**) Three differently coloured species of Metriorrhynchini on a single leaf within an aggregation of net-winged beetles. The photographs (**A**–**E**) were taken in New Guinea. (**F**) The aggregation of *Metriorrhynchus* sp. in southern Queensland. (**G**–**I**) The members of the orange/metallic mimicry ring in the northern Sulawesi: (**G**) *Metriorrhynchus* sp.; (**H**) *Plateros* sp.; (**I**) Eucnemidae indet. All photographs and drawings by authors listed under the title of this study.

**Figure 2:**
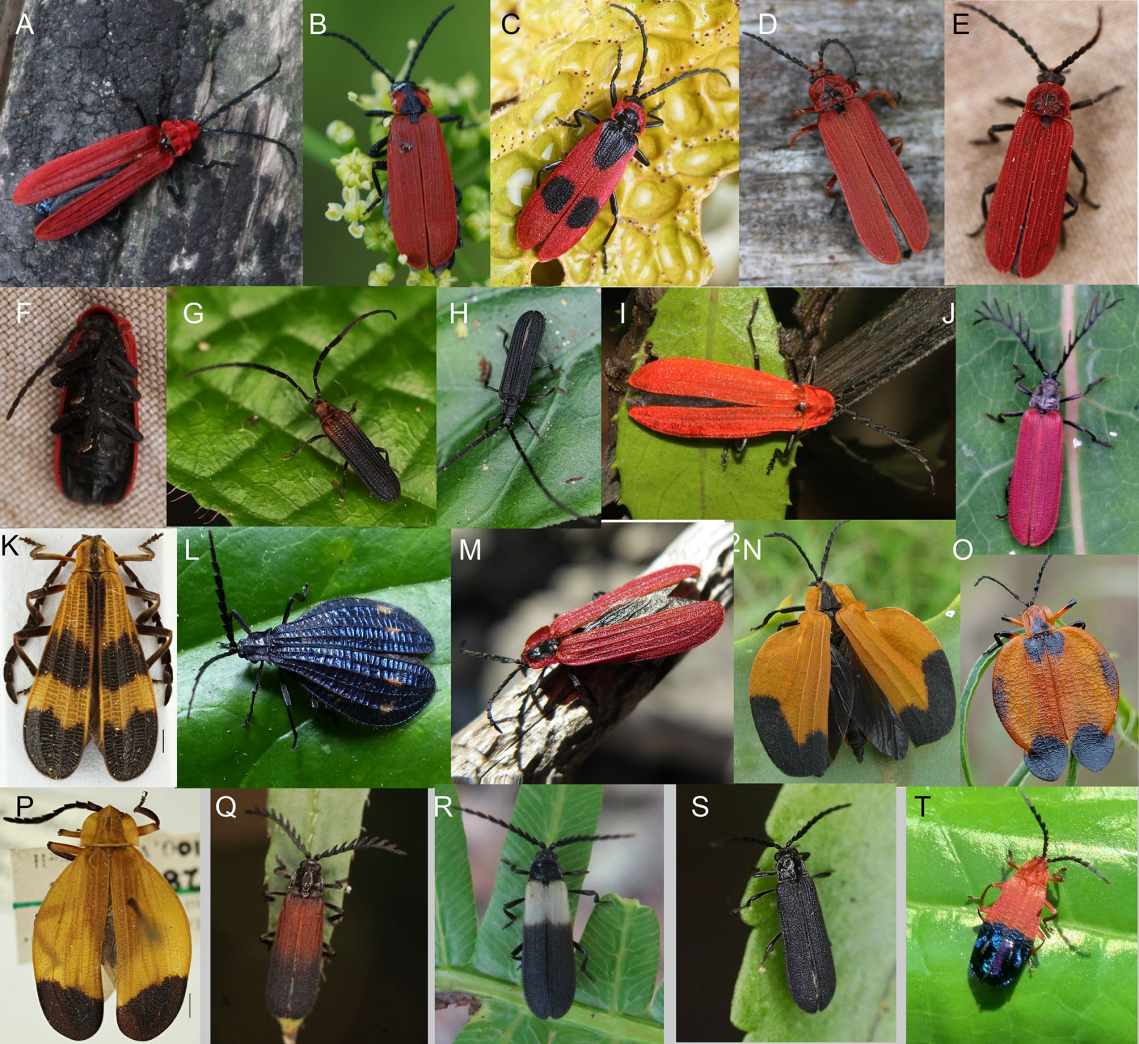
Aposematically coloured Lycidae in nature. (**A**) *Calochromus* sp., Yunnan. (**B**) *Lygistopterus sanguineus*, Czech Republic. (**C**) *Parantis triguttatus*, Yunnan. (**D**) *Dictyoptera* sp., Yunnan. (**E**,**F**) *Dictyoptera* sp., Japan. (**G**) *Scarelus umbrosus*, Malaya. (**H**) *Scarelus anthracinus*, Malaya. (**I**) *Macrolycus* sp., Yunnan. (**J**) *Lyponia quadricollis*, Japan. (**K**) Calopterini, Ecuador, the photograph from the collection). (**L**) *Idiopteron* sp., Ecuador, the photograph by M. J. Eising, Utrecht. (**M**) *Lycostomus kraatzi*, Turkey. (**N**) *Lycus* sp., the photograph by T. Rulkens, CC BY-SA 2.0. (**O**) *Lycus trabeatus*, South Africa, the photograph by B. Dupont, CC BY-SA 2.0; (**P**) *Neolycus* arizonensis, Arizona, the photograph from the collection. (**Q**) *Plateros* sp., Malaya. (**R**) *Plateros* sp., New Guinea. (**S**) *Plateros* sp. Malaya. (**T**) *Thonalmus* sp., Dominican Republic. If not stated otherwise, the photographs were taken by authors listed under the title of this study.

**Figure 3:**
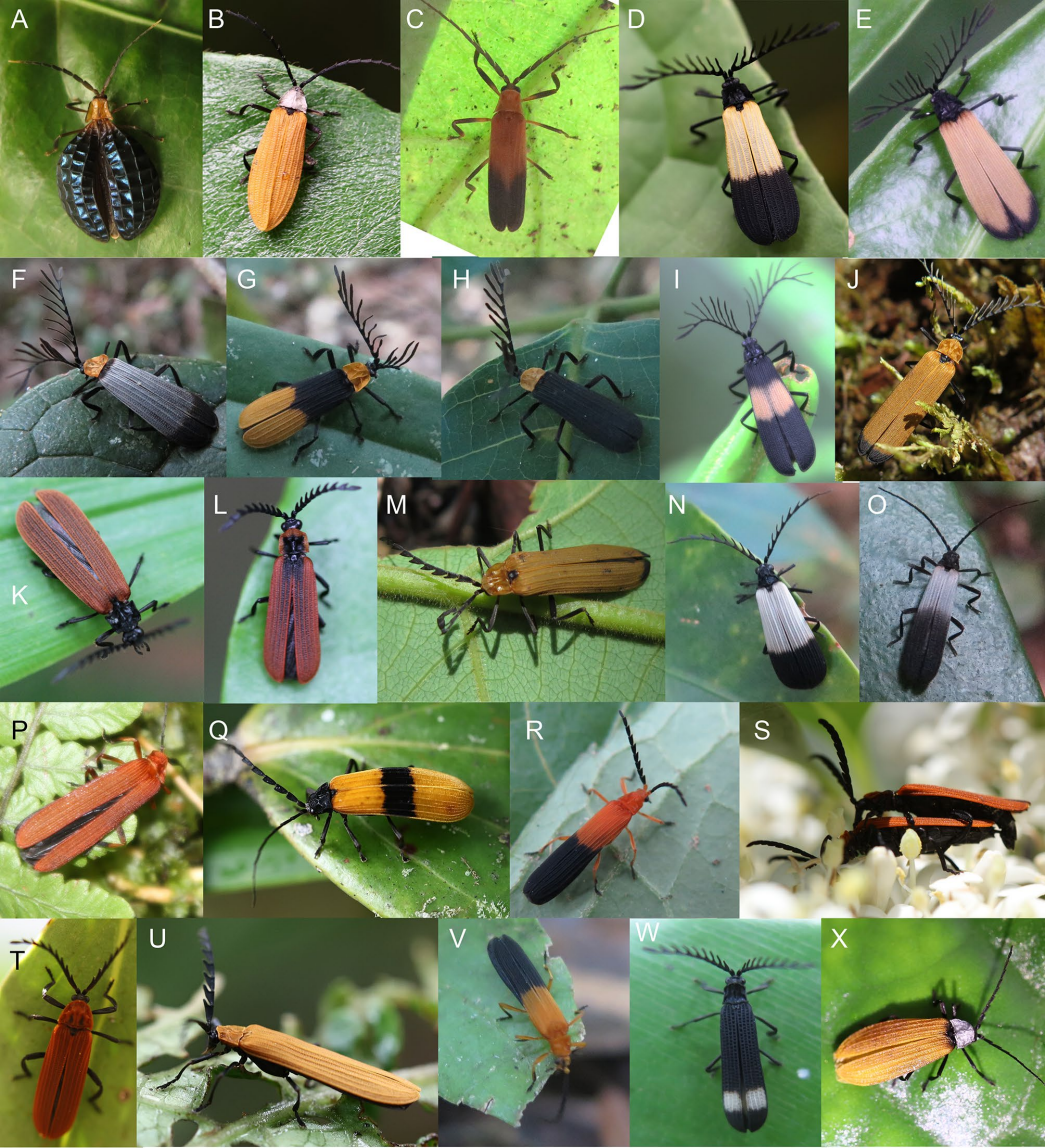
The examples of aposematic patterns displayed by net-winged beetles. (**A**) *Broxylus pfeifferi*, Sulawesi. (**B**) *Diatrichalus* sp., New Guinea. (**C**) *Eniclases* sp., New Guinea. (**D**) *Flabellotrichalus* sp., New Guinea. (**E**–**J**) *Cladophorus* spp., all from New Guinea. (**K**, **L**) *Porrostoma* spp., Queensland. (**M**) *Porrostoma* sp., New Guinea. (**N**–**V**) *Metriorrhynchus* spp., New Guinea. (**W**) Metriorrhynchini indet., New Guinea. (**X**) *Enylus* sp., New Guinea. All photographs by authors listed under the title of this study.

Net-winged beetles are a widely distributed family, related to soldier beetles, click beetles, and lampyroid families^11,12^. Most adults are inactive and sit on leaves under the forest canopy (Figs. 1, 2, 3). Individuals do not activate even when they register a movement very close to their body or when they are gently touched. Only a minority of lycids visit flowers in open-air spaces (Figs. 2P, 3S). The unpalatable lycids use aposematism as a defence strategy and form extensive geographically restricted mimicry rings^13–21^ (Figs. S1–S922). Their anti-predator strategy relies on chemical protection^15,22,23^. The adults produce a pungent odour as a response to handling, and bitter haemolymph is discharged from ruptures in the elytra and intersegmental membranes in legs and antennae^24^. Similar bleeding has been reported in related fireflies^25,26^ (Lampyridae), and soldier beetles discharge protective droplets from cuticular abdominal and thoracic vesicles^27–29^. The haemolymph of net-winged beetles contains l-methyl-2-quinolone and 2-methoxy-3-s-butylpyrazine and is effective in repelling potential predators^22,30^. Net-winged beetles commonly form aggregations^9,31–33^ (Fig. 1E,F), possibly increasing the effectiveness of their protection^34,35^. The protective odour might also serve as an aggregation signal, but it has never been studied in net-winged beetles, unlike other groups of insects^36^.

**Figure 4:**
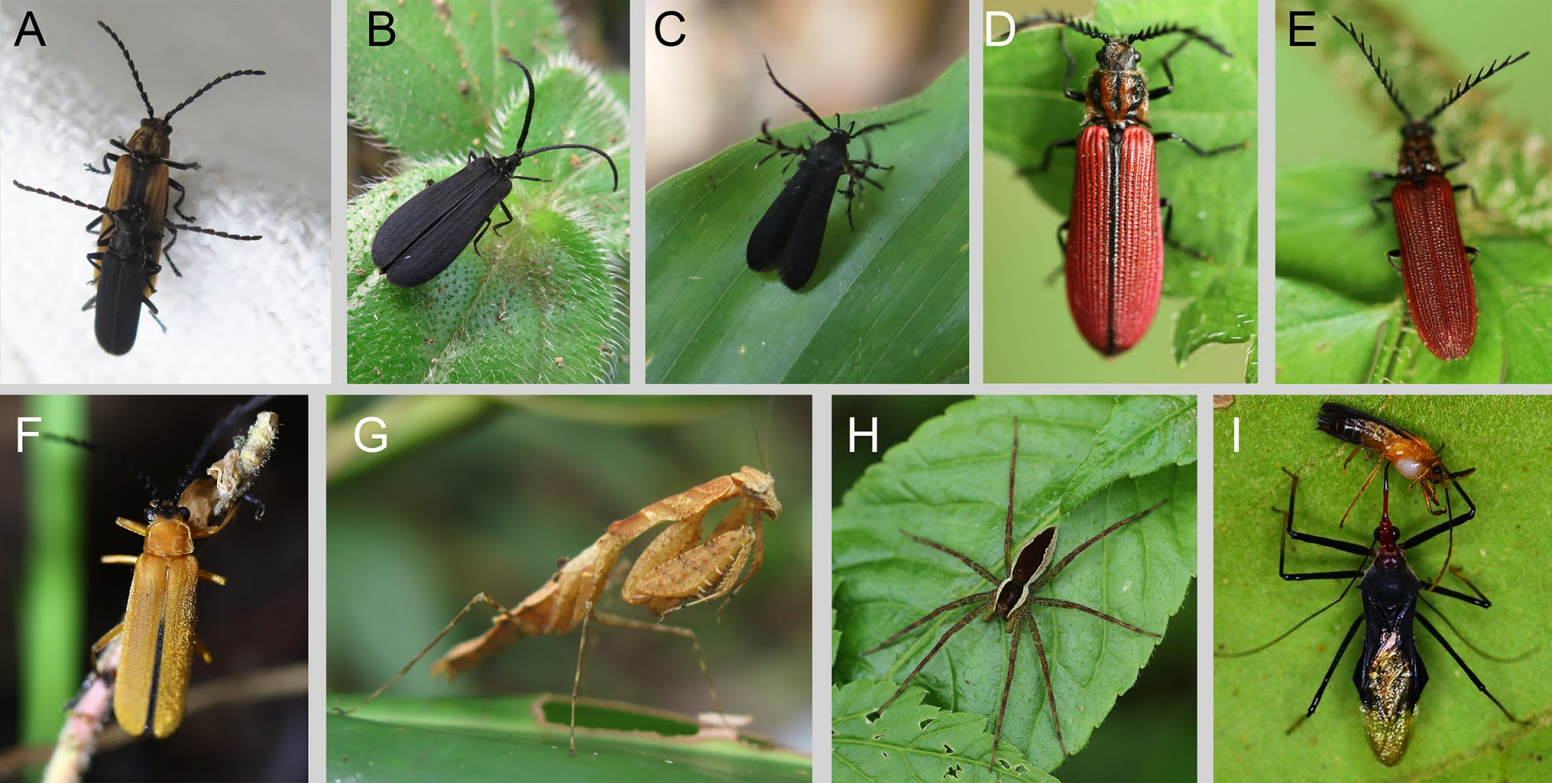
Co-mimics of net-winged beetles. (**A**, **F**) Cantharidae from New Guinea. (**B**,**C**) black coloured *Lyropaeus* sp. (Lycidae) and a moth with a similar body shape in Malaya. (**D**,**E**) Elateridae from Japan. Invertebrate predators of net-winged beetles. (**G**) Mantodea. (**H**) Araneae. (P) Reduviidae. All from New Guinea. All photographs by authors listed under the title of this study.

The unprofitability of net-winged beetles is signalled by bright colouration, a contrast between bright and dark coloured body parts, and by the body shape and size^9,37–42^ (Figs. 1, 2, 3, 4B, Figs. S1–S922). The uniformly black coloured forms are widespread in some regions and supposedly signal their unpalatability by the shape of their body, which provides a strong contrast against translucent leaves and is mimicked by some moths^9,42–44^ (Fig. 4B,C). Brightly coloured net-winged beetles can be uniform or display differently coloured body parts, similar to other unprofitable insect prey^13,14,23,41^ (Figs. 1, 2, 3). The effectiveness of their signal depends on ambient conditions^9,45,46^ (Fig. 1A–F), and prey behaviour^45,47^. Müllerian mimetic rings with the involvement of net-winged beetles are also formed by various sympatrically occurring unrelated insects, but the net-winged beetles regularly dominate in these mimicry rings, both in the number of species and in the number of individuals^9,13,40–42^. Non-lycid mimics belong to several beetle families and other insect orders, and many of them are considered as Batesian mimics^48–51^. Further co-mimics are known for the content of toxic or irritating compounds in their bodies and are putative Müllerian mimics (e.g. soldier and blister beetles^17,27,28,52–54^, Figs. 1I, 4A,C–F).

Information on predators of net-winged beetles is similarly limited, and we must suppose that a whole spectrum of predators attack the beetles. Birds are visually foraging, and they were cited as predators^22^, but no experiments have been conducted and the reports have been based on occasional observations. Several groups of invertebrates were reported on: jumping spiders, mantids, robber flies, and assassin bugs^22^ (Fig. 4G–I). The jumping spiders have experimentally proved to be effective predators in the case of fireflies^55,56^, and it is possible that they also prey on net-winged beetles.

The model of Müllerian mimicry is based on the benefit obtained by a protected prey which signals its unpalatability and shares a predation load with its co-mimics^1–3^. Here, predation load is defined as the proportion of individuals removed from co-mimic populations by naïve predators and because of their errors. A single, effective signal should be shared in the order to maximize the benefits of mimicry. To study the processes of signal evolution and effectiveness, we must answer several questions. First, the shared aposematic signal indicates the presence of a selective force, and only rejects a similarity due to common ancestry when it is displayed by unrelated species. Second, we need to evaluate the effectiveness of signalling (the internal and external contrasts and the signalling power of various components of the signal and eventually the various perceptions of the signal by individual predators^57–61^). Furthermore, the evolution of the signal might be affected by constraints which prevent high levels of similarity (imperfect mimics, relaxed selection^62–65^). We also need to consider how many aposematic patterns are present in a region. Multiple patterns in a single place, often within a single aggregation, have been reported^9,41,66^.

As described above, Lycids are a big group of putatively aposematic, conspicuously signalling species with a world-wide distribution, potentially taking part to several Müllerian mimicry rings. Investigating the diversity of aposematic signalling in ~ 4000 lycids on the scale of zoogeographic regions and over the period of the last hundred million years can provide invaluable information about the origin of aposematism and the evolution of aposematic signals. This work is a starting point for many future experiments focussing on specific local assemblages, but here we focus in discussing the differences between patterns, as well as their phylogenetic origins, i.e. how old are the principal patterns and whether highly conspicuous patterns rapidly evolved from their cryptic relatives or gradually evolved from the aposematically coloured relatives. These factors are also of interest to ecosystem-based studies because phylogenetic structure and signalling plasticity have consequences for the evolution of mimicry in close interactions within each locality.

We assume that the complexity of local rings might partly depend on the regional signalling diversity and on the intensity of migration between neighbouring ecosystems. We thus quantify the diversity of lycid beetle coloration and patterning, focusing on their signal strength in the spectrum visible to humans, relationships, and distribution. Our main aims are to (1) describe colour pattern diversity of lycid beetles; (2) investigate their distribution and evolutionary origins (timing, order of diversification); (3) investigate how alpha-diversity, phylogenetic diversity, and abundance correlate with the number of aposematic patterns occurring in a geographic region.

## Methods

### Field and museum research

Field observations and voucher sampling were conducted by the majority of the authors from 1989 to 2019 in all zoogeographical regions and additional material was provided by colleagues. The studied specimens are deposited in the authors’ Voucher Collection. The total catching effort cannot be standardized as the number of collected individuals depends on the duration of sampling, the season, weather conditions, and habitat conditions. Therefore, the regional abundance is categorized as follows: (a) low (< 100 individuals collected by a collector and a month, 1–5 species in a place for a fraction of a year); (b) medium (100–300 individual/month; 6–30 species present in ecosystems for about a half of the year), high (> 300 individuals/month, present whole year in up to 100 species in a place). Further data on aposematic patterns were obtained from museum collections (Supplements).

### Colour evaluation

Two sets of photographs were used for colour evaluation and estimation of the contrast in the colour spectrum visible to humans. The photographs displaying net-winged beetles in their natural environment were taken by the authors (except Fig. 2L provided by M. J. Eising) using digital cameras under natural light conditions and with automatic colour balance. These photographs are not calibrated. Therefore, their evaluation is limited to the relative contrast within a single snapshot. The further set of photographs consists of calibrated photographs of selected representatives for each aposematic pattern and three plant leaves representing backgrounds (the upper and bottom sides of each leaf). Specimens and leaves were illuminated with three Solux lightbulbs (Tailored Lighting Inc., Rochester, NY) with a colour temperature 4700 K and photographed with a Canon EOS M6 camera equipped with a Canon zoom lens 18–150 mm f/3.5–6.3 IS STM. Three plants were arbitrarily selected as background types for net-winged beetles in order to achieve a range of contrasts: *Ficus lyrata* Warb. (medium thickness, green upper side, light coloured bottom side), *Hoya carnosa* (Linnaeus *filius*) Brown (dark coloured, glossy upper side), and *Yucca guatemalensis* Baker (light-green leaf).

Images were converted from a Canon raw format (CR2) to a 48-bit tiff with dcraw program (https://www.dechifro.org/dcraw/) in linear intensity scale, calibrated with a IT8.7 calibration target (R110112, www.coloraid.de) using a CoCa program with implemented Argyll Colour Management System (www.argyllcms.com), and finally converted to 24-bit sRGB IEC61966-2.1 colour space using perceptual rendering intent. The colour space characteristics of the bright and dark coloured elytral areas were quantified in the CIE La*b* absolute colour space (International Commission on Illumination, CIE 1976). i.e., the colour spectrum visible to humans. The colour samples were taken five times from an area of 31 × 31 pixels at various points of measured body parts in Photoshop 13.0. The average colour values were computed and the colour distance DeltaE La*b* CIE 76 was counted as the Euclidian distance between colour positions in the 3D CIE La*b* colour space. The processing of photographs follows the methods used by the authors of an earlier study^41^. The DeltaE distances were counted between beetle dorsum and background (both, bright and dark coloured body parts versus each of the six leaf backgrounds, i.e. the upper and bottom side of each leaf, the external contrast is further designated as EC-DeltaE/Upper, EC-DeltaE/Bottom) and distance between colours displayed on the dorsum, i.e. internal contrast, is designated as IC-DeltaE^67^.

Additionally, the collection and voucher specimens were photographed so we could document the diversity of aposematic signalling, the variability of aposematic patterns, and intraspecific variability. These illustrations were taken using a Canon EOS 700D digital camera attached to an Olympus SZX-16 binocular microscope, using two StoneMaster LED light panels (temperature 5700 K) and a white reflective panel. The white balance was custom set with a white target panel. The photographs were taken at various focus levels and the final photographs were assembled using Helicon Focus v.7.6.1.

We mapped the geographic distribution of aposematic patterns through the examination of sequenced individuals, and we additionally, evaluated specimens in collections and searched the relevant literature. Nineteen categories were used for descriptions of patterns; aberrant patterns were described individually as non-categorized. We considered the uniform colouration displayed on the dorsal side of the body, the different colours displayed on the pronotum and the elytra, and a group of patterns with two colours displayed on the elytra. Additional patterns include striate, punctate, tricoloured, and reticulate types, and several unique patterns, e.g. green, green/black, black/amethyst; see Figs. S1–S922, Table S1). Further, we noted the morphological modifications of elytra connected with the strength of the signal—the posteriorly widened elytra and globuliform elytra (Figs. 2L, 3A).

### Molecular data, phylogenetic analyses, and zoogeography

The phylogenetic investigation is based on earlier published *cox1*, *nadh5*, and *rrnL* mtDNA, S*SU* and d2 loop of *LSU* rRNA, and the transcriptomic and genomic data^11,24,68^. No data were specifically produced for this. We assembled a matrix of 751 lycid terminals and *Iberobaenia minuta*, a sister to the Lycidae^69^, as an outgroup (Table S2). The length invariable protein-coding *cox1* and *nadh5* sequences were aligned using TRANSALIGN^70^ and the rRNA fragments using default parameters of MAFFT v.7.2^71^. The individual gene alignments were concatenated into a supermatrix using FASconCAT-G v.1.04^72^. The substitution models were identified using ModelFinder^73^. All models and partitions are listed in Table S3. We estimated maximum likelihood (ML) trees using IQ-Tree v.1.6.1^74^ and the option-g allowing us to constrain the deep topology. The bootstrap support (BS) was obtained by applying UFboot^75^ with 5000 replicates. To obtain a tree with deep splits supported by the phylogenomic analysis, the relative position was fixed for 23 terminals whose relationships have been inferred from ~ 4200 orthologs (representatives of all subfamilies and 22 of 30 recognized tribes, Fig. S923). The positions of other terminals, including the tribes for which transcriptomic data are still unavailable, were unconstrained. In such a way, the relationships among most tribes are based on transcriptomics and the shallow splits on rRNA and mtDNA fragments.

The complete dataset could not be reliably analysed using BEAST 1.8.1^76^. Therefore, we pruned the dataset to 3–5 taxa per tribe (the terminals representing the deepest splits). We dated the tree with the widely used rates for *cox1* (0.0115 substitutions per lineage and a million years, s/l/my), *nadh5* (0.0177s/l/my), and *rrnL* (0.054s/l/my^77,78^). Subsequently, we validated the results with the available records of *Burmolycus minutus*, Erotini indet., and *Prototrichalus* spp.^79,80^^,unpubl data^. The analysis was set to HKY+I+G model and the birth–death speciation prior; genes and codon positions were partitioned, and analyses were run for 10^8^ generations with sampling frequency 10,000 generations. A maximum credibility tree was generated with TREEANNOTATOR v.1.8.1^76^ discarding 25% of the trees as a burn-in after checking the ESS values and a plateau phase in TRACER v.1.6^81^. Further, we used the database of biogeographic distribution data^11^ to relate the phylogenetic diversity (estimated as a number of tribes recorded in the region) and alpha diversity to the regional diversity of aposematic patterns.

### Ancestral colour pattern reconstruction and phylogenetic signal analysis

For the reconstruction of ancestral colouration, we used six categories: (1) the upper side uniformly black, yellow, orange, or red; (2) a different pronotal and elytral colouration resulting in a combination of dark and bright coloured body parts; (3) bicoloured elytra with a combination of dark and bright coloured parts of the elytra; (4) fasciate elytra; (5) striate elytra; and (6) non-categorized patterns (e.g. metallic, reticulate, red/blue, red/green, uniformly green, red pronotum and metallic elytra). As only a few dimorphic species were present in the dataset, each specimen was separately coded and intraspecific polymorphism was expressed as a different code for specimens. The reconstruction of ancestral colour patterns was performed using the ‘discrete traits’ function in BEAST v.1.8.1. Each terminal was assigned to one of six states (Figs. S1–S922). The representation of patterns in the molecular analysis is incomplete and several rare patterns were unavailable for the phylogenetic analysis (Table S4). Therefore, they were studied using the dry-mounted specimens only and assigned to their respective relatives using morphology. The analysis was run using the same settings as described at https://beastclassic.googlecode.com/files/ARv2.0.1.pdf for 10^8^ generations with sampling every 10,000 generations. A burn-in was set to the first 25% of trees. The effective sample size values and plateau phase were checked in Tracer v.1.6 and the maximum credibility tree was generated using TREEANNOTATOR v.1.8.1^76^. We set the age of the Lycidae root to 1 and dated the origins in relative time units (RTU), with 1.0 for the oldest and 0.0 for the extant splits.

The phylogenetic signal was calculated using the δ Bayesian approach, suitable for analysing categorical traits^82^. To calculate δ-statistics, we used the R package ape^83^ and the code provided in GitHub (https://github.com/mrborges23/delta_statistic^82^). We removed all taxa that had no information about adult colouration (several terminals representing larvae) and the tree was split into clades with more than 20 taxa as it is impossible to compute δ-statistics for subtrees smaller than this threshold. All parameters were set to default with the exception of sim = 1,000,000, thin = 100, and burn = 2500.

## Results

### Characteristics of aposematic patterns

The net-winged beetles display a wide range of visually distinct patterns and we present photographs and distribution data for ~ 600 lycid species and ~ 200 species of non-lycid co-mimics from all zoogeographic regions (Figs. 1, 2, 3, 4, Figs. S1–S922). The illustrated individuals represent a subset of ~ 4000 lycid species studied in collections and in the field. The detailed descriptions and measured contrasts are given in the Supplementary Text and Tables S5 and S6.

#### Uniform upper body side

The monochromatic patterns include uniform black forms with low external contrast (> 500 spp. from the majority of regions; Fig. 2S), uniform yellow forms with high contrast on the upper leaf side (> 500 spp.; Fig. 3U), uniform orange patterns (Fig. 3P), and the bright red pattern, both with high contrast (~ 200 spp.; Figs. 2A,D,E,I, 3P). The external contrast of bright forms is ~ 1.35 times higher than those of the dark coloured ones. The metallic colouration is rare and has generally low external contrast in the measured spectrum (< 50 spp., Fig. 2L).

#### The bicoloured upper body side

Bicoloured forms signal their presence by the internal contrast between bright and dark coloured body parts and by the external contrast between respective body parts and background. Their internal contrast is generally higher than the external contrast between a bright body part and the background. The principal component of the aposematic signal is the internal contrast as summarized in Table S6. The bicoloured patterns include a differently coloured pronotum and elytra, or bicoloured elytra (Fig. 3G). A unique bicoloured pattern is displayed by Thonalmini (Fig. 2T).

#### Fasciate elytral patterns

The fasciate type contains several forms (Figs. 2M, 3I,Q). The internal contrast is variable (Figs. 2K, 3Q) but mostly higher than the external one. The characteristic bands are conspicuous and easily remembered by a human observer. The fasciate forms are dominant in the Neotropical region.

#### Striate bright and dark elytral patterns

The striate pattern is uncommon, and most species are known from the Neotropical region (Fig. S610).

#### Punctate bright and dark elytral patterns

The punctate pattern is very characteristic and easy to remember for a human observer, but the number of species with clearly delimited elytral patches is very low, e.g., *Parantis* sp. (Fig. 2C).

#### Tricoloured dorsum

The combination of three distinct colours in the upper side of an individual is uncommon and only a few cases have been reported from New Guinea (Fig. 3F).

#### Reticulate elytral patterns

Reticulate patterns are highly conspicuous on any background but were noted in a few species. Several species were found in the higher elevations of the Kinabalu massif in north-eastern Borneo (Figs. S222–S227). Further, some *Calopteron* have a similar structure of costae in a humeral (Fig. S582) or apical part of the elytra (Fig. S581).

#### Non-categorised patterns

Several colour patterns do not fit in any of the above-listed categories. These were mostly recorded in New Guinea where we identified the highest diversity of colour patterns and they are shown in Figs. 3B,X and Figs. S786, S814–S819. Several of these patterns are represented by a single species.

Differently coloured males and females were identified in a few species. Both sexes are aposematically coloured, they are differently coloured but both sexes belong to the same category of aposematic patterns as defined for the purpose of the reconstruction (see “Methods”). Only the patterns of the specimens included in the analysis were coded as details have already been discussed in earlier publications^9,40,84,85^.

### The origins of aposematic patterns

**Figure 5:**
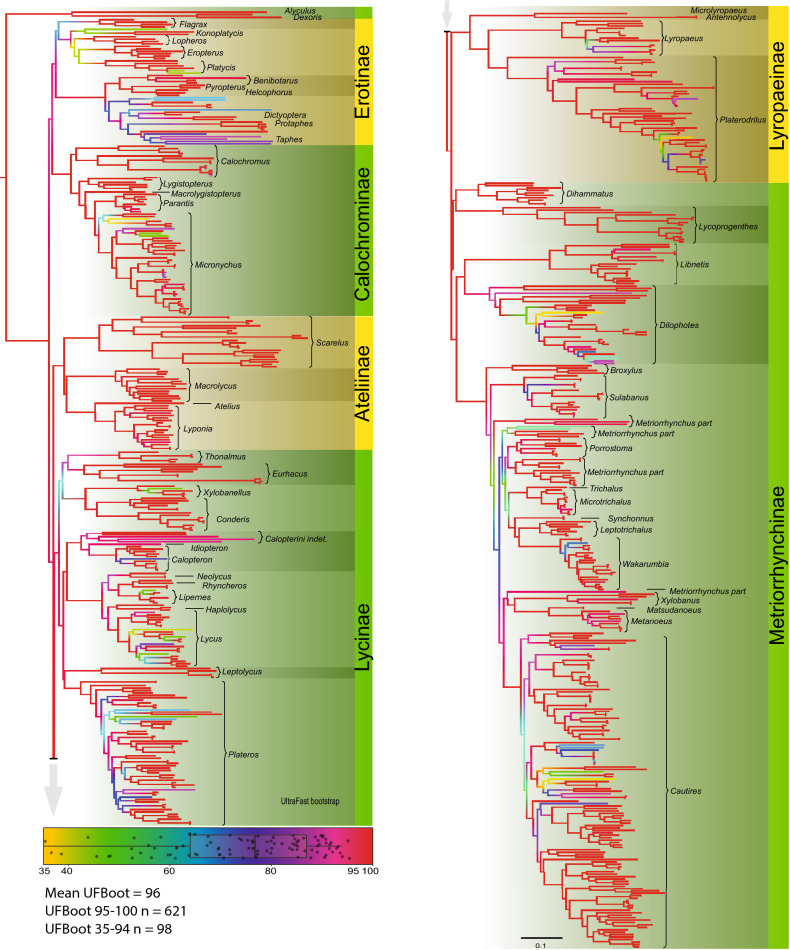
The phylogenetic tree of net-winged beetles recovered by the maximum likelihood analysis of five rRNA and mtDNA fragments and constrained by the relationships of 22 terminals for which are available genomic data. The constrained topology is shown in Fig. S915. The figure was produced in a whole by authors listed under the title of this study.

The integration of the phylogenomic backbone and five markers has led to a well-resolved phylogeny. The tribes whose relationships have not been constrained and whose positions were inferred only from rRNA and mtDNA markers, were found in the expected phylogenetic relationships, except for the Alyculini which were found in the relationships to Dexorini (Fig. 5). The mean UFboot value was 96% (n = 718). Only 97 values were lower than 95% (i.e. the threshold for a significantly resolved split) and these mostly represent the terminal splits.

**Figure 6:**
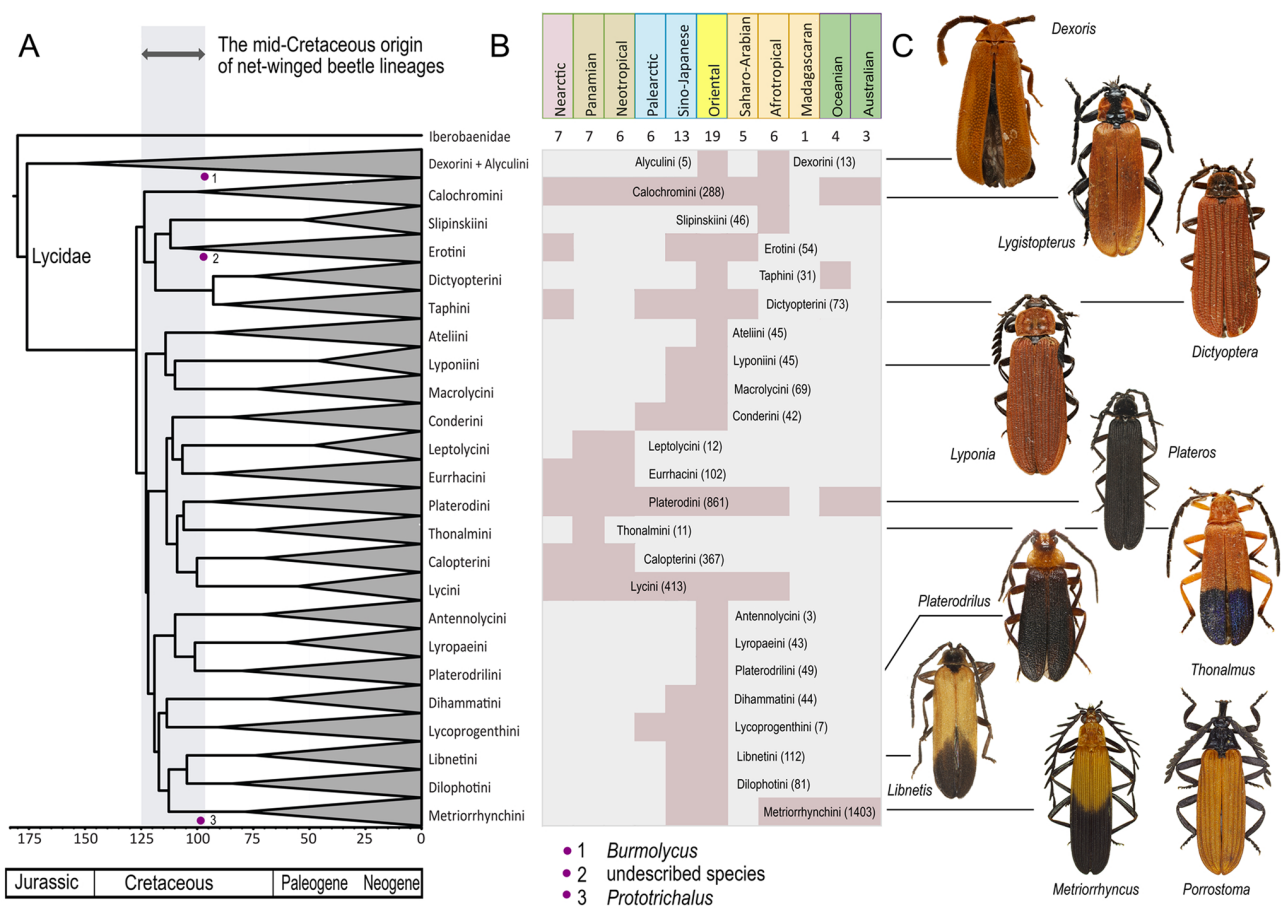
(**A**) The simplified dated tree of Lycidae, the red dots show the position of available fossils. (**B**) The distribution of lycid tribes with the number of described species for each tribe. (**C**) The examples of lycid general appearance. The full resolution tree is available in Fig. S917. All photographs and drawings by authors listed under the title of this study.

Using the constrained topology, we dated the origins of crucial clades of net-winged beetles (Fig. 6). The present analysis was dated with molecular evolution rates, but the available fossils^79,80^ indicate that the inferred dated splits among subfamilies and tribes do not violate any of the available hard evidence. We suggest that the majority of the tribes diversified within 30 my in the mid-Cretaceous (125–95 mya), even though the origin of net-winged beetles is earlier, about the mid-Jurassic. The deepest splits among constituent taxa in individual tribes are often delayed (the mid-Paleocene to Palaeocene/Eocene diversification; Fig. 6A, Fig. S925).

**Figure 7:**
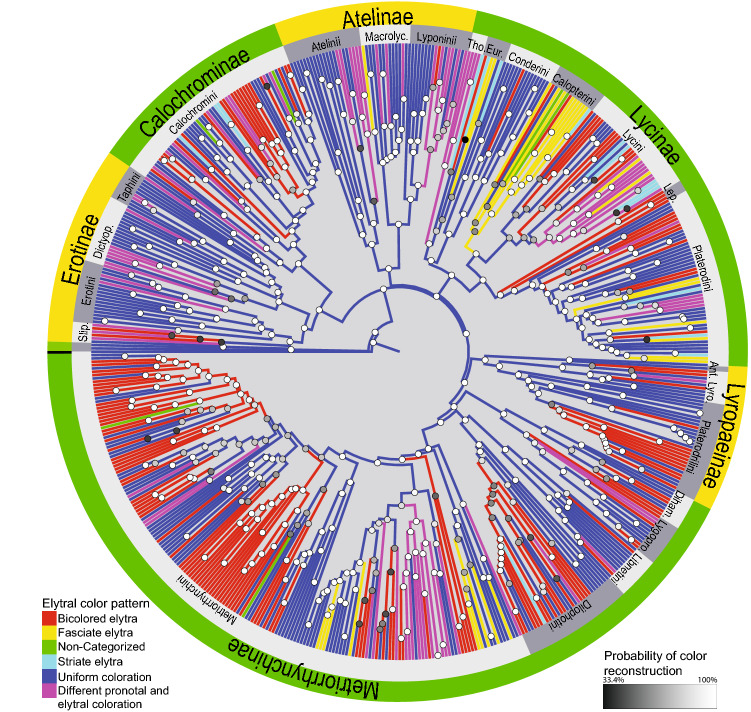
The Bayesian reconstruction of the net-winged beetle colouration. The full resolution tree is available in Fig. S918. The figure was produced in a whole by authors listed under the title of this study.

The reconstruction of the simplified spectrum of the six groups of aposematic patterns indicates the uniform coloured forms as ancestral (Fig. 7, Fig. S926). The uniform colouration was recovered for all splits among tribes and subfamilies and similarly, we found a uniform pattern at the deepest split in 20 of the 23 evaluated tribe-level clades. The fasciate pattern was reconstructed for the earliest split among the Calopterini, and the bicoloured elytra for the Leptolycini and Thonalmini. Among tribes with ancestral uniform colouration, the earliest origins of the bicoloured elytra were inferred at 0.57 RTU (= relative time units, see “Methods”) for Metriorrhynchini, followed by thirteen other tribes with this pattern originating from 0.52 RTU up to the present. Similarly, delayed origins were identified in relation to the contrast between the colours of the pronotum and the elytra (ten times, since 0.49 RTU up to the present). The fasciate pattern was established in three instances; the first was at 0.59 RTU in Calopterini, but as late as 0.28 RTU in Metriorrhynchini and Platerodini, and only recently in Eurrhacini and Lycini. Other patterns are evolutionarily young and include the striate forms whose origin is hypothesized to be 0.09 RTU in Calochromini and later in Platerodini, Eurrhacini, Dilophotini, and Lycini. In the latter cases the pattern is only displayed in individual species in terminal positions and their origins cannot be dated with confidence. Non-categorized patterns, i.e. punctate, metallic, green, orange/green, and tricoloured forms, were only recovered in terminal lineages and we assume they have recent origins. We only detected a limited number of reversions from bicoloured forms to uniformly coloured patterns (Fig. 7).

The phylogenetic signal tests show that colouration patterns are moderately conserved in the phylogeny of the Lycidae. The deepest lineages exhibit a lower phylogenetic signal, which suggests that the colouration is weakly conserved (Calochrominae 2.13, Erotinae 3.23, Ateliinae 2.81, Lyropaeinae 3.11), whereas the most species-rich subfamilies Lycinae and Metriorrhynchinae express nearly twice stronger effect of the evolutionary history in their colouration pattern trait (5.90 and 4.77, respectively).

All subfamilies produce uniform bright coloured forms, and all contain forms with different colourations of the pronotum and the elytra, and most contain species with bicoloured elytra (Table S8; Figs. S1–922). The fasciate patterns are less common and were recorded in Lyropaeinae (a single species), Lycinae (several *Lycus* F.; numerous Calopterini, a few Eurrhacini), and a few species of Metriorrhynchina. Similarly, the lycids with striate elytra are only known in some lineages (Lycini, Platerodini, Calopterini, Calochromini, Metriorrhynchini). The patterns with punctate elytra are limited to a few lineages (Fig. 2C, Figs. S584, S585, S587, S862). Additionally, some forms have reversed the positions of bright and dark coloured parts of the elytra (Fig. 3G, Figs. S211, S560–S568, S788–792). A complete overview of patterns in the subfamilies and tribes is presented in Table S8. The analysis of the samples included in the molecular study indicates that the most abundant species are those with uniform colouration, followed by those with bicoloured elytra. Only Erotinae and Ateliinae often display internal contrast with different colouration of the pronotum and the elytra. Our results indicate that uniform colouration which is dependent solely on external contrast (277 terminals) was found in less than half the terminals and patterns displaying the internal contrast were present in slightly higher numbers (299 terminals; Figs. S927–S928).

The characteristic sets of patterns for each region are shown in Figs. S1–S922 and Table S7. The lowest diversity of aposematic patterns was identified in the western part of the Palearctic region where net-winged beetles are generally rare. A similarly low diversity was recorded in Madagascar (a limited area, a single genus). The highest diversity of aposematic patterns was identified in New Guinea (22 patterns, nine of them unique and endemic, Table S7, Figs. S702–S822), the northern Andes (14 patterns; Figs. S547–S638) and in Sundaland, Malaya, and the Philippines (10 patterns, S97–S242). The number of patterns in other regions varied between 3 and 7. Higher signalling diversity was identified in Southern China, which includes the transitional Oriental/Palearctic areas of Yunnan and Guizhou, and in northern Queensland, where the species displaying aposematic patterns from south-eastern Australia and New Guinea occur in sympatry (Figs. S1–S922). Despite the large area and a relatively high number of species, a low number of patterns was recorded in Brazil, including Amazonia and the Atlantic forests, and in Sub-Saharan Africa (Fig. 8C, Figs. S307–S387).

**Figure 8:**
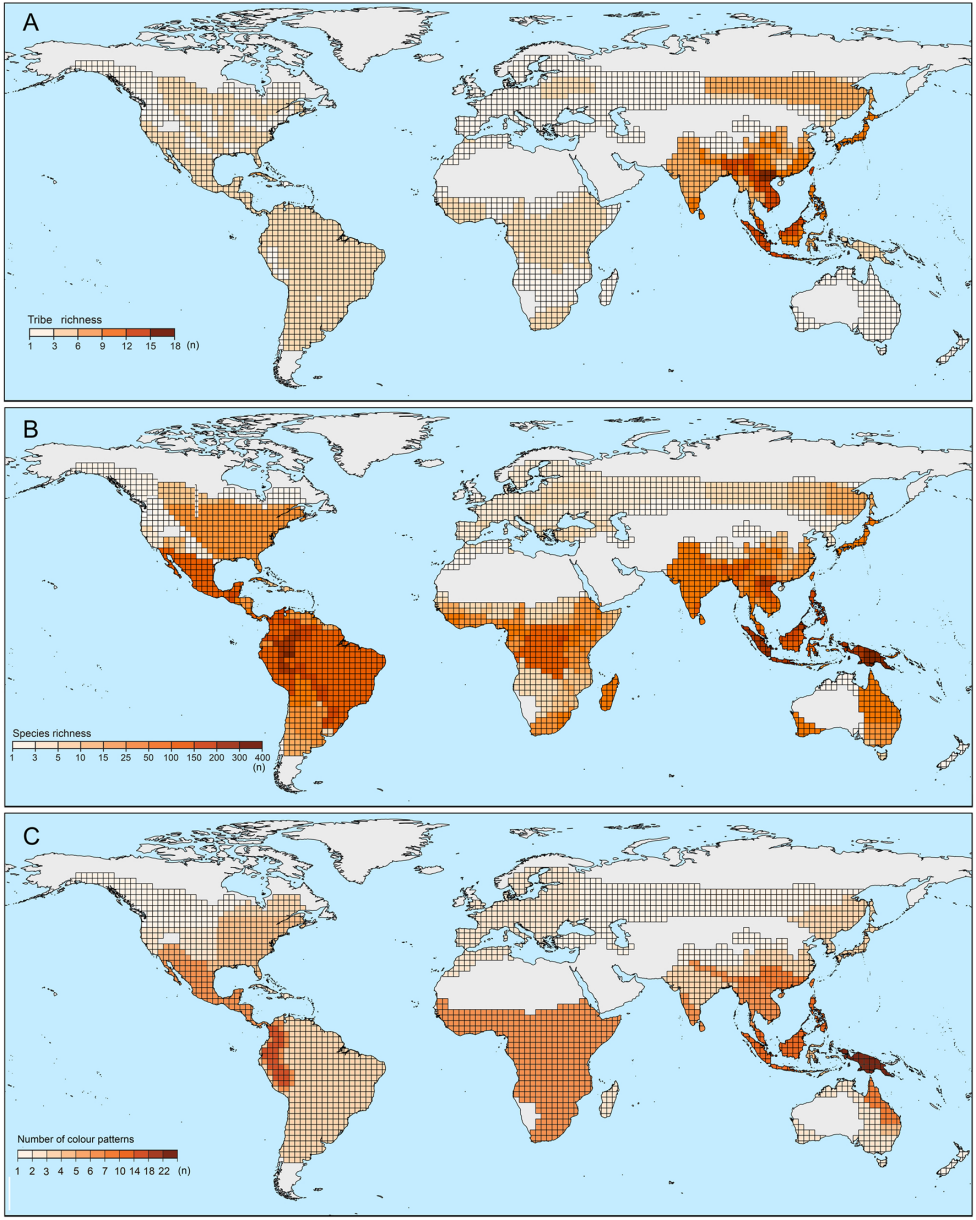
(**A**) The phylogenetic diversity of net-winged beetles as the number of recorded tribes. (**B**) The alpha-diversity as the number of species. (**C**) Aposematic signal diversity as the number of colour patterns recorded in the region. The distribution is displayed on the 2° grid. The figure was produced in a whole by authors listed under the title of this study.

### Abundance and diversity

No exact regional quantification of the abundance is available due to considerable local fluctuations. We can only approximately categorize three levels based on the collecting results in undisturbed forest habitats: (a) low: Europe, the lowlands to lower mountain forests of most of China, the Russian Far East, most areas of Sub-Saharan Africa, North America, the Caribbean, some seasonally dry regions of South America; (b) medium: the lowlands of South East Asia and Indo-Burma, the Himalayas, mountain forests of China, Japan, the Philippines, Sulawesi, the Moluccas, the humid regions of Sub-Saharan Africa, the majority of the Nearctic region, eastern Australia; (c) high: the mountains of the Great Sundas, mountains of northern Laos, mid to high altitudes in the northern Andes, and New Guinea. The estimation of abundance excludes the local and temporal occurrence of some flower-visiting species in secondary habitats and savannahs. Unlike their relatives, some species from these genera aggregate on flowers outside the forest canopy (see Supplements for further information) and they are usually not present under the canopy, which is where most species occur. Based on field observation and museum collections, Lycidae are more common in areas without a pronounced dry season. Even in the humid tropics, a higher abundance was observed in elevations 1300–2000 m a. s. l. compared to adjacent lowlands. The highest numbers of species have been reported from Sundaland and the Philippines (1270 spp. in total), continental Africa (656 spp.), South America (385 spp.), and New Guinea (341 spp.).

### Supporting field observations

The reflex-bleeding and pungent smell were confirmed for all adults when they were harshly disturbed. Haemolymph oozed from elytral ruptures, from intersegmental membranes of the thorax, antennae, and legs and was precipitated by alcohol when individuals were fixed (Figs. 4I, Figs. S38, S676, S764).

The conspicuousness of the aposematic patterns depends on the beetles’ behaviour and is quite low under some circumstances. We found many individuals sitting on the under sides of leaves and they often sat parallel with the main veins or at the tip with their heads directed towards the base of the leaf (Figs. 1A,B,E, 3Q). In such a position an individual controls access to its location, it is partly camouflaged, and cannot easily be identified from above, e.g. by flying birds. We often collected adults in multi-species aggregations where 100–300 individuals from up to 20 species were sitting on the leaves of a single shrub or a small group of shrubs under the forest canopy. Lycids were recorded outside such aggregations in much lower density.

The vivid aposematic colouration of Lycidae is often mimicked by other beetles (Figs. S1–S922), but these are not studied in detail in the present study. The similarity of some co-mimics is imperfect (Figs. S487–S491), but many species are very similar, and they cannot be recognized as non-lycids at first glance. The most common co-mimics are soldier beetles (Cantharidae) and fire-coloured beetles (Pyrochroidae), but co-mimics include a number of other beetle families. Additional co-mimics were identified in moths and true bugs. The mimetic polymorphism analogous to that of net-winged beetles was observed in New Guinean soldier beetles (Fig. 4A). We seldom observed attacks by predators. We commonly found net-winged beetles trapped in spider webs, but they were alive (Japan). When a net-winged beetle was thrown in a web, the spider checked the cause of the disturbance but never attacked a net-winged beetle (authors’ observation). We saw mantids and assassin bugs as they attacked and ate net-winged beetles in the Malay Peninsula and New Guinea, respectively (Fig. 4I).

## Discussion

### Conspicuousness of patterns

The signalling power of aposematic patterns depends on the conspicuousness of potential prey and its abundance, i.e. positive frequency-dependent selection^4,6,86–90^. We do not have data on putative predators, and therefore we will only discuss the conspicuousness of individual patterns in the colour spectrum visible to humans. It is highly probable that various patterns will have different levels of effectiveness in other colour spectra. Monochromatic perception can also provide a strong signal^91^, therefore we also consider the arrangement of differently coloured body parts as a component of conspicuousness.

Unprofitable prey often warn predators through their bright red, orange, and yellow colouration^30,92,93^ and these colours are commonly found in the patterns displayed by net-winged beetles (Figs. 1–3, 4B, Fig. S1–S922). The simplest signal is the contrast of the prey's uniform colour against background (external contrast). Our data show that the average external contrast increases gradually from a uniformly black colour, to dark red, metallic blue, yellow to light orange, and bright red forms which are the most conspicuous (average EC-DeltaE 32, 36, 38, 48, 53, respectively).

Net-winged beetles often have differently coloured elytra and pronotum (Figs. 1, 2, 3, 4, Fig. S1–S922), and in this way another component of conspicuousness is provided by the internal contrast which is apparent in the colour spectrum visible to humans as well as in monochromatic spectra^57,88,91^. The red colouration warns birds and mammals but it is not visible for some invertebrate predators. The black/bright contrast might provide at least some detectability in warning predators with a different visual system^94,95^. The internal contrast is regularly higher than the external contrast of the uniformly coloured forms (average IC-DeltaE 62–66 compared to EC-DeltaE 32–53 for uniform patterns). Additionally, the internal contrast is higher than the contrast between brightly coloured body parts and the background alone (~ 24% higher IC than EC-bright/background; Table S6). The signalling power of the forms with a differently coloured pronotum and elytra is putatively limited by the size difference between body parts. High contrast has evolved in some large bodied Metriorrhynchini (Fig. 3F,H). In this case, we have to consider the possibility that these net-winged beetles can be predated by birds which can clearly see red and yellow colours^96^.

Additionally, numerous species produce the internal contrast in bicoloured elytra (e.g. Figs. 1, 2, 3). The effectiveness of such a signal mainly depends on the colouration of the bright part of the elytra (compare 2Q and T, IC-DeltaE 38 versus 83), and the volume of the brightly coloured area. The proportion of the brightly coloured part of the elytra varies, but commonly occupies one to two thirds of the elytral surface and these net-winged beetles are very conspicuous on any kind of leaf surface. The patterns based on bi-coloured elytra are not limited by the size of the pronotum and the elytra and are more widespread than the previous pattern (Table S6, Fig. S927).

All the above listed colour patterns are very simple concerning the arrangement of differently coloured areas, and we assume that with many bicoloured palatable insects, these patterns might not be perceived as unique by some predators, especially if the colours of the body parts are not substantially different (IC-DeltaE < 50). We might classify various fasciate forms as more complex signals (Figs. 2K,L, 3Q). These patterns contain the internal contrast component (average DeltaE 62) and the characteristic arrangement of differently coloured areas. They include the various forms of striped elytra (Figs. S1–S922) and punctate patterns that are similarly eye-catching (Figs. 2C, 3W). Without any increase in the internal contrast, which might be constrained by the availability of pigments, these patterns potentially provide better protection because the mottled forms suffered a lower predation rate in earlier experiments^4^. Further, some lycids signal their unpalatability with reticulate elytra, usually with differently coloured costae and large elytral cells. The pattern and its evolution have been described in detail earlier^41^.

The effectiveness of the signal can also be increased by the greater area of the elytral surface. The body of a species can be slender, similar to its relatives, with only its elytra expanded or globulous in shape (Figs. 2N–P, 3A). The inflated elytra evolved independently in Metriorrhynchini (Fig. 3A), Lycini (Fig. 2N–P), and Calopterini (Fig. 2L), and always in a single or a few species and supposedly only recently as a derived trait.

The conspicuousness of various forms depends on their positions on leaves (Tables S5, S6). We, as representatives of visual predators, cannot clearly see colours from below if individuals sit on the bottom of translucent leaves. In that case, we mostly perceive the body shape and size (Fig. 1A–E). We suppose that natural predators face the same problem and we assume that observed differences in perception show that net-winged beetles can suppress or increase the effectiveness of various components of their colour signals through their behavioural preferences and resting positions. In such a way, the natural selection for a similar colouration can be at least partly relaxed. Such a process also limits the power of natural selection to achieve the close similarity in colours, as imperfectness might not be temporarily observable.

Even with limitations to the colour spectrum visible to humans, our results indicate a variable degree of detectability, i.e., conspicuousness, of patterns in the natural environment^97,98^. The uniform forms are the least apparent, and therefore potentially the least effective^4^ (Table S6). The bicoloured forms display an internal contrast which potentially diminishes the role of the external contrast under some ambient conditions^61^. Using internal contrast, individuals depend to a lesser degree on the choice of leaf on which they sit, including a preference for the upper or bottom side. Other forms display more complex arrangements of differently coloured areas on the dorsum as an additional visual signal component. These patterns can gradually increase signalling effectiveness^4,58^.

Other characteristics of patterns could be considered if a single community was studied: different volumes of brightly coloured areas, body size, and gradual and/or sudden transitions between colours (Figs. S1–S922). Additionally, the high perception of a colour pattern and/or shape should be evaluated under various light conditions and environmental settings^99,100^. The ecology and behaviour of net-winged beetles add further factors which might define the effectiveness of signalling (diurnal activity, time spent on the upper and bottom sides of a leaf, the availability of some plants, the width of their ecological niche, i.e. the diversity of conditions under which a species interacts with potential predators^96^). To complicate the interactions leading to the evolution of multi-pattern communities, predators perceive lycids in different visual spectra, and they have abilities which allow them to learn and forget aversion. They have a differential perception of the salient components of a signal, and there is a potential for neophobia in the interactions with uncommon but highly apparent patterns^37,38,101–104^. These impact of these factors can only be investigated with dedicated experiments.

### Phylogeny and aposematism of net-winged beetles

We have produced a new phylogeny with ~ 570 species from 25 lycid tribes and each tribe is represented proportionally to its diversity (these tribes represent 99.7% of the alpha-diversity^68,105–108^, Fig. 5, Fig. S926). Further, we describe and record the distribution of aposematic patterns in all zoogeographic regions, both sequenced species and a further ~ 4000 species studied in collections (Figs. 1, 2, 3, 4B, Fig. S1–S922). Until recently only a few aposematically coloured net-winged beetles had been reported on in the literature^13,14^ and the present study deals with all known patterns in the family. Based on the amount of data (~ 570 spp. sequenced, ~ 4000 spp. examined), we consider the present phylogenetic hypothesis to be sufficient for the investigation into the origins of aposematic patterns in lycids.

A highly visually conspicuous colouration is common in net-winged beetles, and the presence of various insects which phenotypically resemble them has been noted in the literature^13–15,17,49–51^ (Figs. 1, 2, 3, 4B, Figs. S1–S922). The likely aposematic function of the lycids’ bright colouration has not been experimentally proven for most species, but is supported by the presence of bitter and poisonous compounds in their bodies^23,27,28,52,109^. The phylogenetic relationships suggest that similarly coloured patterns evolved independently (e.g. Fig. 3B,X, Figs. S1–S922). Based on these facts, we assume that unrelated, visually similar, and sympatrically occurring lycids are Müllerian co-mimics. Putative non-lycid co-mimic can also be protected by toxic compounds, and they are considered to be Müllerian mimics, although with unknown relative protection (soldier, blister, and some longhorn beetles). Others are unprotected by toxic compounds and their resemblance can be classified as Batesian mimicry^27,28,52,109^. The net-winged beetles supposedly serve as models in local mimicry rings because they are usually predominant in terms of numbers and their co-mimics display a visual signal which is exceptional for their lineage. These findings make lycids, with > 4000 species, one of the largest aposematically coloured animal lineages. Until now, the largest analysed groups of aposematically coloured insects contained about a hundred species^7,10^.

Net-winged beetles are a relatively ancient lineage and, presumably, they separated from the Iberobaeniidae ~ 177 mya^69^ (Figs. 5, 6, 7). Our results show the early origin of Dexorinae as a sister to other lycids (Figs. 5, 6). Without information on their chemical protection and ecology, the species-poor Dexorinae can possibly be left out as a potentially non-aposematic group^110,111^ (Figs. S383, S384). The representatives of other lycid lineages are unpalatable and most are brightly coloured, and we assume that the origin of aposematically signalling in lycids can be set, at the latest, to ~ 125 mya when the radiation of major, highly diverse lycid lineages started (Fig. 7A, Fig. S925).

We prove that similar aposematic patterns evolved repeatedly in unrelated lineages although weak phylogenetic conservatism was identified (δ-statistics values 2.13–5.90). The indicated level of phylogenetic conservativism must be interpreted cautiously because the colouration is at least partly linked with the geographical origin and large clades from a single area are present in the tree (Fig. S918). Therefore, the δ-statistics value of 5.9 for Metriorrhynchini might also be affected by the geographical structure of the dataset. Conversely, colour conservativism is highly probable in the Dictyopterini and Taphini. Both lineages seldom signal their unpalatability via their bicoloured elytra, even in the ecosystems where the highly conspicuous forms that use such signals dominate (Figs. S6, S176). Our results indicate that the most recent common ancestor of Dictyopterini and Taphini was bright red, and the lineage is ancient (the earliest split in the clade ~ 92 mya). The early origin of aposematism in this clade is supported by the recently discovered mid-Cretaceous net-winged beetles, which are morphologically similar to the extant bright red Erotinae^79,80^.

Further, we suggest the gradual evolution of aposematic signalling. The reconstruction recovers simple, relatively low-contrast, uniformly coloured patterns in an initial phase of lycid diversification (Fig. 7, Fig. S926). We avoid the estimation of the colour tone for the earliest lycids as the shifts between the yellow and dark coloured forms are controlled by the same set of genes, and therefore, they are simple and common^112,113^ and the estimation would be prone to error. We hypothesize subsequent origins of further aposematic patterns such as the contrast between the differently coloured pronotum and the elytra, bicoloured elytra, and various characteristically mottled forms^41^ (Fig. 7, Fig. S926). All unique, uncommon patterns such as punctate, metallic blue, red/blue, green, reticulate, and tricoloured are only present in terminal lineages, mostly in a few separate species. Therefore, we cannot date them with confidence and must surmise their recent origins (Fig. S929).

We assume that the initial dependence on external contrast is supplemented by an internal contrast whose effectiveness should be similar under various ambient conditions. Later, the signalling is enhanced by more complex patterns combining different colours in a characteristic arrangement. We propose that various other conspicuous patterns continuously evolve and persist in net-winged beetles’ rings, regardless of the continuing dominant presence of less conspicuous ancestral forms. A low number of aberrantly coloured aposematics is a disadvantage, so the origin and persistence of new patterns have been discussed by several authors^114–118^.

Gregariousness was proposed as the trait which supported the origin of new highly conspicuous signals^114,115^. Conspicuous new patterns are often displayed by a single species with low abundance in a very restricted area (Figs. S1–S922). Therefore, they cannot assemble their own aggregations and we found them as members of aggregations predominantly formed by other species, which regularly contained several aposematic patterns^9,40,41^. Potentially, all brightly coloured individuals in multi-species aggregations^40,41^ can be collectively perceived as unprofitable or they may emit a smell, even when they are not disturbed^30,36,119,120^. In such a way, participation in an aggregation can enhance the survival of visually aberrant aposematics. Detailed information about these factors is unavailable for the present study which considers several thousand species from all zoogeographical regions and theoretically should have taken into account a similar number of predators^97^, as well as the impact of the presence of imperfect Müllerian and Batesian mimics from other groups of insects^120^. Nevertheless, our results show that a wide spectrum of aposematic signals can evolve within a single lineage of beetles (Table S8), numerous patterns are present within a single area (Table S7), differently coloured unprofitable prey co-exist in close interaction, and low contrast patterns, transitional colouration, and imperfect mimics regularly occur alongside highly conspicuous forms (Figs. S1–922).

As a result, the interactions of multi-species and multi-pattern assemblages with various local predators are possibly very complex^96^. For example, the predators’ learning may be affected by the complexity of local mimetic rings, and the fluctuations in their composition. Conversely, the effectiveness of the aposematic signalling will reciprocally depend on the dynamically changing spectrum of predators, both in time and space. At least some predators should be able to enhance the avoidance of aposematic prey by social learning^121,122^, but such a factor is limited to birds and cannot be expected in various invertebrates, which are putatively important predators of small-bodied lycids.

Strictly speaking, a pattern displayed by a single species is not a case of mimicry. But, if such a species is unpalatable, emits an aposematic signal, and occurs sympatrically with other unprofitable prey, it contributes to the predators’ perception of the whole community, and over the course of time the single-species pattern might be adverged as a model by co-mimics. Therefore, we propose a potential role for the multi-pattern environment in the evolution of new patterns^117^. Under such conditions, prior experience with the unprofitability of less conspicuous prey produces a positive bias for avoidance learning of more conspicuous defence signals^123^. The learning process needs high frequency encounters and possibly that is one of the reasons why New Guinea and the Colombian-Ecuadorian Andes, where lycids are highly diverse and common, are apparent hot-spots of aposematic signal diversity (Fig. 8, Fig. S1–S922). The observed persistence of multi-pattern communities may also be supported by phylogenetic conservativism, high levels of migration between local mimetic assemblages, the diversity of predators, and microhabitat diversity in mountain ecosystems, but the evaluation of these factors will need differently designed research.

### Aposematic patterns and diversity

We assume that there are several potential predictors of the diversity of aposematic signalling in a region: phylogenetic diversity, alpha-diversity, and abundance. Further factors include the ecosystem diversity^62,124,125^ and genetic constraints^126^ on which we do not have sufficient information. The evaluation of considered factors depends on the availability of data. Although we can reliably record the number of tribes occurring in a region (as an indicator of phylogenetic diversity), we currently do not have data on the phylogenetic structures of net-winged beetle communities. Similarly, our estimation of alpha-diversity depends on the number of species already described^11^ and these are only a part of the real diversity^44,84^. Additionally, we dependent on an approximate estimation of abundance in various ecosystems (see “Methods”).

The highest phylogenetic diversity was identified in the Oriental region (the Greater Sundas and Indo-Burma, 18 and 17 tribes, respectively) and in Eastern Asia (15 tribes; Fig. 8A). Nevertheless, the number of aposematic patterns is moderate in these regions (6–10 patterns, usually under five patterns in a location; Fig. 8C, Table S7). We only identified some endemic aberrant patterns in the high mountains of the Greater Sundas^41,44^. In contrast with the low phylogenetic diversity, the highest numbers of patterns were recorded in New Guinea (only four tribes, with Metriorrhynchini predominant, but 22 recorded patterns, Tables S7, S8) and the northern Andes (five tribes recorded, three of them dominant; 14 patterns, Table S7). The low correlation between phylogenetic diversity and the number of aposematic patterns is shown in Fig. S924 (R^2^ = 0.057).

We can consider alpha-taxonomic diversity in separate regions (Table S7, Fig. 8B, Fig. S924). If we compare the number of species and the number of patterns, we identify an expected positive slope, but with a low fit of the model (R^2^ = 0.23), although the interpretation is limited by the present state of the biodiversity inventory^84,85,127,128^. If we consider the real number of species identified in New Guinea (~ 1300 spp., unpublished data), the R^2^ value doubles. Although we can keep the working hypothesis that the number of aposematic patterns and the number of species are positively correlated, the evidence is ambiguous and only future studies of a high number of mimetic communities where individuals are in real contact can produce reliable data.

Abundance is another factor, although it is potentially linked with alpha-diversity. Currently we do not have hard data to evaluate the abundance of net-winged beetles in various regions and, preferably, individual mimicry communities should be the subject of analyses rather than regions, as in this study. An estimation can only be based on typical collecting success in various regions, and then we could see that regions where net-winged beetles are most common also have the highest diversity of aposematic signals, i.e. New Guinea, the northern Andes, and South East Asian mountains such as Sumatran volcanoes and Mt. Kinabalu in Borneo.

## Conclusion

The main goals of our study were to assemble information on the aposematic patterns of > 4000 net-winged beetles, investigate the link between patterns and phylogenetic relationships, and identify the factors affecting aposematic signalling.

We have documented numerous aposematic signals (Figs. 1, 2, 3, 4, Figs. S4–S922). The patterns displaying internal contrast and the complex arrangement of differently coloured patches evolved from simple uniformly coloured forms (Fig. 7). Further, we show that the most conspicuous patterns evolved in limited areas from their aposematic relatives and are displayed by a limited number of species. Despite conspicuousness and putative high level of protection, they sympatrically occur with their less conspicuous ancestral forms, and the local faunas regularly contain a relatively high number of imperfect mimics and transitional forms (Figs. S1–S922). As a result, predator avoidance learning is not a simple choice between cryptic and aposematic prey, but each of many predators has to discriminate between cryptic prey and the whole spectrum of aposematic net-winged beetles in a ring which also contains Batesian and Müllerian co-mimics^9^.

We assume that regional signalling diversity will be taken in account when specific lycid mimetic complexes are studied in the future. We found the highest number of aposematic patterns in the areas with very high alpha-diversity and abundance but low phylogenetic diversity (New Guinea, the northern Andes), as well as in areas with moderate abundance but high phylogenetic diversity (South East Asia as the whole). The relative importance of these factors needs further study limited to a strictly defined community of interacting mimics and predators. The lycid colour patterns are so diverse in some regions that continuous migration could affect convergence to a common signal. Although lycids species often occur in multi-species communities, it is possible that some of them face a different spectrum of predators and so their patterns are a compromise, which indicates the presence of more complex conditions than the traditionally considered simple prey/predator pairs.

## Supplementary Information

Supplementary information.
