# Supplementary material for: Conspicuousness, phylogenetic structure, and origins of Müllerian mimicry in 4000 lycid beetles from all zoogeographic regions
Source: Sci Rep. 2021 Mar 16;11:5961. doi: 10.1038/s41598-021-85567-x (PMC7971032; doi:10.1038/s41598-021-85567-x)
Supplement: Supplementary file 1 — Supplementary information. [file 41598_2021_85567_MOESM1_ESM.pdf]

Conspicuousness, phylogenetic structure, and origins of Müllerian mimicry in 4,000 lycid beetles from all zoogeographic regions

Michal Motyka, Dominik Kusy, Michal Masek, Matej Bocek, Yun Li, Renata Bilkova, Josef Kapitan, Takashi Yagi, Ladislav Bocak

Supporting information

Supplementary text

Table S1. The colour patterns of net-winged beetles.
Table S2. The list of the samples used for phylogenetic reconstruction and GenBank accession numbers.
Table S3. The partition scheme, the best substitution models and information about IQ-tree run.
Table S4. The list of samples with details of colour patterns.
Table S5. Internal and external contrasts in nature.
Table S6. Internal and external contrast of typical representatives of net-winged beetle patterns. DeltaE values were counted as Euclidian distances between colours in the L*a*b CIE 76 colour space.
Table S7. The distribution of the colour patterns.
Table S8. The summary of the colour patterns recorded in lycids tribes.

Figures S1–S69. The overview of the lycid aposematic patterns and co-mimics: Oriental region: Indo-Burma.
Figures S70–S95. The overview of the lycid aposematic patterns and co-mimics: Oriental region: Southern India, and Sri Lanka.
Figures S96–S296. The overview of the lycid aposematic patterns and co-mimics: Oriental region: The Greater Sundas, and the Philippines.
Figures S297–S397. The overview of the lycid aposematic patterns and co-mimics: Afrotropical region: Continental Africa, the southern part of the Arabian Peninsula, and Madagascar.
Figures S398–S484. The overview of the lycid aposematic patterns and co-mimics: Palearctic region: Eastern Asia.
Figures S485–S498. The overview of the lycid aposematic patterns and co-mimics: Palearctic region: Europe, northern Africa, Asia Minor.
Figures S499–S537. The overview of the lycid aposematic patterns and co-mimics: Mesoamerica, and the Caribbean.
Figures S538–S653. The overview of the lycid aposematic patterns and co-mimics: Neotropical region.
Figures S654–S660. The overview of the lycid aposematic patterns and co-mimics: Nearctic region.
Figures S661–S655. The overview of the lycid aposematic patterns and co-mimics: Australian region: The Wallacea.
Figures S656–S844. The overview of the lycid aposematic patterns and co-mimics: Australian region: The Lesser Sundas, New Guinea.
Figures S845–S914. The overview of the lycid aposematic patterns and co-mimics: Australian region: Continental Australia.
Figure S915. The guiding phylogenomic topology for the maximum likelihood estimation.
Figure S916. Alpha diversity, phylogenetic diversity, area size and the numbers of endemic aposematic patterns.
Figure S917. Time-calibrated, maximum clade credibility tree computed using BEAST.
Figure S918. The reconstruction of the ancestral colour patterns using BEAST and constrained topology from the IQ-Tree analysis.
Figure S919. Colour patterns recorded in lycid subfamilies.
Figure S920. The dominant dorsal colouration in net-winged beetles.
Figure S921. The dating of the origin of aposematic patterns.

Supplementary text

Additional information on field research. The research was conducted throughout the World by all authors of the study: the Palearctic region (central Europe, Japan, China; MMo, MMa, YL, LB), the Oriental region (the Great Sundas, the Malay Peninsula, the Philippines; MMo, DK, MB, RB, LB), the Australian region (the Wallacea, the Lesser Sundas, New Guinea, eastern Australia; MMo, MMa, DK, LB), the Afrotropical region (Cameroon, Ethiopia, Tanzania; LB), the Nearctic region (the eastern U. S. A., Rocky Mountains; LB), and the Neotropical region (Dominican Republic, Ecuador; LB). Further specimens used for pattern descriptions and distribution were obtained from various colleagues and all voucher specimens are deposited in the mounted collection of the senior author's laboratory.

Information on the research in museum collections. We obtained further data on net-winged beetle aposematism by the extensive search in world collections. Major collections with a high proportion of the primary types and worldwide coverage were studied by the first and last author in the Museum and Institute of Zoology in Warsaw (coll. R. Kleine), the National Museum of Natural History in Paris (coll. M. Pic, J. Bourgeois, and L. Fairmaire), and the Natural History Museum in London (the species described by C. O. Waterhouse). Further rich material of net-winged beetles was studied in the Naturalis Museum, Leiden (including the recently transferred collection of the Amsterdam University), Museum of Natural History in Brussels (coll. Guérin-Méneville), Museum Alexander Koenig in Bonn (coll. J. Klapperich), Bavarian State Collection of Zoology, Munich, Senckenberg Natural History Collections, Dresden (coll. Kirsch), Museum of the Moscow State University (coll. Motschulsky), Natural History Museum in St. Petersburg (coll. V. V. Barovsky), Natural History Museum in Vienna, Natural History Museum in Basel (coll. Wittmer), Natural History Museum in Geneva, Museum of the Hokkaido University in Sapporo (coll. T. Nakane, K. Ohbayashi, and other Japanese entomologists), the Queensland Museum in Brisbane, Natural History Museum in Berlin, Stuttgart State Museum of Natural History, National Museum in Prague, Hungarian Natural History Museum, Budapest, Entomological Collection of the University of the Philippines, Los Baños (coll. C. F. Baker), Indonesian Institute of Sciences (LIPI) in Cibinong, Zoological Museum of the National University of Singapore, and the American Museum of Natural History, New York. Some photographs were taken from the collection of O. Konvička (Czech Republic).

Addtional information on the estimation of regional abundance. The estimation is based on the average numbers of individuals collected by a field researcher per month in a region. The research was primarily focused on primary ecosystems never or only moderately modified by human activities. Such ecosystems are the most species rich. We do not include in our average numbers of collected specimens the occasional aggregation of a single or a few species in secondary ecosystems.

Species of some genera, such as *Lipernes* and *Lycostomus* in China and South East Asia, *Lycus* in Sub-Saharan Africa, *Lygistopterus* in Europe, *Rhyncheros*, *Celiasis*, and *Calopteron* in the USA and South America, Calochromini in the semidry regions of India, *Metriorrhynchus* and *Leptotrichalus* in the Philippines and *Metriorrhynchus* and *Porrostoma* in Eastern Australia are sometimes found in masses in flowers, but their occurrence is

temporal, they are most common in the secondary habitats or semidry ecosystems where low numbers of net-winged beetles occur. We observed that in all regions these species do not occur in primary habitats even if these are very close. For example, high numbers of *Lipernes* were occasionally observed in carrot flowers in the fields of local farmers in SE Asia, but none or a few individuals were collected in humid mountain forests adjacent to the fields. As these net-winged beetles do not interact with local mimics in the forest, we do not consider them in our regional abundance estimation.
Information on the authorship of the photograps. The photographs of the dry mounted long-horn beetles were provided by Lubos Dembicky (Brno), the photographs of African Hispa sp. was provides by Alain Coache (La Brillanne) and all photographs of the South American co-mimics were provided by Jim McClarin (Cosanga)

The detailed overview of aposematic patterns in Lycidae

Uniform upper body side
The monochromatic patterns are common in net-winged beetles and include uniform black forms with low external contrast especially on the upper leaf side (>500 spp., EC-DeltaE/Upper 23–32, EC-DeltaE/Bottom 29–45, average 32; various Platerodini, Metriorrhynchini, etc. from the majority of regions; Fig. 2S). [Note. The uniform black pattern is considered among aposematic patterns as black coloured individuals are highly conspicuous especially on the bottom side of a leaf and their body size and silhouette are commonly copied by other beetles and some small moths]. Further, we recorded the species with the uniform yellow dorsum with high contrast, especially on the upper leaf side (>500 spp.; EC-DeltaE/Upper 43–67, EC-DeltaE/Bottom 30–53, average 47; e.g. Platerodini, Metriorrhynchini, mainly from Afrotropical and Oriental regions; e.g. Fig. 3U), the uniform orange pattern (e.g. *Platycis schneideri*, Erotini in Caspian broad-leaf forests, *Metriorrhynchus* in New Guinea, Fig. 3P), and the bright red pattern with high contrast on both sides of a leaf (~200 spp.; EC-DeltaE/Upper 40–67, EC-DeltaE/Bottom 40–68, average 53; most Dictyopterini in the Holarctic region, Calochromini in Yunnan and Sichuan mountains, some Dilophotini and Macrolycini; e.g. Figs. 2A, D, E, I, 3P). The tone of bright colours is variable and, additionally, the external contrast depends on the colour of a leaf. Nevertheless, we can generally say that the external contrast of bright forms is ~1.35 higher than those of the dark coloured ones (Figs. 2I, J). Some Calochromini, and Dictyopterini commonly sit on dead or burned wood and are highly apparent (Figs. 2A, D; EC-DeltaE 48–60). The metallic uniform colouration is rare and has low external contrast on the upper side of a leaf and slightly higher contrast on the bottom side (<50 spp., some *Idiopteron* in the Nearctic region, *Diatrichalus* and *Cladophorinus* in New Guinea; Fig. 2L, EC-DeltaE/Upper 22–40, EC-DeltaE/Bottom 22–52, average 38).

The patterns with bicoloured upper body side
The bicoloured forms signal their presence by the internal contrast between bright and dark coloured body parts (average IC-DeltaE 57) and the external contrast between respective body parts and the background. Their internal contrast is generally higher than the external contrast between a bright body part and the background (average EC-DeltaE 50). The external contrast between a dark coloured body part and background is low (average EC-DeltaE 36). The principal component of the aposematic signal is the internal contrast as summarized in Table S6.

a/ Differently coloured pronotum and elytra. This pattern is usually produced by a combination of the black pronotum and red elytra, sometimes the pronotum has bright margins (>100 spp., often Dictyopterini, Erotini, Lyponiini, Macrolycini; e.g. Figs. 2B, J, 3K; IC-DeltaE 46–52). Less common are the combinations of the black pronotum and yellow elytra (<50 spp., some African Platerodini, and Metriorrhynchini) and a reversed pattern with the brightly coloured pronotum and black elytra (some Metriorrhynchini and Platerodini in New Guinea, *Macrolygistopterus ruficollis* in North America, *Dictyoptera elegans* in Japan). The red/metallic pattern is limited to Sulawesi (>10 spp. of *Metriorrhynchus*, *Calochromus*, and *Plateros*; Figs. 1G, H; IC-DeltaE 62–76).

b/ The colour difference between elytral humeri and apex. The signal characterized by bicoloured elytra is common and widespread. Most species have a brightly, usually the yellow, brown, or red coloured humeral part of elytra and the dark coloured apex (altogether >800 spp.). The individuals with a high IC are mostly found in Metriorrhynchini, Lycini and Platerodini, but the pattern is found in most lineages. The extend of differently coloured parts is variable and the here defined pattern contains several fairly distinct signals (e.g. Figs. 2G, N, P–R, 3D, E, N, O, V). The pattern contains the types with a low internal contrast (e.g. Ateliini: *Scarelus*, Figs. 2G; IC-DeltaE 27.7) as well as the high-contrast types (various Metriorrhynchini; Figs. 3D, R; IC-DeltaE 77–80; New Guinean *Ditua* with bright red/velvet black coloured dorsum, IC-DeltaE 72; yellow/black African *Cautires* IC-DeltaE 72, and Lycini such as yellow/black *Lycus* spp., Figs. 2N, P; IC-DeltaE 69–72). Only a very limited number of species has dark coloured humeri and the brightly coloured apical part of elytra (Fig. 3G). These patterns are usually very conspicuous due to the high contrast (IC-DeltaE 65–79). The colouration of the pronotum is not considered in this pattern and variants include the species with a bright coloured as well as dark pronotum. Therefore, the variants can be perceived as distinct aposematic signals in some mimetic complexes. A unique pattern is shown by Thonalmini (only *Thonalmus*, ~10 spp., endemic to the Caribbean) which are all bright red coloured with metallic blue elytral apex (Fig. 2T). The pattern of *Thonalmus* is exceptional due to internal contrast slightly lower than the external one when we measured it using the photograph taken in the field (IC-DeltaE 83, EC-DeltaE 92). In most species, the external contrast of the brightly coloured body parts is lower and reach ~80% of the internal contrast (average EC-DeltaE/bright 54). The dark coloured parts contribute to the conspicuousness of an individual only moderately (~55% of the internal contrast).

Fasciate elytral patterns

The fasciate type contains two basic forms – either the elytral apex and humeri are dark coloured and a middle part of the elytron is yellow (New Guinean Metriorrhynchini, e.g. Figs. 3I, 12O, Neotropical and Nearctic Calopterini, Figs. 10J, K, O, IC-DeltaE 52–71) or a black band occupies the middle part of the elytron and humeri and the apex are brightly coloured (e.g. some New Guinean Metriorrhynchini, Figs. 2M, 3Q, Calopterini, Fig. 10Y, IC-DeltaE 68–75). Only a few species have two light bands in elytra (Calopterini, Figs. 9L, 10Z, AA; Lycini, Fig. 8D), the humeral one might be incomplete (Calopterini, Fig. 10O). Further, some species of Neotropical Calopterini (some *Calopteron* spp., *Idiopteron* spp.) are large-bodied and have considerably dilated posterior part of elytra which differs from small-bodied, slender forms in general appearance (compare Figs. S590, S592, S593 and Figs. S598–S601, IC-DeltaE 54–75). The dark coloured part of elytra has a metallic blue appearance in some *Calopteron* (Figs. 10R).

The internal contrast is variable, moderate if the surface of elytra is only sparsely pubescent (Fig. 2K, IC-DeltaE 70) to extremely high if the black colour is produced by the dense black velvet pubescence (Fig. 3Q, IC-DeltaE 93). Generally, the internal contrast of this pattern is higher than the external one and characteristic bands are conspicuous and easy to be remembered for a human observer. The fasciate forms are dominant in the Neotropical region (Calopterini, Platerodini, Eurrhacini), some Lycini display this pattern in savannah ecosystems of Sub-Saharan Africa (Fig. 2D), few Metriorrhynchini display the variant of the fasciate pattern in the Wallacea and New Guinea, but not in the Oriental and Afrotropical regions (Figs. S675–S679, S712–S714, S822).

Striate bright and dark elytral patterns

The striate pattern is uncommon, and most species are known from the Neotropical region (Figs. 10L–N). Under the striate pattern are included also forms with prolonged numeral patches (Fig. 10W), and differently coloured costae and intercostal spaces (10D, T). Only a few species classified as striped are known from the Oriental region (some Dilophotini and Platerodini; Fig. 7J). Similarly, a low number of species has the dark middle part of elytra and brightly coloured lateral margins (Figs. 12C, D, H). The conspicuousness of the patterns can be very low as in the Oriental representatives (Fig. 7J) or high, e.g. South American Calopterini (Fig. 10N). The internal contrast reaches IC-DeltaE 52–65. If multiple stripes are present in each elytron, the internal contrast is low (Fig. 7J) and reaches higher values only in Neotropical *Idiopteron* sp. (EC-DeltaE 50).

Punctate bright and dark elytral patterns

The punctate pattern is very characteristic and easy to remember to a human observer, but the number of species with clearly delimited elytral patches is very low. Some Australian *Enylus* display an intraspecific polymorphism and the light-coloured individuals have dark strips reduced to unclear patches (Fig. 13B). Distinct brightly coloured patches on the dark background are found in Calopterini from Peru (Figs. 10E, F). The highest internal contrast was measured for a Himalayan *Parantis* (Fig. 2C, IC-DeltaE 84).

Tricoloured dorsum

The combination of three distinct colours in the upper side of an individual is uncommon and only a few cases have been reported from New Guinea (Figs. 3F, 12D). The contrast between differently coloured elytral humeri and the apex is high (IC-DeltaE 60, *Cladophorus* sp., Fig. S791) or low (IC-DeltaE 28, Fig. 10F), but these patterns are unique in general appearance and putatively easy to remember (Fig. 3F). The variants are displayed by a single lycid species and no co-mimics were identified.

Reticulate elytral patterns

The reticulate patterns are highly apparent against any background but were noted in a few species. Several species with dense bright pubescence on at least the humeral part of elytral costae were found in the higher elevations of the Kinabalu massif in north-eastern Borneo (Figs. 7R, S), eventually, the signal is produced by differently coloured setae in a calochromine species which does not possess transverse costae in elytra (*Micronychus* sp.[41], Fig. 7O). Further, some *Calopteron* have a similar structure of costae in the humeral part (Fig. 10V) or the apical part of the elytra (Fig. 10S). Species with an intermediate cell volume were reported in both regions (Fig. 10C).

Non-categorised patterns

Several colour patterns do not fit in any of the above-listed categories. These were recorded in New Guinea where we identified the highest diversity of colour patterns. As examples, we present white/yellow combination characterized by very low internal contrast (Fig. 11V, IC-DeltaE 36), black/green (Figs. S818; IC-DeltaE 47), orange/green (Figs. 12I, J, IC-DeltaE 24–30), a red/blue pattern (Fig. 12K, IC-DeltaE 42) and unique green uniform colouration with extremely low contrast on leaves in the visible spectrum (Fig. 12L, EC-DeltaE/Upper 27, EC-DeltaE/Bottom 15). Several species have a characteristic densely white pubescent pronotum (Figs. 3B, 3X, 12D, N). Most of these patterns are represented by a single lycid species, at least concerning the present knowledge, and we have not identified any co-mimics from other beetle groups.

Table S1. The colour patterns of net-winged beetles.

Pattern	Description	Abbreviation	Simplified category
**Uniform coloration**	uniform black	Uni-Black	Uni
	uniform yellow	Uni-Yellow	Uni
	uniform orange/red	Uni-Red	Uni
	uniform metallic (blue, green)	Uni-Metal	Uni
**Bi-colored: pronotum / elytra**	black pronotum / yellow elytra	B1-Black/Yellow	PvE
	black pronotum / red-orange elytra	B1-Black/Red	PvE
	bright pronotum / black elytra	B1-Bright/Black	PvE
	red pronotum / metallic elytra	B1-Red/Metalic	PvE
**Bi-colored: elytra**	yellow humeri / black apex	B2-Yellow/Black	BiElyt
	red-orange humeri / black apex	B2-Red/Black	BiElyt
	black humeri / bright yellow-red-orange apex	B2-Black/Bright	BiElyt
**Fasciate elytra**	humeri & apex black / middle yellow	Fas-mid-Yell	Fasci
	humeri & apex bright / middle black	Fas-mid-Black	Fasci
	humeri & apex metallic / middle yellow	Fas-metal-mid-Yell	Fasci
**Striate elytra**	elytra brown/brightly coloured, suture or middle of elytron black	Stria	Stria
**Punctate elytra**	elytra brown/brightly coloured, black patch in each elytron	Punct	N-Cat
**Tricolored elytra**	elytron with three differently coloured parts	Tricolor	N-Cat
**Reticulate elytra**	background colour of elytra dark, costae large, brightly coloured	Reti	N-Cat
**Non-categorized**	see listed examples for further information	Non-Cat	N-Cat

Table S2. The list of the samples used for phylogenetic reconstruction and GenBank accession numbers.

**Outgroup**										
**Tribe**	**Genus**	**Species**	**Voucher**		**Location**	**18S**	**28S**	**16S**	**COI**	**NADH5**
Iberobaenini	Iberobaenia	minuta	UPOL	RK0790	Spain	KT339296	KT339297	KT825140	KT825141	KT825142
**Ingroup**										
**Tribe**	**Genus**	**Species**	**Voucher**		**Location**	**18S**	**28S**	**16S**	**COI**	**NADH5**
Alyculini	Alyculus	kurbatovi	UPOL	000543	Java	DQ181072	DQ181146	DQ180998	DQ181220	DQ181374
Antennolycini	Microlyropaeus	dembickyi	UPOL	000542	Sumatra	DQ181071	DQ181145	DQ180997	DQ181219	DQ181373
Antennolycini	Antenolycus	constrictus	UPOL	000L22*	Malaysia	DQ181051	DQ181125	DQ180977	DQ181199	DQ181353
Ateliini	Scarelus	sp.	UPOL	000582	Borneo	DQ181085	DQ181159	DQ181011	DQ181233	DQ181387
Ateliini	Scarelus	sp.	UPOL	000583	Borneo	DQ181086	DQ181160	DQ181012	DQ181234	DQ181388
Ateliini	Scarelus	sp.	UPOL	000L15	Borneo	DQ181046	DQ181120	DQ180972	DQ181194	DQ181348
Ateliini	Atelius	sp.	UPOL	VK0733	China	–	–	KT752146	KT751822	KT751975
Ateliini	Scarelus	pseudoumbosus	UPOL	VM0002	Malaysia	HM451121	HM451079	HM450999	HM451038	HM451207
Ateliini	Scarelus	pahangensis	UPOL	VM0004	Malaysia	HM451123	HM451081	HM451000	HM451040	HM451209
Ateliini	Scarelus	similis	UPOL	VM0005	Borneo	HM451124	HM451082	HM451001	HM451041	HM451210
Ateliini	Scarelus	anthracinus	UPOL	VM0006	Malaysia	HM451125	HM451083	HM451002	HM451042	HM451211
Ateliini	Scarelus	cibodasensis	UPOL	VM0007	Java	HM451126	HM451084	HM451003	HM451043	HM451212
Ateliini	Scarelus	saranganensis	UPOL	VM0011	Java	HM451130	HM451087	HM451005	HM451046	HM451216
Ateliini	Scarelus	brastagiensis	UPOL	VM0012	Sumatra	HM451131	HM451088	HM451006	HM451047	HM451217
Ateliini	Scarelus	loksadoensis	UPOL	VM0014	Borneo	HM451133	–	HM451008	HM451049	–
Ateliini	Scarelus	baranciki	UPOL	VM0019	Borneo	HM451137	HM451093	HM451012	HM451051	–
Ateliini	Scarelus	nigricornis	UPOL	VM0021	Borneo	HM451139	HM451095	HM451014	HM451053	HM451222
Ateliini	Scarelus	emasensis	UPOL	VM0022	Borneo	HM451140	–	HM451015	HM451054	HM451223
Ateliini	Scarelus	sanguineus	UPOL	VM0023	Sumatra	HM451141	HM451096	HM451016	HM451055	HM451224
Ateliini	Scarelus	longicornis	UPOL	VM0026	Sumatra	HM451144	HM451099	HM451018	HM451057	HM451227
Ateliini	Scarelus	flavicolis	UPOL	VM0031	Sumatra	HM451149	HM451104	HM451021	HM451061	HM451232
Ateliini	Scarelus	ruficolis	UPOL	VM0033	Sumatra	HM451151	HM451106	HM451023	HM451063	HM451234
Ateliini	Scarelus	rufus	UPOL	VM0034	Sumatra	HM451152	HM451107	HM451024	HM451064	HM451235
Ateliini	Scarelus	crudus	UPOL	VM0049	Philippines	HM451165	HM451118	HM451035	HM451076	HM451248
Ateliini	Scarelus	salvani	UPOL	VM0050	Philippines	HM451166	HM451119	HM451036	HM451077	HM451249
Calochromini	Calochromus	sp.	UPOL	000033	Borneo	DQ181060	DQ181134	DQ180986	DQ181208	DQ181362
Calochromini	Calochromus	sp.	UPOL	000124	Borneo	DQ181061	DQ181135	DQ180987	DQ181209	DQ181363
Calochromini	Calochromus	sp.	UPOL	000347	Borneo	DQ181068	DQ181142	DQ180994	DQ181216	DQ181370
Calochromini	Calochromus	sp.	UPOL	000L16	China	DQ181047	DQ181121	DQ180973	DQ181195	DQ181349
Calochromini	Calochromus	sp.	UPOL	A00477	Malaysia	KT752159	KT752318	KT751987	KT751669	KT751829
Calochromini	Calochromus	sp.	UPOL	A00617	Laos	KT752282	KT752446	KT752115	KT751789	KT751948
Calochromini	Calochromus	sp.	UPOL	MT0001	China	KU496008	KU496036	KU495936	KU496068	KU496184
Calochromini	Calochromus	sp.	UPOL	MT0002	Borneo	KU496017	KU496038	KU495939	KU496070	KU496152
Calochromini	Calochromus	sp.	UPOL	MT0003	Laos	–	–	KU495998	KU496137	KU496196
Calochromini	Calochromus	sp.	UPOL	MT0004	Malaysia	KU496032	KU496060	KU495992	KU496122	KU496187
Calochromini	Calochromus	sp.	UPOL	MT0005	Sumatra	–	–	KU495942	KU496073	KU496155
Calochromini	Calochromus	sp.	UPOL	MT0006	China	–	–	KU495957	KU496079	KU496174
Calochromini	Calochromus	sp.	UPOL	MT0007	Borneo	–	–	KU495940	KU496071	KU496153
Calochromini	Calochromus	sp.	UPOL	MT0008	Laos	–	–	–	KU496118	KU496163
Calochromini	Calochromus	sp.	UPOL	MT0009	India	–	–	KU495960	–	KU496175
Calochromini	Calochromus	sp.	UPOL	MT0010	Malaysia	–	–	KU495994	KU496123	KU496188
Calochromini	Calochromus	sp.	UPOL	MT0011	Malaysia	–	–	KU495938	KU496100	KU496186
Calochromini	Calochromus	sp.	UPOL	MT0012	Borneo	KU496024	KU496045	KU495952	KU496082	KU496148
Calochromini	Calochromus	sp.	UPOL	MT0013	India	–	–	KU495937	KU496069	KU496185
Calochromini	Calochromus	sp.	UPOL	MT0014	Malaysia	KU496009	KU496037	KU495941	KU496072	KU496154
Calochromini	Calochromus	sp.	UPOL	MT0015	India	–	–	–	KU496086	KU496164
Calochromini	Calochromus	sp.	UPOL	MT0016	India	–	–	–	KU496087	KU496166
Calochromini	Calochromus	sp.	UPOL	MT0017	India	KU496014	KU496049	KU495968	KU496112	KU496172
Calochromini	Micronychus	sp.	UPOL	MT0019	RSA	KU496010	KU496051	KU495971	KU496109	KU496202
Calochromini	Micronychus	sp.	UPOL	MT0020	Zambia	–	–	KU495973	KU496101	KU496205
Calochromini	Micronychus	sp.	UPOL	MT0021	Zambia	–	–	KU495974	KU496102	KU496170
Calochromini	Micronychus	sp.	UPOL	MT0022	RSA	–	–	KU495977	KU496115	KU496206
Calochromini	Micronychus	sp.	UPOL	MT0023	Kenya	–	–	KU495972	KU496116	KU496171
Calochromini	Calochromus	sp.	UPOL	MT0025	PNG	KU496035	KU496067	–	KU496129	KU496189
Calochromini	Calochromus	sp.	UPOL	MT0026	India	KU496012	KU496046	KU495963	–	–
Calochromini	Micronychus	sp.	UPOL	MT0027	Kenya	–	–	KU495975	KU496110	KU496204
Calochromini	Micronychus	sp.	UPOL	MT0028	RSA	–	–	–	KU496144	–
Calochromini	Micronychus	sp.	UPOL	MT0029	RSA	–	–	–	KU496140	KU496207
Calochromini	Lygistopterus	sp.	UPOL	MT0030	Greece	KU496025	KU496053	KU495979	KU496120	KU496182
Calochromini	Calochromus	sp.	UPOL	MT0031	India	–	–	–	KU496088	KU496165
Calochromini	Calochromus	sp.	UPOL	MT0032	Sumatra	KU496027	KU496063	KU496001	KU496130	KU496190
Calochromini	Micronychus	sp.	UPOL	MT0033	Kenya	–	–	KU495976	KU496111	KU496203
Calochromini	Calochromus	sp.	UPOL	MT0034	Laos	–	–	KU495953	KU496083	KU496149
Calochromini	Calochromus	sp.	UPOL	MT0035	Sumatra	–	–	KU495985	KU496105	KU496211
Calochromini	Calochromus	sp.	UPOL	MT0036	Laos	–	–	KU495999	KU496138	KU496195
Calochromini	Calochromus	sp.	UPOL	MT0037	Malaysia	–	–	KU495948	KU496078	KU496161

Calochromini	Calochromus	sp.	UPOL	MT0038	China	–	–	KU495980	–	–
Calochromini	Calochromus	sp.	UPOL	MT0039	Cambodia	–	–	KU495995	KU496125	
Calochromini	Calochromus	sp.	UPOL	MT0040	Malaysia	–	–	KU495954	KU496084	KU496150
Calochromini	Calochromus	sp.	UPOL	MT0041	Borneo	–	–	KU496002	KU496134	KU496192
Calochromini	Calochromus	sp.	UPOL	MT0043	Malaysia	–	–	KU495955	KU496085	KU496151
Calochromini	Calochromus	sp.	UPOL	MT0044	Borneo	–	–	KU496003	KU496135	KU496193
Calochromini	Calochromus	sp.	UPOL	MT0045	Borneo	–	–	KU496004	KU496136	KU496194
Calochromini	Calochromus	sp.	UPOL	MT0046	Laos	–	–	KU496005	KU496131	–
Calochromini	Calochromus	sp.	UPOL	MT0047	Laos	KU496029	KU496065	KU496000	KU496139	KU496197
Calochromini	Calochromus	sp.	UPOL	MT0048	China	–	–	KU495986	KU496104	KU496210
Calochromini	Calochromus	sp.	UPOL	MT0049	Malaysia	KU496018	KU496039	KU495945	KU496075	KU496158
Calochromini	Calochromus	sp.	UPOL	MT0050	Laos	–	–	KU495949	KU496106	KU496146
Calochromini	Calochromus	sp.	UPOL	MT0051	Laos	KU496020	KU496040	KU495946	KU496077	KU496160
Calochromini	Calochromus	sp.	UPOL	MT0052	Laos	–	–	KU495991	KU496090	KU496169
Calochromini	Calochromus	sp.	UPOL	MT0053	China	–	–	KU495958	KU496080	–
Calochromini	Calochromus	sp.	UPOL	MT0054	Laos	KU496028	KU496064	KU496006	KU496132	–
Calochromini	Calochromus	sp.	UPOL	MT0055	Malaysia	–	–	KU495950	KU496107	KU496147
Calochromini	Calochromus	sp.	UPOL	MT0056	China	–	–	KU495964	KU496094	–
Calochromini	Calochromus	sp.	UPOL	MT0057	Malaysia	KU496019	KU496041	KU495947	KU496076	–
Calochromini	Calochromus	sp.	UPOL	MT0058	Cambodia	–	–	KU496007	KU496133	KU496191
Calochromini	Calochromus	sp.	UPOL	MT0059	Malaysia	KU496021	KU496044	KU495943	KU496141	KU496156
Calochromini	Micronychus	sp.	UPOL	MT0060	RSA	KU496011	KU496052	–	KU496142	KU496201
Calochromini	Calochromus	sp.	UPOL	MT0061	China	–	–	KU495988	KU496143	KU496200
Calochromini	Calochromus	sp.	UPOL	MT0062	India	KU496013	KU496048	KU495961	KU496092	KU496176
Calochromini	Calochromus	sp.	UPOL	MT0063	India	–	–	KU495962	KU496093	KU496177
Calochromini	Calochromus	sp.	UPOL	MT0064	India	KU496023	KU496042	–	KU496089	KU496167
Calochromini	Calochromus	sp.	UPOL	MT0065	India	–	–	KU495966	KU496091	KU496178
Calochromini	Calochromus	sp.	UPOL	MT0066	India	–	–	KU495967	KU496119	KU496209
Calochromini	Calochromus	sp.	UPOL	MT0067	India	KU496016	KU496050	KU495969	KU496113	KU496208
Calochromini	Calochromus	sp.	UPOL	MT0068	Malaysia	KU496022	KU496043	KU495944	KU496074	KU496157
Calochromini	Calochromus	sp.	UPOL	MT0069	Malaysia	KU496033	KU496061	KU495993	KU496124	–
Calochromini	Calochromus	sp.	UPOL	MT0070	Laos	KU496030	KU496058	KU495951	KU496108	KU496145
Calochromini	Calochromus	sp.	UPOL	MT0071	India	–	–	KU495956	KU496103	KU496168
Calochromini	Calochromus	sp.	UPOL	MT0072	India	–	–	KU495970	KU496114	KU496173
Calochromini	Macrolygistopterus	sp.	UPOL	MT0073	Ecuador	KU496031	KU496059	KU495987	KU496099	KU496215
Calochromini	Calochromus	sp.	UPOL	MT0074	California	KU496026	KU496066	KU495978	KU496121	KU496183
Calochromini	Calochromus	sp.	UPOL	MT0075	China	–	–	KU495989	KU496128	KU496199
Calochromini	Calochromus	sp.	UPOL	MT0076	China	KU496034	KU496062	KU495996	KU496126	–
Calochromini	Calochromus	sp.	UPOL	MT0077	China	KU496015	KU496047	KU495959	KU496081	KU496214
Calochromini	Calochromus	sp.	UPOL	MT0078	China	–	–	KU495965	–	KU496213
Calochromini	Calochromus	sp.	UPOL	MT0079	Laos	–	–	KU495997	KU496127	–
Calochromini	Calochromus	sp.	UPOL	MT0080	China	–	–	KU495990	–	KU496198
Calopterini	Calopteron	sp.	UPOL	000L25	Ecuador	DQ181053	DQ181127	DQ180979	DQ181201	DQ181355
Calopterini	Idiopteron	biplagiatum	UPOL	000M44	Ecuador	DQ181057	DQ181131	DQ180983	DQ181205	DQ181359
Calopterini	Calopteron	sp.	UPOL	A00621	Panama	KT752284	KT752449	KT752118	KT751792	KT751951
Calopterini	Calopteron	sp.	UPOL	A00628	Ecuador	–	–	KT752125	KT751798	KT751957
Calopterini	Calopteron	sp.	UPOL	A00629	Ecuador	–	–	KT752126	KT751799	KT751958
Calopterini	Calopteron	sp.	UPOL	A00630	Nicaragua	KT752289	KT752454	KT752127	KT751800	KT751959
Calopterini	Calopteron	sp.	UPOL	A00631	Ecuador	–	–	KT752128	KT751801	KT751960
Calopterini	Calopteron	sp.	UPOL	A00633	Ecuador	KT752291	KT752456	KT752129	KT751803	KT751962
Calopterini	Calopteron	sp.	UPOL	A00637	Argentina	–	–	KT752133	KT751807	–
Calopterini	Calopteron	sp.	UPOL	A00640	Nicaragua	KT752295	KT752460	KT752136	KT751809	KT751967
Calopterini	Calopteron	sp.	UPOL	A00645	Nicaragua	KT752296	KT752462	KT752139	KT751812	KT751970
Calopterini	Calopteron	sp.	UPOL	A00647	Bolivia	KT752298	KT752463	KT752141	KT751814	–
Calopterini	Calopteron	sp.	UPOL	A00650	Bolivia	KT752301	KT752466	KT752144	KT751817	KT751973
Calopterini	Calopteron	sp.	UPOL	A00651	Ecuador	–	–	KT752145	KT751818	KT751974
Calopterini	Metapteron	sp.	UPOL	IR2002	Peru	AF451946	DQ198757	–	DQ198588	–
Conderini	Conderis	signicollis	UPOL	000194	Malaysia	DQ181062	DQ181136	DQ180988	DQ181210	DQ181364
Conderini	Conderis	rufohumeralis	UPOL	000581	Japan	DQ181084	DQ181158	DQ181010	DQ181232	DQ181386
Conderini	Conderis	sp.	UPOL	A00601	Laos	KT752267	KT752431	KT752099	KT751774	KT751933
Conderini	Conderis	sp.	UPOL	A00602	China	KT752268	KT752432	KT752100	KT751775	KT751934
Conderini	Conderis	sp.	UPOL	A00603	China	KT752269	KT752433	KT752101	KT751776	KT751935
Conderini	Xylobanellus	sp.	UPOL	A00604	China	KT752270	KT752434	KT752102	KT751777	KT751936
Conderini	Conderis	sp.	UPOL	A00605	Vietnam	KT752271	KT752435	KT752103	KT751778	KT751937
Conderini	Conderis	sp.	UPOL	A00606	India	KT752272	KT752436	KT752104	KT751779	KT751938
Conderini	Conderis	sp.	UPOL	A00607	China	KT752273	KT752437	KT752105	KT751780	KT751939
Conderini	Xylobanellus	sp.	UPOL	A00608	Japan	KT752274	KT752438	KT752106	KT751781	KT751940
Conderini	Conderis	sp.	UPOL	A00609	Japan	KT752275	KT752439	KT752107	KT751782	KT751941
Conderini	Conderis	sp.	UPOL	A00610	Cambodia	KT752276	KT752440	KT752108	KT751783	KT751942
Conderini	Xylobanellus	sp.	UPOL	A00611	China	–	–	KT752109	–	–
Conderini	Conderis	sp.	UPOL	A00612	India	KT752277	KT752441	KT752110	KT751784	KT751943

Conderini	Conderis	sp.	UPOL	A00613	Borneo	KT752278	KT752442	KT752111	KT751785	KT751944
Conderini	Conderis	sp.	UPOL	A00614	Malaysia	KT752279	KT752443	KT752112	KT751786	KT751945
Conderini	Xylobanellus	sp.	UPOL	A00615	Laos	KT752280	KT752444	KT752113	KT751787	KT751946
Conderini	Xylobanellus	sp.	UPOL	A00616	Laos	KT752281	KT752445	KT752114	KT751788	KT751947
Dexorinae	Dexoris	chome	UPOL	A00654	Tanzania	KT752302	KT752467	KT752148	KT751819	–
Dexorinae	Dexoris	sp.	UPOL	VP0045	Cameroon	KT752309	KT752468	KT752147	KT751823	–
Dictyopterini	Dictyoptera	elegans	UPOL	000570	Japan	DQ181073	DQ181147	DQ180999	DQ181221	DQ181375
Dictyopterini	Dictyoptera	speciosa	UPOL	000571	Japan	DQ181074	DQ181148	DQ181000	DQ181222	DQ181376
Dictyopterini	Benibotarus	nigripennis	UPOL	000572	Japan	DQ181075	DQ181149	DQ181001	DQ181223	DQ181377
Dictyopterini	Benibotarus	spinicoxis	UPOL	000573	Japan	DQ181076	DQ181150	DQ181002	DQ181224	DQ181378
Dictyopterini	Pyropterus	nigroruber	UPOL	000574	Japan	DQ181077	DQ181151	DQ181003	DQ181225	DQ181379
Dictyopterini	Dictyoptera	sp.	UPOL	001275	USA	KF625686	KF626272	KF625976	KF625385	–
Dictyopterini	Dictyoptera	aurora	UPOL	001276	Czech Repu	KF625687	KF626273	KF625977	KF625386	–
Dictyopterini	Pyropterus	nigroruber	UPOL	001277	Czech Repu	KF625688	KF626274	KF625978	KF625387	–
Dictyopterini	Dictyopterini	indet.	UPOL	001278	Malaysia	KF625689	KF626275	KF625979	KF625388	–
Dictyopterini	Dictyopterini	indet.	UPOL	001282	Japan	KF625691	KF626277	KF625981	KF625390	–
Dictyopterini	Dictyopterini	indet.	UPOL	001283	Japan	KF625692	KF626278	KF625982	KF625391	–
Dictyopterini	Benibotarus	taygetanus	UPOL	001285	Czech Repu	KF625694	KF626280	KF625983	KF625392	–
Dictyopterini	Helcophorus	sp.	UPOL	001369	China	KF625699	KF626285	KF625988	KF625397	–
Dictyopterini	Dictyopterini	indet.	UPOL	A00519	China	KT752191	KT752352	KT752022	KT751703	–
Dictyopterini	Dictyopterini	indet.	UPOL	A00520	Malaysia	KT752192	KT752353	KT752023	KT751704	KT751862
Dictyopterini	Dictyopterini	indet.	UPOL	A00534	Malaysia	–	KT752365	KT752033	KT751716	KT751871
Dictyopterini	Dictyoptera	sp.	UPOL	A00540	Sumatra	KT752209	KT752371	KT752038	–	–
Dictyopterini	Benibotarus	taygetanus	UPOL	A00568	Hungary	KT752234	KT752399	KT752066	KT751744	KT751901
Dictyopterini	Benibotarus	spinicoxis	UPOL	A00569	China	KT752235	KT752400	KT752067	KT751745	KT751902
Dictyopterini	Helcophorus	sp.	UPOL	A00570	Thailand	KT752236	KT752401	KT752068	–	KT751903
Dictyopterini	Dictyopterini	indet.	UPOL	A00597	Malaysia	KT752263	KT752427	KT752095	KT751771	KT751929
Dictyopterini	Helcophorus	sp.	UPOL	A00618	China	KT752283	KT752447	KT752116	KT751790	KT751949
Dictyopterini	Dictyopterini	gen.sp.	UPOL	ZL2013	Japan	–	–	EF143223	EF143238	EF143252
Dihammatini	Dihammatus	sp.	UPOL	001001	Sumatra	DQ181103	DQ181177	DQ181029	DQ181251	DQ181405
Dihammatini	Dihammatus	sp.	UPOL	001009	Sumatra	DQ181106	DQ181180	DQ181032	DQ181254	DQ181408
Dihammatini	Dihammatus	sp.	UPOL	001017	Sumatra	DQ181108	DQ181182	DQ181034	DQ181256	DQ181410
Dihammatini	Dihammatus	sp.	UPOL	000L12	Borneo	DQ181043	DQ181117	DQ180969	DQ181191	DQ181345
Dihammatini	Dihammatus	sp.	UPOL	A00546	China	KT752215	KT752377	KT752044	KT751724	KT751881
Dihammatini	Dihammatus	sp.	UPOL	A00594	Malaysia	KT752260	KT752424	KT752092	–	KT751926
Dihammatini	Dihammatus	sp.	UPOL	A00596	Sumatra	KT752262	KT752426	KT752094	KT751770	KT751928
Dihammatini	Dihammatus	sp.	UPOL	A00598	Malaysia	KT752264	KT752428	KT752096	–	KT751930
Dihammatini	Dihammatus	sp.	UPOL	A00599	China	KT752265	KT752429	KT752097	KT751772	KT751931
Dihammatini	Dihammatus	sp.	UPOL	A00600	Laos	KT752266	KT752430	KT752098	KT751773	KT751932
Dihammatini	Dihammatus	sp.	UPOL	A00625	Malaysia	KT752287	KT752452	KT752122	KT751796	KT751954
Dilophotini	Dilophotes	sp.	UPOL	000244	Borneo	DQ181066	DQ181140	DQ180992	DQ181214	DQ181368
Dilophotini	Dilophotes	sp.	UPOL	A00003	Sumatra	–	–	KJ404888	KJ405081	KJ405272
Dilophotini	Dilophotes	sp.	UPOL	A00013	Sumatra	–	–	KJ404898	KJ405091	–
Dilophotini	Dilophotes	sp.	UPOL	A00060	Laos	KC538072	KC537863	KC538740	KC538359	KC538552
Dilophotini	Dilophotes	sp.	UPOL	TH0006	Sumatra	–	–	KJ404907	KJ405100	KJ405283
Dilophotini	Dilophotes	sp.	UPOL	TH0007	Sumatra	–	–	KJ404908	KJ405101	KJ405284
Dilophotini	Dilophotes	sp.	UPOL	TH0020	Sumatra	–	–	KJ404920	KJ405112	KJ405292
Dilophotini	Dilophotes	sp.	UPOL	TH0023	Sumatra	–	–	KJ404923	KJ405115	KJ405294
Dilophotini	Dilophotes	sp.	UPOL	TH0036	Borneo	–	–	KJ404928	KJ405128	–
Dilophotini	Dilophotes	sp.	UPOL	TH0039	Java	–	–	KJ404931	KJ405131	–
Dilophotini	Dilophotes	sp.	UPOL	TH0041	Sumatra	–	–	KJ404933	KJ405133	–
Dilophotini	Dilophotes	sp.	UPOL	TH0043	Sumatra	–	–	KJ404934	KJ405135	–
Dilophotini	Dilophotes	sp.	UPOL	TH0046	Laos	–	–	KJ404937	KJ405138	–
Dilophotini	Dilophotes	sp.	UPOL	TH0054	Java	–	–	KJ404945	KJ405146	–
Dilophotini	Dilophotes	sp.	UPOL	TH0059	Sumatra	–	–	KJ404950	KJ405151	–
Dilophotini	Dilophotes	sp.	UPOL	TH0068	Malaysia	–	–	KJ404957	KJ405156	KJ405307
Dilophotini	Dilophotes	sp.	UPOL	TH0069	Malaysia	–	–	KJ404958	KJ405157	–
Dilophotini	Dilophotes	sp.	UPOL	TH0076	Sumatra	–	–	KJ404965	KJ405164	KJ405314
Dilophotini	Dilophotes	sp.	UPOL	TH0078	Laos	–	–	KJ404967	KJ405166	KJ405316
Dilophotini	Dilophotes	sp.	UPOL	TH0079	Laos	–	–	KJ404968	KJ405167	KJ405317
Dilophotini	Dilophotes	sp.	UPOL	TH0080	Laos	–	–	KJ404969	KJ405168	KJ405318
Dilophotini	Dilophotes	sp.	UPOL	TH0081	Laos	–	–	KJ404970	KJ405169	KJ405319
Dilophotini	Dilophotes	sp.	UPOL	TH0093	Borneo	–	–	KJ404982	KJ405177	KJ405331
Dilophotini	Dilophotes	sp.	UPOL	TH0095	Laos	–	–	KJ404984	KJ405179	KJ405333
Dilophotini	Dilophotes	sp.	UPOL	TH0101	Borneo	–	–	KJ404990	KJ405185	KJ405339
Dilophotini	Dilophotes	sp.	UPOL	TH0125	Mindanao	–	–	KJ405014	KJ405208	KJ405356
Dilophotini	Dilophotes	sp.	UPOL	TH0127	Mindanao	–	–	KJ405016	KJ405210	KJ405357
Dilophotini	Dilophotes	sp.	UPOL	TH0131	China	–	–	KJ405020	KJ405214	–
Dilophotini	Dilophotes	sp.	UPOL	TH0138	Malaysia	–	–	KJ405027	KJ405221	KJ405361
Dilophotini	Dilophotes	sp.	UPOL	TH0152	Malaysia	–	–	KJ405040	KJ405234	KJ405365
Dilophotini	Dilophotes	sp.	UPOL	TH0160	Malaysia	–	–	KJ405048	KJ405242	KJ405368

Dilophotini	Dilophotes	sp.	UPOL	TH0190	China	–	–	KJ405078	KJ405270	KJ405398
Erotini	Platycis	minutus	UPOL	000348	Czech Repu	DQ181069	DQ181143	DQ181995	DQ181217	DQ181371
Erotini	Konoplatycis	otome	UPOL	000575	Japan	DQ181078	DQ181152	DQ181004	DQ181226	DQ181380
Erotini	Platycis	nasutus	UPOL	000576	Japan	DQ181079	DQ181153	DQ181005	DQ181227	DQ181381
Erotini	Lopheros	sp.	UPOL	000577	Japan	DQ181080	DQ181154	DQ181006	DQ181228	DQ181382
Erotini	Lopheros	sp.	UPOL	000578	Japan	DQ181081	DQ181155	DQ181007	DQ181229	DQ181383
Erotini	Eropterus	nothus	UPOL	000579	Japan	DQ181082	DQ181156	DQ181008	DQ181230	DQ181384
Erotini	Eropterus	sp.	UPOL	000580	USA	DQ181083	DQ181157	DQ181009	DQ181231	DQ181385
Erotini	Lopheros	sp.	UPOL	001284	Japan	KF625693	KF626279	–	–	–
Erotini	Platycis	cosnardi	UPOL	001286	Czech Repu	KF625695	KF626281	KF625984	KF625393	–
Erotini	Platycis	sp.	UPOL	001365	China	KF625696	KF626282	KF625985	–	–
Erotini	Platycis	sp.	UPOL	001366	USA	KF625697	KF626283	KF625986	KF625395	–
Erotini	Platycis	sp.	UPOL	A00547	China	KT752216	KT752378	KT752045	–	KT751882
Erotini	Eropterus	sp.	UPOL	A00548	China	KT752217	KT752379	KT752046	KT751725	KT751883
Erotini	Eropterus	sp.	UPOL	A00549	Laos	–	KT752380	KT752047	KT751726	KT751884
Erotini	Eropterus	sp.	UPOL	A00550	China	KT752218	KT752381	KT752048	KT751727	KT751885
Erotini	Eropterus	sp.	UPOL	A00551	Laos	KT752219	KT752382	KT752049	KT751728	KT751886
Erotini	Eropterus	sp.	UPOL	A00552	Laos	KT752220	KT752383	KT752050	KT751729	KT751887
Erotini	Eropterus	sp.	UPOL	A00553	Slovakia	–	KT752384	KT752051	KT751730	KT751888
Erotini	Eropterus	sp.	UPOL	A00554	Japan	–	KT752385	KT752052	KT751731	KT751889
Erotini	Lopheros	sp.	UPOL	A00556	Japan	KT752222	KT752387	KT752054	KT751733	KT751891
Erotini	Platycis	nasutus	UPOL	A00557	Korea	KT752223	KT752388	KT752055	KT751734	–
Erotini	Eropterus	sp.	UPOL	A00558	Japan	KT752224	KT752389	KT752056	KT751735	KT751892
Erotini	Platycis	comardi	UPOL	A00559	Czech Repu	KT752225	KT752390	KT752057	KT751736	–
Erotini	Platycis	sp.	UPOL	A00560	China	KT752226	KT752391	KT752058	–	KT751893
Erotini	Lopheros	sp.	UPOL	A00567	Laos	KT752233	KT752398	KT752065	KT751743	KT751900
Erotini	Platycis	sp.	UPOL	ZL2008	Japan	–	–	EF143218	EF143233	EF143247
Eurrhacini	Eurrhacus	sp.	UPOL	000M43	Ecuador	DQ181056	DQ181130	DQ180982	DQ181204	DQ181358
Eurrhacini	Eurrhacini	sp.	UPOL	A00622	Nicaragua	KT752285	KT752450	KT752119	KT751793	KT751952
Eurrhacini	Eurrhacini	sp.	UPOL	A00623	Ecuador	KT752286	KT752451	KT752120	KT751794	KT751953
Eurrhacini	Eurrhacini	sp.	UPOL	A00624	Ecuador	–	–	KT752121	KT751795	–
Eurrhacini	Eurrhacini	sp.	UPOL	A00627	Ecuador	–	–	KT752124	KT751797	KT751956
Eurrhacini	Eurrhacini	sp.	UPOL	A00636	Ecuador	KT752294	KT752459	KT752132	KT751806	KT751965
Eurrhacini	Eurrhacini	sp.	UPOL	A00638	Ecuador	–	–	KT752134	–	–
Eurrhacini	Eurrhacini	sp.	UPOL	A00641	Ecuador	–	–	KT752137	KT751810	KT751968
Eurrhacini	Eurrhacini	sp.	UPOL	A00648	Costa Rica	KT752299	KT752464	KT752142	KT751815	–
Eurrhacini	Eurrhacini	sp.	UPOL	A00649	Bolivia	KT752300	KT752465	KT752143	KT751816	KT751972
Leptolycini	Leptolycini	sp.	UPOL	000592	Costa Rica	DQ181092	DQ181166	DQ181018	DQ181240	DQ181394
Leptolycini	Leptolycus	sp.	UPOL	VP0022	Puerto Ricc	KT752303	KT752469	–	–	KT751976
Leptolycini	Leptolycus	sp.	UPOL	VP0023	Puerto Ricc	KT752304	KT752470	–	–	–
Leptolycini	Leptolycus	sp.	UPOL	VP0024	Puerto Ricc	KT752305	KT752471	KT752149	–	KT751977
Leptolycini	Leptolycus	sp.	UPOL	VP0025	Puerto Ricc	KT752306	KT752472	–	KT751820	–
Leptolycini	Leptolycus	sp.	UPOL	VP0026	Puerto Ricc	KT752307	KT752473	KT752150	–	KT751978
Leptolycini	Leptolycus	sp.	UPOL	VP0027	Puerto Ricc	KT752308	KT752474	KT752151	KT751821	–
Libnetinae	Libnetis	sp.	UPOL	001002	Sumatra	DQ181104	DQ181178	DQ181030	DQ181252	DQ181406
Libnetinae	Libnetis	sp.	UPOL	001008	Malaysia	DQ181105	DQ181179	DQ181031	DQ181253	DQ181407
Libnetinae	Libnetis	granicollis	UPOL	001012	Japan	DQ181107	DQ181181	DQ181033	DQ181255	DQ181409
Libnetinae	Libnetis	sp.	UPOL	000L02	Borneo	DQ181038	DQ181112	DQ180964	DQ181186	DQ181340
Libnetinae	Libnetis	sp.	UPOL	A00577	Malaysia	KT752243	–	KT752075	KT751752	KT751909
Libnetinae	Libnetis	sp.	UPOL	A00578	China	KT752244	KT752408	KT752076	KT751753	KT751910
Libnetinae	Libnetis	sp.	UPOL	A00581	China	KT752247	KT752411	KT752079	KT751756	KT751913
Libnetinae	Libnetis	sp.	UPOL	A00582	Laos	KT752248	KT752412	KT752080	KT751757	KT751914
Libnetinae	Libnetis	sp.	UPOL	A00583	China	KT752249	KT752413	KT752081	KT751758	KT751915
Libnetinae	Libnetis	sp.	UPOL	A00584	Laos	KT752250	KT752414	KT752082	KT751759	KT751916
Libnetinae	Libnetis	sp.	UPOL	A00585	Malaysia	KT752251	KT752415	KT752083	KT751760	KT751917
Libnetinae	Libnetis	sp.	UPOL	A00586	Malaysia	KT752252	KT752416	KT752084	KT751761	KT751918
Libnetinae	Libnetis	sp.	UPOL	A00587	Malaysia	KT752253	KT752417	KT752085	KT751762	KT751919
Libnetinae	Libnetis	sp.	UPOL	A00588	Malaysia	KT752254	KT752418	KT752086	KT751763	KT751920
Libnetinae	Libnetis	sp.	UPOL	A00589	Sumatra	KT752255	KT752419	KT752087	KT751764	KT751921
Libnetinae	Libnetis	sp.	UPOL	A00590	Sumatra	KT752256	KT752420	KT752088	KT751765	KT751922
Libnetinae	Libnetis	sp.	UPOL	A00591	Laos	KT752257	KT752421	KT752089	KT751766	KT751923
Lycini	Lycus	sp.	UPOL	000L03	RSA	DQ181039	DQ181113	DQ180965	DQ181187	DQ181341
Lycini	Lycostomus	sp.	UPOL	000L27	China	DQ181055	DQ181129	DQ180981	DQ181203	DQ181357
Lycini	Lycostomus	sp.	UPOL	A00328	China	–	–	KF806834	KF806756	KF806798
Lycini	Lycus	sp.	UPOL	A00469	Zambia	KT752152	KT752310	KT751979	KT751662	KT751824
Lycini	Lycus	sp.	UPOL	LF0021	Ethiopia	–	–	KF806835	KF806757	KF806799
Lycini	Lycus	sp.	UPOL	LF0031	Cameroon	–	–	KF806836	KF806758	KF806800
Lycini	Lycus	sp.	UPOL	LF0035	Cameroon	–	–	KF806837	KF806759	KF806801
Lycini	Lycus	sp.	UPOL	LF0043	Ethiopia	–	–	KF806838	KF806760	KF806802
Lycini	Lycus	sp.	UPOL	LF0077	RSA	–	–	KF806839	KF806761	KF806803
Lycini	Lycus	sp.	UPOL	LF0093	Tanzania	–	–	–	KF806762	KF806804

Lycini	Lycus	sp.	UPOL	LF0110	Zambia	–	–	KF806840	KF806763	KF806805
Lycini	Lycus	sp.	UPOL	LF0116	RSA	–	–	KF806841	KF806764	KF806806
Lycini	Lycus	sp.	UPOL	LF0143	Zambia	–	–	KF806842	KF806765	KF806807
Lycini	Lycus	sp.	UPOL	LF0167	Ethiopia	–	–	KF806843	KF806766	KF806808
Lycini	Lycus	sp.	UPOL	LF0168	Ethiopia	–	–	KF806844	KF806767	KF806809
Lycini	Lycus	sp.	UPOL	LF0230	Zambia	–	–	–	KF806768	KF806810
Lycini	Lycus	sp.	UPOL	LF0262	Cameroon	–	–	KF806845	KF806769	KF806811
Lycini	Lycus	sp.	UPOL	LF0264	Cameroon	–	–	KF806846	KF806770	KF806812
Lycini	Lycus	sp.	UPOL	LF0266	Cameroon	–	–	KF806847	KF806771	KF806813
Lycini	Lycus	sp.	UPOL	LF0270	Cameroon	–	–	KF806848	KF806772	KF806814
Lycini	Lycostomus	sp.	UPOL	LF0303	Laos	–	–	KF806849	KF806773	–
Lycini	Lycostomus	sp.	UPOL	LF0304	Laos	–	–	KF806850	KF806774	–
Lycini	Lycostomus	sp.	UPOL	LF0308	Laos	–	–	KF806851	KF806775	–
Lycini	Lycostomus	sp.	UPOL	LF0319	India	–	–	–	KF806776	–
Lycini	Lycostomus	sp.	UPOL	LF0320	India	–	–	KF806852	KF806777	–
Lycini	Lycostomus	sp.	UPOL	LF0323	India	–	–	KF806853	KF806778	–
Lycini	Lycostomus	sp.	UPOL	LF0331	Indonesia	–	–	KF806854	KF806779	KF806815
Lycini	Lycostomus	sp.	UPOL	LF0340	Taiwan	–	–	KF806855	KF806780	KF806816
Lycini	Lycostomus	sp.	UPOL	LF0350	Japan	–	–	–	KF806781	KF806817
Lycini	Lycostomus	sp.	UPOL	LF0354	Japan	–	–	KF806856	KF806782	KF806818
Lycini	Lycostomus	sp.	UPOL	LF0359	Taiwan	–	–	–	KF806783	KF806819
Lycini	Lycostomus	sp.	UPOL	LF0365	Malaysia	–	–	KF806857	KF806784	KF806820
Lycini	Lycostomus	sp.	UPOL	LF0370	India	–	–	KF806858	KF806785	KF806821
Lycini	Lycostomus	sp.	UPOL	LF0381	India	–	–	–	KF806786	KF806822
Lycini	Lycostomus	sp.	UPOL	LF0388	Namibia	–	–	KF806859	KF806787	KF806823
Lycini	Lycus	sp.	UPOL	LF0394	Namibia	–	–	KF806860	KF806788	KF806824
Lycini	Lycini	indet.	UPOL	LF0428	Mexico	–	–	–	KF806789	KF806825
Lycini	Lycini	indet.	UPOL	LF0429	Nicaragua	–	–	KF806861	KF806790	KF806826
Lycini	Lycini	indet.	UPOL	LF0430	Ecuador	–	–	KF806862	KF806791	KF806827
Lycini	Lycus	sp.	UPOL	LF0444	Ethiopia	–	–	KF806863	KF806792	KF806828
Lycini	Neoycus	arizonensis	UPOL	LF0451	USA	–	–	KF806864	KF806793	KF806829
Lycini	Neoycus	arizonensis	UPOL	LF0452	USA	–	–	KF806865	KF806794	KF806830
Lycini	Neoycus	arizonensis	UPOL	LF0454	USA	–	–	KF806866	KF806795	KF806831
Lycini	Lycostomus	loripes	UPOL	LF0464	USA	–	–	KF806867	KF806796	KF806832
Lycini	Lycostomus	loripes	UPOL	LF0466	USA	–	–	KF806868	KF806797	KF806833
Lycoprogenthini	Lycoprogentes	sp.	UPOL	000358	Java	DQ181070	DQ181144	DQ180996	DQ181218	DQ181372
Lycoprogenthini	Lycoprogentes	sp.	UPOL	000801	Sumatra	DQ181095	DQ181169	DQ181021	DQ181243	DQ181397
Lycoprogenthini	Lycoprogentes	sp.	UPOL	000802	Java	DQ181096	DQ181170	DQ181022	DQ181244	DQ181398
Lycoprogenthini	Lycoprogentes	sp.	UPOL	000805	Sumatra	DQ181097	DQ181171	DQ181023	DQ181245	DQ181399
Lycoprogenthini	Lycoprogentes	sp.	UPOL	A00523	Java	KT752193	KT752354	KT752024	KT751705	–
Lycoprogenthini	Lycoprogentes	sp.	UPOL	A00524	Malaysia	KT752194	KT752355	–	KT751706	KT751863
Lycoprogenthini	Lycoprogentes	sp.	UPOL	A00530	Malaysia	KT752200	KT752361	KT752029	KT751712	KT751867
Lycoprogenthini	Lycoprogentes	sp.	UPOL	A00532	Java	KT752202	KT752363	KT752031	KT751714	KT751869
Lycoprogenthini	Lycoprogentes	sp.	UPOL	A00533	Sumatra	KT752203	KT752364	KT752032	KT751715	KT751870
Lycoprogenthini	Lycoprogentes	sp.	UPOL	A00541	Sumatra	KT752210	KT752372	KT752039	KT751720	KT751876
Lycoprogenthini	Lycoprogentes	sp.	UPOL	A00542	Sumatra	KT752211	KT752373	KT752040	KT751721	KT751877
Lycoprogenthini	Lycoprogentes	sp.	UPOL	A00545	Sumatra	KT752214	KT752376	KT752043	–	KT751880
Lycoprogenthini	Lycoprogentes	sp.	UPOL	A00572	India	KT752238	KT752403	KT752070	KT751747	KT751904
Lycoprogenthini	Lycoprogentes	sp.	UPOL	A00626	India	KT752288	KT752453	KT752123	–	KT751955
Lyponiini	Lyponia	delicatula	UPOL	000815	Japan	DQ181099	DQ181173	DQ181025	DQ181247	DQ181401
Lyponiini	Lyponia	sp.	UPOL	000816	China	DQ181100	DQ181174	DQ181026	DQ181248	DQ181402
Lyponiini	Lyponia	quadricollis	UPOL	000817	Korea	DQ181101	DQ181175	DQ181027	DQ181249	DQ181403
Lyponiini	Lyponia	nigrohumeralis	UPOL	000L17	China	DQ181048	DQ181122	DQ180974	DQ181196	DQ181350
Lyponiini	Ponyalis	laticornis	UPOL	A00460	China	KU184270	KU184278	ab123456	KJ650401	ab123456
Lyponiini	Lyponia	sp.	UPOL	A00467	China	KU184276	KU184284	ab123456	KJ650453	ab123456
Lyponiini	Lyponia	tianquanensis	UPOL	A00468	China	KU184277	KU184285	ab123456	KJ650450	ab123456
Lyponiini	Lyponia	sp.	UPOL	A00571	Laos	KT752237	KT752402	KT752069	KT751746	–
Lyponiini	Ponyalis	alternata	UPOL	YL0217	China	–	–	ab123456	KJ650408	ab123456
Lyponiini	Ponyalis	ishigakiana	UPOL	YL0259	Japan	–	–	ab123456	KJ650438	ab123456
Lyponiini	Ponyalis	oshimana	UPOL	YL0263	Japan	–	–	ab123456	KJ650415	ab123456
Lyponiini	Ponyalis	fukiensis	UPOL	YL0282	China	–	–	ab123456	KJ650420	ab123456
Lyponiini	Lyponia	debilis	UPOL	YL0296	China	–	–	ab123456	KJ650468	ab123456
Lyponiini	Lyponia	muyuensis	UPOL	YL0314	China	–	–	ab123456	KJ650493	ab123456
Lyponiini	Lyponia	kuatunensis	UPOL	YL0319	China	–	–	ab123456	KJ650482	ab123456
Lyponiini	Ponyalis	gracilis	UPOL	YL0409	China	–	–	ab123456	KJ650437	ab123456
Lyponiini	Lyponia	sp.	UPOL	ZL2014	Japan	–	–	FJ390408	FJ390410	FJ390412
Lyponiini	Lyponia	sp.	UPOL	ZL2016	Japan	–	–	EF143225	EF143240	EF143253
Lyropaeini	Lyropaeus	waterhousei	UPOL	000584	Sumatra	DQ181087	DQ181161	DQ181013	DQ181235	DQ181389
Lyropaeini	Lyropaeus	optabilis	UPOL	000585	Malaysia	DQ181088	DQ181162	DQ181014	DQ181236	DQ181390
Lyropaeini	Lyropaeus	rubrostriatus	UPOL	000L11	Borneo	DQ181042	DQ181116	DQ180968	DQ181190	DQ181344
Lyropaeini	Lyropaeus	sp.	UPOL	A00574	Malaysia	KT752240	KT752405	KT752072	KT751749	KT751906

Lyropaeini	Lyropaeus	ritsemae	UPOL	VP0001	Sumatra	KC736888	KC736898	KC736880	KC736907	KC736916
Lyropaeini	Lyropaeus	waterhouesi	UPOL	VP0002	Sumatra	KC736889	–	KC736881	KC736908	KC736917
Lyropaeini	Lyropaeus	dominator	UPOL	VP0003	Malaysia	KC736890	KC736899	KC736882	KC736909	KC736918
Lyropaeini	Lyropaeus	optabilis	UPOL	VP0004	Malaysia	KC736891	KC736900	KC736883	KC736910	KC736919
Lyropaeini	Lyropaeus	ritsemae	UPOL	VP0006	Sumatra	KC736892	KC736901	KC736884	KC736911	KC736920
Lyropaeini	Lyropaeus	sp.	UPOL	VP0016	India	KC736893	KC736902	KC736885	KC736912	KC736921
Lyropaeini	Lyropaeus	sp.	UPOL	VP0017	India	KC736894	KC736903	KC736886	–	–
Lyropaeini	Lyropaeus	philippinensis	UPOL	VP0018	Philippines	KC736895	KC736904	–	KC736913	KC736922
Lyropaeini	Lyropaeus	philippinensis	UPOL	VP0019	Philippines	KC736896	KC736905	–	KC736914	KC736923
Lyropaeini	Lyropaeus	sp.	UPOL	VP2312	India	KC736897	KC736906	KC736887	KC736915	KC736924
Macrolycini	Macrolycus	sp.	UPOL	000828	Thailand	DQ181102	DQ181176	DQ181028	DQ181250	DQ181404
Macrolycini	Macrolycus	sp.	UPOL	000L18	China	DQ181049	DQ181123	DQ180975	DQ181197	DQ181351
Macrolycini	Macrolycus	bowringi	UPOL	A00461	China	KU184271	KU184279	ab123456	ab123456	ab123456
Macrolycini	Macrolycus	ligulatus	UPOL	A00462	China	–	–	ab123456	ab123456	ab123456
Macrolycini	Macrolycus	sichuanensis	UPOL	A00463	China	KU184272	KU184280	ab123456	ab123456	ab123456
Macrolycini	Macrolycus	galinae	UPOL	A00464	China	KU184273	KU184281	ab123456	ab123456	ab123456
Macrolycini	Macrolycus	atronotatus	UPOL	A00465	China	KU184274	KU184282	ab123456	ab123456	ab123456
Macrolycini	Macrolycus	oreophilus	UPOL	A00466	China	KU184275	KU184283	ab123456	ab123456	ab123456
Macrolycini	Macrolycus	flabellatus	UPOL	YL0032	Japan	–	–	ab123456	ab123456	ab123456
Macrolycini	Macrolycus	dotatus	UPOL	YL0185	China	–	–	ab123456	ab123456	ab123456
Macrolycini	Macrolycus	ochraceus	UPOL	YL0192	China	–	–	ab123456	KJ650495	ab123456
Macrolycini	Macrolycus	mucronatus	UPOL	YL0206	China	–	–	ab123456	ab123456	ab123456
Macrolycini	Macrolycus	bicolor	UPOL	YL0403	China	–	–	ab123456	ab123456	ab123456
Macrolycini	Macrolycus	sp.	UPOL	ZL2005	Japan	–	–	EF143217	EF143232	EF143246
Metriorrhynchini	Metriorrhynchus	lineatus	UPOL	000009	Sumatra	KC538123	KC537913	KC538628	DQ904297	DQ904259
Metriorrhynchini	Metriorrhynchus	sp.	UPOL	000010	Sulawesi	KC538124	KC537914	–	DQ144659	DQ144685
Metriorrhynchini	Metriorrhynchus	sp.	UPOL	000011	Sulawesi	KC538125	KC537915	KC538629	DQ144660	DQ144686
Metriorrhynchini	Metriorrhynchus	lobatus	UPOL	000017	Sulawesi	KC538126	KC537916	KC538630	DQ144662	DQ144688
Metriorrhynchini	Metanoeus	sp.	UPOL	000026	Borneo	KC538127	KC537917	KC538631	KC538244	KC538436
Metriorrhynchini	Cautires	sp.	UPOL	000030	Borneo	KC538128	KC537918	KC538632	KC538245	KC538437
Metriorrhynchini	Cautires	sp.	UPOL	000037	Borneo	KC538129	KC537919	KC538633	KC538246	KC538438
Metriorrhynchini	Cautires	sp.	UPOL	000040	Borneo	KC538130	KC537920	KC538634	KC538247	KC538439
Metriorrhynchini	Cautires	sp.	UPOL	000043	Borneo	KC538131	KC537921	KC538635	KC538248	KC538440
Metriorrhynchini	Cautires	sp.	UPOL	000044	Borneo	KC538132	KC537922	KC538636	KC538249	KC538441
Metriorrhynchini	Cautires	sp.	UPOL	000047	Sumatra	KC538133	KC537923	KC538637	KC538250	KC538442
Metriorrhynchini	Cautires	sp.	UPOL	000048	Sumatra	KC538134	KC537924	KC538638	KC538251	KC538443
Metriorrhynchini	Cautires	sp.	UPOL	000050	Sumatra	KC538135	KC537925	KC538639	KC538252	KC538444
Metriorrhynchini	Cautires	sp.	UPOL	000052	Sumatra	KC538136	KC537926	KC538640	KC538253	KC538445
Metriorrhynchini	Cautires	sp.	UPOL	000056	Sumatra	KC538137	KC537927	KC538641	KC538254	KC538446
Metriorrhynchini	Cautires	sp.	UPOL	000060	Sumatra	KC538138	KC537928	KC538642	KC538255	KC538447
Metriorrhynchini	Cautires	sp.	UPOL	000064	Laos	KC538139	KC537929	KC538643	KC538256	KC538448
Metriorrhynchini	Cautires	sp.	UPOL	000066	Laos	KC538140	KC537930	KC538644	KC538257	KC538449
Metriorrhynchini	Cautires	sp.	UPOL	000068	Borneo	KC538141	KC537931	KC538645	KC538258	KC538450
Metriorrhynchini	Cautires	sp.	UPOL	000069	Borneo	KC538142	KC537932	KC538646	KC538259	KC538451
Metriorrhynchini	Cautires	sp.	UPOL	000070	Malaysia	KC538143	KC537933	KC538647	KC538260	KC538452
Metriorrhynchini	Xylobanus	sp.	UPOL	000071	Borneo	KC538144	KC537934	KC538648	KC538261	KC538453
Metriorrhynchini	Cautires	sp.	UPOL	000074	Borneo	KC538145	KC537935	KC538649	KC538262	KC538454
Metriorrhynchini	Cautires	sp.	UPOL	000075	Laos	KC538146	KC537936	–	KC538263	KC538455
Metriorrhynchini	Cautires	sp.	UPOL	000079	Borneo	KC538147	KC537937	KC538650	KC538264	KC538456
Metriorrhynchini	Cautires	sp.	UPOL	000080	Borneo	KC538148	KC537938	KC538651	KC538265	KC538457
Metriorrhynchini	Cautires	sp.	UPOL	000081	Borneo	KC538149	KC537939	KC538652	KC538266	KC538458
Metriorrhynchini	Cautires	sp.	UPOL	000084	Borneo	KC538150	KC537940	KC538653	KC538267	KC538459
Metriorrhynchini	Cautires	sp.	UPOL	000088	Malaysia	KC538151	KC537941	KC538654	KC538268	KC538460
Metriorrhynchini	Cautires	sp.	UPOL	000090	Borneo	KC538152	KC537942	KC538655	KC538269	KC538461
Metriorrhynchini	Cautires	sp.	UPOL	000104	Borneo	KC538153	KC537943	KC538656	KC538270	KC538462
Metriorrhynchini	Metanoeus	sp.	UPOL	000105	Borneo	KC538154	KC537944	KC538657	KC538271	KC538463
Metriorrhynchini	Cautires	sp.	UPOL	000109	Borneo	–	KC537945	KC538658	KC538272	KC538464
Metriorrhynchini	Xylobanus	sp.	UPOL	000120	Laos	KC538155	KC537946	KC538659	KC538273	KC538465
Metriorrhynchini	Metanoeus	sp.	UPOL	000121	Sumatra	KC538156	KC537947	KC538660	KC538274	KC538466
Metriorrhynchini	Cautires	sp.	UPOL	000122	Borneo	KC538157	KC537948	KC538661	KC538275	KC538467
Metriorrhynchini	Cautires	sp.	UPOL	000123	Java	KC538158	KC537949	–	KC538276	–
Metriorrhynchini	Metanoeus	sp.	UPOL	000125	Sumatra	KC538159	KC537950	KC538662	KC538277	KC538468
Metriorrhynchini	Xylobanus	sp.	UPOL	000132	Sumatra	KC538160	KC537951	KC538663	HQ456987	HQ457009
Metriorrhynchini	Cautires	sp.	UPOL	000147	India	KC538161	KC537952	KC538664	KC538278	KC538470
Metriorrhynchini	Xylobanus	sp.	UPOL	000152	Laos	KC538162	KC537953	KC538665	KC538279	KC538471
Metriorrhynchini	Xylobanus	sp.	UPOL	000153	Laos	KC538163	KC537954	KC538666	KC538280	KC538472
Metriorrhynchini	Xylobanus	sp.	UPOL	000154	Laos	KC538164	KC537955	–	KC538281	–
Metriorrhynchini	Cautires	sp.	UPOL	000164	Laos	KC538165	KC537956	KC538667	KC538282	KC538473
Metriorrhynchini	Cautires	sp.	UPOL	000174	Malaysia	KC538166	KC537957	–	KC538283	KC538474
Metriorrhynchini	Cautires	sp.	UPOL	000177	Malaysia	KC538167	–	KC538668	KC538284	KC538475
Metriorrhynchini	Cautires	sp.	UPOL	000178	Malaysia	KC538168	KC537958	KC538669	KC538285	KC538476

Metriorrhynchini	Xylobanus	sp.	UPOL	000184	Borneo	KC538169	KC537959	KC538670	KC538286	KC538477
Metriorrhynchini	Cautires	sp.	UPOL	000188	Laos	KC538170	KC537960	KC538671	KC538287	KC538478
Metriorrhynchini	Cautires	sp.	UPOL	000189	Laos	KC538171	KC537961	KC538672	KC538288	KC538479
Metriorrhynchini	Cautires	sp.	UPOL	000195	RSA	KC538172	KC537962	KC538673	KC538289	KC538480
Metriorrhynchini	Microtrichalus	sp.	UPOL	000199	Sulawesi	KC538173	KC537963	KC538674	KC538290	KC538481
Metriorrhynchini	Cautires	sp.	UPOL	000205	Sumatra	KC538174	KC537964	KC538675	KC538291	KC538482
Metriorrhynchini	Cautires	sp.	UPOL	000206	Sumatra	KC538175	KC537965	KC538676	KC538292	KC538483
Metriorrhynchini	Leptotrichalus	sp.	UPOL	000208	Borneo	DQ181064	DQ181138	DQ180990	DQ181212	DQ181366
Metriorrhynchini	Cautires	sp.	UPOL	000217	Japan	KC538176	KC537966	KC538678	KC538293	KC538484
Metriorrhynchini	Cautires	sp.	UPOL	000219	Japan	KC538177	KC537967	KC538679	KC538294	KC538485
Metriorrhynchini	Cautires	sp.	UPOL	000220	Japan	KC538178	KC537968	KC538680	KC538295	KC538486
Metriorrhynchini	Xylometanoeus	basivittatus	UPOL	000221	Japan	KC538179	–	KC538681	KC538296	KC538487
Metriorrhynchini	Xylometanoeus	basivittatus	UPOL	000222	Japan	–	–	–	–	KF652128
Metriorrhynchini	Xylometanoeus	basivittatus	UPOL	000223	Japan	–	–	–	–	KF652129
Metriorrhynchini	Xylobanus	sp.	UPOL	000224	Japan	KC538180	KC537969	KC538682	KC538297	KC538488
Metriorrhynchini	Cautires	sp.	UPOL	000246	Sumatra	KC538181	KC537970	KC538683	KC538298	KC538489
Metriorrhynchini	Metanoeus	sp.	UPOL	000248	Sumatra	KC538182	KC537971	KC538684	KC538299	KC538490
Metriorrhynchini	Xylobanus	sp.	UPOL	000262	Borneo	–	KC537972	KC538685	KC538300	KC538491
Metriorrhynchini	Xylobanus	sp.	UPOL	000274	Borneo	KC538183	KC537973	KC538686	KC538301	KC538492
Metriorrhynchini	Cautires	sp.	UPOL	000290	Laos	KC538184	KC537974	KC538687	KC538302	KC538493
Metriorrhynchini	Cautires	sp.	UPOL	000294	Sumatra	KC538185	KC537975	KC538688	KC538303	KC538494
Metriorrhynchini	Cautires	sp.	UPOL	000295	Sumatra	KC538186	KC537976	KC538689	KC538304	KC538495
Metriorrhynchini	Cautires	sp.	UPOL	000297	Sumatra	KC538187	KC537977	KC538690	KC538305	KC538496
Metriorrhynchini	Cautires	sp.	UPOL	000314	Sumatra	KC538188	KC537978	KC538691	KC538306	KC538497
Metriorrhynchini	Xylobanus	sp.	UPOL	000315	Sumatra	KC538189	KC537979	KC538692	KC538307	KC538498
Metriorrhynchini	Cautires	sp.	UPOL	000335	Borneo	–	–	KC538693	KC538308	KC538499
Metriorrhynchini	Cautires	sp.	UPOL	000339	Borneo	KC538190	KC537980	KC538694	KC538309	KC538500
Metriorrhynchini	Cautires	sp.	UPOL	000342	Borneo	KC538191	KC537981	KC538695	KC538310	KC538501
Metriorrhynchini	Cautires	sp.	UPOL	000346	Borneo	KC538192	KC537982	KC538696	KC538311	KC538502
Metriorrhynchini	Cautires	sp.	UPOL	000355	Java	–	KC537983	KC538697	KC538312	KC538503
Metriorrhynchini	Metriorrhynchus	palawensis	UPOL	000366	Palawan	–	–	KC538698	DQ144665	DQ144691
Metriorrhynchini	Porrostoma	rhipidum	UPOL	000372	Australia	KC538193	KC537984	KC538699	DQ144678	DQ144702
Metriorrhynchini	Microtrichalus	sp.	UPOL	000373	Australia	KC538194	KC537985	KC538700	KC538313	KC538504
Metriorrhynchini	Metriorrhynchus	sp.	UPOL	000374	Australia	KC538195	KC537986	KC538701	KC538314	KC538505
Metriorrhynchini	Microtrichalus	sp.	UPOL	000375	Australia	KC538196	KC537987	KC538702	KC538315	KC538506
Metriorrhynchini	Microtrichalus	sp.	UPOL	000376	Australia	KC538197	KC537988	KC538703	KC538316	KC538507
Metriorrhynchini	Porrostoma	haemorrhoidalis	UPOL	000378	Australia	KC538198	KC537989	KC538704	DQ144679	DQ144703
Metriorrhynchini	Xylobanus	sp.	UPOL	000379	Palawan	KC538199	–	KC538705	KC538317	KC538508
Metriorrhynchini	Cautiromimus	sp.	UPOL	000388	Palawan	–	–	–	KC538318	KC538509
Metriorrhynchini	Cautires	sp.	UPOL	000395	Palawan	–	–	KC538706	KC538319	KC538510
Metriorrhynchini	Leptotrichalus	sp.	UPOL	000396	Palawan	–	–	KC538707	KC538320	KC538511
Metriorrhynchini	Xylobanus	sp.	UPOL	000402	Palawan	KC538200	–	KC538708	KC538322	KC538513
Metriorrhynchini	Cautires	sp.	UPOL	000403	Palawan	–	KC537990	KC538709	KC538323	KC538514
Metriorrhynchini	Cautires	sp.	UPOL	000411	Palawan	KC538201	–	KC538710	KC538324	KC538515
Metriorrhynchini	Microtrichalus	sp.	UPOL	000412	Palawan	KC538202	KC537991	KC538711	KC538325	KC538516
Metriorrhynchini	Leptotrichalus	sp.	UPOL	000419	Palawan	KC538203	KC537992	KC538712	KC538326	KC538517
Metriorrhynchini	Cautires	sp.	UPOL	000425	Palawan	–	–	KC538713	KC538327	KC538518
Metriorrhynchini	Metanoeus	sp.	UPOL	000434	Palawan	–	–	KC538714	KC538328	KC538519
Metriorrhynchini	Metriorrhynchus	lineatus	UPOL	000L05	Borneo	DQ181040	DQ181114	DQ180966	DQ181188	DQ181342
Metriorrhynchini	Cautires	cf. Montanus	UPOL	000L06	Borneo	DQ181041	DQ181115	DQ180967	DQ181189	DQ181343
Metriorrhynchini	Cautires	sp.	UPOL	000L14	RSA	DQ181045	DQ181119	DQ180971	DQ181193	DQ181347
Metriorrhynchini	Microtrichalus	sp.	UPOL	000L23	Borneo	DQ181052	DQ181126	DQ180978	DQ181200	DQ181354
Metriorrhynchini	Cautires	sp.	UPOL	A00017	Taiwan	–	KC537824	–	–	–
Metriorrhynchini	Xylobanus	sp.	UPOL	A00018	Taiwan	KC538033	KC537825	HQ456946	HQ456964	HQ456988
Metriorrhynchini	Cautires	sp.	UPOL	A00019	Taiwan	KC538034	KC537826	KC538715	KC538329	KC538520
Metriorrhynchini	Cautires	sp.	UPOL	A00020	Taiwan	KC538035	KC537827	–	–	–
Metriorrhynchini	Cautires	sp.	UPOL	A00021	Taiwan	KC538036	KC537828	HQ456947	HQ456965	–
Metriorrhynchini	Cautires	sp.	UPOL	A00022	Madagasca	KC538037	KC537829	KC538716	HQ456966	HQ456989
Metriorrhynchini	Cautires	sp.	UPOL	A00023	Madagasca	KC538038	KC537830	KC538717	KC538330	KC538521
Metriorrhynchini	Cautires	sp.	UPOL	A00024	Madagasca	KC538039	KC537831	KC538718	KC538331	KC538522
Metriorrhynchini	Cautires	sp.	UPOL	A00025	Madagasca	KC538040	KC537832	KC538719	KC538332	KC538523
Metriorrhynchini	Cautires	sp.	UPOL	A00026	Madagasca	KC538041	KC537833	–	KC538333	KC538524
Metriorrhynchini	Cautires	sp.	UPOL	A00027	Madagasca	KC538042	KC537834	–	KC538334	KC538525
Metriorrhynchini	Cautires	sp.	UPOL	A00028	Madagasca	KC538043	KC537835	–	KC538335	–
Metriorrhynchini	Cautires	sp.	UPOL	A00029	Madagasca	KC538044	KC537836	–	KC538336	KC538526
Metriorrhynchini	Cautires	sp.	UPOL	A00030	Madagasca	KC538045	KC537837	KC538720	KC538337	KC538527
Metriorrhynchini	Synchonnus	sp.	UPOL	A00031	Australia	KC538046	KC537838	KC538721	KC538338	KC538528
Metriorrhynchini	Trichalus	sp.	UPOL	A00032	Australia	KC538047	KC537839	KC538722	KC538339	KC538529
Metriorrhynchini	Ditua	sp.	UPOL	A00033	Australia	KC538048	KC537840	KC538723	–	KC538530
Metriorrhynchini	Metriorrhynchus	sp.	UPOL	A00034	Australia	KC538049	KC537841	KC538724	KC538340	KC538531
Metriorrhynchini	Porrostoma	sp.	UPOL	A00035	Australia	KC538050	KC537842	KC538725	KC538341	KC538532

Metriorrhynchini	Porrostoma	sp.	UPOL	A00036	Australia	KC538051	KC537843	KC538726	KC538342	KC538533
Metriorrhynchini	Porrostoma	sp.	UPOL	A00037	Australia	KC538052	KC537844	KC538727	KC538343	KC538534
Metriorrhynchini	Metriorrhynchus	sp.	UPOL	A00038	Australia	KC538053	KC537845	KC538728	KC538344	KC538535
Metriorrhynchini	Metriorrhynchus	sp.	UPOL	A00039	Australia	KC538054	KC537846	KC538729	KC538345	KC538536
Metriorrhynchini	Porrostoma	sp.	UPOL	A00040	Australia	KC538055	KC537847	KC538730	KC538346	KC538537
Metriorrhynchini	Porrostoma	sp.	UPOL	A00041	Australia	KC538056	KC537848	KC538731	KC538347	KC538538
Metriorrhynchini	Porrostoma	sp.	UPOL	A00042	Australia	KC538057	KC537849	–	KC538348	KC538539
Metriorrhynchini	Metriorrhynchus	sp.	UPOL	A00043	Australia	KC538058	KC537850	KC538732	KC538349	KC538540
Metriorrhynchini	Porrostoma	sp.	UPOL	A00044	Australia	KC538059	KC537851	KC538733	KC538350	KC538541
Metriorrhynchini	Porrostoma	sp.	UPOL	A00045	Australia	KC538060	KC537852	KC538734	KC538351	KC538542
Metriorrhynchini	Metriorrhynchus	sp.	UPOL	A00046	Australia	KC538061	KC537853	KC538735	KC538352	KC538543
Metriorrhynchini	Cautires	sp.	UPOL	A00048	Malaysia	KC538063	KC537855	HQ456948	HQ456967	HQ456990
Metriorrhynchini	Metriorrhynchus	sp.	UPOL	A00049	Malaysia	KC538064	KC537856	KC538736	KC538354	KC538545
Metriorrhynchini	Cautires	sp.	UPOL	A00050	Malaysia	KC538065	KC537857	–	–	KC538546
Metriorrhynchini	Leptotrichalus	sp.	UPOL	A00052	Philippines	KC538066	KC537858	HQ456949	HQ456968	HQ456991
Metriorrhynchini	Xylobanus	sp.	UPOL	A00053	Palawan	KC538067	KC537859	KC538737	KC538355	KC538547
Metriorrhynchini	Xylobanus	sp.	UPOL	A00054	Palawan	KC538068	–	KC538738	–	KC538548
Metriorrhynchini	Cautires	sp.	UPOL	A00057	Philippines	KC538069	KC537860	–	KC538356	KC538549
Metriorrhynchini	Cautires	sp.	UPOL	A00058	Philippines	KC538070	KC537861	–	KC538357	KC538550
Metriorrhynchini	Leptotrichalus	sp.	UPOL	A00059	Philippines	KC538071	KC537862	KC538739	KC538358	KC538551
Metriorrhynchini	Leptotrichalus	sp.	UPOL	A00061	Philippines	KC538073	KC537864	KC538741	KC538360	KC538553
Metriorrhynchini	Cautires	sp.	UPOL	A00062	Philippines	KC538074	KC537865	KC538742	KC538361	KC538554
Metriorrhynchini	Metanoeus	sp.	UPOL	A00063	Philippines	KC538075	KC537866	–	KC538362	KC538555
Metriorrhynchini	Metanoeus	sp.	UPOL	A00064	Philippines	KC538076	KC537867	–	KC538363	KC538556
Metriorrhynchini	Metanoeus	sp.	UPOL	A00065	Philippines	KC538077	KC537868	–	KC538364	KC538557
Metriorrhynchini	Sulabanus	sp.	UPOL	A00066	Philippines	KC538078	KC537869	KC538743	KC538365	KC538558
Metriorrhynchini	Sulabanus	sp.	UPOL	A00067	Philippines	KC538079	KC537870	KC538744	KC538366	KC538559
Metriorrhynchini	Microtrichalus	sp.	UPOL	A00068	Philippines	KC538080	KC537871	KC538745	KC538367	KC538560
Metriorrhynchini	Microtrichalus	sp.	UPOL	A00069	Philippines	KC538081	KC537872	KC538746	KC538368	KC538561
Metriorrhynchini	Sulabanus	sp.	UPOL	A00070	Philippines	KC538082	KC537873	KC538747	KC538369	KC538562
Metriorrhynchini	Sulabanus	sp.	UPOL	A00071	Philippines	KC538083	KC537874	KC538748	KC538370	KC538563
Metriorrhynchini	Microtrichalus	sp.	UPOL	A00073	Philippines	KC538084	KC537875	KC538749	KC538371	–
Metriorrhynchini	Xylobanus	sp.	UPOL	A00074	Philippines	KC538085	KC537876	KC538750	–	–
Metriorrhynchini	Sulabanus	sp.	UPOL	A00075	Philippines	KC538086	KC537877	KC538751	KC538372	KC538564
Metriorrhynchini	Xylobanus	sp.	UPOL	A00076	Philippines	KC538087	KC537878	KC538752	KC538373	KC538565
Metriorrhynchini	Sulabanus	sp.	UPOL	A00077	Philippines	KC538088	KC537879	KC538753	KC538374	KC538566
Metriorrhynchini	Cautires	sp.	UPOL	A00078	Cameroon	KC538089	KC537880	KC538754	KC538375	KC538567
Metriorrhynchini	Cautires	sp.	UPOL	A00079	Cameroon	KC538090	KC537881	KC538755	KC538376	KC538568
Metriorrhynchini	Cautires	sp.	UPOL	A00080	Cameroon	KC538091	KC537882	HQ456950	HQ456969	HQ456992
Metriorrhynchini	Cautires	sp.	UPOL	A00081	Cameroon	KC538093	KC537884	KC538756	KC538377	KC538569
Metriorrhynchini	Cautires	sp.	UPOL	A00082	Cameroon	KC538092	KC537883	KC538757	KC538378	–
Metriorrhynchini	Cautires	sp.	UPOL	A00083	Cameroon	KC538094	KC537885	KC538758	KC538379	KC538570
Metriorrhynchini	Cautires	sp.	UPOL	A00084	Cameroon	KC538095	KC537886	KC538759	KC538380	KC538571
Metriorrhynchini	Cautires	sp.	UPOL	A00085	Cameroon	KC538096	KC537887	KC538760	KC538381	KC538572
Metriorrhynchini	Cautires	sp.	UPOL	A00086	Cameroon	KC538097	KC537888	KC538761	KC538382	KC538573
Metriorrhynchini	Cautires	sp.	UPOL	A00087	Cameroon	KC538098	KC537889	KC538762	KC538383	KC538574
Metriorrhynchini	Cautires	sp.	UPOL	A00088	Cameroon	KC538099	KC537890	KC538763	KC538384	KC538575
Metriorrhynchini	Cautires	sp.	UPOL	A00089	Cameroon	KC538100	KC537891	KC538764	KC538385	KC538576
Metriorrhynchini	Cautires	sp.	UPOL	A00090	Cameroon	KC538101	KC537892	KC538765	KC538386	KC538577
Metriorrhynchini	Xylobanus	sp.	UPOL	A00091	Cameroon	KC538102	KC537893	–	KC538387	KC538578
Metriorrhynchini	Cautires	sp.	UPOL	A00092	Cameroon	KC538103	KC537894	KC538766	KC538388	KC538579
Metriorrhynchini	Cautires	sp.	UPOL	A00093	Cameroon	KC538104	KC537895	KC538767	KC538389	KC538580
Metriorrhynchini	Xylobanus	sp.	UPOL	A00094	Cameroon	KC538105	KC537896	KC538768	KC538390	KC538581
Metriorrhynchini	Xylobanus	sp.	UPOL	A00095	Cameroon	KC538106	KC537897	KC538769	–	KC538582
Metriorrhynchini	Xylobanus	sp.	UPOL	A00096	Cameroon	KC538107	KC537898	KC538770	–	KC538583
Metriorrhynchini	Xylobanus	sp.	UPOL	A00097	Cameroon	KC538108	KC537899	KC538771	KC538391	KC538584
Metriorrhynchini	Xylobanus	sp.	UPOL	A00098	Cameroon	KC538109	KC537900	KC538772	KC538392	KC538585
Metriorrhynchini	Cautires	sp.	UPOL	A00099	Cameroon	KC538110	KC537901	KC538773	KC538393	KC538586
Metriorrhynchini	Cautires	sp.	UPOL	A00100	Cameroon	KC538111	–	KC538774	KC538394	KC538587
Metriorrhynchini	Cautires	sp.	UPOL	A00101	Cameroon	KC538112	KC537902	–	KC538395	–
Metriorrhynchini	Cautires	sp.	UPOL	A00102	Cameroon	KC538113	KC537903	–	KC538396	KC538588
Metriorrhynchini	Cautires	sp.	UPOL	A00103	Cameroon	KC538114	KC537904	–	KC538397	–
Metriorrhynchini	Xylobanus	sp.	UPOL	A00104	Cameroon	KC538115	KC537905	KC538775	–	KC538589
Metriorrhynchini	Cautires	sp.	UPOL	A00105	Cameroon	KC538116	KC537906	KC538776	–	KC538590
Metriorrhynchini	Cautires	sp.	UPOL	A00106	Cameroon	KC538117	KC537907	KC538777	KC538398	KC538591
Metriorrhynchini	Cautires	sp.	UPOL	A00107	Cameroon	KC538118	KC537908	–	KC538399	–
Metriorrhynchini	Cautires	sp.	UPOL	A00109	Cameroon	KC538119	KC537909	KC538778	KC538400	KC538592
Metriorrhynchini	Cautires	sp.	UPOL	A00110	Cameroon	KC538120	KC537910	KC538779	–	KC538593
Metriorrhynchini	Cautires	sp.	UPOL	A00111	Cameroon	KC538121	KC537911	KC538780	KC538401	KC538594
Metriorrhynchini	Cautires	sp.	UPOL	A00112	Cameroon	KC538122	KC537912	KC538781	KC538402	KC538595
Metriorrhynchini	Metriorrhynchus	sp.	UPOL	A00116	PNG	ab123456	ab123456	ab123456	–	ab123456

Metriorrhynchini	Metriorrhynchus	sp.	UPOL	A00120	PNG	ab123456	ab123456	ab123456	–	ab123456
Metriorrhynchini	Metriorrhynchus	sp.	UPOL	A00128	PNG	ab123456	ab123456	ab123456	ab123456	ab123456
Metriorrhynchini	Metriorrhynchus	sp.	UPOL	A00132	PNG	ab123456	ab123456	ab123456	ab123456	ab123456
Metriorrhynchini	Metriorrhynchus	sp.	UPOL	A00148	PNG	ab123456	ab123456	ab123456	ab123456	ab123456
Metriorrhynchini	Metriorrhynchus	sp.	UPOL	A00160	PNG	ab123456	ab123456	–	ab123456	ab123456
Metriorrhynchini	Metriorrhynchus	sp.	UPOL	A00164	PNG	ab123456	ab123456	ab123456	ab123456	ab123456
Metriorrhynchini	Metriorrhynchus	sp.	UPOL	A00185	PNG	ab123456	ab123456	ab123456	ab123456	–
Metriorrhynchini	Cautires	apterus	UPOL	A00652	Tanzania	–	–	KF588381	–	KF588386
Metriorrhynchini	Cautires	apterus	UPOL	A00653	Tanzania	–	–	KF588382	–	KF588387
Metriorrhynchini	Cautires	apterus	UPOL	A00655	Tanzania	–	–	–	KF588384	KF588388
Metriorrhynchini	Cautires	apterus	UPOL	A00656	Tanzania	–	–	KF588383	KF588385	KF588389
Metriorrhynchini	Xylobanus	kundratai	UPOL	MD0029	Sulawesi	KC538204	KC537993	–	HQ456972	HQ456994
Metriorrhynchini	Sulabanus	lalui	UPOL	MD0030	Sulawesi	KC538205	KC537994	–	KC538403	KC538596
Metriorrhynchini	Sulabanus	katarinae	UPOL	MD0033	Sulawesi	KC538206	KC537995	KC538783	KC538404	KC538597
Metriorrhynchini	Sulabanus	lineatus	UPOL	MD0034	Sulawesi	KC538207	KC537996	KC538784	KC538405	KC538598
Metriorrhynchini	Xylobanus	kundratai	UPOL	MD0036	Sulawesi	KC538208	KC537997	–	HQ456973	HQ456995
Metriorrhynchini	Sulabanus	mamasensis	UPOL	MD0044	Sulawesi	KC538209	KC537998	KC538785	KC538406	KC538599
Metriorrhynchini	Sulabanus	gracilis	UPOL	MD0064	Sulawesi	KC538210	KC537999	KC538786	KC538407	KC538600
Metriorrhynchini	Sulabanus	similis	UPOL	MD0065	Sulawesi	KC538211	KC538000	–	KC538408	KC538601
Metriorrhynchini	Sulabanus	gracilis	UPOL	MD0067	Sulawesi	KC538212	KC538001	KC538787	KC538409	KC538602
Metriorrhynchini	Sulabanus	cordatus	UPOL	MD0069	Sulawesi	KC538213	KC538002	KC538788	KC538410	KC538603
Metriorrhynchini	Sulabanus	gracilis	UPOL	MD0071	Sulawesi	KC538214	KC538003	KC538789	KC538411	KC538604
Metriorrhynchini	Sulabanus	cordatus	UPOL	MD0081	Sulawesi	KC538215	KC538004	KC538790	KC538412	KC538605
Metriorrhynchini	Microtrichalus	sp.	UPOL	MD0097	Sulawesi	KC538216	KC538005	KC538791	HQ456978	HQ457000
Metriorrhynchini	Microtrichalus	sp.	UPOL	MD0098	Sulawesi	KC538217	KC538006	HQ456956	HQ456979	HQ457001
Metriorrhynchini	Broxylus	pfeifferi	UPOL	MD0099	Sulawesi	KC538218	KC538007	HQ456957	HQ456980	HQ457002
Metriorrhynchini	Broxylus	malinensis	UPOL	MD0101	Sulawesi	KC538219	KC538008	HQ456958	HQ456981	HQ457003
Metriorrhynchini	Broxylus	pendolensis	UPOL	MD0106	Sulawesi	KC538220	KC538009	KC538792	KC538413	KC538606
Metriorrhynchini	Broxylus	kalamensis	UPOL	MD0107	Sulawesi	KC538221	KC538010	KC538793	KC538414	KC538607
Metriorrhynchini	Wakarumbia	pendolensis	UPOL	MD0109	Sulawesi	KC538222	KC538011	KC538794	KC538415	KC538608
Metriorrhynchini	Wakarumbia	monacha	UPOL	MD0111	Sulawesi	KC538223	KC538012	KC538795	KC538416	KC538609
Metriorrhynchini	Wakarumbia	petri	UPOL	MD0118	Sulawesi	KC538224	KC538013	HQ456960	HQ456983	HQ457005
Metriorrhynchini	Wakarumbia	montana	UPOL	MD0119	Sulawesi	KC538225	KC538014	HQ456961	HQ456984	HQ457006
Metriorrhynchini	Wakarumbia	fasciata	UPOL	MD0121	Sulawesi	KC538226	KC538015	KC538796	KC538418	KC538610
Metriorrhynchini	Wakarumbia	sp.	UPOL	MD0126	Sulawesi	KC538227	KC538016	KC538797	KC538419	KC538611
Metriorrhynchini	Wakarumbia	grisea	UPOL	MD0127	Sulawesi	KC538228	KC538017	–	KC538420	KC538612
Metriorrhynchini	Wakarumbia	nepeensis	UPOL	MD0129	Sulawesi	KC538229	KC538018	KC538798	KC538421	KC538613
Metriorrhynchini	Wakarumbia	kundratai	UPOL	MD0130	Sulawesi	KC538230	KC538019	KC538799	KC538422	KC538614
Metriorrhynchini	Wakarumbia	sp.	UPOL	MD0132	Sulawesi	KC538231	KC538020	KC538800	KC538423	KC538615
Metriorrhynchini	Wakarumbia	kalamensis	UPOL	MD0133	Sulawesi	KC538232	KC538021	KC538801	KC538424	KC538616
Metriorrhynchini	Wakarumbia	linearis	UPOL	MD0134	Sulawesi	KC538233	KC538022	KC538802	KC538425	KC538617
Metriorrhynchini	Wakarumbia	nepeensis	UPOL	MD0135	Sulawesi	KC538234	KC538023	KC538803	KC538426	KC538618
Metriorrhynchini	Wakarumbia	aurea	UPOL	MD0136	Sulawesi	KC538235	KC538024	KC538804	KC538427	KC538619
Metriorrhynchini	Wakarumbia	aurea	UPOL	MD0137	Sulawesi	KC538236	KC538025	KC538805	KC538428	KC538620
Metriorrhynchini	Wakarumbia	fascicularis	UPOL	MD0140	Sulawesi	KC538237	KC538026	KC538806	KC538429	KC538621
Metriorrhynchini	Wakarumbia	pendolensis	UPOL	MD0143	Sulawesi	KC538238	KC538027	KC538807	KC538430	KC538622
Metriorrhynchini	Wakarumbia	fascicularis	UPOL	MD0145	Sulawesi	KC538239	KC538028	KC538808	KC538431	KC538623
Metriorrhynchini	Wakarumbia	mamasensis	UPOL	MD0155	Sulawesi	KC538240	KC538029	KC538809	KC538432	KC538624
Metriorrhynchini	Wakarumbia	grisea	UPOL	MD0156	Sulawesi	KC538241	KC538030	KC538810	KC538433	KC538625
Metriorrhynchini	Wakarumbia	mamasensis	UPOL	MD0157	Sulawesi	KC538242	KC538031	KC538811	KC538434	KC538626
Metriorrhynchini	Wakarumbia	kalamensis	UPOL	MD0169	Sulawesi	KC538243	KC538032	KC538812	KC538435	KC538627
Metriorrhynchini	Xylometanoeus	japonicus	UPOL	VK0038	Japan	–	–	–	KF652116	KF652124
Metriorrhynchini	Xylometanoeus	japonicus	UPOL	VK0039	Japan	–	–	–	KF652117	KF652125
Metriorrhynchini	Xylometanoeus	basivittatus	UPOL	VK0075	Japan	–	–	KF652135	KF652115	KF652123
Metriorrhynchini	Xylometanoeus	japonicus	UPOL	VK0090	Japan	–	–	–	KF652118	KF652126
Metriorrhynchini	Xylometanoeus	japonicus	UPOL	VK0093	Japan	–	–	–	KF652119	KF652127
Metriorrhynchini	Matsudanoeus	yuasai	UPOL	VK0248	Japan	–	–	KF652136	–	KF652130
Metriorrhynchini	Matsudanoeus	yuasai	UPOL	VK0249	Japan	–	–	KF652137	KF652120	KF652131
Metriorrhynchini	Matsudanoeus	yuasai	UPOL	VK0395	Japan	–	–	–	–	KF652132
Metriorrhynchini	Leptotrichalus	sp.	UPOL	ZL2002	Java	–	–	EF143215	EF143230	EF143244
Metriorrhynchini	Cautires	sp.	UPOL	ZL2009	Sumatra	–	–	EF143219	EF143234	EF143248
Metriorrhynchini	Sulabanus	sp.	UPOL	ZL2010	Sulawesi	–	–	EF143220	EF143235	EF143249
Platerodini	Plateros	sp.	UPOL	000031	Borneo	DQ181059	DQ181133	DQ180985	DQ181207	DQ181361
Platerodini	Plateros	sp.	UPOL	000243	Borneo	DQ181065	DQ181139	DQ180991	DQ181213	DQ181367
Platerodini	Plateros	sp.	UPOL	000303	Sumatra	DQ181067	DQ181141	DQ180993	DQ181215	DQ181369
Platerodini	Plateros	sp.	UPOL	001031	USA	DQ181109	DQ181183	DQ181035	DQ181257	DQ181411
Platerodini	Plateros	sp.	UPOL	000L13	Borneo	DQ181044	DQ181118	DQ180970	DQ181192	DQ181346
Platerodini	Plateros	sp.	UPOL	A00470	Malaysia	KT752153	KT752311	KT751980	KT751663	KT751825
Platerodini	Plateros	sp.	UPOL	A00471	Malaysia	KT752154	KT752312	KT751981	KT751664	KT751826
Platerodini	Plateros	sp.	UPOL	A00472	Malaysia	KT752155	KT752313	KT751982	KT751665	–
Platerodini	Plateros	sp.	UPOL	A00473	Malaysia	–	KT752314	KT751983	KT751666	KT751827

Platerodini	Plateros	sp.	UPOL	A00474	Malaysia	KT752156	KT752315	KT751984	KT751667	–
Platerodini	Plateros	sp.	UPOL	A00475	Malaysia	KT752157	KT752316	KT751985	–	KT751828
Platerodini	Plateros	sp.	UPOL	A00476	Malaysia	KT752158	KT752317	KT751986	KT751668	–
Platerodini	Plateros	sp.	UPOL	A00478	Vietnam	KT752160	KT752319	KT751988	KT751670	KT751830
Platerodini	Plateros	sp.	UPOL	A00479	Vietnam	KT752161	KT752320	KT751989	KT751671	KT751831
Platerodini	Plateros	sp.	UPOL	A00480	Laos	KT752162	KT752321	KT751990	KT751672	KT751832
Platerodini	Plateros	sp.	UPOL	A00481	Laos	KT752163	KT752322	KT751991	–	KT751833
Platerodini	Plateros	sp.	UPOL	A00482	Laos	–	KT752323	KT751992	KT751673	KT751834
Platerodini	Plateros	sp.	UPOL	A00483	Laos	KT752164	–	KT751993	KT751674	KT751835
Platerodini	Plateros	sp.	UPOL	A00484	Thailand	KT752165	KT752324	KT751994	KT751675	KT751836
Platerodini	Plateros	sp.	UPOL	A00485	India	KT752166	KT752325	KT751995	KT751676	KT751837
Platerodini	Plateros	sp.	UPOL	A00486	India	–	KT752326	KT751996	KT751677	KT751838
Platerodini	Plateros	sp.	UPOL	A00487	India	KT752167	KT752327	KT751997	KT751678	KT751839
Platerodini	Plateros	sp.	UPOL	A00488	Taiwan	–	KT752328	KT751998	KT751679	KT751840
Platerodini	Plateros	sp.	UPOL	A00489	Taiwan	KT752168	KT752329	KT751999	KT751680	KT751841
Platerodini	Plateros	sp.	UPOL	A00490	Taiwan	KT752169	KT752330	KT752000	KT751681	KT751842
Platerodini	Plateros	sp.	UPOL	A00491	Japan	KT752170	KT752331	KT752001	KT751682	KT751843
Platerodini	Plateros	sp.	UPOL	A00492	Japan	KT752171	KT752332	KT752002	KT751683	KT751844
Platerodini	Plateros	sp.	UPOL	A00493	Kenya	KT752172	KT752333	KT752003	KT751684	KT751845
Platerodini	Plateros	sp.	UPOL	A00494	Cameroon	KT752173	KT752334	KT752004	KT751685	KT751846
Platerodini	Plateros	sp.	UPOL	A00495	Cameroon	KT752174	KT752335	KT752005	KT751686	KT751847
Platerodini	Plateros	sp.	UPOL	A00496	Cameroon	KT752175	KT752336	KT752006	KT751687	–
Platerodini	Plateros	sp.	UPOL	A00497	Cameroon	KT752176	KT752337	KT752007	KT751688	–
Platerodini	Plateros	sp.	UPOL	A00498	Cameroon	KT752177	KT752338	KT752008	KT751689	KT751848
Platerodini	Plateros	sp.	UPOL	A00499	Zambia	KT752178	KT752339	KT752009	KT751690	KT751849
Platerodini	Plateros	sp.	UPOL	A00500	Zambia	KT752179	KT752340	KT752010	KT751691	KT751850
Platerodini	Plateros	sp.	UPOL	A00501	RSA	KT752180	KT752341	KT752011	KT751692	KT751851
Platerodini	Plateros	sp.	UPOL	A00502	Cameroon	KT752181	KT752342	KT752012	KT751693	KT751852
Platerodini	Plateros	sp.	UPOL	A00503	Cameroon	KT752182	KT752343	KT752013	KT751694	KT751853
Platerodini	Plateros	sp.	UPOL	A00504	Cameroon	KT752183	KT752344	KT752014	KT751695	KT751854
Platerodini	Plateros	sp.	UPOL	A00505	Canada	KT752184	KT752345	KT752015	KT751696	KT751855
Platerodini	Plateros	sp.	UPOL	A00507	Canada	KT752185	KT752346	KT752016	KT751697	KT751856
Platerodini	Plateros	sp.	UPOL	A00509	Argentina	KT752186	KT752347	KT752017	KT751698	KT751857
Platerodini	Plateros	sp.	UPOL	A00510	Argentina	KT752187	KT752348	KT752018	KT751699	KT751858
Platerodini	Plateros	sp.	UPOL	A00511	Costa Rica	KT752188	KT752349	KT752019	KT751700	KT751859
Platerodini	Plateros	sp.	UPOL	A00512	Costa Rica	KT752189	KT752350	KT752020	KT751701	KT751860
Platerodini	Plateros	sp.	UPOL	A00513	Panama	KT752190	KT752351	KT752021	KT751702	KT751861
Platerodini	Plateros	sp.	UPOL	A00555	Thailand	KT752221	KT752386	KT752053	KT751732	KT751890
Platerodini	Plateros	sp.	UPOL	A00579	Nicaragua	KT752245	KT752409	KT752077	KT751754	KT751911
Platerodini	Plateros	sp.	UPOL	A00580	USA	KT752246	KT752410	KT752078	KT751755	KT751912
Platerodini	Plateros	sp.	UPOL	A00619	Ecuador	–	KT752448	KT752117	KT751791	KT751950
Platerodini	Plateros	sp.	UPOL	A00632	Ecuador	KT752290	KT752455	–	KT751802	KT751961
Platerodini	Plateros	sp.	UPOL	A00634	Ecuador	KT752292	KT752457	KT752130	KT751804	KT751963
Platerodini	Plateros	sp.	UPOL	A00635	Nicaragua	KT752293	KT752458	KT752131	KT751805	KT751964
Platerodini	Plateros	sp.	UPOL	A00639	Ecuador	–	–	KT752135	KT751808	KT751966
Platerodini	Plateros	sp.	UPOL	A00643	Ecuador	–	KT752461	KT752138	KT751811	KT751969
Platerodini	Plateros	sp.	UPOL	A00646	Ecuador	KT752297	–	KT752140	KT751813	KT751971
Platerodini	Plateros	sp.	UPOL	RK0377	French Guy	KF625685	KF626271	KF625975	KF625384	–
Platerodini	Plateros	sp.	UPOL	ZL2012	Japan	–	–	EF143222	EF143237	EF143251
Platerodini	Plateros	sp.	UPOL	ZL2018	Japan	–	–	EF143227	EF143241	EF143255
Platerodrilini	Platerodrilus	palawanensis	UPOL	000371	Philippines	–	–	–	–	KF802552
Platerodrilini	Platerodrilus	strbai	UPOL	000472	Borneo	–	–	–	KF802537	KF802561
Platerodrilini	Macrolibnetis	depressus	UPOL	000515	Malaysia	–	–	–	FJ390411	FJ390413
Platerodrilini	Platerodrilus	ijenensis	UPOL	000586	Java	DQ181089	DQ181163	DQ181015	DQ181237	DQ181391
Platerodrilini	Platerodrilus	ranauensis	UPOL	000587	Sumatra	DQ181090	DQ181164	DQ181016	DQ181238	DQ181392
Platerodrilini	Platerodrilus	foliaceus	UPOL	000588	Borneo	DQ181091	DQ181165	DQ181017	DQ181239	DQ181393
Platerodrilini	Platerodrilus	foliaceus	UPOL	000589	Borneo	–	–	EF143214	EF143229	EF143243
Platerodrilini	Horakiella	emasensis	UPOL	001043	Borneo	DQ181110	DQ181184	DQ181036	DQ181258	DQ181412
Platerodrilini	Platerodrilus	montanus	UPOL	001371	Sumatra	KF625700	KF626286	KF625989	KF625398	–
Platerodrilini	Platerodrilus	sibayakensis	UPOL	001372	Sumatra	KF625701	KF626287	KF625990	KF625399	–
Platerodrilini	Platerodrilus	corporaali	UPOL	001373	Sumatra	KF625702	KF626288	KF625991	KF625400	–
Platerodrilini	Platerodrilus	maninjauensis	UPOL	001374	Sumatra	KF625703	KF626289	KF625992	KF625401	–
Platerodrilini	Platerodrilus	talamauensis	UPOL	001375	Sumatra	KF625780	KF626364	KF626072	KF625473	–
Platerodrilini	Platerodrilus	talamauensis	UPOL	001376	Sumatra	KF625704	KF626290	KF625993	KF625402	–
Platerodrilini	Platerodrilus	maninjauensis	UPOL	001377	Sumatra	KF625705	KF626291	KF625994	–	–
Platerodrilini	Platerodrilus	robinsoni	UPOL	001378	Sumatra	KF625706	KF626292	KF625995	–	–
Platerodrilini	Platerodrilus	luteus	UPOL	001379	Sumatra	KF625707	KF626293	KF625996	KF625403	–
Platerodrilini	Platerodrilus	curtus	UPOL	001380	Philippines	KF625708	KF626294	KF625997	KF625404	–
Platerodrilini	Platerodrilus	curtus	UPOL	001381	Philippines	KF625781	KF626365	KF626073	–	–
Platerodrilini	Platerodrilus	curtus	UPOL	001383	Philippines	KF625782	KF626366	KF626074	KF625474	–
Platerodrilini	Platerodrilus	atricolor	UPOL	001384	Malaysia	KF625710	KF626296	–	–	–

Platerodrilini	Platerodrilus	tujuhensis	UPOL	001385	Sumatra	KF625711	KF626297	KF625999	KF625405	–
Platerodrilini	Platerodrilus	major	UPOL	001387	Sumatra	KF625712	KF626298	KF626000	–	–
Platerodrilini	Platerodrilus	angustatus	UPOL	001388	Sumatra	KF625713	KF626299	KF626001	KF625406	–
Platerodrilini	Platerodrilus	sibayakensis	UPOL	001389	Sumatra	KF625784	KF626367	–	KF625475	–
Platerodrilini	Platerodrilus	sp.	UPOL	000L01	Borneo	DQ181037	DQ181111	DQ180963	DQ181185	DQ181339
Platerodrilini	Macrolibnetis	depressus	UPOL	000L21	Malaysia	DQ181050	DQ181124	DQ180976	DQ181198	DQ181352
Platerodrilini	Pendola	sp.	UPOL	000M45	Java	DQ181058	DQ181132	DQ180984	DQ181206	DQ181360
Platerodrilini	Platerodrilus	sp.	UPOL	A00575	Malaysia	KT752241	KT752406	KT752073	KT751750	KT751907
Platerodrilini	Platerodrilus	sp.	UPOL	A00576	Malaysia	KT752242	KT752407	KT752074	KT751751	KT751908
Platerodrilini	Platerodrilini	gen. sp.	UPOL	VP0009	Malaysia	KF802497	KF802507	KF802457	KF802527	KF802539
Platerodrilini	Platerodrilini	gen. sp.	UPOL	VP0010	Sumatra	KF802498	KF802508	KF802480	KF802533	KF802542
Platerodrilini	Platerodrilini	gen. sp.	UPOL	VP0012	Malaysia	KF802499	KF802509	KF802458	KF802528	KF802541
Platerodrilini	Platerodrilini	gen. sp.	UPOL	VP0013	Malaysia	KF802500	KF802510	–	KF802529	KF802540
Platerodrilini	Platerodrilus	sp.	UPOL	VP0014	Philippines	KF802482	KF802519	KF802459	–	KF802553
Platerodrilini	Platerodrilus	sp.	UPOL	VP0020	Malaysia	KF802501	–	KF802460	KF802535	KF802554
Platerodrilini	Platerodrilus	ngi	UPOL	VP0021	Singapore	KF802481	KF802520	KF802461	–	–
Platerodrilini	Platerodrilini	gen. sp.	UPOL	VP0030	India	KF802493	KF802511	KF802462	KF802530	KF802543
Platerodrilini	Platerodrilini	gen. sp.	UPOL	VP0031	India	KF802495	KF802512	KF802463	KF802531	–
Platerodrilini	Platerodrilini	gen. sp.	UPOL	VP0034	India	KF802496	KF802513	KF802464	KF802532	KF802544
Platerodrilini	Platerodrilus	sp.	UPOL	VP0044	Borneo	KF802505	–	KF802465	–	KF802550
Platerodrilini	Platerodrilus	sp.	UPOL	VP0047	Sumatra	KF802502	–	KF802466	–	KF802555
Platerodrilini	Macrolibnetis	depressus	UPOL	VP0050	Malaysia	KF802504	KF802506	KF802467	–	KF802538
Platerodrilini	Platerodrilus	sp.	UPOL	VP2301	Borneo	KF802483	KF802518	KF802468	–	KF802551
Platerodrilini	Platerodrilus	sp.	UPOL	VP2302	Malaysia	KF802484	KF802521	KF802469	–	KF802556
Platerodrilini	Platerodrilus	maninjauensis	UPOL	VP2303	Sumatra	KF802485	KF802514	KF802470	–	KF802545
Platerodrilini	Platerodrilus	sp.	UPOL	VP2304	Thailand	KF802486	KF802522	KF802471	–	–
Platerodrilini	Platerodrilus	tujuhensis	UPOL	VP2305	Sumatra	KF802494	KF802523	KF802472	–	KF802548
Platerodrilini	Platerodrilus	maninjauensis	UPOL	VP2306	Sumatra	KF802487	KF802515	KF802473	–	KF802546
Platerodrilini	Platerodrilus	maninjauensis	UPOL	VP2307	Sumatra	KF802488	KF802516	KF802474	–	KF802547
Platerodrilini	Platerodrilus	montanus	UPOL	VP2308	Sumatra	KF802489	KF802517	KF802475	–	KF802549
Platerodrilini	Platerodrilus	sp.	UPOL	VP2309	Malaysia	KF802490	KF802524	KF802476	–	KF802557
Platerodrilini	Platerodrilus	sp.	UPOL	VP2310	Malaysia	KF802491	KF802525	KF802477	KF802536	KF802558
Platerodrilini	Platerodrilus	sp.	UPOL	VP2311	Laos	KF802503	–	KF802478	–	KF802559
Platerodrilini	Platerodrilus	sp.	UPOL	VP2316	Philippines	KF802492	KF802526	KF802479	–	KF802560
Slipinskiini	Flagrax	sp.	UPOL	000L26	RSA	DQ181054	DQ181128	DQ180980	DQ181202	DQ181356
Slipinskiini	Flagrax	sp.	UPOL	A00561	RSA	KT752227	KT752392	KT752059	KT751737	KT751894
Slipinskiini	Flagrax	sp.	UPOL	A00562	Cameroon	KT752228	KT752393	KT752060	KT751738	KT751895
Slipinskiini	Flagrax	sp.	UPOL	A00563	Cameroon	KT752229	KT752394	KT752061	KT751739	KT751896
Slipinskiini	Flagrax	sp.	UPOL	A00564	RSA	KT752230	KT752395	KT752062	KT751740	KT751897
Slipinskiini	Flagrax	sp.	UPOL	A00565	RSA	KT752231	KT752396	KT752063	KT751741	KT751898
Slipinskiini	Flagrax	sp.	UPOL	A00566	RSA	KT752232	KT752397	KT752064	KT751742	KT751899
Taphini	Taphes	brevicollis	UPOL	000812	Laos	DQ181098	DQ181172	DQ181024	DQ181246	DQ181400
Taphini	Protaphes	sp.	UPOL	A00525	Laos	KT752195	KT752356	KT752025	KT751707	–
Taphini	Taphes	brevicollis	UPOL	A00526	Laos	KT752196	KT752357	–	KT751708	KT751864
Taphini	Protaphes	sp.	UPOL	A00527	Sumatra	KT752197	KT752358	KT752026	KT751709	–
Taphini	Taphes	brevicollis	UPOL	A00528	Laos	KT752198	KT752359	KT752027	KT751710	KT751865
Taphini	Taphes	sp.	UPOL	A00529	India	KT752199	KT752360	KT752028	KT751711	KT751866
Taphini	Protaphes	sp.	UPOL	A00531	Malaysia	KT752201	KT752362	KT752030	KT751713	KT751868
Taphini	Protaphes	sp.	UPOL	A00535	Sumatra	KT752204	KT752366	KT752034	KT751717	KT751872
Taphini	Taphes	brevicollis	UPOL	A00536	Sumatra	KT752205	KT752367	KT752035	KT751718	KT751873
Taphini	Taphes	sp.	UPOL	A00537	China	KT752206	KT752368	KT752036	–	KT751874
Taphini	Taphes	brevicollis	UPOL	A00538	Sumatra	KT752207	KT752369	–	KT751719	KT751875
Taphini	Taphes	sp.	UPOL	A00539	Laos	KT752208	KT752370	KT752037	–	–
Taphini	Protaphes	sp.	UPOL	A00543	Malaysia	KT752212	KT752374	KT752041	KT751722	KT751878
Taphini	Protaphes	sp.	UPOL	A00544	Malaysia	KT752213	KT752375	KT752042	KT751723	KT751879
Taphini	Protaphes	sp.	UPOL	A00573	Laos	KT752239	KT752404	KT752071	KT751748	KT751905
Thonalmini	Thonalmus	sinuaticostis	UPOL	000594	Montserrat	DQ181093	DQ181167	DQ181019	DQ181241	DQ181395
Thonalmini	Thonalmus	hubbardi	UPOL	000595	Montserrat	DQ181094	DQ181168	DQ181020	DQ181242	DQ181396
Thonalmini	Thonalmus	sp.	UPOL	A00592	Cuba	KT752258	KT752422	KT752090	KT751767	KT751924
Thonalmini	Thonalmus	sp.	UPOL	A00593	Cuba	KT752259	KT752423	KT752091	KT751768	KT751925
Thonalmini	Thonalmus	sp.	UPOL	A00595	Dominican	KT752261	KT752425	KT752093	KT751769	KT751927

Table S3. The partition scheme, the best substution models and information about IQ-tree run.

Fragment	# of Seq	# of Site	Unique	Infor	Invar	Const	Model
ND1	501	125	123	99	13	13	TIM+F+I+G4
COX2	542	263	252	219	29	29	TIM+F+I+G4
COX1-2-TRNA	538	61	44	34	23	23	GTR+F+I+G4
NADH5	652	1032	975	863	108	108	GTR+F+I+G4
NADH5	677	780	601	522	221	221	GTR+F+I+G4
NADH5-TRNAs	633	358	299	234	103	103	TIM3+F+I+G4
rrnl-ND1-TRNA	546	70	64	50	13	13	GTR+F+I+G4
rrnl	710	678	585	419	190	190	GTR+F+I+G4
18S	583	1953	770	352	1434	1434	SYM+I+G4
28S	580	667	299	180	438	438	TIM2e+I+G4

Column meanings:
Unique: Number of unique site patterns
Infor: Number of parsimony-informative sites
Invar: Number of invariant sites
Const: Number of constant sites (can be subset of invariant sites)
Model: The best model chosen by ModelFinder according to BIC (Bayesian information criterion scores and weights)

Table S4. The list of samples with details of colour patterns.

Voucher	Colour-Pattern	Simplified Colour-pattern	Major Colour	Uni	PvE	BiElyt	Fasci	Stria	N-Cat
Iberobaenia	Uni-Black	Uni	Black	Yes	No	No	No	No	No
000543	Uni-Black	Uni	Black	Yes	No	No	No	No	No
A00654	Uni-Yellow	Uni	Yellow	Yes	No	No	No	No	No
A00601	Uni-Yellow	Uni	Yellow	Yes	No	No	No	No	No
A00566	B1-Black/Red	pronVSelyt	Black	No	Yes	No	No	No	No
A00563	B2-Red/Black	BiElyt	Red	No	No	Yes	No	No	No
A00565	B1-Black/Red	pronVSelyt	Black	No	Yes	No	No	No	No
000L26	B2-Yellow/Black	BiElyt	Yellow	No	No	Yes	No	No	No
A00560	B1-Black/red	pronVSelyt	Red	No	Yes	No	No	No	No
MK1110	Uni-Red	Uni	Red	Yes	No	No	No	No	No
000575	Uni-Red	Uni	Red	Yes	No	No	No	No	No
A00553	Uni-Red	Uni	Red	Yes	No	No	No	No	No
A00556	Uni-Red	Uni	Red	Yes	No	No	No	No	No
000578	Uni-Black	Uni	Black	Yes	No	No	No	No	No
A00551	Uni-Yellow	Uni	Yellow	Yes	No	No	No	No	No
000580	Uni-Black	Uni	Black	Yes	No	No	No	No	No
000579	Uni-Black	Uni	Black	Yes	No	No	No	No	No
A00549	Uni-Black	Uni	Black	Yes	No	No	No	No	No
A00554	Uni-Black	Uni	Black	Yes	No	No	No	No	No
A00548	Uni-Black	Uni	Black	Yes	No	No	No	No	No
A00558	Uni-Black	Uni	Black	Yes	No	No	No	No	No
000348	B1-Black/red	pronVSelyt	Red	No	Yes	No	No	No	No
001366	Uni-Black	Uni	Black	Yes	No	No	No	No	No
A00557	B1-Black/red	pronVSelyt	Red	No	Yes	No	No	No	No
000576	Uni-Orange	Uni	Orange	Yes	No	No	No	No	No
001365	Uni-Orange	Uni	Orange	Yes	No	No	No	No	No
A00559	B1-Black/red	pronVSelyt	Red	No	Yes	No	No	No	No
A00568	B1-Black/red	pronVSelyt	Red	No	Yes	No	No	No	No
000572	Uni-Black	Uni	Black	Yes	No	No	No	No	No
A00569	B1-Black/red	pronVSelyt	Red	No	Yes	No	No	No	No
000573	B1-Black/red	pronVSelyt	Red	No	Yes	No	No	No	No
000574	B1-Black/red	pronVSelyt	Red	No	Yes	No	No	No	No
001277	B1-Black/red	pronVSelyt	Red	No	Yes	No	No	No	No
A00618	Uni-Red	Uni	Red	Yes	No	No	No	No	No
A00570	Uni-Red	Uni	Red	Yes	No	No	No	No	No
001369	Uni-Red	Uni	Red	Yes	No	No	No	No	No
ZL2013	Uni-Red	Uni	Red	Yes	No	No	No	No	No
A00540	Uni-Red	Uni	Red	Yes	No	No	No	No	No
001283	Uni-Red	Uni	Red	Yes	No	No	No	No	No
A00520	Uni-Black	Uni	Black	Yes	No	No	No	No	No
A00597	Uni-Black	Uni	Black	Yes	No	No	No	No	No
A00539	Uni-Black	Uni	Black	Yes	No	No	No	No	No
001275	Uni-Red	Uni	Red	Yes	No	No	No	No	No
001276	Uni-Red	Uni	Red	Yes	No	No	No	No	No
000571	B1-Black/Red	pronVSelyt	Red	No	Yes	No	No	No	No
000570	Uni-Red	Uni	Red	Yes	No	No	No	No	No
A00525	Uni-Red	Uni	Red	Yes	No	No	No	No	No
A00544	Uni-Black	Uni	Black	Yes	No	No	No	No	No

Legend
Uni - uniform coloured dorsal body side, i.e. the pronotum and elytra; **PvE** - bicoloured, i.e. the different colouration of the pronotum and elytra); **BiElyt** - bicoloured elytra, either humeri or the apex brightly coloured; **Fasci** - a fasciate pattern on elytra; **Stria** a striate elytral pattern; **N-Cat** - others patterns (non-categorized patterns, such as reticulate, tricolored, green, red and blue; for more information, see Supplementary information, Figs 1–914).

A00527	B1-Black/red	pronVSelyt	Red	No	Yes	No	No	No	No
A00535	B1-Black/red	pronVSelyt	Red	No	Yes	No	No	No	No
A00543	Uni-Black	Uni	Black	Yes	No	No	No	No	No
A00519	Uni-Red	Uni	Red	Yes	No	No	No	No	No
001282	Uni-Red	Uni	Red	Yes	No	No	No	No	No
A00529	Uni-Red	Uni	Red	Yes	No	No	No	No	No
A00537	Uni-Red	Uni	Red	Yes	No	No	No	No	No
A00538	B1-Black/red	pronVSelyt	Red	No	Yes	No	No	No	No
MT0076	Uni-Red	Uni	Red	Yes	No	No	No	No	No
MT0039	Uni-Red	Uni	Red	Yes	No	No	No	No	No
MT0069	B2-Red/Black	BiElyt	Red	No	No	Yes	No	No	No
MT0025	B1-Red/Metalic	pronVSelyt	Metalic	No	Yes	No	No	No	No
MT0047	Uni-Red	Uni	Red	Yes	No	No	No	No	No
MT0003	Uni-Red	Uni	Red	Yes	No	No	No	No	No
MT0045	B2-Red/Black	BiElyt	Red	No	No	Yes	No	No	No
MT0044	B2-Red/Black	BiElyt	Red	No	No	Yes	No	No	No
MT0032	B2-Red/Black	BiElyt	Red	No	No	Yes	No	No	No
MT0058	B1-Black/Yellow	pronVSelyt	Yellow	No	Yes	No	No	No	No
MT0061	Uni-Red	Uni	Red	Yes	No	No	No	No	No
MT0075	Uni-Red	Uni	Red	Yes	No	No	No	No	No
MT0030	B1-Black/Red	pronVSelyt	Red	No	Yes	No	No	No	No
MT0074	B2-Red/Black	BiElyt	Red	No	No	Yes	No	No	No
000L16	Uni-Red	Uni	Red	Yes	No	No	No	No	No
MT0038	Stria	Striate	Red	No	No	No	No	Yes	No
MT0073	B2-Red/Black	BiElyt	Red	No	No	Yes	No	No	No
MT0078	Uni-Red	Uni	Red	Yes	No	No	No	No	No
MT0077	Uni-Red	Uni	Red	Yes	No	No	No	No	No
MT0006	Uni-Red	Uni	Red	Yes	No	No	No	No	No
MT0053	Uni-Red	Uni	Red	Yes	No	No	No	No	No
MT0056	Uni-Red	Uni	Red	Yes	No	No	No	No	No
MT0026	Punct	Non-Cat	Red	No	No	No	No	No	Yes
MT0009	Uni-Red	Uni	Red	Yes	No	No	No	No	No
MT0062	Uni-Red	Uni	Red	Yes	No	No	No	No	No
MT0035	B2-Red/Black	BiElyt	Red	No	No	Yes	No	No	No
MT0048	B1-Black/Red	pronVSelyt	Red	No	Yes	No	No	No	No
MT0065	Uni-Red	Uni	Red	Yes	No	No	No	No	No
MT0011	Uni-Black	Uni	Black	Yes	No	No	No	No	No
MT0001	Stria	Striate	Red	No	No	No	No	Yes	No
MT0013	Stria	Striate	Red	No	No	No	No	Yes	No
MT0066	B2-Yellow/Black	BiElyt	Yellow	No	No	Yes	No	No	No
MT0072	B2-Yellow/Black	BiElyt	Yellow	No	No	Yes	No	No	No
MT0017	B1-Black/Yellow	pronVSelyt	Yellow	No	Yes	No	No	No	No
MT0067	B1-Black/Yellow	pronVSelyt	Yellow	No	Yes	No	No	No	No
MT0020	B2-Yellow/Black	BiElyt	Yellow	No	No	Yes	No	No	No
MT0021	B2-Yellow/Black	BiElyt	Yellow	No	No	Yes	No	No	No
MT0022	B2-Yellow/Black	BiElyt	Yellow	No	No	Yes	No	No	No
MT0033	B2-Yellow/Black	BiElyt	Yellow	No	No	Yes	No	No	No
MT0023	B2-Yellow/Black	BiElyt	Yellow	No	No	Yes	No	No	No
MT0019	B2-Yellow/Black	BiElyt	Yellow	No	No	Yes	No	No	No
MT0060	B2-Yellow/Black	BiElyt	Yellow	No	No	Yes	No	No	No

MT0052	Uni-Yellow	Uni	Yellow	Yes	No	No	No	No	No
MT0070	Uni-Yellow	Uni	Yellow	Yes	No	No	No	No	No
MT0043	Uni-Black	Uni	Black	Yes	No	No	No	No	No
MT0012	B2-Yellow/Black	BiElyt	Yellow	No	No	Yes	No	No	No
MT0034	B1-Black/Red	pronVSelyt	Red	No	Yes	No	No	No	No
MT0071	Uni-Red	Uni	Red	Yes	No	No	No	No	No
MT0064	B1-Black/Red	pronVSelyt	Red	No	Yes	No	No	No	No
MT0014	Reti	Non-Cat	Red	No	No	No	No	No	Yes
000347	Uni-Black	Uni	Black	Yes	No	No	No	No	No
MT0007	B2-Red/Black	BiElyt	Red	No	No	Yes	No	No	No
MT0037	Uni-Black	Uni	Black	Yes	No	No	No	No	No
MT0005	B2-Red/Black	BiElyt	Red	No	No	Yes	No	No	No
MT0068	B2-Red/Black	BiElyt	Red	No	No	Yes	No	No	No
MT0051	Uni-Red	Uni	Red	Yes	No	No	No	No	No
MT0057	Uni-Red	Uni	Red	Yes	No	No	No	No	No
VM0021	Uni-Black	Uni	Black	Yes	No	No	No	No	No
VM0022	Uni-Black	Uni	Black	Yes	No	No	No	No	No
000583	Uni-Black	Uni	Black	Yes	No	No	No	No	No
VM0005	Uni-Red	Uni	Red	Yes	No	No	No	No	No
VM0014	Uni-Red	Uni	Red	Yes	No	No	No	No	No
VM0019	Uni-Yellow	Uni	Yellow	Yes	No	No	No	No	No
000582	Uni-Yellow	Uni	Yellow	Yes	No	No	No	No	No
VM0049	Uni-Yellow	Uni	Yellow	Yes	No	No	No	No	No
VM0050	Uni-Yellow	Uni	Yellow	Yes	No	No	No	No	No
VM0002	Uni-Yellow	Uni	Yellow	Yes	No	No	No	No	No
VM0004	Uni-Yellow	Uni	Yellow	Yes	No	No	No	No	No
Scarelus	Uni-Yellow	Uni	Yellow	Yes	No	No	No	No	No
VM0007	B1-Black/Yellow	pronVSelyt	Yellow	No	Yes	No	No	No	No
VM0011	B1-Black/Yellow	pronVSelyt	Yellow	No	Yes	No	No	No	No
VM0026	Uni-Red	Uni	Red	Yes	No	No	No	No	No
VM0031	Uni-Red	Uni	Red	Yes	No	No	No	No	No
VM0012	B1-Black/Red	pronVSelyt	Red	No	Yes	No	No	No	No
VM0034	B1-Black/Red	pronVSelyt	Red	No	Yes	No	No	No	No
VM0023	B1-Black/Red	pronVSelyt	Red	No	Yes	No	No	No	No
VM0033	B1-Black/Red	pronVSelyt	Red	No	Yes	No	No	No	No
A00466	Fas-mid-Yell	Fasciate	Yellow	No	No	No	Yes	No	No
A00461	Uni-Orange	Uni	Orange	Yes	No	No	No	No	No
000828	Uni-Orange	Uni	Orange	Yes	No	No	No	No	No
YL0206	Uni-Orange	Uni	Orange	Yes	No	No	No	No	No
YL0403	B1-Black/Red	pronVSelyt	Red	No	Yes	No	No	No	No
YL0032	Uni-Red	Uni	Red	Yes	No	No	No	No	No
ZL2005	Uni-Red	Uni	Red	Yes	No	No	No	No	No
A00465	B1-Black/Red	pronVSelyt	Red	No	Yes	No	No	No	No
YL0185	Uni-Orange	Uni	Orange	Yes	No	No	No	No	No
YL0192	Uni-Orange	Uni	Orange	Yes	No	No	No	No	No
000L18	Uni-Red	Uni	Red	Yes	No	No	No	No	No
A00462	Uni-Yellow	Uni	Yellow	Yes	No	No	No	No	No
A00463	B1-Black/Red	pronVSelyt	Red	No	Yes	No	No	No	No
A00464	Uni-Red	Uni	Red	Yes	No	No	No	No	No
VK0733	Uni-Yellow	Uni	Yellow	Yes	No	No	No	No	No

YL0296	Uni-Red	Uni	Red	Yes	No	No	No	No	No
YL0319	Uni-Red	Uni	Red	Yes	No	No	No	No	No
000815	Uni-Red	Uni	Red	Yes	No	No	No	No	No
YL0314	Uni-Red	Uni	Red	Yes	No	No	No	No	No
000816	Uni-Red	Uni	Red	Yes	No	No	No	No	No
A00467	Uni-Red	Uni	Red	Yes	No	No	No	No	No
A00468	Uni-Red	Uni	Red	Yes	No	No	No	No	No
YL0259	B1-Black/Red	pronVSelyt	Red	No	Yes	No	No	No	No
000817	B2-Red/Black	BiElyt	Red	No	No	Yes	No	No	No
A00460	B1-Black/Red	pronVSelyt	Red	No	Yes	No	No	No	No
A00571	Uni-Red	Uni	Red	Yes	No	No	No	No	No
YL0217	B1-Black/Red	pronVSelyt	Red	No	Yes	No	No	No	No
YL0263	B1-Black/Red	pronVSelyt	Red	No	Yes	No	No	No	No
YL0282	Uni-Red	Uni	Red	Yes	No	No	No	No	No
000L17	B1-Black/Red	pronVSelyt	Red	No	Yes	No	No	No	No
YL0409	Uni-Red	Uni	Red	Yes	No	No	No	No	No
ZL2014	B1-Black/Red	pronVSelyt	Red	No	Yes	No	No	No	No
A00595	B1-Red/Metalic	pronVSelyt	Metalic	No	Yes	No	No	No	No
A00592	B1-Red/Metalic	pronVSelyt	Metalic	No	Yes	No	No	No	No
000594	B1-Red/Metalic	pronVSelyt	Metalic	No	Yes	No	No	No	No
000595	B1-Red/Metalic	pronVSelyt	Metalic	No	Yes	No	No	No	No
A00627	Stria	Striate	Yellow	No	No	No	No	Yes	No
A00649	B2-Yellow/Black	BiElyt	Yellow	No	No	Yes	No	No	No
A00641	Stria	Striate	Yellow	No	No	No	No	Yes	No
000M43	Fas-mid-Yell	Fasciate	Yellow	No	No	No	Yes	No	No
A00636	Fas-mid-Yell	Fasciate	Yellow	No	No	No	Yes	No	No
A00638	Uni-Yellow	Uni	Yellow	Yes	No	No	No	No	No
A00648	Stria	Striate	Yellow	No	No	No	No	Yes	No
A00616	Uni-Red	Uni	Red	Yes	No	No	No	No	No
A00615	Uni-Red	Uni	Red	Yes	No	No	No	No	No
A00608	Uni-Red	Uni	Red	Yes	No	No	No	No	No
A00611	Uni-Red	Uni	Red	Yes	No	No	No	No	No
A00606	Uni-Red	Uni	Red	Yes	No	No	No	No	No
A00607	Uni-Red	Uni	Red	Yes	No	No	No	No	No
000581	B2-Red/Black	BiElyt	Red	No	No	Yes	No	No	No
A00609	Uni-Black	Uni	Black	Yes	No	No	No	No	No
A00603	Uni-Black	Uni	Black	Yes	No	No	No	No	No
000194	Uni-Red	Uni	Red	Yes	No	No	No	No	No
A00605	Uni-Red	Uni	Red	Yes	No	No	No	No	No
A00610	Uni-Red	Uni	Red	Yes	No	No	No	No	No
A00601	Uni-Red	Uni	Red	Yes	No	No	No	No	No
A00612	Uni-Red	Uni	Red	Yes	No	No	No	No	No
A00614	Uni-Red	Uni	Red	Yes	No	No	No	No	No
A00631	Fas-mid-Yell	Fasciate	Yellow	No	No	No	Yes	No	No
A00650	B2-Yellow/Black	BiElyt	Yellow	No	No	Yes	No	No	No
000592	Uni-Black	Uni	Black	Yes	No	No	No	No	No
A00651	Fas-mid-Yell	Fasciate	Yellow	No	No	No	Yes	No	No
IR2002	Punct	Non-Cat	Yellow	No	No	No	No	No	Yes
000M44	Fas-mid-Yell	Fasciate	Yellow	No	No	No	Yes	No	No
A00628	Non-Cat	Non-Cat	Black	No	No	No	No	No	Yes

A00647	B2-Yellow/Black	BiElyt	Yellow	No	No	Yes	No	No	No
A00645	Fas-mid-Yell	Fasciate	Yellow	No	No	No	Yes	No	No
A00621	Fas-mid-Yell	Fasciate	Yellow	No	No	No	Yes	No	No
A00637	Fas-mid-Yell	Fasciate	Yellow	No	No	No	Yes	No	No
A00630	Fas-mid-Yell	Fasciate	Yellow	No	No	No	Yes	No	No
000L25	Fas-met-mid-Yell	Fasciate	Metalic	No	No	No	Yes	No	No
A00629	Fas-mid-Yell	Fasciate	Yellow	No	No	No	Yes	No	No
A00640	Stria	Striate	Yellow	No	No	No	No	Yes	No
LF0451	B2-Red/Black	BiElyt	Red	No	No	Yes	No	No	No
LF0454	Uni-Yellow	Uni	Yellow	Yes	No	No	No	No	No
LF0466	B2-Red/Black	BiElyt	Red	No	No	Yes	No	No	No
LF0429	B2-Yellow/Black	BiElyt	Yellow	No	No	Yes	No	No	No
LF0430	B2-Yellow/Black	BiElyt	Yellow	No	No	Yes	No	No	No
LF0303	Uni-Orange	Uni	Orange	Yes	No	No	No	No	No
LF0331	Uni-Red	Uni	Red	Yes	No	No	No	No	No
LF0370	Uni-Red	Uni	Red	Yes	No	No	No	No	No
LF0323	Uni-Red	Uni	Red	Yes	No	No	No	No	No
LF0388	B2-Yellow/Black	BiElyt	Yellow	No	No	Yes	No	No	No
LF0264	B2-Yellow/Black	BiElyt	Yellow	No	No	Yes	No	No	No
LF0266	B2-Yellow/Black	BiElyt	Yellow	No	No	Yes	No	No	No
LF0320	Uni-Yellow	Uni	Yellow	Yes	No	No	No	No	No
A00328	Stria	Striate	Yellow	No	No	No	No	Yes	No
LF0354	Uni-Red	Uni	Red	Yes	No	No	No	No	No
LF0340	Stria	Striate	Yellow	No	No	No	No	Yes	No
LF0365	B2-Red/Black	BiElyt	Red	No	No	Yes	No	No	No
000L27	B2-Yellow/Black	BiElyt	Yellow	No	No	Yes	No	No	No
LF0394	B2-Yellow/Black	BiElyt	Yellow	No	No	Yes	No	No	No
LF0035	B1-Black/Yellow	pronVSelyt	Yellow	No	Yes	No	No	No	No
LF0270	Fas-mid-Yell	Fasciate	Yellow	No	No	No	Yes	No	No
LF0031	B1-Black/Yellow	pronVSelyt	Yellow	No	Yes	No	No	No	No
LF0116	B1-Black/Yellow	pronVSelyt	Yellow	No	Yes	No	No	No	No
A00469	B1-Black/Yellow	pronVSelyt	Yellow	No	Yes	No	No	No	No
LF0262	B1-Black/Yellow	pronVSelyt	Yellow	No	Yes	No	No	No	No
LF0077	Fas-mid-Yell	Fasciate	Yellow	No	No	No	Yes	No	No
LF0143	B1-Black/Yellow	pronVSelyt	Yellow	No	Yes	No	No	No	No
LF0043	B1-Black/Yellow	pronVSelyt	Yellow	No	Yes	No	No	No	No
LF0167	Stria	Striate	Yellow	No	No	No	No	Yes	No
LF0168	Stria	Striate	Yellow	No	No	No	No	Yes	No
LF0444	Stria	Striate	Yellow	No	No	No	No	Yes	No
000L03	B1-Black/Yellow	pronVSelyt	Yellow	No	Yes	No	No	No	No
VP0024	B2-Yellow/Black	BiElyt	Yellow	No	No	Yes	No	No	No
VP0022	B2-Yellow/Black	BiElyt	Yellow	No	No	Yes	No	No	No
VP0027	B2-Yellow/Black	BiElyt	Yellow	No	No	Yes	No	No	No
A00634	B2-Yellow/Black	BiElyt	Yellow	No	No	Yes	No	No	No
A00472	Uni-Black	Uni	Black	Yes	No	No	No	No	No
000303	Uni-Black	Uni	Black	Yes	No	No	No	No	No
A00473	Uni-Black	Uni	Black	Yes	No	No	No	No	No
000031	B2-Yellow/Black	BiElyt	Yellow	No	No	Yes	No	No	No
A00497	B2-Yellow/Black	BiElyt	Yellow	No	No	Yes	No	No	No
A00498	B2-Yellow/Black	BiElyt	Yellow	No	No	Yes	No	No	No

A00501	B1-Bright/Black	pronVSelyt	Metalic	No	Yes	No	No	No	No
A00493	B2-Yellow/Black	BiElyt	Yellow	No	No	Yes	No	No	No
A00500	B2-Yellow/Black	BiElyt	Yellow	No	No	Yes	No	No	No
A00483	Uni-Yellow	Uni	Yellow	Yes	No	No	No	No	No
A00475	Uni-Black	Uni	Black	Yes	No	No	No	No	No
A00481	Uni-Black	Uni	Black	Yes	No	No	No	No	No
000243	B2-Red/Black	BiElyt	Red	No	No	Yes	No	No	No
000L13	Uni-Black	Uni	Black	Yes	No	No	No	No	No
A00470	Uni-Yellow	Uni	Yellow	Yes	No	No	No	No	No
A00491	Uni-Black	Uni	Black	Yes	No	No	No	No	No
A00492	Uni-Black	Uni	Black	Yes	No	No	No	No	No
A00480	Uni-Black	Uni	Black	Yes	No	No	No	No	No
A00489	Uni-Black	Uni	Black	Yes	No	No	No	No	No
A00474	Uni-Black	Uni	Black	Yes	No	No	No	No	No
A00503	Uni-Yellow	Uni	Yellow	Yes	No	No	No	No	No
A00502	B2-Yellow/Black	BiElyt	Yellow	No	No	Yes	No	No	No
A00504	B2-Yellow/Black	BiElyt	Yellow	No	No	Yes	No	No	No
A00496	Uni-Yellow	Uni	Yellow	Yes	No	No	No	No	No
A00619	Fas-mid-Yell	Fasciate	Yellow	No	No	No	Yes	No	No
A00632	Fas-mid-Yell	Fasciate	Yellow	No	No	No	Yes	No	No
A00485	Uni-Black	Uni	Black	Yes	No	No	No	No	No
A00487	Stria	Striate	Yellow	No	No	No	No	Yes	No
ZL2018	Uni-Black	Uni	Black	Yes	No	No	No	No	No
A00488	B1-Black/Red	pronVSelyt	Red	No	Yes	No	No	No	No
A00490	B1-Black/Red	pronVSelyt	Red	No	Yes	No	No	No	No
A00482	B1-Black/Yellow	pronVSelyt	Yellow	No	Yes	No	No	No	No
A00555	B1-Black/Red	pronVSelyt	Red	No	Yes	No	No	No	No
A00484	Uni-Black	Uni	Black	Yes	No	No	No	No	No
A00471	Uni-Yellow	Uni	Yellow	Yes	No	No	No	No	No
A00478	Uni-Yellow	Uni	Yellow	Yes	No	No	No	No	No
A00479	Uni-Black	Uni	Black	Yes	No	No	No	No	No
RK0377	Fas-mid-Yell	Fasciate	Yellow	No	No	No	Yes	No	No
A00505	Uni-Black	Uni	Black	Yes	No	No	No	No	No
A00580	Uni-Black	Uni	Black	Yes	No	No	No	No	No
A00643	B2-Yellow/Black	BiElyt	Yellow	No	No	Yes	No	No	No
A00513	Stria	Striate	Yellow	No	No	No	No	Yes	No
A00639	Fas-mid-Yell	Fasciate	Yellow	No	No	No	Yes	No	No
A00507	Uni-Red	Uni	Red	Yes	No	No	No	No	No
A00512	Uni-Red	Uni	Red	Yes	No	No	No	No	No
A00509	Uni-Red	Uni	Red	Yes	No	No	No	No	No
A00511	Uni-Red	Uni	Red	Yes	No	No	No	No	No
A00646	Stria	Striate	Yellow	No	No	No	No	Yes	No
A00510	Fas-mid-Yell	Fasciate	Yellow	No	No	No	Yes	No	No
A00579	Fas-mid-Yell	Fasciate	Yellow	No	No	No	Yes	No	No
000542	Uni-Black	Uni	Black	Yes	No	No	No	No	No
000L22	Uni-Black	Uni	Black	Yes	No	No	No	No	No
VP0018	B2-Yellow/Black	BiElyt	Yellow	No	No	Yes	No	No	No
VP0019	B2-Yellow/Black	BiElyt	Yellow	No	No	Yes	No	No	No
VP2312	Uni-Yellow	Uni	Yellow	Yes	No	No	No	No	No
VP0003	B1-Black/Red	pronVSelyt	Red	No	Yes	No	No	No	No

000585	Uni-Black	Uni	Black	Yes	No	No	No	No	No
VP0006	B2-Yellow/Black	BiElyt	Yellow	No	No	Yes	No	No	No
000584	Uni-Black	Uni	Black	Yes	No	No	No	No	No
000L11	Uni-Black	Uni	Black	Yes	No	No	No	No	No
VP0016	Uni-Yellow	Uni	Yellow	Yes	No	No	No	No	No
VP0010	Uni-Yellow	Uni	Yellow	Yes	No	No	No	No	No
VP0012	Uni-Black	Uni	Black	Yes	No	No	No	No	No
VP0009	Uni-Black	Uni	Black	Yes	No	No	No	No	No
VP0013	Uni-Black	Uni	Black	Yes	No	No	No	No	No
VP0050	Uni-Yellow	Uni	Yellow	Yes	No	No	No	No	No
000M45	Uni-Yellow	Uni	Yellow	Yes	No	No	No	No	No
001043	Uni-Black	Uni	Black	Yes	No	No	No	No	No
000L01	Uni-Black	Uni	Black	Yes	No	No	No	No	No
000589	B2-Yellow/Black	BiElyt	Yellow	No	No	Yes	No	No	No
001383	Uni-Yellow	Uni	Yellow	Yes	No	No	No	No	No
VP0014	Uni-Yellow	Uni	Yellow	Yes	No	No	No	No	No
VP2316	Uni-Yellow	Uni	Yellow	Yes	No	No	No	No	No
001380	Uni-Yellow	Uni	Yellow	Yes	No	No	No	No	No
001381	Uni-Yellow	Uni	Yellow	Yes	No	No	No	No	No
001379	Uni-Yellow	Uni	Yellow	Yes	No	No	No	No	No
001387	B2-Yellow/Black	BiElyt	Yellow	No	No	Yes	No	No	No
VP0021	B2-Yellow/Black	BiElyt	Yellow	No	No	Yes	No	No	No
001388	B2-Yellow/Black	BiElyt	Yellow	No	No	Yes	No	No	No
001372	B2-Yellow/Black	BiElyt	Yellow	No	No	Yes	No	No	No
000586	B2-Yellow/Black	BiElyt	Yellow	No	No	Yes	No	No	No
001373	B2-Red/Black	BiElyt	Red	No	No	Yes	No	No	No
VP2308	B2-Yellow/Black	BiElyt	Yellow	No	No	Yes	No	No	No
001378	Uni-Yellow	Uni	Yellow	Yes	No	No	No	No	No
000587	B2-Yellow/Black	BiElyt	Yellow	No	No	Yes	No	No	No
001376	B2-Yellow/Black	BiElyt	Yellow	No	No	Yes	No	No	No
001385	Uni-Yellow	Uni	Yellow	Yes	No	No	No	No	No
001377	B2-Yellow/Black	BiElyt	Yellow	No	No	Yes	No	No	No
A00576	Uni-Black	Uni	Black	Yes	No	No	No	No	No
A00546	Uni-Black	Uni	Black	Yes	No	No	No	No	No
000L12	Uni-Black	Uni	Black	Yes	No	No	No	No	No
A00625	Uni-Black	Uni	Black	Yes	No	No	No	No	No
001001	B2-Yellow/Black	BiElyt	Yellow	No	No	Yes	No	No	No
A00596	B2-Yellow/Black	BiElyt	Yellow	No	No	Yes	No	No	No
A00599	Uni-Black	Uni	Black	Yes	No	No	No	No	No
A00600	Uni-Black	Uni	Black	Yes	No	No	No	No	No
001009	B2-Yellow/Black	BiElyt	Yellow	No	No	Yes	No	No	No
001017	B2-Yellow/Black	BiElyt	Yellow	No	No	Yes	No	No	No
A00542	B2-Red/Black	BiElyt	Red	No	No	Yes	No	No	No
A00626	B1-Black/Red	pronVSelyt	Red	No	Yes	No	No	No	No
A00572	B1-Black/Red	pronVSelyt	Red	No	Yes	No	No	No	No
A00545	B2-Red/Black	BiElyt	Red	No	No	Yes	No	No	No
000802	Uni-Red	Uni	Red	Yes	No	No	No	No	No
A00524	Uni-Red	Uni	Red	Yes	No	No	No	No	No
000805	Uni-Red	Uni	Red	Yes	No	No	No	No	No
A00530	Uni-Black	Uni	Black	Yes	No	No	No	No	No

A00532	Uni-Yellow	Uni	Yellow	Yes	No	No	No	No	No
A00533	Uni-Orange	Uni	Orange	Yes	No	No	No	No	No
A00541	B2-Yellow/Black	BiElyt	Yellow	No	No	Yes	No	No	No
000801	B2-Yellow/Black	BiElyt	Yellow	No	No	Yes	No	No	No
A00577	Uni-Black	Uni	Black	Yes	No	No	No	No	No
001002	B2-Yellow/Black	BiElyt	Yellow	No	No	Yes	No	No	No
001012	Uni-Black	Uni	Black	Yes	No	No	No	No	No
A00582	Uni-Black	Uni	Black	Yes	No	No	No	No	No
A00584	Uni-Black	Uni	Black	Yes	No	No	No	No	No
A00583	Uni-Black	Uni	Black	Yes	No	No	No	No	No
A00581	Uni-Black	Uni	Black	Yes	No	No	No	No	No
A00590	Uni-Black	Uni	Black	Yes	No	No	No	No	No
A00588	Uni-Black	Uni	Black	Yes	No	No	No	No	No
A00589	B1-Black/Red	pronVSelyt	Red	No	Yes	No	No	No	No
A00586	B2-Yellow/Black	BiElyt	Yellow	No	No	Yes	No	No	No
001008	Uni-Black	Uni	Black	Yes	No	No	No	No	No
TH0080	Uni-Black	Uni	Black	Yes	No	No	No	No	No
TH0007	Uni-Yellow	Uni	Yellow	Yes	No	No	No	No	No
TH0043	Uni-Yellow	Uni	Yellow	Yes	No	No	No	No	No
TH0079	Uni-Red	Uni	Red	Yes	No	No	No	No	No
TH0068	Uni-Black	Uni	Black	Yes	No	No	No	No	No
TH0046	Uni-Yellow	Uni	Yellow	Yes	No	No	No	No	No
TH0078	Uni-Yellow	Uni	Yellow	Yes	No	No	No	No	No
TH0041	B2-Yellow/Black	BiElyt	Yellow	No	No	Yes	No	No	No
TH0093	B2-Yellow/Black	BiElyt	Yellow	No	No	Yes	No	No	No
TH0006	B2-Yellow/Black	BiElyt	Yellow	No	No	Yes	No	No	No
TH0101	B2-Yellow/Black	BiElyt	Yellow	No	No	Yes	No	No	No
TH0131	B1-Black/Red	pronVSelyt	Red	No	Yes	No	No	No	No
TH0095	Uni-Red	Uni	Red	Yes	No	No	No	No	No
TH0190	B2-Red/Black	BiElyt	Red	No	No	Yes	No	No	No
A00060	Uni-Black	Uni	Black	Yes	No	No	No	No	No
TH0081	Uni-Black	Uni	Black	Yes	No	No	No	No	No
TH0020	B2-Yellow/Black	BiElyt	Yellow	No	No	Yes	No	No	No
TH0023	B2-Yellow/Black	BiElyt	Yellow	No	No	Yes	No	No	No
TH0160	Uni-Black	Uni	Black	Yes	No	No	No	No	No
000244	B2-Yellow/Black	BiElyt	Yellow	No	No	Yes	No	No	No
TH0152	Uni-Black	Uni	Black	Yes	No	No	No	No	No
TH0039	B2-Yellow/Black	BiElyt	Yellow	No	No	Yes	No	No	No
A00003	B2-Yellow/Black	BiElyt	Yellow	No	No	Yes	No	No	No
TH0059	Stria	Striate	Yellow	No	No	No	No	Yes	No
TH0054	B2-Red/Black	BiElyt	Red	No	No	Yes	No	No	No
TH0036	B2-Red/Black	BiElyt	Red	No	No	Yes	No	No	No
TH0076	B2-Yellow/Black	BiElyt	Yellow	No	No	Yes	No	No	No
TH0125	Uni-Black	Uni	Black	Yes	No	No	No	No	No
TH0138	Uni-Black	Uni	Black	Yes	No	No	No	No	No
TH0127	B2-Yellow/Black	BiElyt	Yellow	No	No	Yes	No	No	No
A00013	B2-Yellow/Black	BiElyt	Yellow	No	No	Yes	No	No	No
MD0099	B1-Red/Metalic	pronVSelyt	Metalic	No	Yes	No	No	No	No
MD0107	Uni-Black	Uni	Black	Yes	No	No	No	No	No
MD0101	Uni-Black	Uni	Black	Yes	No	No	No	No	No

MD0106	Fas-mid-Yell	Fasciate	Yellow	No	No	No	Yes	No	No
MD0033	Uni-Black	Uni	Black	Yes	No	No	No	No	No
MD0030	Uni-Black	Uni	Black	Yes	No	No	No	No	No
A00070	Uni-Black	Uni	Black	Yes	No	No	No	No	No
MD0065	Fas-mid-Yell	Fasciate	Yellow	No	No	No	Yes	No	No
A00067	Uni-Black	Uni	Black	Yes	No	No	No	No	No
A00077	Uni-Black	Uni	Black	Yes	No	No	No	No	No
MD0069	Fas-mid-Yell	Fasciate	Yellow	No	No	No	Yes	No	No
MD0034	Uni-Black	Uni	Black	Yes	No	No	No	No	No
MD0044	Fas-mid-Yell	Fasciate	Yellow	No	No	No	Yes	No	No
MD0071	Fas-mid-Yell	Fasciate	Yellow	No	No	No	Yes	No	No
A00128	B2-Yellow/Black	BiElyt	Yellow	No	No	Yes	No	No	No
A00116	B2-Yellow/Black	BiElyt	Yellow	No	No	Yes	No	No	No
A00160	B2-Yellow/Black	BiElyt	Yellow	No	No	Yes	No	No	No
A00033	B2-Red/Black	BiElyt	Red	No	No	Yes	No	No	No
A00038	B2-Yellow/Black	BiElyt	Yellow	No	No	Yes	No	No	No
A00046	B1-Black/Red	pronVSelyt	Red	No	Yes	No	No	No	No
A00132	Uni-Yellow	Uni	Yellow	Yes	No	No	No	No	No
A00148	Uni-Black	Uni	Black	Yes	No	No	No	No	No
A00040	B1-Black/Red	pronVSelyt	Red	No	Yes	No	No	No	No
000372	B1-Black/Red	pronVSelyt	Red	No	Yes	No	No	No	No
A00044	B1-Black/Red	pronVSelyt	Red	No	Yes	No	No	No	No
A00041	B1-Black/Red	pronVSelyt	Red	No	Yes	No	No	No	No
A00042	B1-Black/Red	pronVSelyt	Red	No	Yes	No	No	No	No
A00045	B1-Black/Red	pronVSelyt	Red	No	Yes	No	No	No	No
000378	B2-Red/Black	BiElyt	Red	No	No	Yes	No	No	No
000374	B1-Black/Red	pronVSelyt	Red	No	Yes	No	No	No	No
A00034	B1-Black/Red	pronVSelyt	Red	No	Yes	No	No	No	No
A00037	B1-Black/Red	pronVSelyt	Red	No	Yes	No	No	No	No
000366	Uni-Yellow	Uni	Yellow	Yes	No	No	No	No	No
000010	B1-Red/Metalic	pronVSelyt	Metalic	No	Yes	No	No	No	No
000011	B1-Red/Metalic	pronVSelyt	Metalic	No	Yes	No	No	No	No
000017	B1-Red/Metalic	pronVSelyt	Metalic	No	Yes	No	No	No	No
A00049	B2-Red/Black	BiElyt	Red	No	No	Yes	No	No	No
000009	B2-Red/Black	BiElyt	Red	No	No	Yes	No	No	No
A00032	B1-Black/Yellow	pronVSelyt	Yellow	No	Yes	No	No	No	No
000375	B2-Yellow/Black	BiElyt	Yellow	No	No	Yes	No	No	No
000373	B2-Yellow/Black	BiElyt	Yellow	No	No	Yes	No	No	No
MD0098	Uni-Yellow	Uni	Yellow	Yes	No	No	No	No	No
A00069	B2-Yellow/Black	BiElyt	Yellow	No	No	Yes	No	No	No
000L23	Uni-Black	Uni	Black	Yes	No	No	No	No	No
000412	B2-Yellow/Black	BiElyt	Yellow	No	No	Yes	No	No	No
A00039	B1-Black/Red	pronVSelyt	Red	No	Yes	No	No	No	No
A00061	Uni-Yellow	Uni	Yellow	Yes	No	No	No	No	No
A00059	B1-Black/Yellow	pronVSelyt	Yellow	No	Yes	No	No	No	No
000419	B2-Yellow/Black	BiElyt	Yellow	No	No	Yes	No	No	No
ZL2002	B2-Yellow/Black	BiElyt	Yellow	No	No	Yes	No	No	No
000396	B2-Yellow/Black	BiElyt	Yellow	No	No	Yes	No	No	No
A00052	Uni-Yellow	Uni	Yellow	Yes	No	No	No	No	No
MD0157	Fas-mid-Yell	Fasciate	Yellow	No	No	No	Yes	No	No

MD0126	Uni-Black	Uni	Black	Yes	No	No	No	No	No
MD0111	Uni-Black	Uni	Black	Yes	No	No	No	No	No
MD0127	Uni-Black	Uni	Black	Yes	No	No	No	No	No
MD0118	Uni-Black	Uni	Black	Yes	No	No	No	No	No
MD0133	Uni-Black	Uni	Black	Yes	No	No	No	No	No
MD0136	Uni-Yellow	Uni	Yellow	Yes	No	No	No	No	No
MD0137	Uni-Yellow	Uni	Yellow	Yes	No	No	No	No	No
MD0143	Fas-mid-Yell	Fasciate	Yellow	No	No	No	Yes	No	No
MD0121	Fas-mid-Yell	Fasciate	Yellow	No	No	No	Yes	No	No
MD0145	Fas-mid-Yell	Fasciate	Yellow	No	No	No	Yes	No	No
MD0132	Uni-Black	Uni	Black	Yes	No	No	No	No	No
MD0130	Uni-Black	Uni	Black	Yes	No	No	No	No	No
MD0119	Uni-Black	Uni	Black	Yes	No	No	No	No	No
MD0134	Uni-Black	Uni	Black	Yes	No	No	No	No	No
MD0135	Uni-Black	Uni	Black	Yes	No	No	No	No	No
A00185	Uni-Black	Uni	Black	Yes	No	No	No	No	No
A00076	Uni-Yellow	Uni	Yellow	Yes	No	No	No	No	No
000071	Uni-Black	Uni	Black	Yes	No	No	No	No	No
000184	Uni-Black	Uni	Black	Yes	No	No	No	No	No
VK0075	Uni-Black	Uni	Black	Yes	No	No	No	No	No
VK0249	Uni-Black	Uni	Black	Yes	No	No	No	No	No
000434	Uni-Yellow	Uni	Yellow	Yes	No	No	No	No	No
000121	Uni-Yellow	Uni	Yellow	Yes	No	No	No	No	No
000105	B2-Red/Black	BiElyt	Red	No	No	Yes	No	No	No
000248	B2-Yellow/Black	BiElyt	Yellow	No	No	Yes	No	No	No
000125	Uni-Yellow	Uni	Yellow	Yes	No	No	No	No	No
000026	B2-Yellow/Black	BiElyt	Yellow	No	No	Yes	No	No	No
A00065	Uni-Yellow	Uni	Yellow	Yes	No	No	No	No	No
A00090	B2-Yellow/Black	BiElyt	Yellow	No	No	Yes	No	No	No
A00080	B2-Yellow/Black	BiElyt	Yellow	No	No	Yes	No	No	No
A00099	B2-Yellow/Black	BiElyt	Yellow	No	No	Yes	No	No	No
000335	Uni-Black	Uni	Black	Yes	No	No	No	No	No
000205	Uni-Black	Uni	Black	Yes	No	No	No	No	No
000069	Reti	Non-Cat	Red	No	No	No	No	No	Yes
A00058	B2-Red/Black	BiElyt	Red	No	No	Yes	No	No	No
A00656	Uni-Yellow	Uni	Yellow	Yes	No	No	No	No	No
A00079	B2-Yellow/Black	BiElyt	Yellow	No	No	Yes	No	No	No
A00092	B2-Yellow/Black	BiElyt	Yellow	No	No	Yes	No	No	No
A00094	B2-Yellow/Black	BiElyt	Yellow	No	No	Yes	No	No	No
A00096	B2-Yellow/Black	BiElyt	Yellow	No	No	Yes	No	No	No
A00091	B2-Yellow/Black	BiElyt	Yellow	No	No	Yes	No	No	No
A00098	B2-Yellow/Black	BiElyt	Yellow	No	No	Yes	No	No	No
A00105	B2-Yellow/Black	BiElyt	Yellow	No	No	Yes	No	No	No
A00082	B2-Yellow/Black	BiElyt	Yellow	No	No	Yes	No	No	No
A00107	B2-Yellow/Black	BiElyt	Yellow	No	No	Yes	No	No	No
000195	B2-Yellow/Black	BiElyt	Yellow	No	No	Yes	No	No	No
A00078	B2-Yellow/Black	BiElyt	Yellow	No	No	Yes	No	No	No
A00106	B2-Yellow/Black	BiElyt	Yellow	No	No	Yes	No	No	No
A00088	B2-Yellow/Black	BiElyt	Yellow	No	No	Yes	No	No	No
A00102	B2-Yellow/Black	BiElyt	Yellow	No	No	Yes	No	No	No

A00089	B2-Yellow/Black	BiElyt	Yellow	No	No	Yes	No	No	No
A00085	B2-Yellow/Black	BiElyt	Yellow	No	No	Yes	No	No	No
A00111	B2-Yellow/Black	BiElyt	Yellow	No	No	Yes	No	No	No
A00110	B2-Yellow/Black	BiElyt	Yellow	No	No	Yes	No	No	No
A00112	B2-Yellow/Black	BiElyt	Yellow	No	No	Yes	No	No	No
000132	B2-Red/Black	BiElyt	Red	No	No	Yes	No	No	No
A00022	B2-Red/Black	BiElyt	Red	No	No	Yes	No	No	No
A00023	B2-Red/Black	BiElyt	Red	No	No	Yes	No	No	No
A00024	Uni-Black	Uni	Black	Yes	No	No	No	No	No
A00027	Uni-Black	Uni	Black	Yes	No	No	No	No	No
A00074	Uni-Yellow	Uni	Yellow	Yes	No	No	No	No	No
000379	B2-Yellow/Black	BiElyt	Yellow	No	No	Yes	No	No	No
000120	Uni-Red	Uni	Red	Yes	No	No	No	No	No
000152	Uni-Red	Uni	Red	Yes	No	No	No	No	No
000154	Uni-Yellow	Uni	Yellow	Yes	No	No	No	No	No
000153	Uni-Red	Uni	Red	Yes	No	No	No	No	No
MD0036	Uni-Black	Uni	Black	Yes	No	No	No	No	No
000224	B2-Red/Black	BiElyt	Red	No	No	Yes	No	No	No
000274	Uni-Yellow	Uni	Yellow	Yes	No	No	No	No	No
A00018	Uni-Yellow	Uni	Yellow	Yes	No	No	No	No	No
000402	Uni-Yellow	Uni	Yellow	Yes	No	No	No	No	No
000220	Uni-Black	Uni	Black	Yes	No	No	No	No	No
000070	Uni-Black	Uni	Black	Yes	No	No	No	No	No
000030	B1-Black/Yellow	pronVSelyt	Yellow	No	Yes	No	No	No	No
000068	B1-Black/Yellow	pronVSelyt	Yellow	No	Yes	No	No	No	No
000066	Uni-Black	Uni	Black	Yes	No	No	No	No	No
000L06	B2-Yellow/Black	BiElyt	Yellow	No	No	Yes	No	No	No
000043	B2-Red/Black	BiElyt	Red	No	No	Yes	No	No	No
000403	B2-Yellow/Black	BiElyt	Yellow	No	No	Yes	No	No	No
000052	B2-Red/Black	BiElyt	Red	No	No	Yes	No	No	No
000056	B2-Red/Black	BiElyt	Red	No	No	Yes	No	No	No
000122	B2-Red/Black	BiElyt	Red	No	No	Yes	No	No	No
000290	Uni-Yellow	Uni	Yellow	Yes	No	No	No	No	No
ZL2009	B1-Black/Red	pronVSelyt	Red	No	Yes	No	No	No	No
A00021	Uni-Yellow	Uni	Yellow	Yes	No	No	No	No	No
000217	Uni-Black	Uni	Black	Yes	No	No	No	No	No
000219	Uni-Black	Uni	Black	Yes	No	No	No	No	No
000109	B2-Red/Black	BiElyt	Red	No	No	Yes	No	No	No
000047	B2-Red/Black	BiElyt	Red	No	No	Yes	No	No	No
000178	Uni-Yellow	Uni	Yellow	Yes	No	No	No	No	No
000123	Uni-Yellow	Uni	Yellow	Yes	No	No	No	No	No
000188	B2-Red/Black	BiElyt	Red	No	No	Yes	No	No	No
000189	B2-Red/Black	BiElyt	Red	No	No	Yes	No	No	No
000147	B2-Red/Black	BiElyt	Red	No	No	Yes	No	No	No
000174	B2-Red/Black	BiElyt	Red	No	No	Yes	No	No	No
000079	B2-Yellow/Black	BiElyt	Yellow	No	No	Yes	No	No	No
000064	Uni-Yellow	Uni	Yellow	Yes	No	No	No	No	No
000075	Uni-Red	Uni	Red	Yes	No	No	No	No	No
A00048	B2-Red/Black	BiElyt	Red	No	No	Yes	No	No	No
000050	Uni-Red	Uni	Red	Yes	No	No	No	No	No

000060	Uni-Black	Uni	Black	Yes	No	No	No	No	No
000048	B2-Red/Black	BiElyt	Red	No	No	Yes	No	No	No
000164	Uni-Red	Uni	Red	Yes	No	No	No	No	No
000044	B2-Red/Black	BiElyt	Red	No	No	Yes	No	No	No
000104	B2-Red/Black	BiElyt	Red	No	No	Yes	No	No	No
000084	Reti	Non-Cat	Red	No	No	No	No	No	Yes
000080	B2-Yellow/Black	BiElyt	Yellow	No	No	Yes	No	No	No
000081	B2-Yellow/Black	BiElyt	Yellow	No	No	Yes	No	No	No
000090	B2-Yellow/Black	BiElyt	Yellow	No	No	Yes	No	No	No
000246	B2-Red/Black	BiElyt	Red	No	No	Yes	No	No	No
000295	B2-Red/Black	BiElyt	Red	No	No	Yes	No	No	No
000040	B2-Red/Black	BiElyt	Red	No	No	Yes	No	No	No
000314	B2-Red/Black	BiElyt	Red	No	No	Yes	No	No	No
A00019	Uni-Yellow	Uni	Yellow	Yes	No	No	No	No	No
000346	Uni-Black	Uni	Black	Yes	No	No	No	No	No
000088	B2-Red/Black	BiElyt	Red	No	No	Yes	No	No	No
000206	B2-Red/Black	BiElyt	Red	No	No	Yes	No	No	No
A00062	B2-Yellow/Black	BiElyt	Yellow	No	No	Yes	No	No	No
000074	B2-Red/Black	BiElyt	Red	No	No	Yes	No	No	No
000395	B2-Yellow/Black	BiElyt	Yellow	No	No	Yes	No	No	No
000425	Uni-Yellow	Uni	Yellow	Yes	No	No	No	No	No
000411	B2-Yellow/Black	BiElyt	Yellow	No	No	Yes	No	No	No
000339	B2-Red/Black	BiElyt	Red	No	No	Yes	No	No	No
000342	Uni-Black	Uni	Black	Yes	No	No	No	No	No
000294	B2-Red/Black	BiElyt	Red	No	No	Yes	No	No	No
000355	B2-Red/Black	BiElyt	Red	No	No	Yes	No	No	No
000262	Uni-Black	Uni	Black	Yes	No	No	No	No	No
000297	Uni-Black	Uni	Black	Yes	No	No	No	No	No

Table S5. Internal and external contrasts in nature.

Figure 1	G	H							
Internal contrast (a)	75.8	62.4							
External constrast (bright/backgr.)	n.a.	n.a.							
External constrast (dark/backgr.)	n.a.	n.a.							

Figure 2	A	B	C	D	G	H	I	J	K
Internal contrast	n.a.	51.7	83.8	n.a.	27.7	n.a.	n.a.	46.7	70.2
External constrast (bright/backgr.)	59.3	68.7	82.4	48.8	48.6	n.a.	79.3	43.7	61.9
External constrast (dark/backgr.)	n.a.	73.27	65.8	n.a.	52.5	41.6	n.a.	77.1	51.4
cont.	L	M	N	O	P	Q	R	S	T
Internal contrast	n.a.	n.a.	69.0	66.7	71.5	38.2	44.6	56.6	82.7
External constrast (bright/backgr.)	77.0	60.6	53.1	54.0	47.8	42.7	35.2	n.a.	91.9
External constrast (dark/backgr.)	n.a.	n.a.	40.9	47.8	71.4	41.6	48.0	n.a.	88.1

Figure 3	A	B	C	D	E	F	G	H	I
Internal contrast	64.0	53.1	31.7	79.7	63.0	55.1	58.5	47.7	58.6
External constrast (bright/backgr.)	48.4	51.3	86.3	40.9	48.2	22.9	31.9	36.3	49.1
External constrast (dark/backgr.)	52.2	58.1	90.7	48.5	57.5	39.3	44.6	24.7	53.2
cont.	J	K	L	M	N	O	P	Q	R
Internal contrast	n.a.	43.5	39.8	n.a.	75.3	57.7	n.a.	92.8	77.0
External constrast (bright/backgr.)	n.a.	55.5	60.8	38.6	44.9	26.7	63.9	61.1	68.8
External constrast(dark/backgr.)	n.a.	65.1	63.7	n.a.	43.2	34.9	n.a.	64.0	43.4
cont.	S	T	U	V	W	X			
Internal contrast	n.a.	n.a.	n.a.	68.1	52.1	50.3			
External constrast (bright/backgr.)	59.1	45.6	43.3	55.4	44.0	64.5			
External constrast (dark/backgr.)	n.a.	n.a.	n.a.	51.9	49.8	52.9			

Summary	n	AVERAGE	MIN	MAX	STDEV
Internal contrast(a)	32	61.6	27.7	92.8	15.8
External constrast (bright/backgr.)	29	51.0	22.9	91.9	15.2
External constrast (dark/backgr.)	29	54.2	24.7	88.1	13.9
external contrast (uniform bright)	11	57.6	38.6	79.3	13.7
external contrast (uniform black)	2	49.1	41.6	56.6	n.a.

Legend
(a) contrast between bright and dark upper body parts

Table S6. Internal and external contrast of typical representatives of net-winged beetle patterns. DeltaE values were counted as Euclidian distances between colours in the L*a*b CIE 76 colour space.

Uniform coloured dorsal part of the body

Pattern	Voucher #	Distrib.	Taxon	External contrast upper	bottom	average
black	MK0663	Japan	*Xylobanus* sp.	26.4	37.8	32.1
black	MK0687	Japan	*Plateros* sp.	25.2	36.8	31.0
black	MK1002	Malaya	*Cautires* sp.	29.7	38.0	33.8
			average	**27.1**	**37.5**	**32.3**
yellow	A03995	New Guin.	*Microtrichalus* sp.	62.2	49.7	55.9
yellow	MK0744	Okinawa	*Plateros* sp.	57.2	46.1	51.7
yellow	MK1019	Arunachalpr.	*Xylobanus* sp.	52.5	41.3	46.9
yellow	MK0352	Yunnan	*Plateros* sp.	54.9	43.9	49.4
yellow	n.a.	Costa Rica	*Calopteron* sp.	33.6	30.7	32.1
orang/yell.	MK0015	Yunnan	*Xylometanoeus* sp.	58.4	50.2	54.3
			average	**53.1**	**43.6**	**48.4**
red	MK0043	Yunnan	*Conderis signicollis*	68.0	67.5	67.8
red	n.a.	Sichuan	*Macrolycus* sp.	41.4	42.1	41.8
red	n.a.	Sichuan	*Pyropterus* sp.	49.1	50.5	49.8
			average	**52.9**	**53.4**	**53.1**
dark red	MK0331	Yunnan	*Parantis* sp.	34.4	39.5	37.0
dark red	n.a.	Sichuan	*Parantis* sp.	32.9	40.8	36.8
dark red	n.a.	Sichuan	*Lycostomus* sp.	31.0	39.4	35.2
			average	**32.8**	**39.9**	**36.3**
blue	A04070	New Guin.	*Diatrichalus* sp.	39.3	49.5	44.4
blue	A04090	New Guin.	*Diatrichalus* sp.	37.9	48.1	43.0
blue	A03852	New Guin.	*Diatrichalus* sp.	23.0	31.6	27.3
			average	**33.4**	**43.0**	**38.2**

brown	A03781	New Guinea	Metriorrhynchini	38.9	30.0	34.5
green	A03868	New Guinea	*Porrostoma* sp.	26.9	14.9	20.9
cinamon	A04018	New Guinea	*Cladophorus* sp.	39.3	38.9	39.1

Bi-coloured dorsal part of the body

	Elytral					Contrast						
Pronotum	humeri	apex				internal	external					
							bright/background			dark/background		
Uniform colour of elytra							upper	bott.	aver.	upper	bott.	aver.
black	yellow	yellow	MR0007	Ethiopia	*Plateros* sp.	76.5	62.6	52.8	49.5	31.1	43.1	37.1
black	yellow	yellow	MR0008	Ethiopia	*Plateros* sp.	79.2	64.6	53.8	50.7	29.4	41.4	35.4
black	yellow	yellow	JB0137	New Guinea	Metriorrhynchini	58.8	44.4	30.7	32.2	27.8	38.9	33.4
					average	**71.5**	**57.2**	**45.8**	**44.1**	**29.4**	**41.1**	**35.3**
black	red	red	TV0050	Okinawa	*Cautires* sp.	42.6	29.6	41.7	35.7	40.5	39.5	40.0
black	red	red	MK0743	Okinawa	*Plateros* sp.	58.8	53.4	46.8	50.1	28.9	39.9	34.4
black	red	red	A03669	Spain	*Benibotarus alternatus*	73.9	30.0	42.3	36.2	71.1	66.8	69.0
black	red	red	A03671	Sardinia	*Lygistopt. anorachilus*	70.8	31.6	43.7	37.6	62.0	56.8	59.4
black	red	red	n.a.	Sechuan	*Lyp. nigrohumeralis*	27.0	39.4	43.7	41.6	28.7	40.7	34.7
					average (without *L. nigrohumeralis*)	**61.5**	**36.2**	**43.6**	**39.9**	**50.6**	**50.8**	**50.7**
yellow	black	black	A00727	New Guinea	*Cautiromimus* sp.	51.9	46.6	40.2	43.4	27.4	38.4	32.9
yellow	black	black	A02903	New Guinea	*Cautiromimus* sp.	54.7	52.2	45.9	49.0	26.0	35.8	30.9
yellow	black	black	A00566	South Africa	*Flagrax* sp.	52.2	50.9	43.2	47.1	26.9	34.8	30.9
					average	**52.9**	**49.9**	**43.1**	**46.5**	**26.8**	**36.3**	**31.6**
orange	metall	metall.	A01947	Sulawesi	*Metriorrh. thoracicus*	69.6	51.6	45.4	48.5	30.6	40.4	35.5
orange	metall.	metall.	A03792	New Guinea	Metriorrhynchini indet.	63.3	48.2	44.5	46.3	32.1	42.1	37.1
orange	metall.	metall.	n.a.	Sulawesi	*Calochromus toxopei*	61.4	42.8	41.5	42.1	34.0	42.9	38.5

yellow	metall.	metall.	A02540	Sulawesi	*Broxylus pfeifferi*	48.1	46.1	38.3	42.2	34.7	40.0	37.4
					average (without *Broxylus*)	**64.8**	**47.5**	**43.8**	**45.6**	**32.2**	**41.8**	**37.0**
orange	amet.	amet.	A04006	New Guinea	Metriorrhynchini indet.	46.6	30.1	23.3	26.7	36.8	31.9	34.4
orange	green	green	A04010	New Guinea	Metriorrhynchini indet.	23.5	35.0	21.0	28.0	36.2	31.3	33.8
orange	green	green	A04011	New Guinea	Metriorrhynchini indet.	30.4	38.1	32.1	35.1	38.7	25.1	31.9
					average	**33.5**	**34.4**	**25.5**	**29.9**	**37.2**	**29.4**	**33.3**
<u>Bi-colored elytra</u>												
yell/bl.	yellow	black	MK0477	Zambia	*Cautires* sp.	71.5	60.4	51.7	56.0	27.4	39.1	33.3
yell./bl.	yellow	black	MK0451	Kenya	*Cautires* sp.	56.3	56.6	45.4	51.0	23.8	30.8	27.3
black	yellow	black	A04086	New Guinea	*Flabellotrichalus* sp.	67.6	57.3	44.6	51.0	25.8	36.7	31.3
					average	**65.1**	**58.1**	**47.2**	**52.7**	**25.7**	**35.5**	**30.6**
black	red	black	A04068	New Guinea	*Cautiromimus* sp.	51.2	49.0	46.0	47.5	28.3	40.1	34.2
black	red	black	A04054	New Guinea	*Ditua* sp.	72.1	67.6	63.9	65.8	30.5	43.0	36.8
orange	orange	black	A03709	New Guinea	Metriorrhynchini indet.	52.8	52.6	49.2	50.9	28.3	40.1	34.2
					average	**58.7**	**56.4**	**53.0**	**54.7**	**29.0**	**41.1**	**35.1**
black	black	yellow	A04014	New Guinea	Metriorrhynchini indet.	64.9	53.8	39.5	46.6	26.6	37.5	32.1
black	black	orange	A03998	New Guinea	Metriorrhynchini indet.	77.7	64.3	54.2	59.3	28.4	40.8	34.6
black	black	orange	A04138	New Guinea	Metriorrhynchini indet.	78.9	63.0	52.6	57.8	29.7	42.3	36.0
					average	**73.8**	**60.4**	**48.8**	**54.6**	**28.2**	**40.2**	**34.2**
red	red	metallic	A00593	Cuba	*Thonalmus* sp.	70.1	55.6	53.4	54.5	44.9	54.1	49.5
red	red	metallic	A00592	Cuba	*Thonalmus* sp.	67.2	51.2	46.0	48.6	49.3	58.1	53.7
red	red	metallic	A00595	Dominicana	*Thonalmus* sp.	64.8	56.5	53.2	54.8	45.7	54.8	50.3
					average	**67.4**	**54.4**	**50.9**	**52.6**	**46.6**	**55.7**	**51.2**

Fasciate, slender elytra, black apex

black	fasc./mid. yell.	A01950	Sulawesi	*Sulabanus* sp.	60.1	51.6	40.5	46.1	28.0	37.8	32.9
orange	fasc./mid. yell.	A02047	New Guinea	Metriorrhynchini indet.	53.3	53.6	39.5	46.5	25.0	31.6	28.3
bl./oran.	fasc./mid. yell.	A02080	New Guinea	*Cladophorus* sp.	70.9	61.2	50.4	55.8	27.6	37.8	32.7
yell./bl.	fasc./mid. yell.	A02296	Bolivia	Calopterini indet.	52.0	53.7	41.0	47.4	25.6	31.2	28.4
				average	**59.1**	**55.0**	**42.9**	**49.0**	**26.6**	**34.6**	**30.6**

Fasciate, wide elytra, apex black, metallic shine

black	fasc./mid. yell.	A00663	Equador	*Calopteron* sp.	53.9	45.1	33.2	39.2	29.4	40.5	35.0
bl./oran.	fasc./mid. yell.	A00621	Panama	*Calopteron* sp.	72.0	56.1	45.6	50.8	31.3	43.5	37.4
black	fasc./mid. yell.	A02303	Bolivia	*Calopteron* sp.	74.5	62.5	52.2	57.4	32.0	42.8	37.4
black	fasc./mid. yell.	A02320	Peru	*Calopteron* sp.	56.4	47.2	34.1	40.7	28.7	38.6	33.7
				average	**64.2**	**52.7**	**41.3**	**47.0**	**30.4**	**41.4**	**35.9**

Fasciate, apex brightly coloured

black	fasc./mid. black	A03825	New Guinea	Metriorrhynchini indet.	75.2	59.2	50.6	54.9	31.7	44.8	38.3
black	fasc./mid. black	A04137	New Guinea	*Cladophorus* sp.	68.1	57.3	47.2	52.3	27.5	39.2	33.4
orange	fasc./mid. black	A02170	New Guinea	*Porrostoma* sp.	73.7	62.4	51.3	56.8	26.2	37.5	31.9
				average	**72.3**	**59.6**	**49.7**	**54.7**	**28.5**	**40.5**	**34.5**

Fasciate, humeri black, two bands

yell./bl.	fasc.-two bands	n.a.	Bolivia	Calopterini indet.	30.1	28.0	25.7	26.8	27.1	38.6	32.9

Striate, single stripe in elytron

yell./bl.	striate-black stripe	DK0048	Australia	*Synchonnus* sp.	57.3	55.0	43.4	49.2	25.8	34.1	30.0
yell./bl.	striate-black stripe	A00641	Equador	*Plateros* sp.	52.4	48.7	40.2	44.5	27.4	37.5	32.5
black	striate-black stripe	A00324	Australia	*Synchonnus* sp.	65.3	56.2	46.4	51.3	27.4	38.4	32.9
				average	**58.3**	**53.3**	**43.3**	**48.3**	**26.9**	**36.7**	**31.8**

Striate, multiple stripes in elytron

black	striate metall./yell.	A00628	Equador	*Idiopteron* sp.	50.3	40.5	29.5	35.0	28.4	38.5	33.5

<u>Punctate</u>												
Yell./bl.	punct.(2 patch.)	DK0158	Australia	*Synchonnus* sp.	52.9	59.0	47.3	53.1	25.8	30.6	28.2
Yell./bl.	punct.(2 patch.)	DK0018	Australia	*Synchonnus* sp.	50.6	51.3	42.1	46.7	24.8	32.9	28.9
red/bl.	punct.(3 patch.)	n.a.	Tibet	*Parantis trigutatus*	38.3	38.0	37.7	37.9	30.9	42.7	36.8
				average	**47.3**	**49.4**	**42.4**	**45.9**	**27.2**	**35.4**	**31.3**
<u>Tricolorate patterns</u>											
silver black red		A04028	New Guinea	*Cladophorus* sp.	60.4	53.9	48.0	51.0	27.8	39.7	33.8
orange grey black		A02754	New Guinea	*Cladophorus* sp.	23.0	26.4	25.2	25.8	27.4	37.5	32.5
<u>Additional patterns</u>											
black white yellow		A02081	New Guinea	Metriorrhynchini indet.	52.2	59.5	47.7	53.6	61.4	49.4	55.4
yell./bl. white yellow		A02075	New Guinea	Metriorrhynchini indet.	36.2	56.7	45.1	50.9	58.1	43.7	50.9
black black blue		A03819	New Guinea	Metriorrhynchini indet.	41.7	44.5	45.7	45.1	29.1	41.4	35.3
black green black		A04156	New Guinea	Metriorrhynchini indet.	46.5	43.0	29.3	36.2	24.2	31.9	28.1
black white black		A02761	New Guinea	Metriorrhynchini indet.	62.6	57.8	45.4	51.6	26.2	36.5	31.4
black white black		A02087	New Guinea	Metriorrhynchini indet.	53.4	55.3	43.6	49.4	24.8	32.5	28.7
black white black		A02172	New Guinea	Metriorrhynchini indet.	62.9	57.1	43.5	50.3	25.3	35.5	30.4
black pale brown black		A04154	New Guinea	Metriorrhynchini indet.	28.5	40.6	29.7	35.1	23.9	26.5	25.2

Table S7. The distribution of the colour patterns.

	Area@		Uniform				Bicolored: pronotum/elytra				Bicolored elytra: humeri/apex				Fasclate		Striate	Punctate	Tricolored	Reticulate	
Region	km^2 (1,000)	# pattern	black	yellow	red	metallic	black/yell.	black/red	bright/black	red/metall.	yell./black	red/black	black/red	black/yell.	Yell/black	Yell./metall.	bright/black	bright/black	all comb.	all comb.	addit.patterns
ORIENTAL																					
Indoburma	1938	6	+	+	+			+	+			+									
S. India & Ceylon	900	6		+	+						+	+			+			+			
Sundaa, Philippines	1777	10	+	+	+			+	+		+	+	+				+			+	
AFROTROPICAL																					
Arfica continental	13951	6		+			+				+			+	+		+				
Madagascar	587	2	+						+												
PALEARCTIC																					
China	6024	7	+	+	+		+	+	+								+				
Himalayas	400	6	+		+		+		+								+	+			
Japan	378	5	+	+	+			+	+												
Siberia,Korea	1525	3	+		+			+													
Europe continental	9160	2			+			+													
Turkey	780	1			+																
Northern Africa	384	3			+						+	+									
Azerbaijan, Iran	45	2		+	+																
NEOTROPICAL																					
Mesoamerica cont.	1708	6		+	+						+	+			+			+			
Caribbean	211	2									+										+ *
Northern Andes	4272	14	+	+		+			+		+	+	+	+	+	+	+	+		+	
Bolivia, Argentina	2889	3	+								+			+							
Brazil etc.	9901	3							+		+			+							
NEARCTIC																					
USA SW	600	5	+	+			+				+	+									
USA Eastr	2075	4			+				+		+			+							
USA NW	1485	1			+																
Canada	660	3						+			+			+							
AUSTRALIAN																					
Sulawesi	175	5	+	+						+	+				+						
Lesser Sundas	83	4		+					+		+			+							
Moluccas	74	3		+					+	+	+										
New Guinea	786	22	+	+	+	+	+		+		+	+	+	+	+			+	+		+**
Australia Qeensland	1111	7			+			+	+		+		+	+	+						
Australia South	850	2						+					+								

* *Thonalmus* spp., (orange/blue)

** (1) scutellar spot; (2) black white; (3) yellow white; (4)cinnamon; (5) black humeri; (6) red gree; (7) red blue; (8) gree; (9) silver/yellow

@ the estimation of an area where net-winged beetles occur

Table S8. The summary of the colour patterns recorded in lycids tribes.

Subfamily	Tribe	# pattern	Uniform: black	Uniform: yellow	Uniform: red	Uniform: metallic	Bicolored: pronotum/elytra: black/yell.	Bicolored: pronotum/elytra: black/red	Bicolored: pronotum/elytra: bright/black	Bicolored: pronotum/elytra: red/metall.	Bicolored: humeri/apex: yell./black	Bicolored: humeri/apex: red/black	Bicolored: humeri/apex: black/red	Bicolored: humeri/apex: black/yell.	Fasciate: Yell./black	Fasciate: Yell./metall.	Striate: bright/black	Punctate: bright/black	Tricolored: all comb.	Reticulate: all comb.
Dexorinae	Dexorini (n)	2																		
	Mimolibnetini (n)	2																		
Calochrominae	Calochromini	14																		
Erotinae	Erotini	5																		
	Slipinskiini	3																		
	Dictyopterini	4																		
	Taphini	2																		
Ateliinae	Ateliini (n)	3																		
	Macrolycini	2																		
	Lyponiini	2																		
Lyropaeinae	Alyculini (n)	1																		
	Lyropaeini (n)	6																		
	Platerodrilini (n)	4																		
Lycinae	Conderini	2																		
	Eurrhacini	3																		
	Thonalmini	1																		
	Platerodini	10																		
	Leptolycini (n)	1																		
	Calopterini (n-part)	10																		
	Lycini	7																		
Metriorrhynchinae	Dihammatini	3																		
	Lycoprogenthini	3																		
	Libnetini	4																		
	Dilophotini	7																		
	Metriorrhynchini	15																		
Number of tribes with the pattern			17	16	10	4	5	11	2	2	14	8	1	1	7	1	11	2	1	3
Number of species with the pattern			>500	>500	>100	<50	<50	>100	<50	<10	>500	>300	>50	>50	>300	<10	<50	<50	<10	<10
	Batesian co-mimics		+	+	+	+	+	+	-	+	+	+	-	-	+	-	+	-	-	-

1 Oriental region

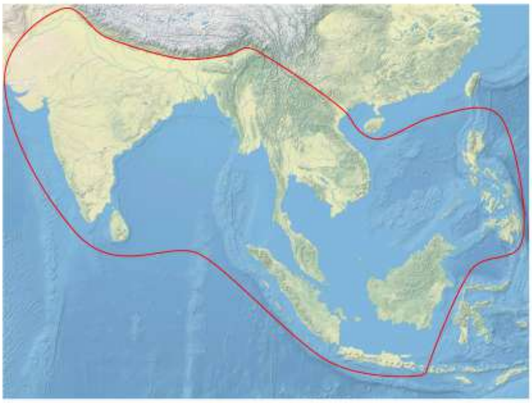

Indo-Burma

2

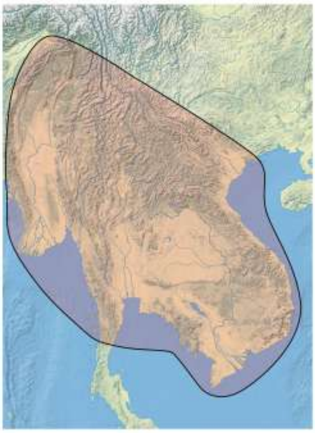

Phylogenetic structure of the fauna

Subfamily	Tribe /Subtribe	# spp. (woldwide)	# spp. (region)
Dexorinae	Dexorini	15 spp.	
	Mimolibnetini	5 spp.	
Erotinae	Erotini	54 spp.	5 spp.
	Dictyopterini	73 spp.	8 spp.
	Taphini	31 spp.	1 sp.
	Slipinskiini	46 spp.	
Calochrominae	Calochromini	289 spp.	20 spp.
Ateliinae	Ateliini	45 spp.	3 spp.
	Lyponiini	45 spp.	5 spp.
	Macrolycini	69 spp.	2 spp.
Lyropaeinae	Lyropaeini	43 spp.	2 spp.
	Alyculini	5 spp.	
	Antennolycini	3 spp.	
	Platerodrilini	49 spp.	2 spp.
Lycinae	Conderini	42 spp.	5 spp.
	Eurrhacini	102 spp.	
	Thonalmini	11 spp.	
	Leptolycini	12 spp.	
	Platerodini	861 spp.	~50 spp.
	Calopterini	367 spp.	
	Lycini	413 spp.	~10 spp.
Metriorrhynchinae	Dihammatini	44 spp.	5 spp.
	Lycoprogenthini	7 spp.	3 spp.
	Libnetini	112 spp.	~20 spp.
	Dilophotini	81 spp.	~10 spp.
	Metriorrhynchini	1410 spp.	
	Metanoeina	40 spp.	5 spp.
	Cautirina	750 spp.	~25 spp.
	Metriorrhynchina	620 spp.	10 spp.
Total		4234 spp.	~180 spp.

The presence of net-winged beetle aposematic patterns in the region

Group	Colour type	Characteristics	+/-	Figures
Uniform coloration	black	pronotum an elytra uniformly black to dark brown	✓	17-24
	yellow	pronotum and elytra yellow to light brown	✓	26-38
	orange and red	pronotum and elytra brightly orange or cinnamon red	✓	7-15, 27-32, 43-45
	metallic (blue, green)	pronotum and elytra metallic, all shades of colours		
Bi-colored pronotum/ elytra	black/yellow	pronotum black (at most with bright margins), elytra yellow		
	black/red	pronotum black (at most with bright margins), elytra red	✓	4-6
	bright/black	pronotum brightly colored, elytra uniformly black	✓	41-42
	red/merallic	pronotum brightly red, elytra metallic blue		
Bi-colored elytra	yellow/black	elytra bi-colored; humeral part yellow, apical part dark coloured		
	red(orange)/black	elytra bi-colored; humeral part orange/red, apical part dark	✓	40
	black/bright	elytra bi-colored; humeral part black, apex yellow/orange/red	✓	46
Fasciate elytra	yellow/black	humeri and apex of elytra black, middle of elytron yellow		
	bright/black	humeri and apex of elytra brigt, middle of elytron black		
	yellow/metallic	most elytra black with blue metallic shine, middle yellow		
Striate elytra	bright/black	elytra brown/brightly coloured, suture or middle of elytron black		
Punctate el.	bright/black	elytra brown/brightly coloured, black patch in each elytron		
Tri-colored el.	all combinations of colors	elytron with three differently coloured parts		
Reticulate	bright/black	backgroud colour of elytra dark, costae large, brightly coloured		
Non-categorized aposematic patterns		see listed examples for further information		

Figures S1–S69. The overview of the lycid fauna, coloration, and co-mimics: Oriental region, Indo-Burma.

All graphics and text produced by the authors as they are listed under the title of this article (CC-BY open acces license).

Indo-Burma

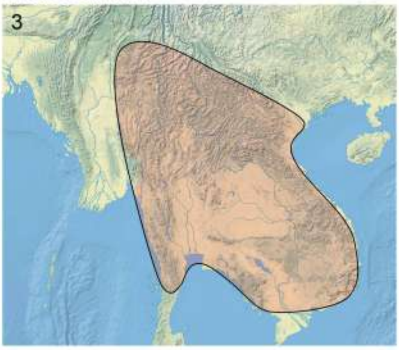

Pattern: bicoloured; black pronotum, red elytra

Body size: 5-13 mm

Distribution: northern Indo-Burma

Non-lycid co-mimics:

Remark: the common pattern in the eastern part of the Palearctic region

Ateliinae: Lyponiini | Lycinae: Platerodini | Dictyopterinae: Taphini

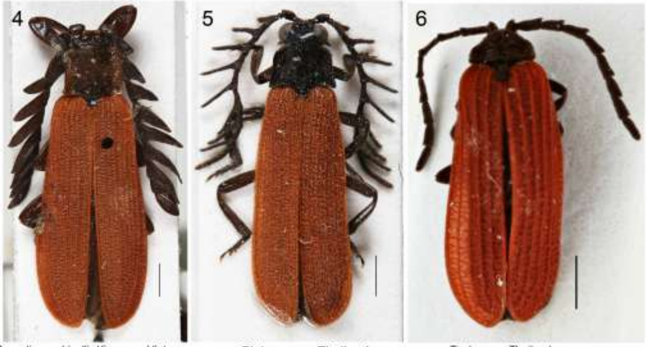

Ponyalis quadricollis Kiesenw., Vietnam | *Plateros* sp., Thailand | *Taphes* sp., Thailand

Pattern: unirform red

Body size: 5-10 mm

Distribution: northern Indo-Burma

Non-lycid co-mimics:

Remark: the common pattern in the eastern part of the Palearctic region

Dictyopterinae: Taphini | Lycinae: Platerodini | Lycinae: Conderini | Metriorrhynchinae: Dilophotini | Lycinae: Platerodini

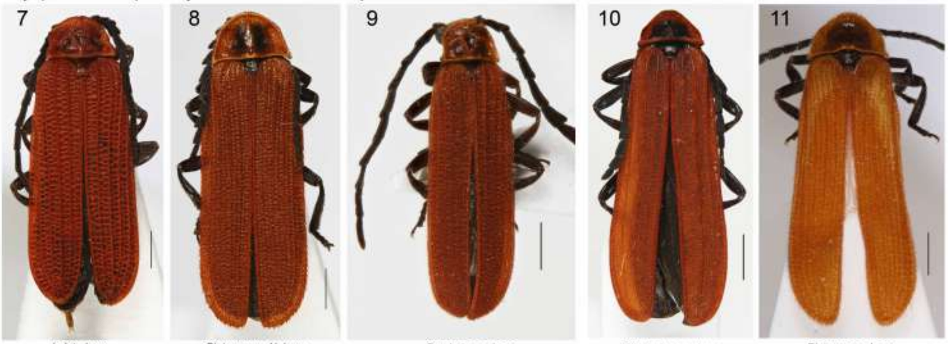

indet., Laos | *Plateros* sp., N. Laos | *Conderis* sp., Laos | *Dilophotes* sp., Laos | *Plateros* sp., Laos

Pattern: uniform cinnamom brown

Body size: 5-9 mm

Distribution: northern Indo-Burma

Non-lycid co-mimics:

Remark: the common pattern in the eastern part of the Palearctic region

Lycinae: Platerodini | Erotinae: Erotini

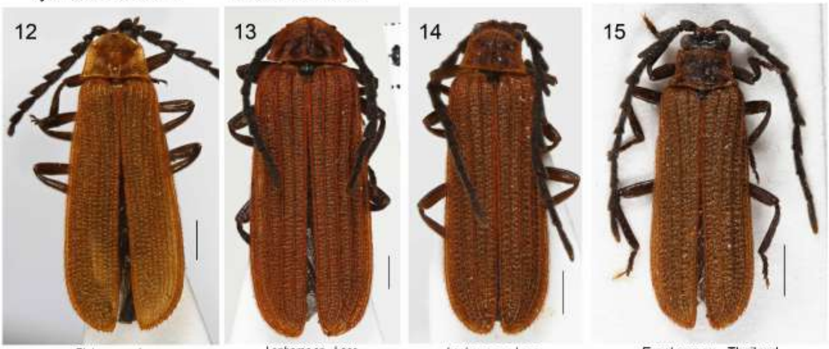

Plateros sp., Laos | *Lopheros* sp., Laos | *Lopheros* sp., Laos | *Eropterus* sp., Thailand

Indo-Burma

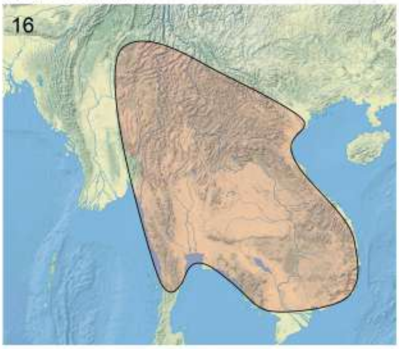

Pattern: unirform black to dark red

Body size: 4-8 mm

Distribution: Indo-Burma

Non-lycid co-mimics: Cantharidae

Remark: widespread pattern in Indo-Burma and all neighbouring regions

Lycinae: Conderini | Metriorrhynchinae: Dilophotini

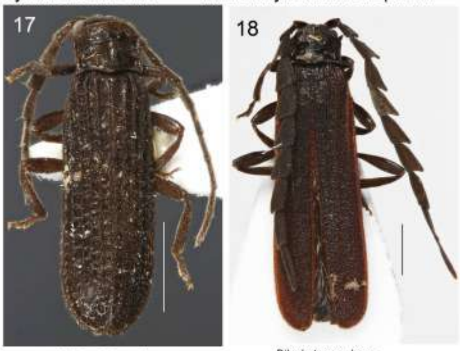

Xylobanellus sp., Laos | *Dilophotes* sp., Laos

All graphics and text produced by the authors as they are listed under the title of this article (CC-BY open acces license).

Metriorrhynchinae: Dilophotini | Metriorrhynchinae: Dihammatini | Dictyopterini

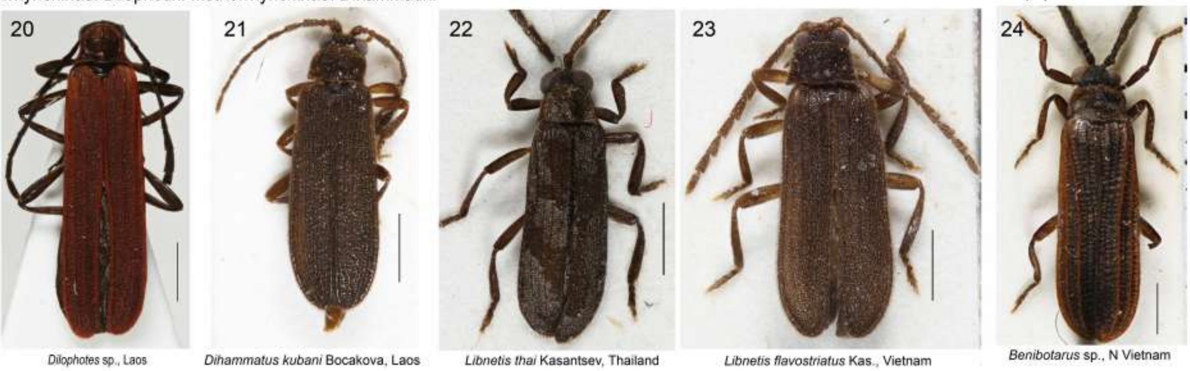

Dilophotes sp., Laos | *Dihammatus kubani* Bocakova, Laos | *Libnetis thai* Kasantsev, Thailand | *Libnetis flavostriatus* Kas., Vietnam | *Benibotarus* sp., N Vietnam

Indo-Burma

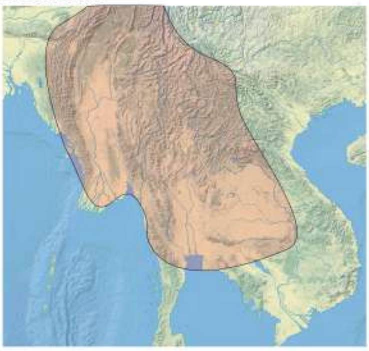

M.:M.:Metriorrhynchina

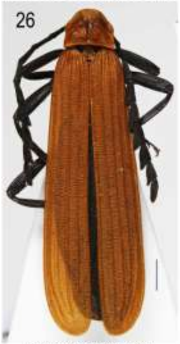

Metriorrhynchus sp., Laos

Metriorrhynchinae: Metriorrhynchini: Cautirina

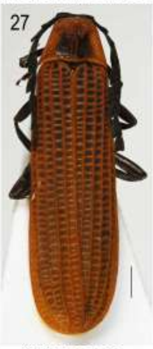

Xylobanus sp., Laos

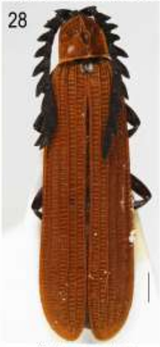

Xylobanus sp., Laos

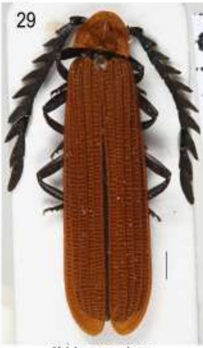

Xylobanus sp., Laos

Pattern: uniform yellow to orange

Body size: 9-16 mm

Distribution:
Indo-Burma

Further Lycidae co-mimics

Non-lycid co-mimics:
Cantharidae, Cerambycidae

Remark: some groups flower visiting (especially *Metriorrhynchus* and *Calochromus*, others seldom)

Metriorrhynchinae: Lycoprogenthini

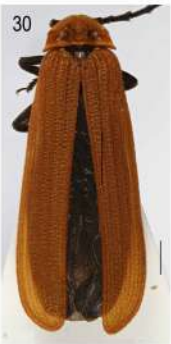

Lycoprogenthes sp., Laos

M.:M.:Metanoeina

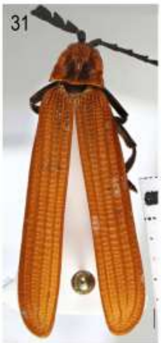

Xylometanoeus sp., Laos

Calochrominae: Calochromini

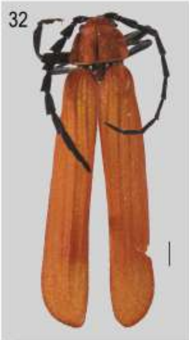

Calochromus giganteus Pic, Thailand

Indo-Burma

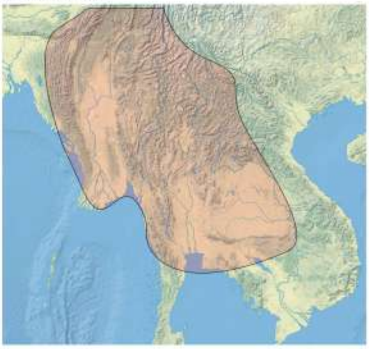

Metriorrhynchinae: Metriorrhynchini: Cautirina

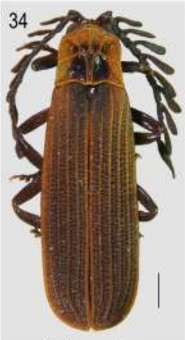

Cautires sp., Laos

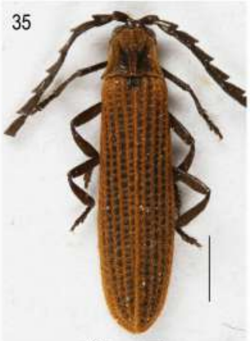

Xylobanus sp., Laos

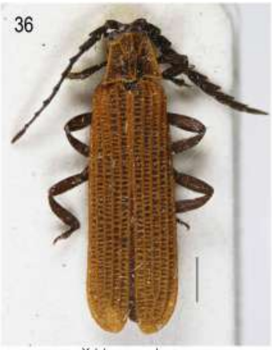

Xylobanus sp., Laos

Pattern: uniform yellow to orange, sometimes bottom of elytra cells dark coloured and reticulate structure apparent from low distance

Body size: 5-8 mm

Distribution:
Indo-Burma

Further Lycidae co-mimics

Non-lycid co-mimics:
Cantharidae, Cerambycidae

Metriorrhynchinae: Metriorrhynchini: Cautirina

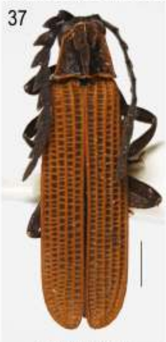

Xylobanus sp., Laos

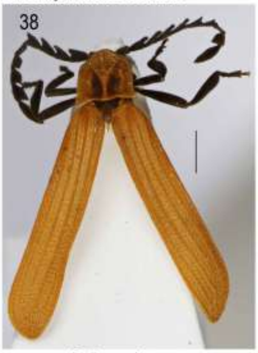

Cautires sp., Laos

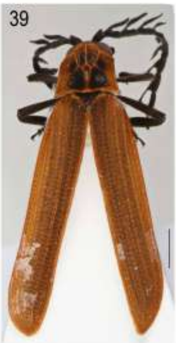

Cautires sp., Laos

All graphics and text produced by the authors as they are listed under the title of this article (CC-BY open acces license).

Pattern: bicoloured elytra (brown/black)

Body size: ~7 mm

Distribution: Indo-Burma (NE India)

Further Lycidae co-mimics: *Plateros* sp.

Non-lycid co-mimics:

Remark: Rare pattern

Metriorrhynchinae: Metriorrhynchini: Cautirina

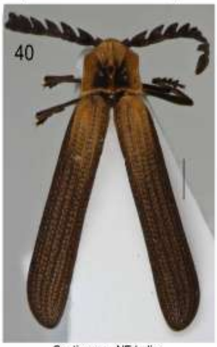

Cautires sp., NE India

Pattern: black elytra, bicoloured pronotum (black patch and bright margins)

Body size: ~7 mm

Distribution: Indo-Burma (Vietnam, Cambodia)

Further Lycidae co-mimics:

Non-lycid co-mimics:

Remark: common pattern in *Plateros* (Taiwan, Mesoamerica)

Lycinae: Platerodini

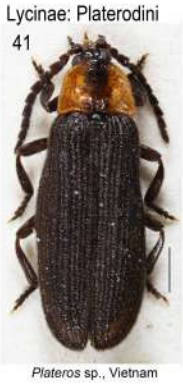

Plateros sp., Vietnam

Metriorrh.: Metriorrh.: Cautirina

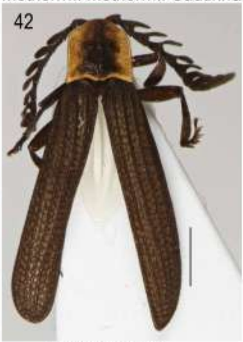

Cautires sp., NE India

Lycinae: Lycini

Pattern: uniform orange to red elytra; the pronotum with a black patch, sometimes uniform bright

Body size: 12-16 mm

Distribution: Indo-Burma

Non-lycid co-mimics: Cantharidae, Cerambycidae

Remark. A widespread pattern of large bodied lycids, *Lipernes* commonly form aggregations on flowers and fly in open situations

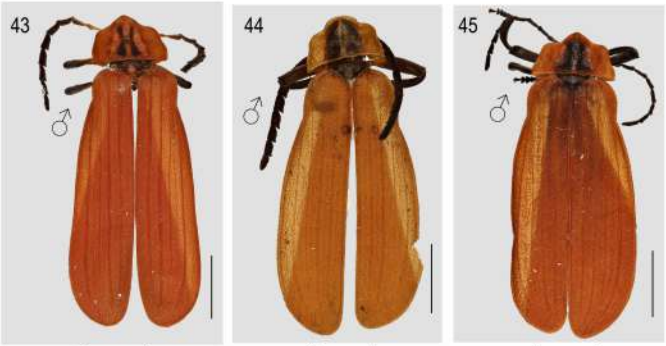

Lipernes sp., Laos | *Lipernes* sp., Laos | *Lipernes* sp., Laos

Lycinae: Lycini

Pattern: bicoloured elytra, most elytra orange to red, apices black; the pronotum with a black patch, sometimes uniform bright

Body size: 12-16 mm

Distribution: Indo-Burma

Non-lycid co-mimics: Cantharidae, Cerambycidae

Remark. A widespread pattern of large bodied lycids, *Lipernes* commonly form aggregations on flowers and fly in open situations. Both Lipernes colour types occur also in India and some species are polymorphic

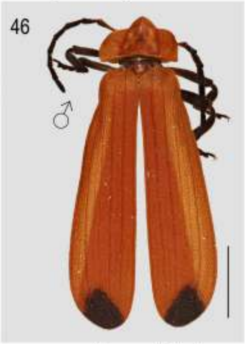

Lipernes sp., Thailand

All graphics and text produced by the authors as they are listed under the title of this article (CC-BY open acces license). Long horn beetle photographs taken by L. Dembicky.

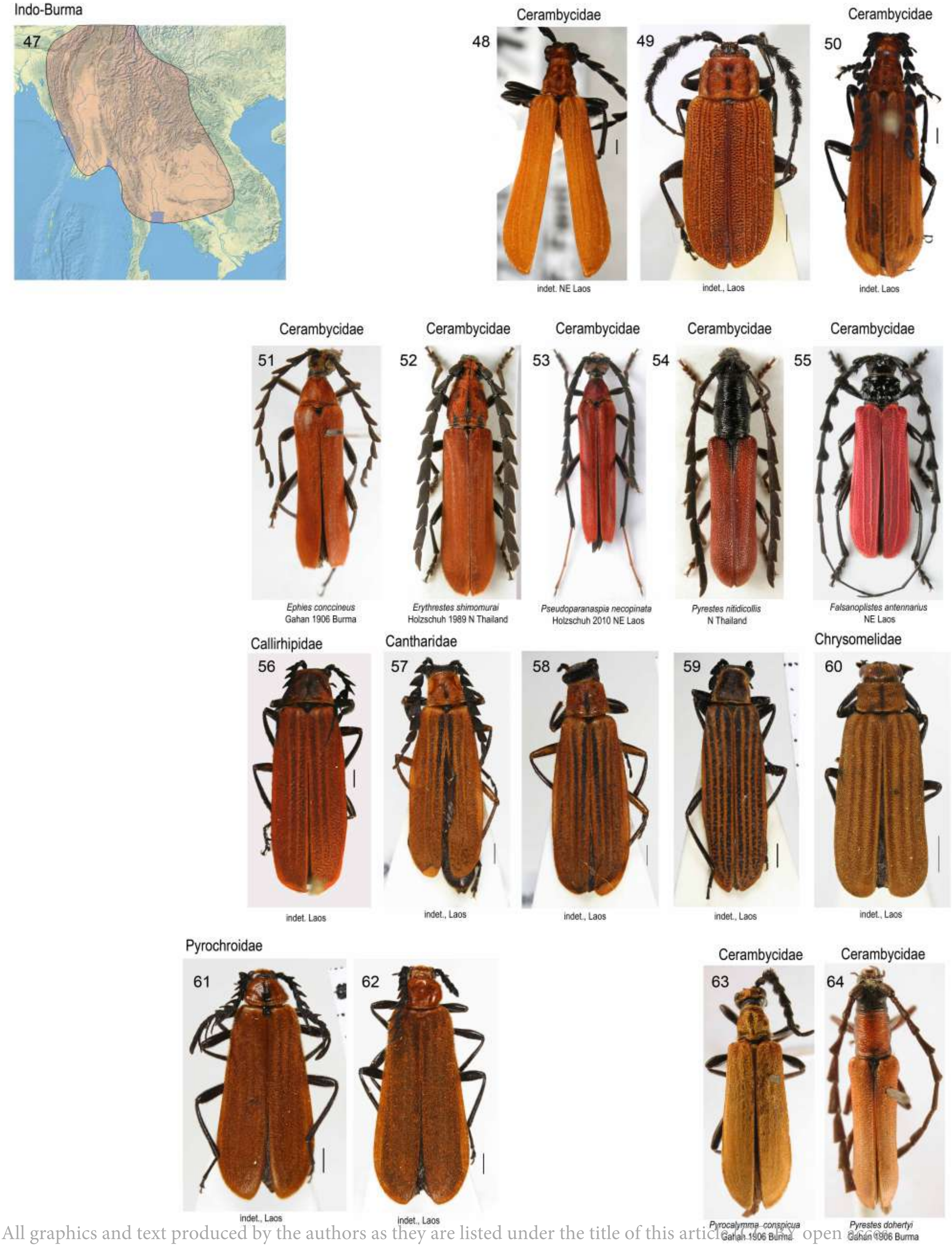

All graphics and text produced by the authors as they are listed under the title of this article (CC-BY open access license). Long horn beetle photographs taken by L. Dembicky.

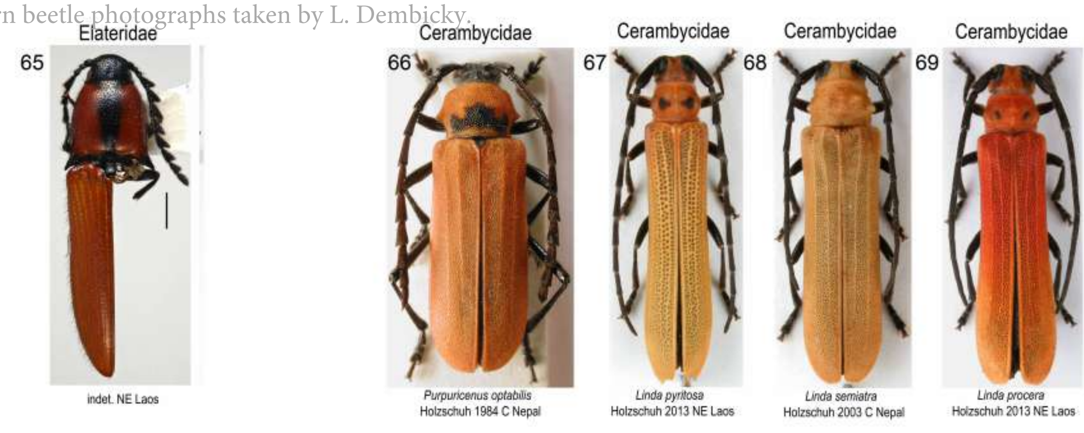

Oriental region

70

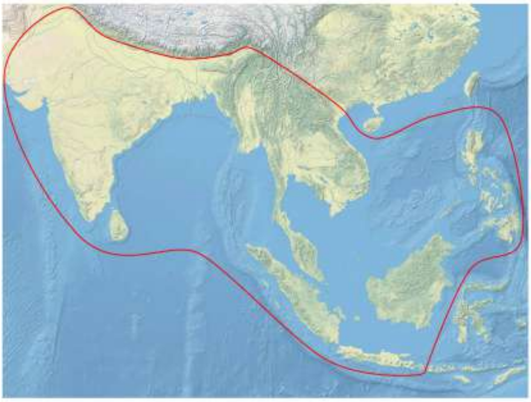

Southern India and Sri Lanka

71

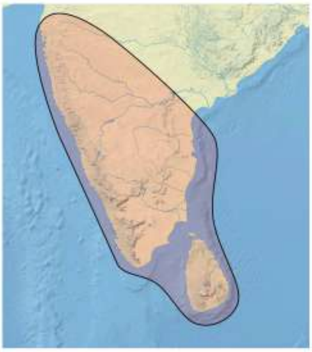

The phylogenetic structure of the Lycidae fauna

Subfamily	Tribe /Subtribe	# spp. (woldwide)	# spp. (India&Sri L.)
Dexorinae	Dexorini	15 spp.	
	Mimolibnetini	5 spp.	
Erotinae	Erotini	54 spp.	
	Dictyopterini	73 spp.	
	Taphini	31 spp.	5 spp.
	Slipinskiini	46 spp.	
Calochrominae	Calochromini	289 spp.	~20 spp.
Ateliinae	Ateliini	45 spp.	3 spp.
	Lyponiini	45 spp.	
	Macrolycini	69 spp.	
Lyropaeinae	Lyropaeini	43 spp.	5 spp
	Alyculini	5 spp.	
	Antennolycini	3 spp.	
	Platerodrilini	49 spp.	
Lycinae	Conderini	42 spp.	
	Eurrhacini	102 spp.	
	Thonalmini	11 spp.	
	Leptolycini	12 spp.	
	Platerodini	861 spp.	50 spp.
	Calopterini	367 spp.	
	Lycini	413 spp.	~10 spp.
Metriorrhynchinae	Dihammatini	44 spp.	
	Lycoprogenthini	7 spp.	3 spp.
	Libnetini	112 spp.	5 spp.
	Dilophotini	81 spp.	
	Metriorrhynchini	1410 spp.	
	Metanoeina	40 spp.	
	Cautirina	750 spp.	20 spp.
	Metriorrhynchina	620 spp.	
Total		4234 spp.	~100 spp,

The presence of net-winged beetle aposematic patterns in the region

Group	Colour type	Characteristics	+/-	Examples
Uniform coloration	black	pronotum an elytra uniformly black to dark brown		
	yellow	pronotum and elytra yellow to light brown	✓	77-81, 86, 87, 92, 93
	orange and red	pronotum and elytra brightly orange or cinnamon red	✓	72-74, 90, 91
	metallic (blue, green)	pronotum and elytra metalic, all shades of colours		
Bi-colored pronotum/ elytra	black/yellow	pronotum black (at most with bright margins), elytra yellow		
	black/red	pronotum black (at most with bright margins), elytra red		
	bright/black	pronotum brightly colored, elytra uniformly black		
	red/merallic	pronotum brightly red, elytra metallic blue		
Bi-colored elytra	yellow/black	elytra bi-colored; humeral part yellow, apical part dark coloured	✓	82, 83
	red(orange)/black	elytra bi-colored; humeral part orange/red, apical part dark	✓	84, 85, 88, 92
	black/bright	elytra bi-colored; humeral part black, apex yellow/orange/red		
Fasciate elytra	yellow/black	humeri and apex of elytra black, middle of elytron yellow		
	bright/black	humeri and apex of elytra brigt, middle of elytron black	✓	*Lyropaeus fallax* Walker (not shown)
	yellow/metallic	most elytra black with blue metallic shine, middle yellow		
Striate elytra	bright/black	elytra brown/brightly coloured, suture or middle of elytron black		
Punctate el.	bright/black	elytra brown/brightly coloured, black patch in each elytron	✓	73, *Lyropaeus biguttatus* Waterhouse (not shown)
Tri-colored el.	all combinations of colors	elytron with three differently coloured parts		
Reticulate	bright/black	backgroud colour of elytra dark, costae large, brightly coloured		
Non-categorized aposematic patterns		see listed examples for further information		

Figures S70–S95. The overview of the lycid fauna, coloration, and co-mimics: Oriental region, Southern India, and Sri Lanka.

All graphics and text produced by the authors as they are listed under the title of this article (CC-BY open acces license). Long horn beetle photographs taken by L. Dembicky.

Pattern: uniform red elytra and pronotum

Body size: ~ 9 mm

Distribution: Sri Lanka

Non-lycid co-mimics: not recorded

Lycinae: Platerodini

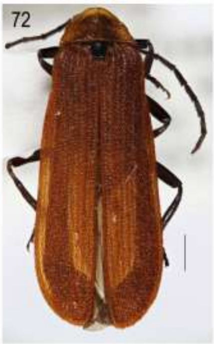

Plateros rubripennis Pic, Sri Lanka

Pattern: uniform red elytra and pronotum, some with apical patches

Body size: ~ 10 mm

Distribution: Sri Lanka

Non-lycid co-mimics: Cantharidae

Remark. The flower visiting lycids that commonly form aggregations

Calochrominae: Calochromini

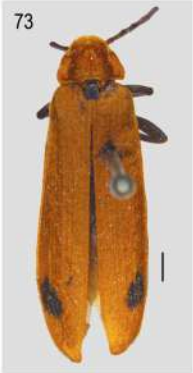

Micronychus bimaculatus Motsch., Sri Lanka

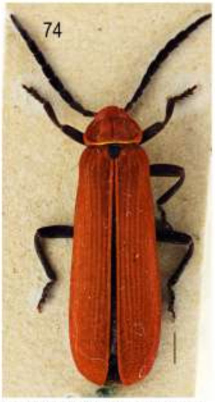

Micronychus patruelis Kleine, Sri Lanka

Pattern: uniform red elytra and pronotum, the elytra with black apices

Body size: ~ 10 mm

Distribution: Sri Lanka, south India

Non-lycid co-mimics: Cantharidae

Remark. The flower visiting lycids that commonly form aggregations with *Lipernes* spp. (see the next page)

Calochrominae: Calochromini

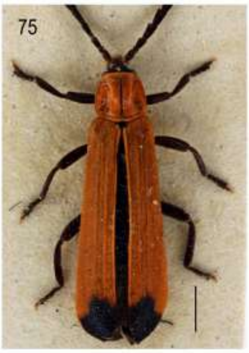

Micronychus terminatus Waterh., Sri Lanka

Pattern: red humeral part of the elytra with black apices; the pronotum red

Body size: ~ 9 mm

Distribution: Sri Lanka, south India

Non-lycid co-mimics: Cantharidae

Remark. The lycids kiving in moist places under the canopy

Lycinae: Platerodini

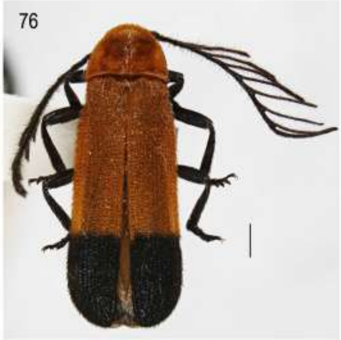

Plateros pubicornis Walker, Sri Lanka

Pattern: uniform testaceous elytra and pronotum

Body size: ~ 6 mm

Distribution: Sri Lanka

Non-lycid co-mimics: Cantharidae

Remark. The small-bodied lycids common on leaves under the forest canopy

Lycinae: Platerodini

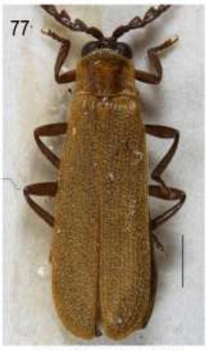

Plateros fulgens Kleine, India

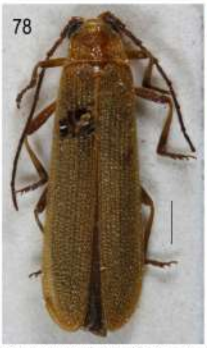

Plateros arcuatus Boc. et Boc., Sri Lanka

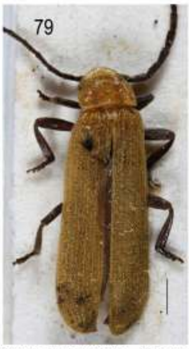

Plateros languidus Waterh., Sri Lanka

Metriorrhynchinae: Metriorrhynchini: Cautirinae

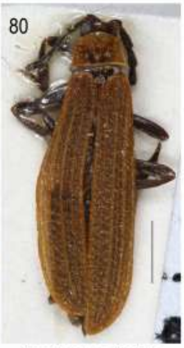

Prometanoeus sp., Sri Lanka

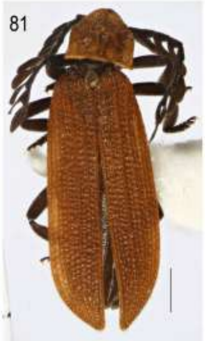

Cautires sp., India

Pattern: bicoloured testaceous and black elytra; the pronotum black or dark coloured, pronotal margins light coloured

Body size: ~ 5 mm

Distribution: Sri Lanka

Non-lycid co-mimics: Cantharidae

Remark. The small-bodied lycids common on leaves under the forest canopy

Lycinae: Platerodini

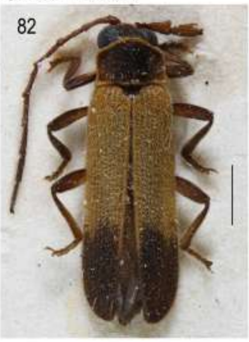

Plateros persubtilis Boc. et Boc., Sri Lanka

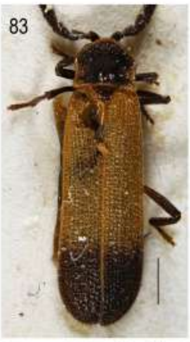

Plateros reconditus Boc.et Boc., Sri Lanka

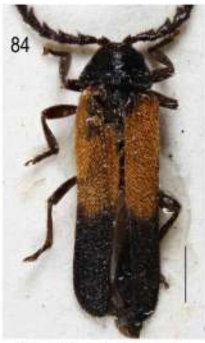

Plateros nietneri Waterh., Sri Lanka

Pattern: bicoloured orange and black elytra; the pronotum orange

Body size: ~ 7 mm

Distribution: northern India

Non-lycid co-mimics: not recorded

Remark. The rare pattern recorded from northeastern India

Lycinae: Platerodini

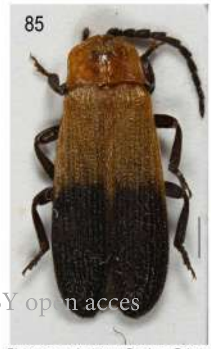

Plateros pseudopicianus Bocakova, Rajastan

All graphics and text produced by the authors as they are listed under the title of this article (CC-BY open acces license). Long horn beetle photographs taken by L. Dembicky.

Pattern: uniform yellow elytra and the pronotum

Body size: ~10 mm

Distribution: Southern India

Non-lycid co-mimics: not recorded

Remark. A rare group of neotenic lycid beetles whose colouration might not be a result of selection for the similarity

Lyropaeinae: Lyropaeini

86

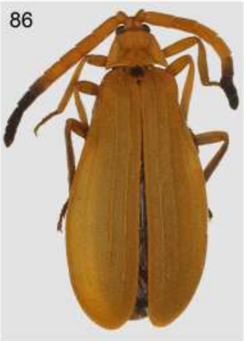

Lyropaeus sp., Southern India

Pattern: uniform yellow elytra and the pronotum

Body size: ~7.5 mm

Distribution: Southern India

Remark. A rare group of neotenic lycid beetles whose colouration resembles *Plateros* spp.

Lyropaeinae: Lyropaeini

87

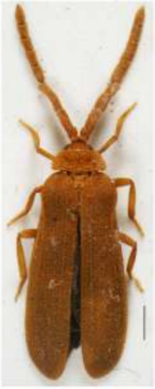

Lyropaeus sp., S. India

Pattern: bicoloured orange/black elytra and the uniform yellow pronotum

Body size: ~10 mm

Distribution: Sri lanka

Non-lycid co-mimics: *Cautires*

Remark. A rare group of neotenic lycid beetles. The similarly coloured *Cautires* differ in the body shape

Lyropaeinae: Lyropaeini

88

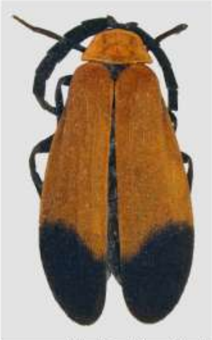

Lyropaeus ceylonicus Boc. et Boc., Sri Lanka

Pattern: uniform cinnamon red elytra and the pronotum

Body size: ~7 mm

Distribution: Sri Lanka

Non-lycid co-mimics: none

Remark. A rare group of neotenic lycid beetles.

Ateliinae: Alteliini

89

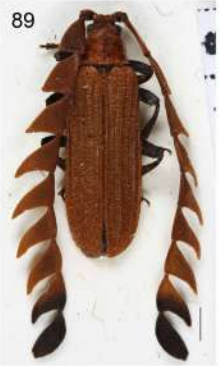

Atelius acuteicornis Kas., Sri Lanka

Pattern: uniform red elytra and the margins of the pronotum; variably sized black patches in the pronotum

Body size: 10-15 mm

Distribution: India, especially low mountain regions of northern India

Non-lycid co-mimics: Cantharidae, Cerambycidae

Remark. A common pattern displayes by flower visiting net-winged beetles. The patterns occurs in the wide area from India and Thailand to the Great Sundas

90 ♂

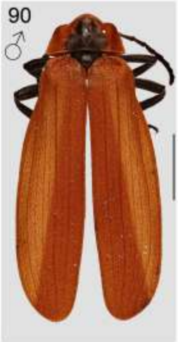

Lipernes sp., India

91 ♂

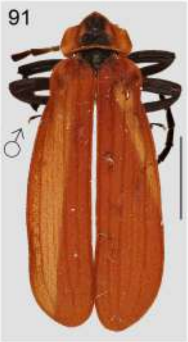

Lipernes sp., India

Pattern: uniform orange elytra and pronotum; some patches might be seen in the pronotum

Body size: 10-14 mm

Distribution: India, Sri Lanka

Non-lycid co-mimics: Cantharidae, Cerambycidae

Remark. A common pattern displayes by flower visiting net-winged beetles. The patterns occurs in the wide area from Sri lanka to Pakistan in the north and Thailand to the east

92 ♂

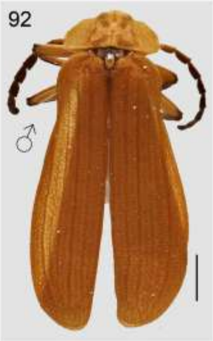

Lipernes sp., India

93 ♂

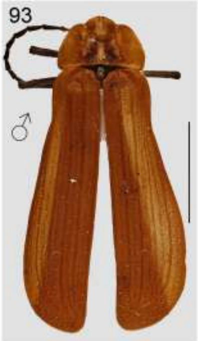

Lipernes sp., India

Pattern: uniform orange or bicoloured elytra with a black apical patch; the pronotum orange

Body size: 10-14 mm

Distribution: India, Sri Lanka

Non-lycid co-mimics: Cantharidae, Cerambycidae

Remark. A common pattern displayes by flower visiting net-winged beetles. The patterns occurs in the wide area from Sri lanka to Pakistan in the north and Thailand to the east

Intraspecific polymorphism

94 ♂

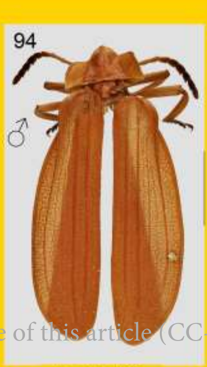

Lipernes sp., India

95 ♀

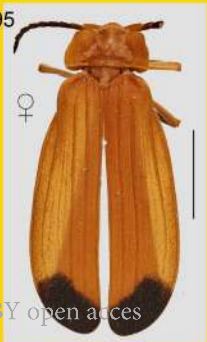

Lipernes sp., India

All graphics and text produced by the authors as they are listed under the title of this article (CC-BY open acces license). Long horn beetle photographs taken by L. Dembicky.

Oriental region

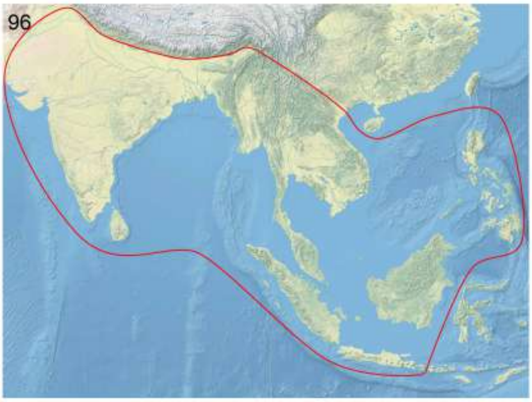

The Great Sunda Islands and the Philippines

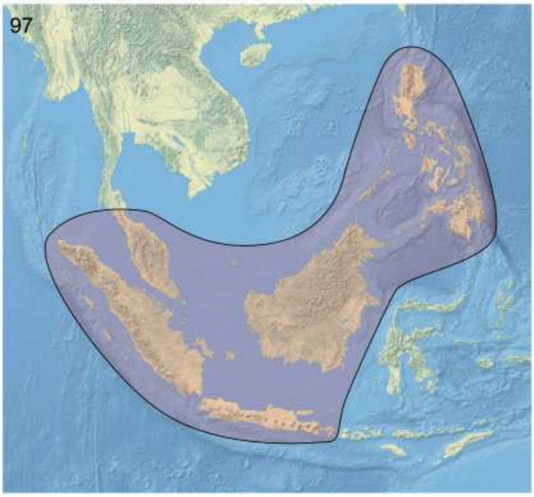

The phylogenetic structure of the Lycidae fauna

Subfamily	Tribe /Subtribe	# spp. (woldwide)	# spp. (region)
Dexorinae	Dexorini	15 spp.	
	Mimolibnetini	5 spp.	
Erotinae	Erotini	54 spp.	5 spp.
	Dictyopterini	73 spp.	5 spp.
	Taphini	31 spp.	20 spp.
	Slipinskiini	46 spp.	
Calochrominae	Calochromini	289 spp.	~80 spp.
Ateliinae	Ateliini	45 spp.	39 spp.
	Lyponiini	45 spp.	1 sp.
	Macrolycini	69 spp.	
Lyropaeinae	Lyropaeini	43 spp.	~35 spp.
	Alyculini	5 spp.	5 spp.
	Antennolycini	3 spp.	3 spp.
	Platerodrilini	49 spp.	~40 spp.
Lycinae	Conderini	42 spp.	~20 spp.
	Eurrhacini	102 spp.	
	Thonalmini	11 spp.	
	Leptolycini	12 spp.	
	Platerodini	861 spp.	~380 spp.
	Calopterini	367 spp.	
	Lycini	413 spp.	~25 spp.
Metriorrhynchinae	Dihammatini	44 spp.	~25 spp.
	Lycoprogenthini	7 spp.	3 spp.
	Libnetini	112 spp.	~60 spp.
	Dilophotini	81 spp.	~40 spp.
	Metriorrhynchini	1410 spp.	
	Metanoeina	40 spp.	~25 spp.
	Cautirina	750 spp.	~450 spp.
	Metriorrhynchina	620 spp.	~30 spp.
Total		4234 spp.	~1260 spp.

The presence of net-winged beetle aposematic patterns in the region

Group	Colour type	Characteristics	+/-	Figures
Uniform coloration	black	pronotum an elytra uniformly black to dark brown	✓	107-126
	yellow	pronotum and elytra yellow to light brown	✓	105, 106, 171, 172, 174, 175, 204-208
	orange and red	pronotum and elytra brightly orange or cinnamon red	✓	104, 201, 202
	metallic (blue, green)	pronotum and elytra metalic, all shades of colours		
Bi-colored pronotum/ elytra	black/yellow	pronotum black (at most with bright margins), elytra yellow		
	black/red	pronotum black (at most with bright margins), elytra red	✓	177, 182, 183
	bright/black	pronotum brightly colored, elytra uniformly black	✓	103, 210-212
	red/merallic	pronotum brightly red, elytra metallic blue		
Bi-colored elytra	yellow/black	elytra bi-colored. humeral part yellow, apical part dark coloured	✓	98-102, 128-14, 153-164, 183, 221-228
	red(orange)/black	elytra bi-colored. humeral part orange/red, apical part dark	✓	167-169, 179, 185-190, 192-199, 229-234
	black/bright	elytra bi-colored. humeral part black, apex yellow/orange/red	✓	203
Fasciate elytra	yellow/black	humeri and apex of elytra black, middle of elytron yellow		
	bright/black	humeri and apex of elytra brigt, middle of elytron black		
	yellow/metallic	most elytra black with blue metallic shine, middle yellow		
Striate elytra	bright/black	elytra brown/brightly coloured, suture or middle of elytron black		
Punctate el.	bright/black	elytra brown/brightly coloured, black patch in each elytron		
Tri-colored el.	all combinations of colors	elytron with three differently coloured parts		
Reticulate	bright/black	backgroud colour of elytra dark, costae large, brightly coloured	✓	214-219
Non-categorized aposematic patterns		see listed examples for further information	✓	175-181 (black elytra with brightlu colored costae)

Figures S96–S296. The overview of the lycid fauna, coloration, and co-mimics: Oriental region, The Greater Sunda Islands, and the Philippines.

All graphics and text produced by the authors as they are listed under the title of this article (CC-BY open acces license). Long horn beetle photographs taken by L. Dembicky.

Lycinae: Platerodini — Metr.: Metriorrhynchini — Metr.: Dilophotini

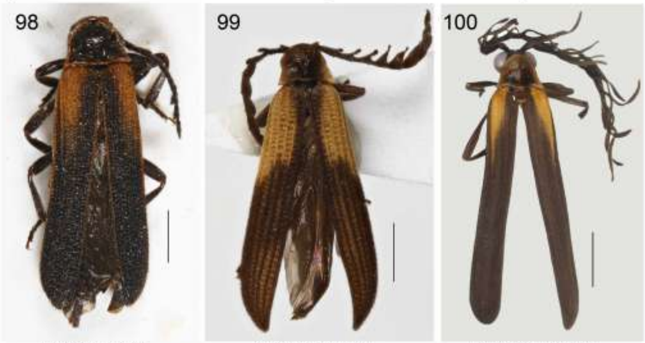

Plateros sp., Sumatra — *Xylobanus* sp., Sumatra — *Dilophotes* sp., Sumatra

Pattern: dark brown elytra with humeral testaceous to brown coloured patches,; the pronotum black with testaceous margins

Body size: ~ 6 mm

Distribution: Sumatra (mountains regions)

Non-lycid co-mimics: not recorded

Remark. The uncommon pattern known only from the Sumatran mountains

Lycinae: Platerodini

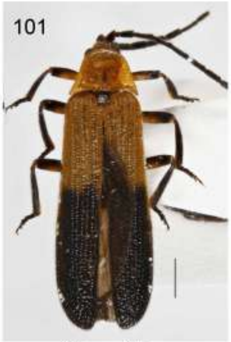

Plateros sp., Bali

Pattern: bicoloured testaceous and black elytra; the pronotum brightly coloured

Body size: ~ 7 mm

Distribution: Bali

Non-lycid co-mimics: Cantharidae

Remark. The large bodied *Plateros* similar to sympatrically occurring Metriorrhynchini

Metr.: Metriorrhynchini

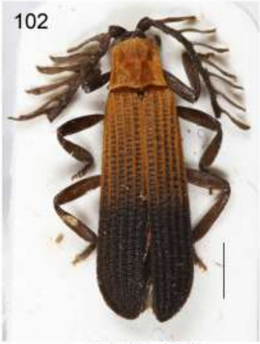

Xylobanus sp., Palawan

Pattern: bicoloured testaceous and black elytra; the pronotum brightly coloured

Body size: ~ 5 mm

Distribution: Palawan

Non-lycid co-mimics: Cantharidae

Remark. The small-bodied *Cautires* similar to sympatrically occurring *Plateros*

Metr.: Metriorrhynchini

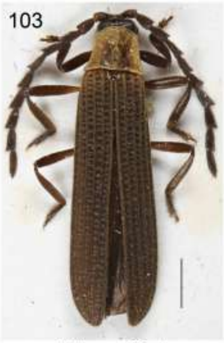

Xylobanus sp., Sumatra

Pattern: dark brown elytra; the pronotum testaceous

Body size: ~ 6 mm

Distribution: Sumatra (mountains of the Western Province)

Non-lycid co-mimics: not recorded

Remark. The small-bodied *Xylobanus* similar to sympatrically occurring *Microtrichalus* sp. The pattern known only from the mountains of the Western Sumatra province.

Lycinae: Platerodini

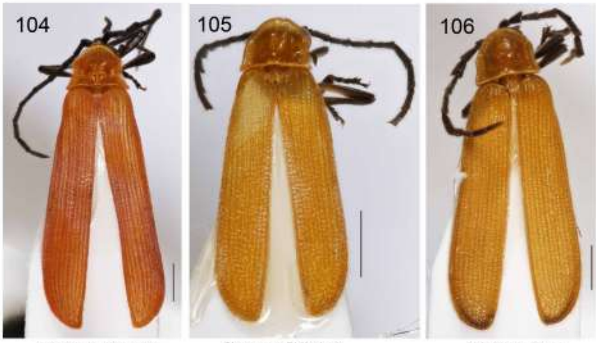

Plateros sp., Philippines — *Plateros* sp., Philippines — *Plateros* sp., Brunei

Pattern: yellow to orange elytra; the pronotum similarly coloured

Body size: 4-8 mm

Distribution: The Great Sundas, Philippines

Non-lycid co-mimics: Cantharidae, Lampyridae (Luciolinae), Chrysomelidae (Galerucinae, Cassidinae: Hispini)

Remark. The small- to medium-sized Plateros similar to sympatrically occurring *Libnetis* spp. The widespread patternin Southeastern Asia

All graphics and text produced by the authors as they are listed under the title of this article (CC-BY open acces license). Long horn beetle photographs taken by L. Dembicky.

Malay Peninsula: Cameron Highlands

107

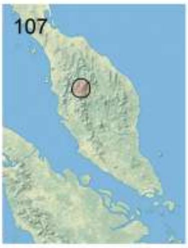

Pattern: uniform black

Body size: 6-13 mm

Distribution: Malaya: Cameron Highlands

Non-lycid co-mimics:
Cantharidae, Pyrochroidae, Oedemeridae, Cerambycidae,

Remark. The black coloured mountain species with a quite large body size. The relationships of black coloured *Cautires* studied by Jiruskova et al. (2019)

Metriorrhynchinae: Metriorrhynchini: Cautirina

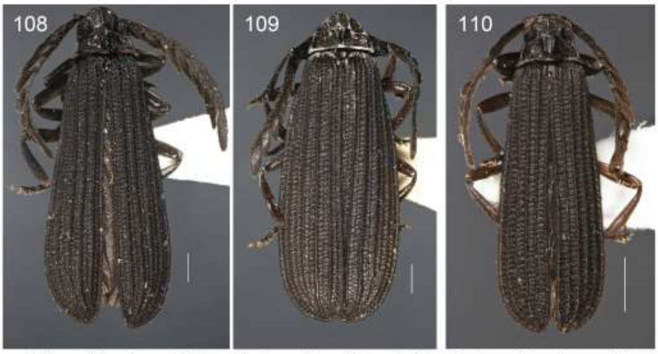

Cautires sp., Malaya (Cameron Highl.) | *Cautires* sp., Malaya (Cameron Highl.) | *Cautires* sp., Malaya (Cameron Highl.)

Metriorrhynchinae: Metriorrhynchini: Cautirina | Dictyopterinae: Dictyopterini | Lyropaeinae: Lyropaeini

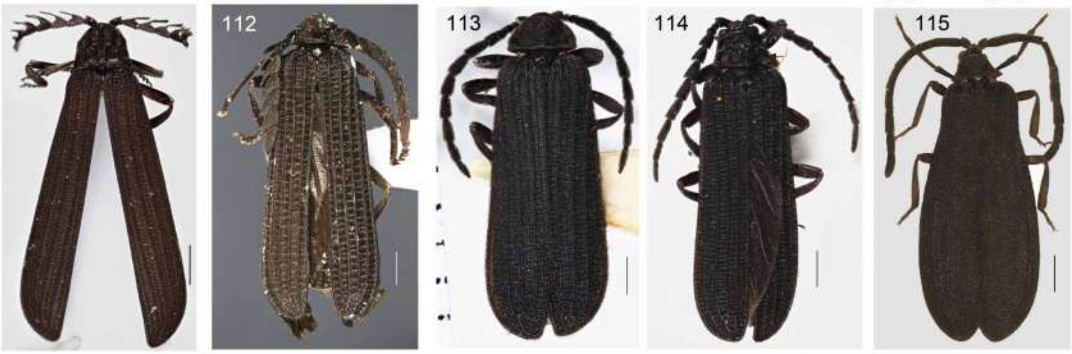

Cautires communis Jiruskova, Malaya | *Xylobanus* sp., Malaya (Cameron Highl.) | indet., Malaya | *Pyropterus* sp., Malaya | *Lyropaeus* sp., Malaya

Malay Peninsula, Sumatra, Borneo, western Java

116

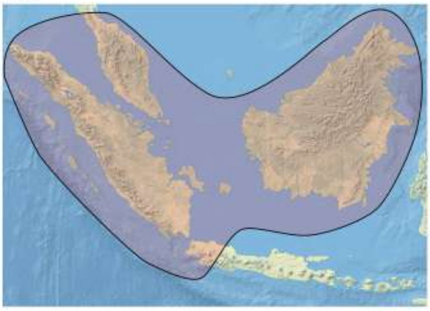

Pattern: uniform black

Body size: ~6 mm

Distribution: the Greats Sundas

Non-lycid co-mimics:
Cantharidae

Remark. The black coloured lowland species with a small body size.

Metriorrhynchinae: Metriorrhynchini: Cautirina

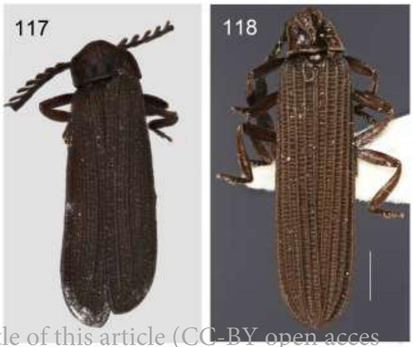

Cautires sp., Borneo (Sabah) | *Xylobanus* sp., Borneo (Sabah)

All graphics and text produced by the authors as they are listed under the title of this article (CC-BY open acces license). Long horn beetle photographs taken by L. Dembicky.

Metr.:Metr.: Cautirina | Lycinae: Platerodini | Metriorrhynchinae: Dilophotini | Ateliinae:Ateliini

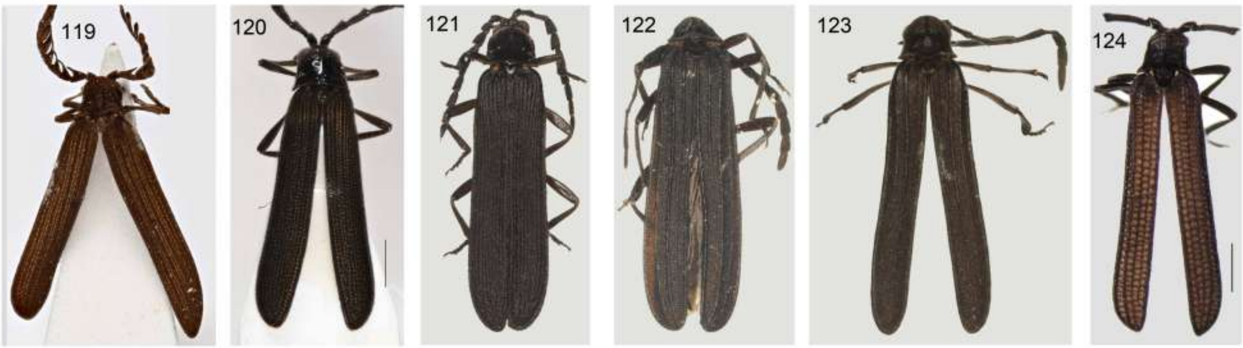

Cautires rianganus Pic, Sumatra | *Plateros* sp., Philippines | *Plateros* sp., Malaya | *Dilophotes* sp., Malaya | *Dilophotes* sp., Borneo | *Scarelus anthracinus* Boc., Malaya

Malay Peninsula, Sumatra, Borneo

125

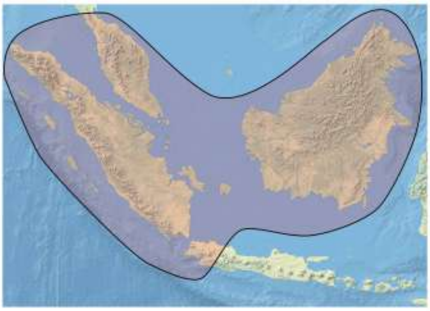

Pattern: uniform black

Body size: ~3 mm

Distribution: the Greats Sundas

Non-lycid co-mimics: not recorded

Remark. The black coloured neotenic lycids with a very small body size. Possibly not involved in mimetic rings due to reareness and a specific life history

Lyropaeinae: Platerodrilini

126

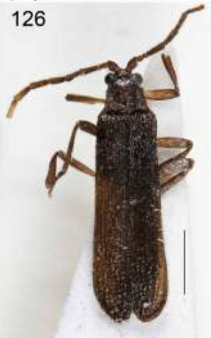

Horakiella pahangenis Boc. et Boc., Malaya

Malay Peninsula, Sumatra, Borneo, western Java

127

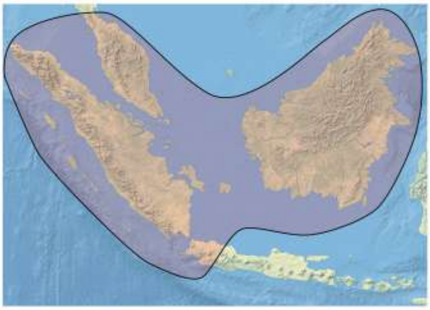

Pattern: bicoloured elytra, humeri brown, apex dark coloured; the pronotum black or dark brown

Body size: 5-11 mm

Distribution: Malaya and the Greater Sundas

Non-lycid co-mimics: Cantharidae, Cerambycidae

Remark. The widespread pattern with local forms

Metriorrhynchinae: Metriorrhynchini: Cautirina

128

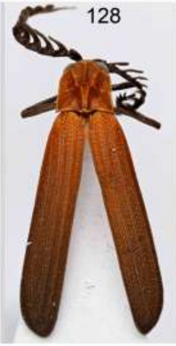

Cautires katarinae Jiruskova, Malaya

129

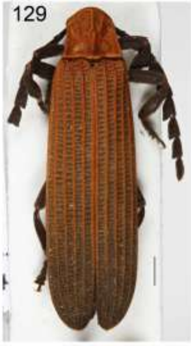

Xylobanus sp., Sumatra

130

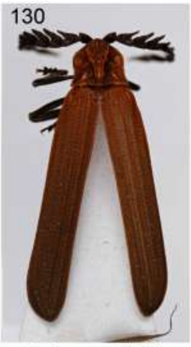

Cautires reverandi Pic, Malaya

Metriorrhynchinae: Metriorrhvnchini: Cautirina

131

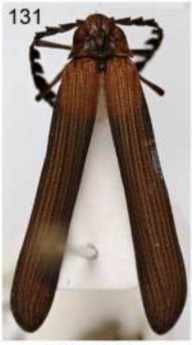

Cautires sp., Malaya

132

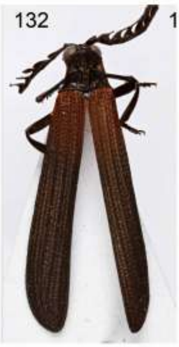

Cautires arribasae Jiruskova, Malaya

133

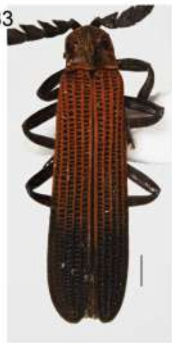

Xylobanus sp., Sumatra

134

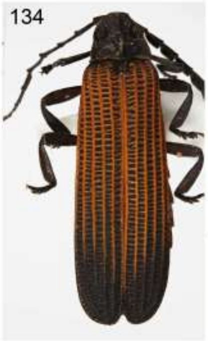

Cautires sp., Malaya

135

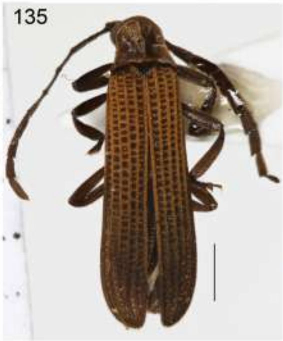

Xylobanus sp., Malaya

136

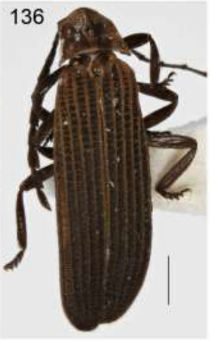

Cautires sp., Sumatra

Metriorrhynchinae: Metriorrhynchini: Cautirina

137

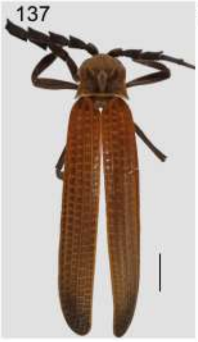

Xylobanus sp., Borneo

138

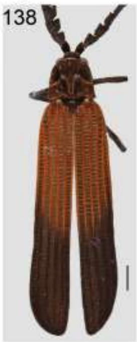

Xylobanus sp., Borneo

Metanoeina

139

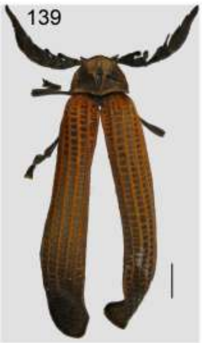

Xylometanoeus sp., Borneo

Metriorrhynchinae: Metriorrhynchini: Cautirina

140

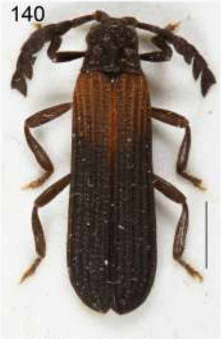

Cautires sp., Borneo (Sarawak)

141

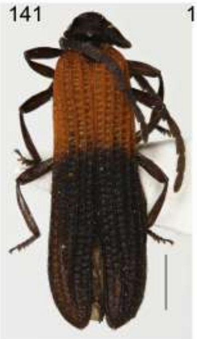

Xylobanus sp., Western Java

142

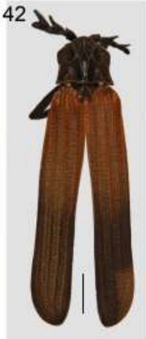

Cautires sp., Borneo

143

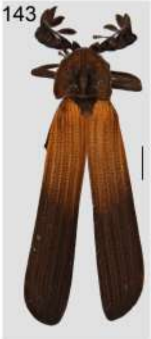

Cautires sp., Borneo

All graphics and text produced by the authors as they are listed under the title of this article (CC-BY open acces license). Long horn beetle photographs taken by L. Dembicky.

Lycinae: Platerodini

144

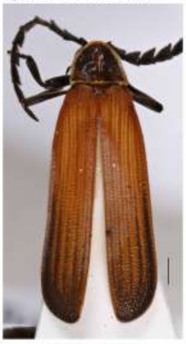

Plateros sp., Borneo

145

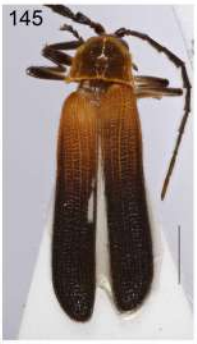

Plateros sp., Borneo

146

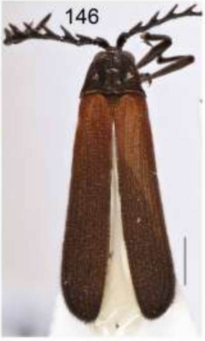

Plateros sp., Sumatra

147

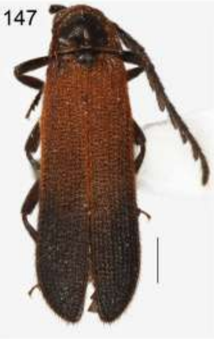

Plateros sp., Sumatra

Lyropaeinae: Platerodrilini

148

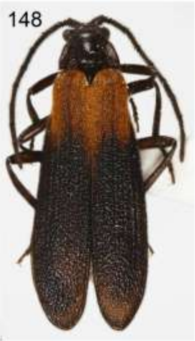

Platerodrilus sp., Malaya

Ateliinae: Ateliini

149

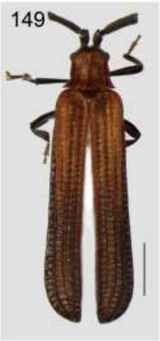

Scarelus umbrosus Kln., Malaya

Pattern: bicoloured elytra, the humeral part with dark red elytral costae, elytral apices black; the pronotum black

Body size: 6-10 mm

Distribution: Sumatra, Malaya

Non-lycid co-mimics: not recorded

Remark. A form of the previous pattern

Metriorrhynchinae: Metriorrhynchini: Cautirina

150

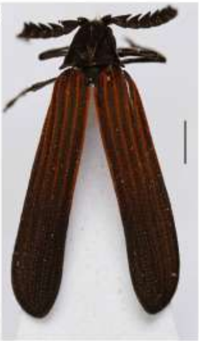

Cautires indus Kirsch, Malaya

151

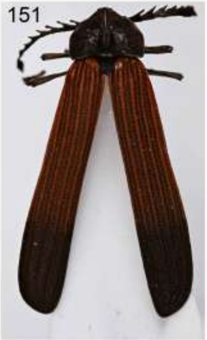

Cautires pauperulus Bourgeois, Malaya

Malay Peninsula, Sumatra, Borneo, western Java

152

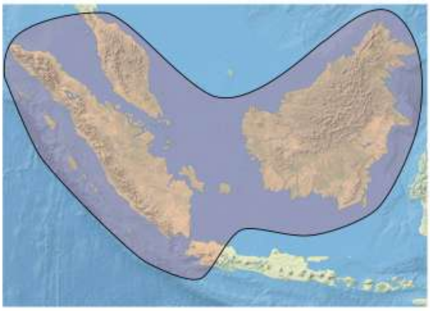

Pattern: bicoloured testaceous and black elytra; the pronotum black or dark coloured, pronotal margins light coloured

Body size: ~ 5 mm

Distribution: Malaya, the Greater Sundas

Non-lycid co-mimics: Cantharidae, Chrysomelidae: Cassidinae: Hispini

Metriorrhynchinae: Dihammatini

153

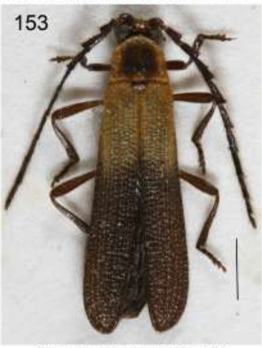

Dihammatus cf. *ponderosus*, Philippines

Lycinae: Platerodini

154

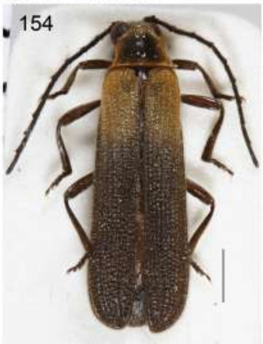

Plateros contiguus, Philippines

Lycinae: Platerodini

Remark. The small-bodied lycids common on leaves under the forest canopy

The dominant pattern in whole area.

155

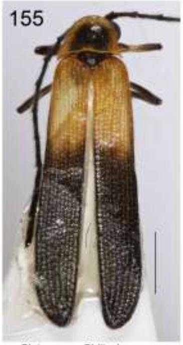

Plateros sp., Philippines

156

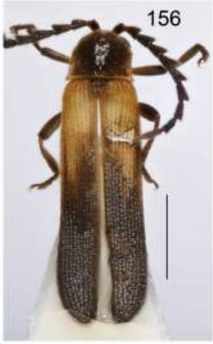

Plateros sp., Borneo

157

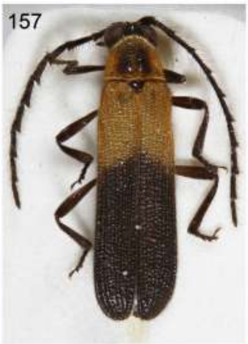

Plateros cf. *tenebrans*, Philippines

158

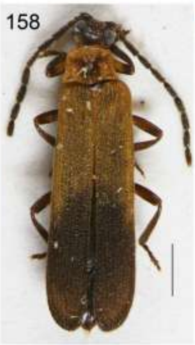

Plateros sp., Philippines

159

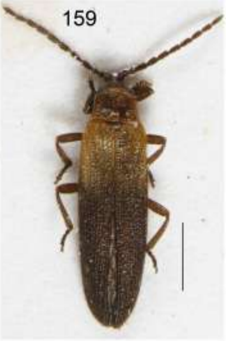

Plateros sp., Philippines

Pattern: bicoloured yellow and black elytra; the pronotum black or dark coloured, pronotal margins sometimes light coloured

Body size: ~ 5 mm

Distribution: Malaya, the Great Sundas

Non-lycid co-mimics: not recorded

Remark. The small-bodied lycids common on leaves under the forest canopy

Metriorrhynchinae: Libnetini

160

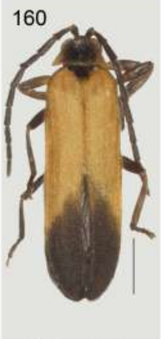

Libnetis sp., Sumatra

161

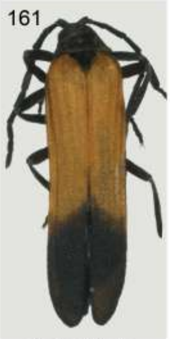

Libnetis sp., Sumatra

Metriorrhynchinae: Dilophotini

162

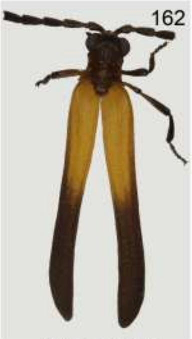

Dilophotes sp., Sumatra

163

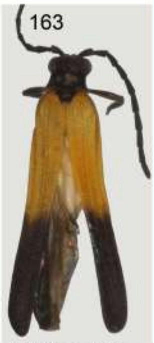

Dilophotes sp., Sumatra

sexual dimorphism

164

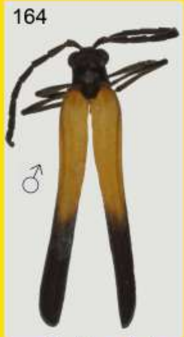

♂

Dilophotes sp., Sumatra

A form of the preceeding pattern, it differs in the colour shade and the extent of apical patches

Metriorrhynchinae: Libnetini

165

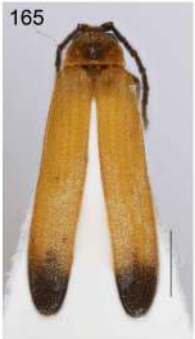

Pattern: bicoloured red and black elytra; the pronotum black

Body size: ~ 5 mm

Distribution: Malaya, the Great Sundas

Non-lycid co-mimics: none

Remark. The putative mimics of red/black lycids (Motyka *et al.* 2018)

Metriorrhynchinae: Dilophotini

167

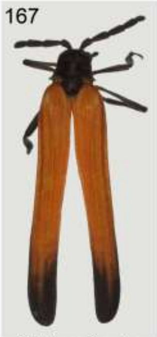

Dilophotes sp., Sumatra

168

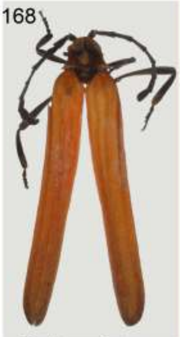

Dilophotes sp., Sumatra

169

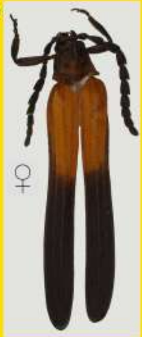

♀

Dilophotes sp., Sumatra

All graphics and text produced by the authors as they are listed under the title of this article (CC-BY open acces license). Long horn beetle photographs taken by L. Dembicky.

Malay Peninsula, Sumatra, Borneo, western Java

170

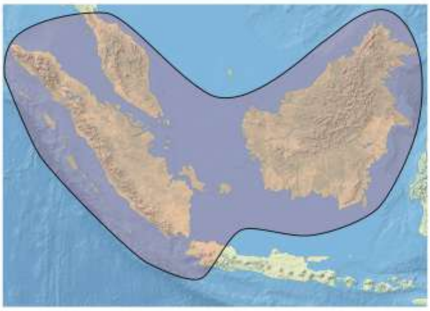

Pattern: uniform yellow elytra elytra; the pronotum also yellow

Body size: ~ 5 mm

Distribution: Malaya, the Great Sundas

Non-lycid co-mimics: Cantharidae

Remark. The small-bodied lycids occurring on leaves under the forest canopy

Metriorrhynchinae: Dihammatini

171

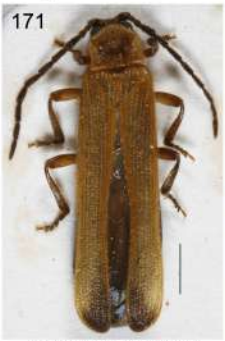

Dihammatus cribripennis, Sumatra

Metr.: Dilophotini

172

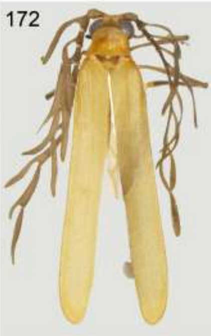

Dilophotes sp., Laos

Malay Peninsula, Sumatra, Borneo

173

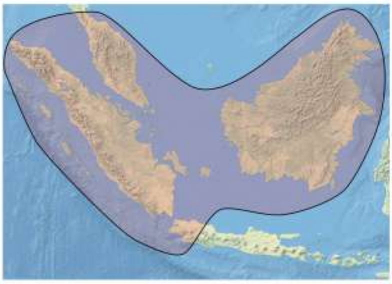

Pattern: uniform red

Body size: ~ 6 mm

Distribution: Malaya, the Great Sundas

Non-lycid co-mimics: none

Remark. The pattern known from Indo-Burma and eastern Palearctic region

Erotinae: Dictyopterini

174

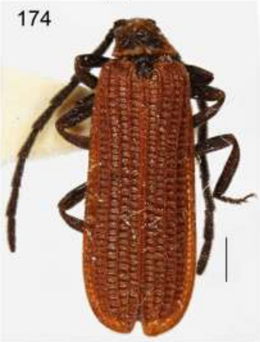

Pyropterus sp., Malaya

175

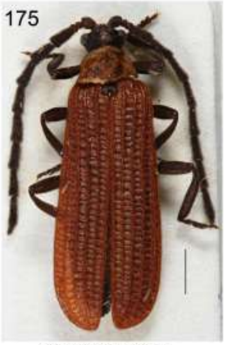

Pyropterus sp., Palawan

Malay Peninsula, Sumatra, Borneo, western Java, Palawan

176

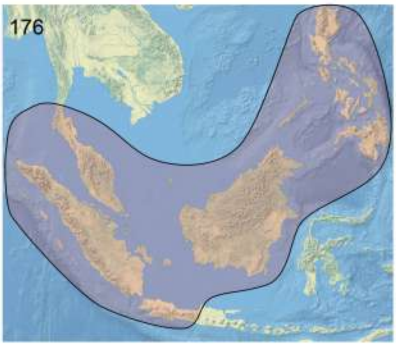

Pattern: uniform red elytra and the black pronotum

Body size: ~ 6 mm

Distribution: Malaya, the Great Sundas, the Philippines

Non-lycid co-mimics: none

Remark. The pattern known from Indo-Burma and eastern Palearctic region

Erotinae: Taphini

177

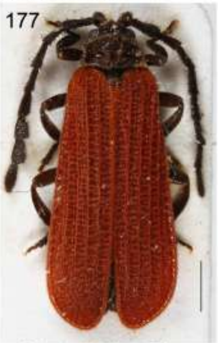

Taphes brevicollis Waterh., Palawan

Philippines: Mindanao

178

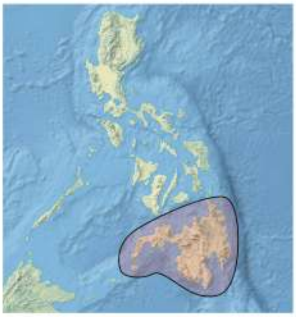

Pattern: bicoloured elytra, humeri orange, pronotum similarly coloured

Body size: ~ 5 mm

Distribution: the philippines

Non-lycid co-mimics: none

Remark. The unique pattern in the Taphini, resembles sumpatrically occurring net-winged beetles

Erotinae: Taphini

179

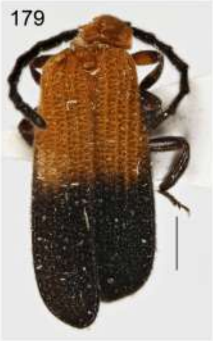

Taphes sp., Mindanao

Borneo: the Kinabalu massif

180

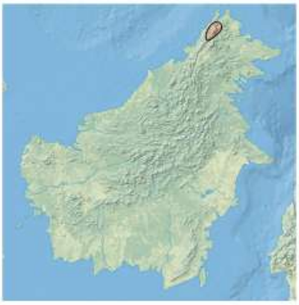

Pattern: bicoloured elytra, black with brightly red parts of longitudinal costae

Body size: ~ 9 mm

Distribution: Borneo - the Kinabalu massif

Non-lycid co-mimics: none

Remark. The pattern known from an extremely limited area in high mountain ecosystems

181

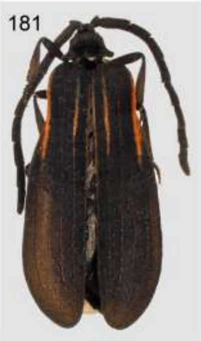

Lyropaeus monticola Kln., Borneo

All graphics and text produced by the authors as they are listed under the title of this article (CC-BY open acces licence). Long horn beetle photographs taken by L. Dembicky.

Philippines

182

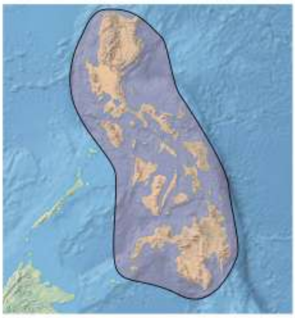

Pattern: bicoloured elytra, humeri yellow, the apex and elytral suture black

Body size: ~ 7 mm

Distribution: the Philiuppines

Non-lycid co-mimics: Cantharidae

Remark. The pattern known from the Philippines only

183

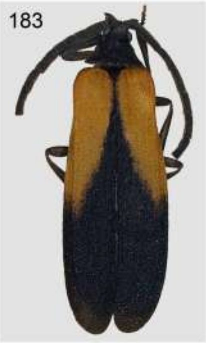

Lyropaeus sp., Philippines

Malay Peninsula, Sumatra, Borneo, western Java

174

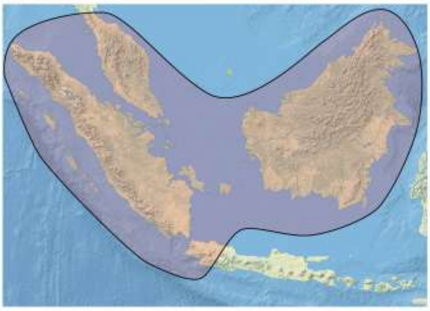

Pattern: bicoloured elytra, black coloured except the basal parts of humeral longitudinal costae; the pronotum black

Body size: ~ 5 mm

Distribution: lowlands of Malaya and the Great Sundas

Furhter lycid comimics: *Microtrichalus rianganus* Pic (Metriorrhynchina)
Non-lycid co-mimics: not recorded

Remark. The pattern uncommon in the region, some individuals found within under-canopy aggregations

Metriorrhynchinae: Metriorrhynchini: Cautirina

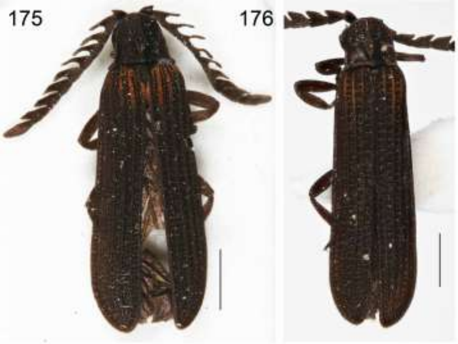

Cautires sp., Sumatra — *Xylobanus* sp., Sumatra

Lycinae: Platerodini — Metriorrhynchinae: Dilophotini

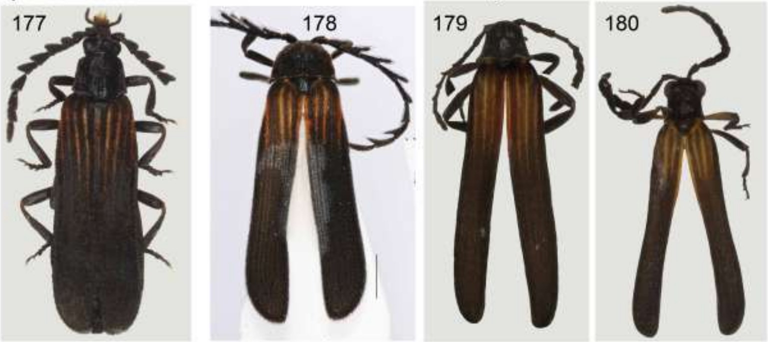

Plateros sp., Borneo — *Plateros* sp., Borneo — *Dilophotes* sp., Borneo — *Dilophotes* sp., Sumatra

Malay Peninsula

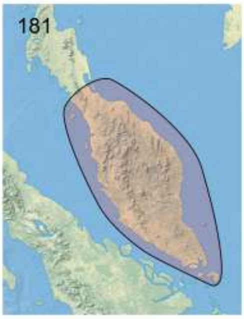

Pattern: uniform cinnamn brown elytra, the pronotum black

Body size: ~ 10 mm

Distribution: the lowlands of Malaya

Non-lycid co-mimics: not recorded

Remark. The pattern rare in the region where dominate species with bi-coloured elytra

Calochrominae: Calochromini

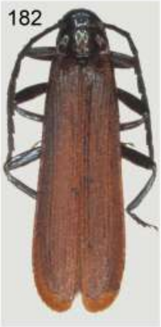

Calochromus sp., Malaya

Pattern: uniform red elytra, the pronotum black

Body size: ~ 10 mm

Distribution: the lowlands of Malaya

Non-lycid co-mimics: not recorded

Remark. The pattern rare in the region where dominate species with bi-coloured elytra. Calochromini visit flowers where they co-occur with large bodied Lycini and seldom Cautirina

Calochrominae: Calochromini

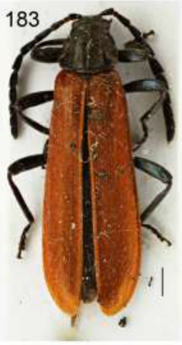

Calochromus sp., Malaya

Malay Peninsula, Sumatra, Borneo, western Java

184

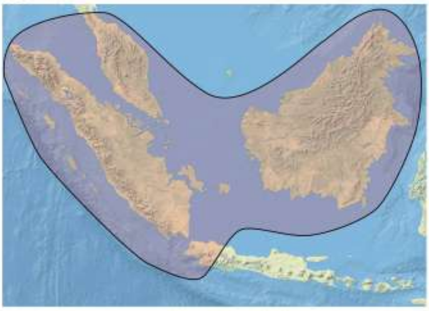

Pattern: bicoloured red/black elytra, the pronotum black or orange, sometimes metallic blue

Body size: ~ 10 mm

Distribution: Malaya and the Great Sundas

Non-lycid co-mimics: Cantharidea, Cerambycidae

Remark. The pattern common in the region; except *Dilophotes* all species visit flowers and interact with the large-bodied red/black Lycini

Calochrominae: Calochromini — MetriorrhynchinaeDilophotini

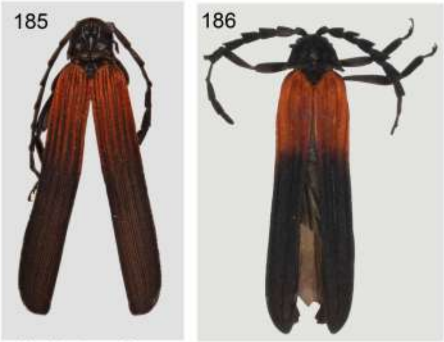

Metriorrhynchus sp., Borneo — *Dilophotes* sp., Sumatra

All graphics and text produced by the authors as they are listed under the title of this article (CC-BY open acces license). Long horn beetle photographs taken by L. Dembicky.

Calochrominae: Calochromini

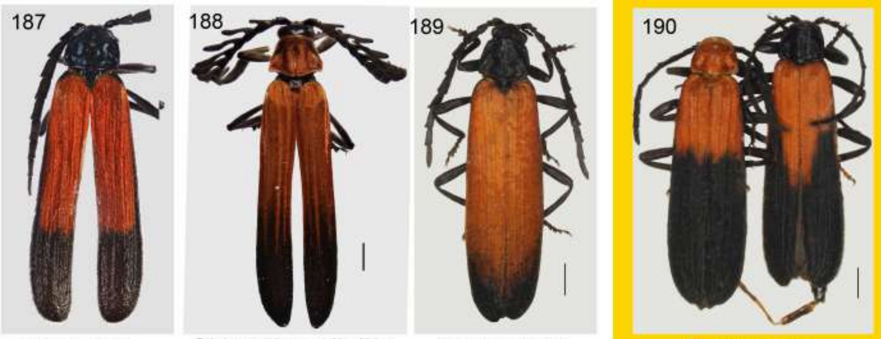

Micronycus sp., Borneo — *Calochromus kelantanensis* Mot., Malaya — *Micronychus* sp., Sumatra — *Micronychus* sp., Sumatra

intraspecific polymorphism

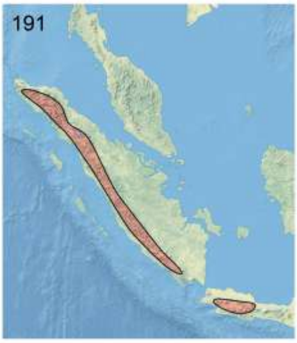

Pattern: bicoloured orange and black elytra; the pronotum orange

Body size: 7-16 mm

Distribution: the mountain of Sumatra and western Java

Non-lycid co-mimics: Cantharidae, Cerambycidae

Remark. The mwdium- to large bodied lycids common on leaves under the forest canopy, occasionally visit flowers but never in masses unlike the Lycini and Calochromini

Metriorrhynchinae: Metriorrhynchini: Cautirina

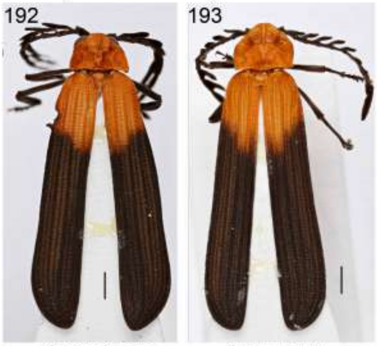

Cautires sp., Sumatra — *Cautires* sp., Sumatra

Metriorrhynchinae: Metriorrhynchini: Cautirina

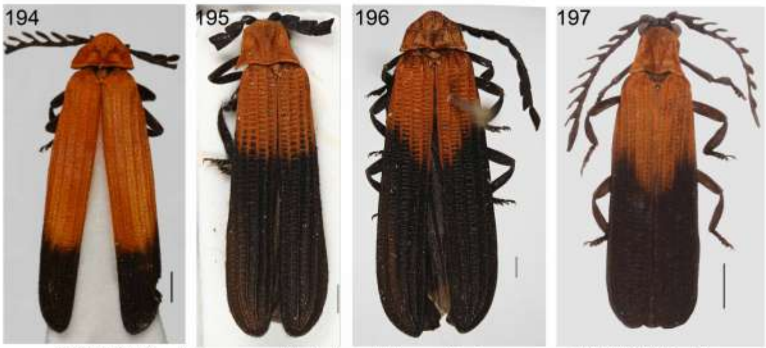

Cautires sp., Sumatra — *Xylobanus corporaali* Pic, Sumatra — *Xylobanus* sp., Sumatra — Cautires wittmeri Boc., Java

Metriorrhynchinae: Metriorrhynchini: Cautirina

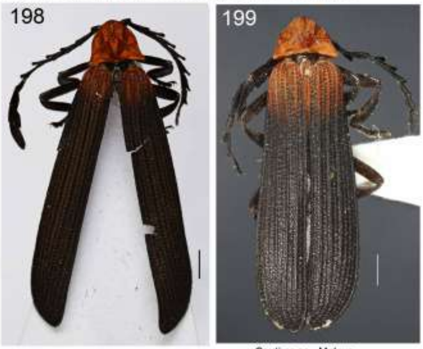

Cautires sp., Malaya — *Cautires* sp., Malaya

Remark. The uncommon pattern in Malaya; most Malay species have brown/black elytra or the mountain species are black. These beetles occasionally visit flowers where they occur together with the Calochromini.

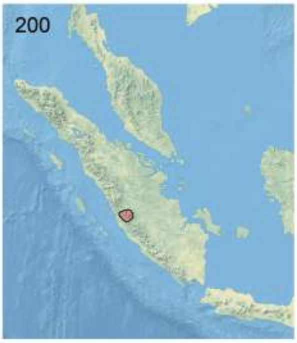

Metriorrhynchinae: Metriorrhynchini: Cautirina

Pattern: uniform orange elytra and the pronotum orange

Body size: ~10 mm

Distribution: the Gunung Kerinci mountain in Jambi (Sumatra) and western Java (Mt. Gede)

Non-lycid co-mimics: Cantharidae, Cerambycidae

Remark. The rare pattern

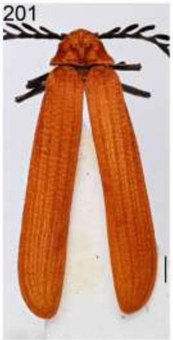

Cautires sp., Sumatra

Metr.: Metr.: Cautirina

Pattern: uniform orange elytra and the yellowish pronotum
Body size: ~10 mm

Distribution: the Gunung Kerinci mountain in Jambi (Sumatra)

Non-lycid co-mimics: none

Remark. The rare pattern known from a similar locality, a similar pattern known from mountains ot eastern New Guinea (Metriorrhynchina)

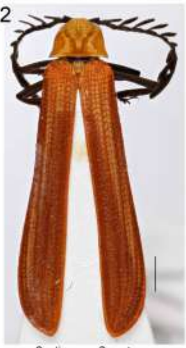

Cautires sp., Sumatra

Metr.: Metr.: Cautirina

Pattern: bicoloured black/red elytra and the black pronotum
Body size: ~10 mm

Distribution: the Gunung Kerinci mountain in Jambi (Sumatra)

Non-lycid co-mimics: none

Remark. The rare pattern known from a singlelocality, the brightly coloured elytra apices and black humeri are rare in the Lycidae and no other species with a similar pattern is known from South East Asia

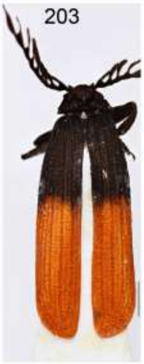

Cautires sp., Sumatra

All graphics and text produced by the authors as they are listed under the title of this article (CC-BY open access license). Long horn beetle photographs taken by L. Dembicky.

Metr.: Metr.: Metanoeina — Metriorrhynchinae: Metriorrhynchini: Cautirina

Pattern: uniform yellow elytra and the pronotum

Body size: ~10 mm

Distribution: South East Asia

Non-lycid co-mimics: Cantharidae, Oedemeridae, Cerambycidae

Remark. The widespread pattern

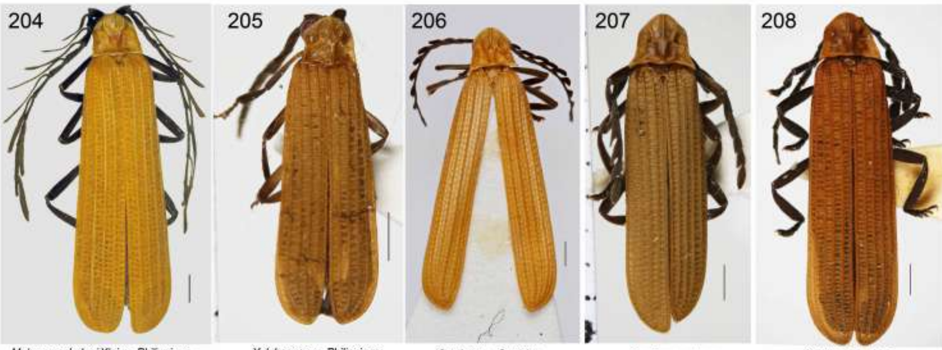

Metanoeus bakeri Kleine, Philippines — *Xylobanus* sp., Philippines — *Cautires* sp., Sumatra — *Cautires* sp., Java — *Cautires* sp., Java

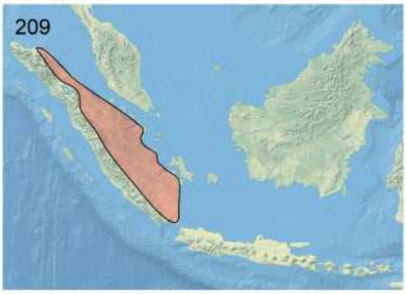

Pattern: uniform black elytra; the blck pronotum sometimes with a black patch(es)
Body size: ~8 mm

Distribution: Malaya and the Great Sundas

Non-lycid co-mimics: not recorded
Not shown: *Leptoptrichalus* sp., *Microtrichalus* sp.
Remark. A quite common pattern known from the lowlands and lower mountains of South East Asia

Metriorrhynchinae: Metriorrhynchini: Cautirina

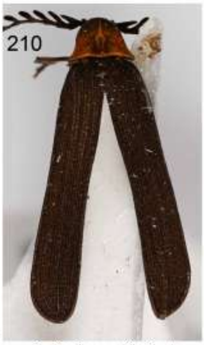

Cautires rianganus Pic, Sumatra

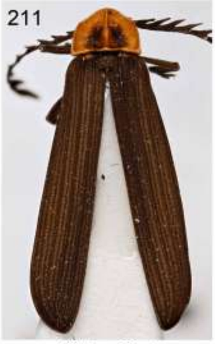

Cautires sp., Sumatra

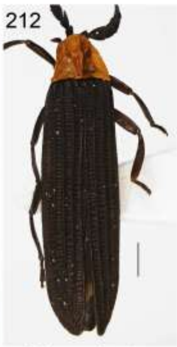

Xylobanus sp., Sumatra

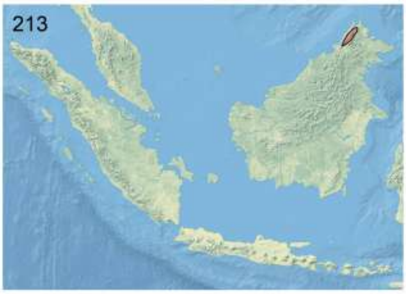

Pattern: elytra with a reticulate structure of costae with large cells, the apex of elytra black, the pronotum black

Body size: 8-13 mm

Distribution: the Mt. Kinabalu massif (Sabah), elevations over 1200 m.

Non-lycid co-mimics: none

Remark. The pattern known from a similar locality, a similar pattern known from mountains ot eastern Andes (Lycinae: Calopterini)

Metriorrhynchinae: Metriorrhynchini: Metanoeina

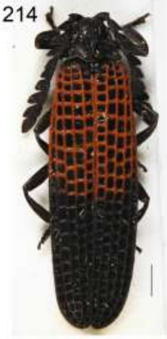

Xylometanoeus longeareolatus Kleine, Borneo

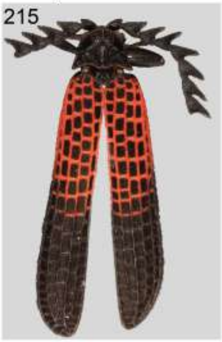

Metriorrhynchinae: Metriorrhynchini: Cautirina

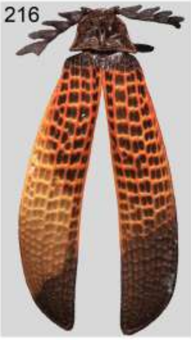

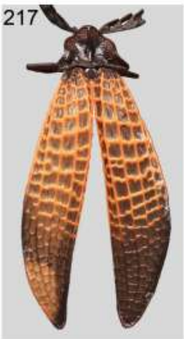

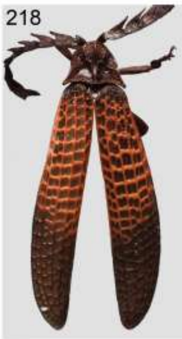

Pattern: elytra with a reticlate structure of setae which form false "elytral cells", the apex of elytra black, the pronotum blue metallic black

Body size: 8-13 mm

Distribution: the Mt. Kinabalu massif (Sabah), elevations over 1200 m.

Non-lycid co-mimics: none

Remark. The evolution of this pattern was discussed by Motyka et al. (in press)

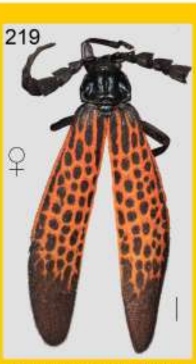

Micronychus pardus Kas., Borneo

sexual dimorphism

All graphics and text produced by the authors as they are listed under the title of this article (CC-BY open acces license). Long horn beetle photographs taken by L. Dembicky.

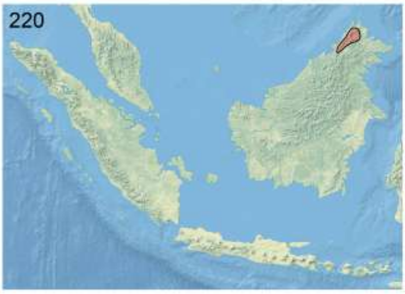

Pattern: bicoloured yellow and black elytra; the pronotum black

Body size: 5-12 mm

Distribution: mountains of Borneo (Sabah)

Non-lycid co-mimics: not recorded

Remark. The pattern with a limited distribution

Metr.: Metr.: Cautirina

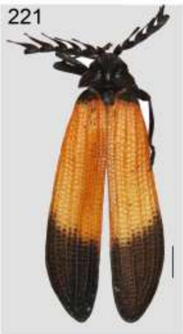

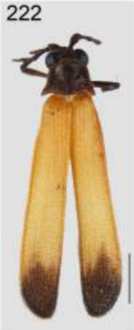

Plateros sp., Borneo

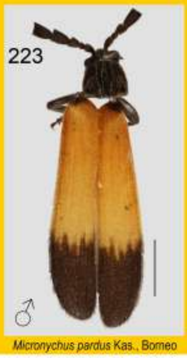

Micronychus pardus Kas., Borneo

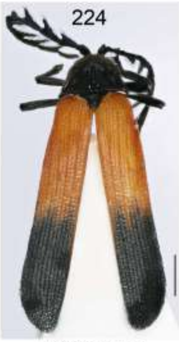

Plateros sp., Borneo

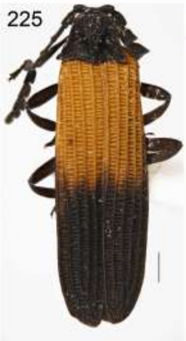

Xylobanus sp., Borneo (Sabah)

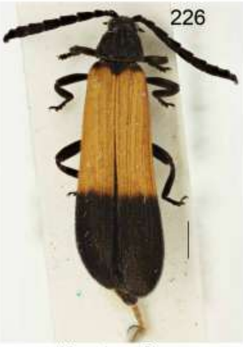

Micronychus sp., Borneo

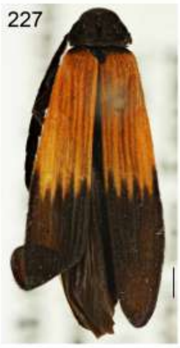

Micronychus sp., Borneo

A possible form of the previous pattern. The characteristic shape of the apical patch known in a single species.

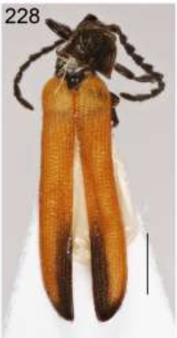

Plateros sp., Borneo

Lycinae: Lycini

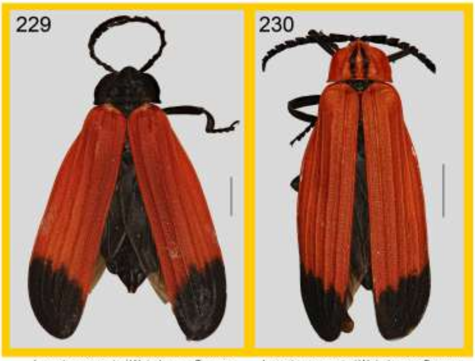

Lycostomus gestroi Waterhouse, Borneo   *Lycostomus gestroi* Waterhouse, Borneo

Pattern: bicoloured red and black elytra; the pronotum black or red coloured

Body size: ~ 20 mm

Distribution: Malaya, Sumatra, Borneo

Non-lycid co-mimics: Cerambycidae

Remark. The large-bodied lycids visiting flowers

The geographically defined intraspecific polymorphism

Lycinae: Lycini

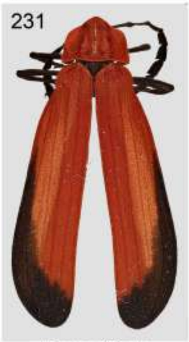

Lipernes sp., Sumatra

Pattern: bicoloured red and black elytra; the pronotum red

Body size: ~ 15 mm

Distribution: Sumatra

Non-lycid co-mimics: none

Remark. The large-bodied lycids visiting flowers; a unique pattern occurring together with other *Lipernes* spp.

Lycinae: Lycini

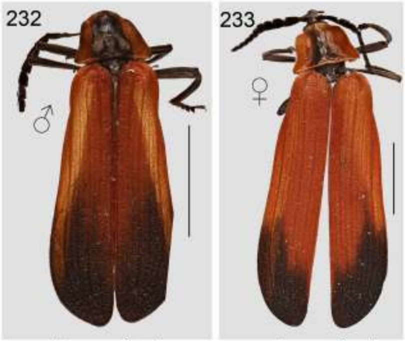

Lipernes sp., Sumatra   *Lipernes* sp., Sumatra

Pattern: bicoloured red and black elytra; the pronotum with a black patch and red pronotal margins

Body size: ~ 15 mm

Distribution: Sumatra

Non-lycid co-mimics: Cerambycidae

Remark. The large-bodied lycids visiting flowers

Calochrominae: Calochromini

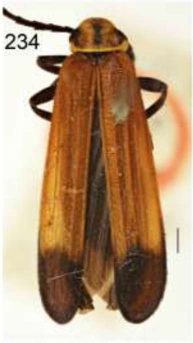

Micronychus vestitus (Wath.), Malaya

Pattern: bicoloured orange and black elytra; the pronotum with a black patchs and orange background

Body size: ~ 12 mm

Distribution: Malaya

Non-lycid co-mimics: Cerambycidae

Remark. The large-bodied lycids visiting flowers

All graphics and text produced by the authors as they are listed under the title of this article (CC-BY open acces license). Long horn beetle photographs taken by L. Dembicky.

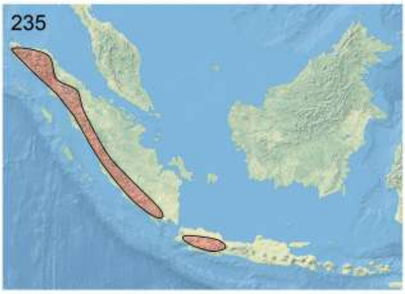

Cantharidae

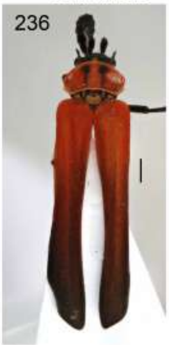

indet. sp. Malaysia, Pahang

Callirhipidae

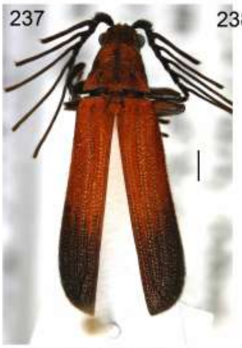

indet. sp. Malaysia, Johor

Cerambycidae

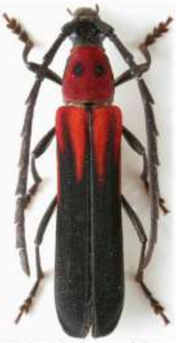

Erythrus rhombeus paucirufus
West Sumatra

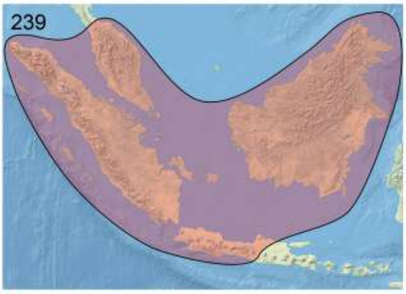

Cantharidae

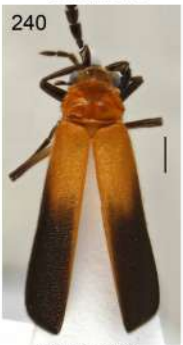

Silinae sp. Sumatra

Cantharidae

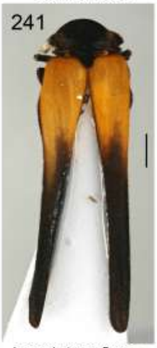

Laemoglyptus sp. Borneo

Cantharidae

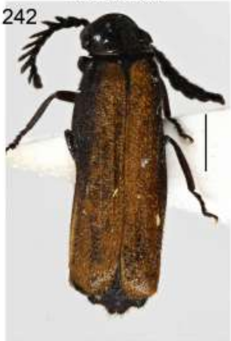

indet. sp. Malaysia, Perak

Cantharidae

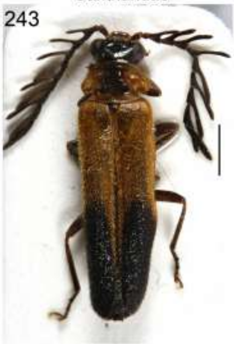

Laemoglyptus sp. Sumatra

Cantharidae

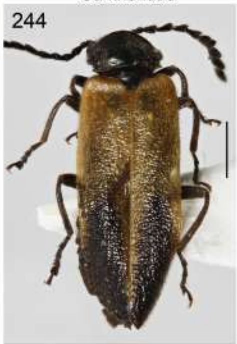

indet. sp. Java

Tenebrionidae

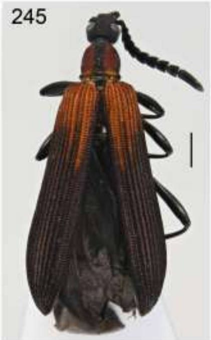

indet. sp. Malaysia, Cameron Highlands

Elateridae

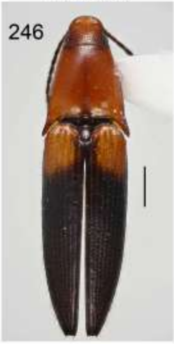

indet. sp. Sumatra

Cantharidae

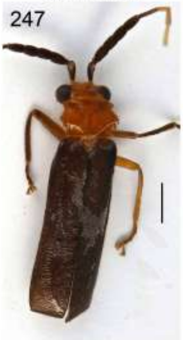

indet. sp. Sumatra

Cerambycidae

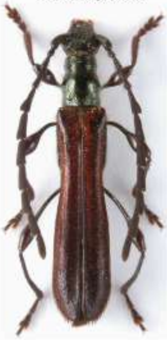

Plutonesthes rubiginea chlorizans
W Sumatra

Cerambycidae

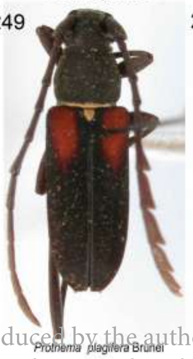

Prothema plagifera Brunei

Cerambycidae

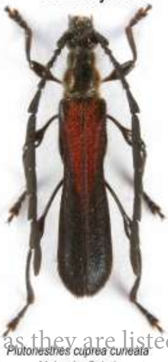

Plutonesthes cuprea cuneata
Malaysia, Sabah

Cerambycidae

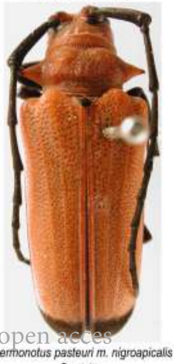

Thermonotus pasteuri m. nigroapicalis
Sumatra

All graphics and text produced by the authors as they are listed under the title of this article (CC-BY open acces license). Long horn beetle photographs taken by L. Dembicky.

Borneo: Sabah, Sarawak

252

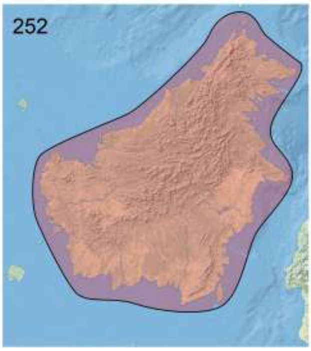

Cerambycidae

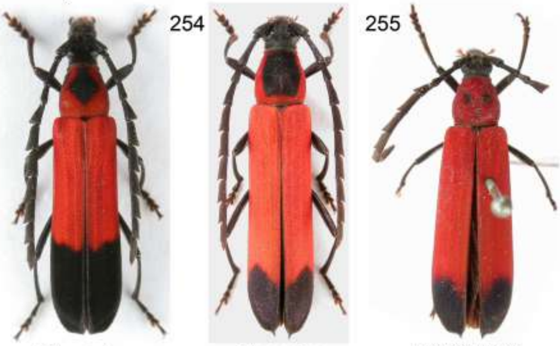

253 *Erythrus rhombeus* Malaysia, Sabah

254 *Erythrus concisus* Malaysia, Sabah

255 *Erythrus bimaculatus* Malaysia, Sarawak

Cerambycidae

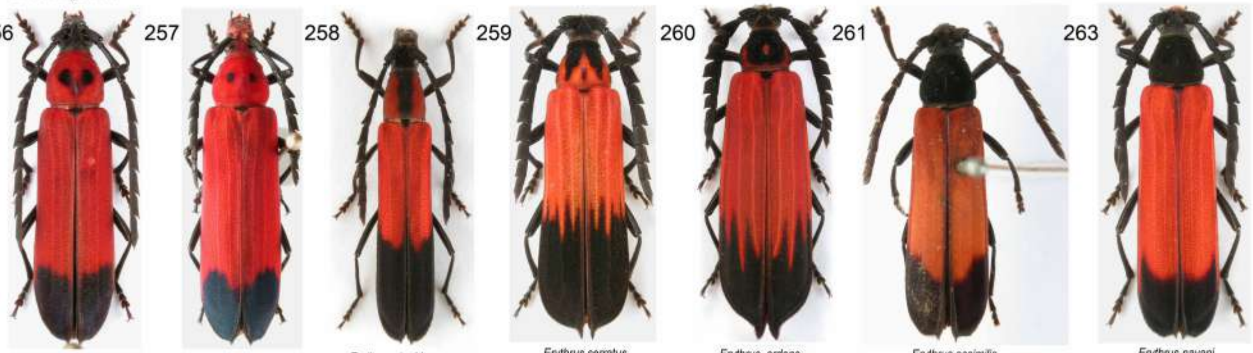

256 *Erythrus usitatus* Malaysia, Sabah

257 *Erythrus magnus* Malaysia, Sabah

258 *Erythrus stenideus* Malaysia, Sabah

259 *Erythrus serratus* Malaysia, Sabah

260 *Erythrus ardens* Malaysia, Sabah

261 *Erythrus assimilis* Malaysia, Sarawak

263 *Erythrus nayani* Malaysia, Sabah

Cerambycidae

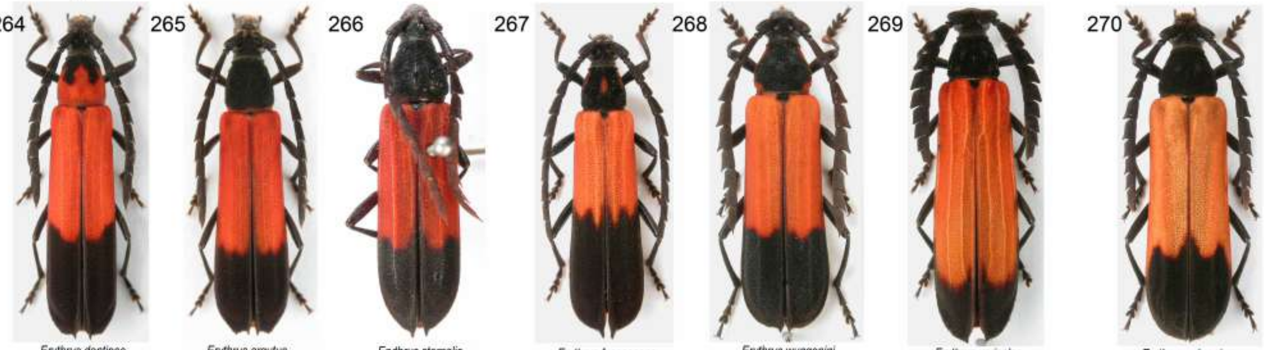

264 *Erythrus dentipes* Malaysia, Sabah

265 *Erythrus argutus* Malaysia, Sabah

266 *Erythrus sternalis* Malaysia, Sabah

267 *Erythrus fuscescens* Malaysia, Sabah

268 *Erythrus wuggenigi* Malaysia, Sabah

269 *Erythrus varicolor* Malaysia, Sabah

270 *Erythrus ochreatus* Malaysia, Sabah

Cerambycidae

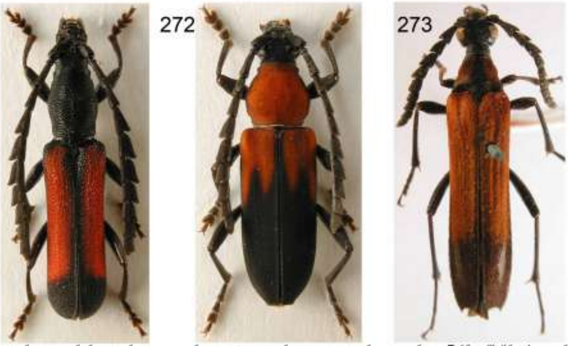

271 *Pyrestes praeceps* Malaysia

272 *Cymaterus recens* Malaysia, Sabah

273 *Ephies dilaticornis* Malaysia, Sarawak

Cerambycidae

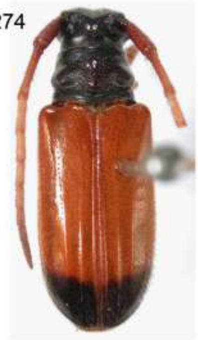

274 *Astathes pseudopartita* Malacca

All graphics and text produced by the authors as they are listed under the title of this article (CC-BY open access license). Long horn beetle photographs taken by L. Dembicky.

Cerambycidae

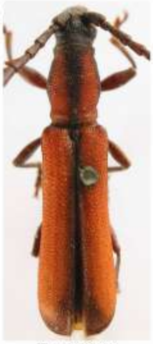

275 *Pyrestes virgata* Malaysia, Penang

Cerambycidae

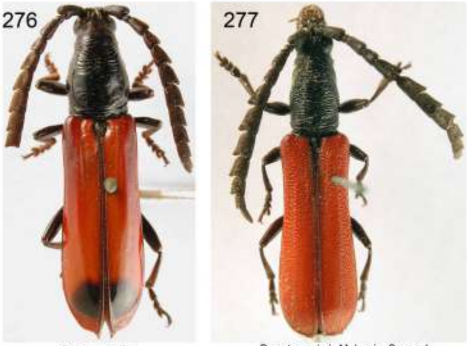

276 *Pyrestes polita* Malaysia, Penang

277 *Pyrestes eximia* Malaysia, Sarawak

Cerambycidae

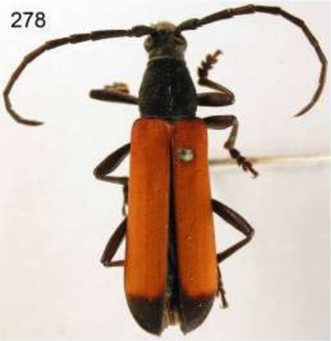

278 *Erythrus atricollis* Malaysia, Penang

Sumatra, the Malay Peninsula, Borneo

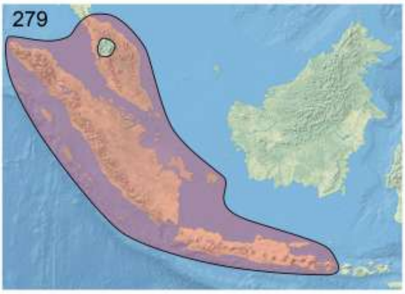

Cerambycidae

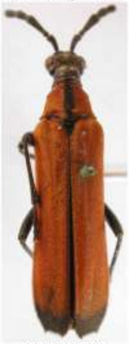

Ephies cruentus
Pascoe 1866 Penang

Cerambycidae

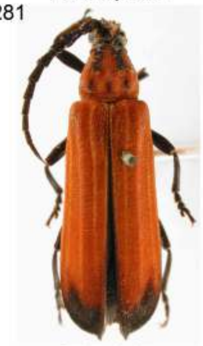

Erythrus apiculatus
Pascoe 1866 Penang

Cantharidae

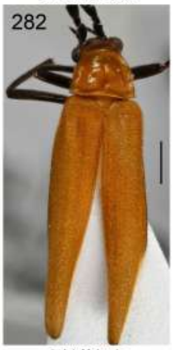

indet. Malaysia

Cerambycidae

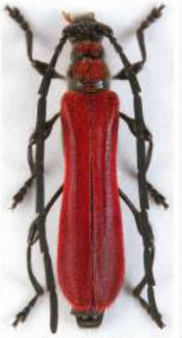

Astathes terminata
Breuning 1956 Malacca

Cerambycidae

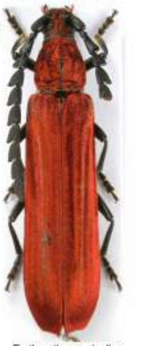

Erythresthes sericellus
Holzschuh 2005 Malaysia, Sabah

Cantharidae

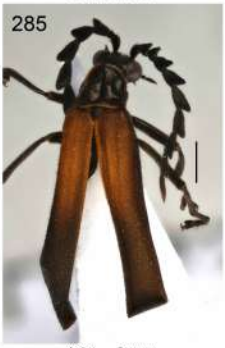

indet. Borneo

Cantharidae

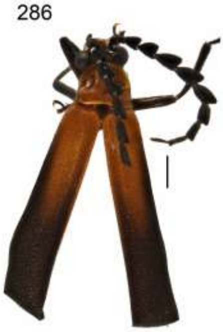

indet. Malaysia, Pahang

Cantharidae

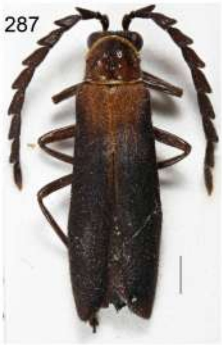

indet., Sumatra

Pyrochroidae

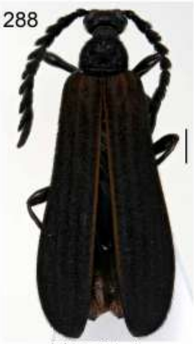

indet. sp. Malaysia
Cameron Highlands

Cantharidae

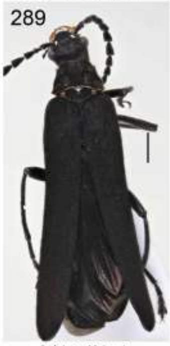

indet. sp. Malaysia
Cameron Highlands

All graphics and text produced by the authors as they are listed under the title of this article (CC-BY open acces license). Long horn beetle photographs taken by L. Dembicky.

290

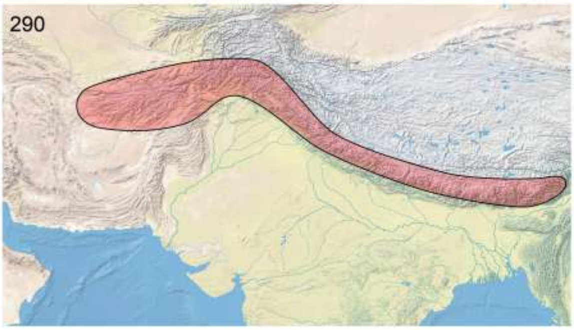

Cerambycidae

291

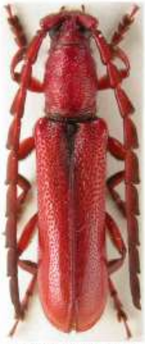

Pyrestes rufipes nepalicus Holzschuh, 1990, E Nepal

Cerambycidae

292

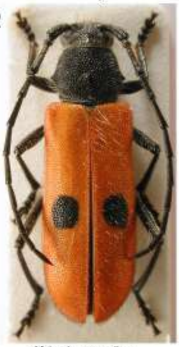

Afghanicenus aulicus, Pakistan

Cerambycidae

293

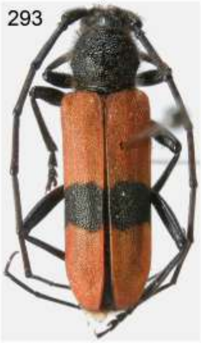

Purpuricenus nuristanicus, Afghanistan

Cerambycidae

294

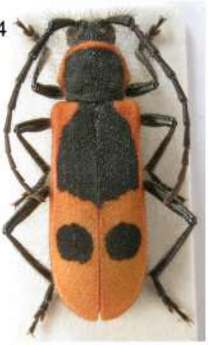

Calchaenesthes oblongomaculatus diversicollis, Iran

Cerambycidae

295

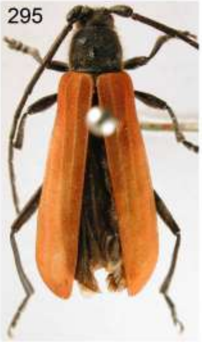

Brototyche adamsi, Chosan

Cerambycidae

296

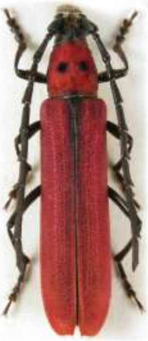

Erythrus suturellus Holzschuh, 1984, India

All graphics and text produced by the authors as they are listed under the title of this article (CC-BY open acces license). Long horn beetle photographs taken by L. Dembicky.

Afrotropical Region

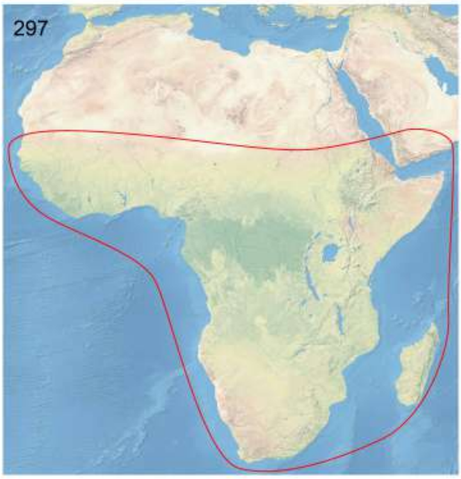

Continental Africa the and southern part of the Arabian Peninsula

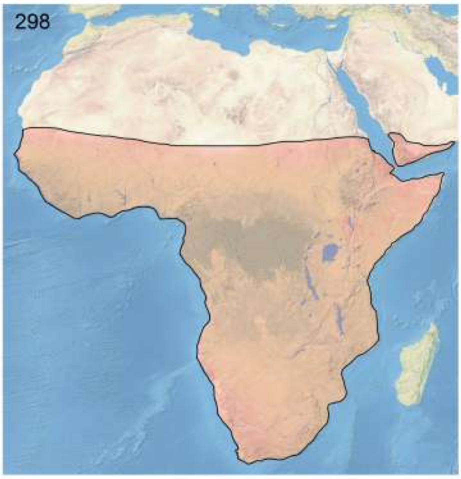

The phylogenetic structure of the Lycidae fauna

Subfamily	Tribe /Subtribe	# spp. (woldwide)	# spp. (region)
Dexorinae	Dexorini	15 spp.	15 spp.
	Mimolibnetini	5 spp.	5 spp.
Erotinae	Erotini	54 spp.	
	Dictyopterini	73 spp.	
	Taphini	31 spp.	
	Slipinskiini	46 spp.	~46 spp.
Calochrominae	Calochromini	289 spp.	~40 spp.
Ateliinae	Ateliini	45 spp.	
	Lyponiini	45 spp.	
	Macrolycini	69 spp.	
Lyropaeinae	Lyropaeini	43 spp.	
	Alyculini	5 spp.	
	Antennolycini	3 spp.	
	Platerodrilini	49 spp.	
Lycinae	Conderini	42 spp.	
	Eurrhacini	102 spp.	
	Thonalmini	11 spp.	
	Leptolycini	12 spp.	
	Platerodini	861 spp.	~ 80 spp.
	Calopterini	367 spp.	
	Lycini	413 spp.	>300 spp.
Metriorrhynchinae	Dihammatini	44 spp.	
	Lycoprogenthini	7 spp.	
	Libnetini	112 spp.	
	Dilophotini	81 spp.	
	Metriorrhynchini	1410 spp.	
	Metanoeina	40 spp.	
	Cautirina	750 spp.	~170 spp.
	Metriorrhynchina	620 spp.	
Total		4234 spp.	~660 spp.

The presence of net-winged beetle aposematic patterns in the region

Group	Colour type	Characteristics	+/-	Figures
Uniform coloration	Black	pronotum an elytra uniformly black to dark btown		
	Yellow	pronotum and elytra yellow to light brown	✓	371-377
	Orange and Red	pronotum and elytra brightly orange or cinnamon red		
	Metallic (blue, green)	pronotum and elytra metalic, all shades of colours		
Bi-colored pronotum/ elytra	black/yellow	pronotum back (at most with bright margins), elytra yellow	✓	365-368
	black/red	pronotum black (at most with bright margins), elytra red		
	bright/black	pronotum brightly colored, elytra uniformly black		
	red/merallic	pronotum brightly red, elytra metallic blue		
Bi-colored elytra	yellow/black	elytra bi-colored. humeral part yellow, apical part dark coloured	✓	300-312,310-326,331-337,339-341,343-363, etc.
	red(orange)/black	elytra bi-colored. humeral part orange/red, apical part dark		
	black/bright	elytra bi-colored. humeral part black, apex yellow/orange/red		
Fasciate elytra	yellow/black	humeri and apex of elytra black, middle of elytron yellow	✓	324-326
	bright/black	humeri and apex of elytra brigt, middle of elytron black	✓	313-314
	yellow/metallic	most elytra black with blue metallic shine, middle yellow		
Striate elytra	bright/black	elytra brown/brightly coloured, suture or middle of elytron black	✓	327-329, 338
Punctate el.	bright/black	elytra brown/brightly coloured, black patch in each elytron		
Tri-colored el.	all combinations of colors	elytron with three differently coloured parts		
Reticulate	bright/black	backgroud colour of elytra dark, costae large, brightly coloured		
Non-categorized aposematic patterns		see listed examples for further information		

Figures S297–S397. The overview of the lycid fauna, coloration, and co-mimics: Afrotropical region, Continental Africa, the southern part of the Arabian Peninsula, and Madagascar.

All graphics and text produced by the authors as they are listed under the title of this article (CC-BY open acces license). Long horn beetle photographs taken by L. Dembicky.

Sub-Saharan contineṭal Africa
S of the Arabian Peninsula

>300 spp. of Atrotropical *Lycus*

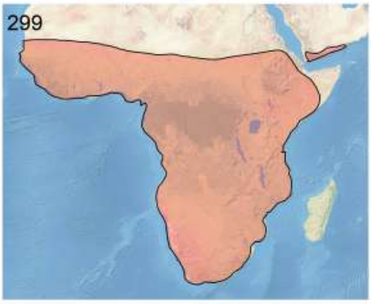

Lycinae: Lycini

Pattern: bi-coloured elytra
pronotum mostly with median black patch and brihtly coloured lateral margins
with/without scutellar patch and
black elytral apex

Body size: 15-22 mm

Distribution:
The eastern part of continental Africa, the southern part of the Arabian Peninsula

Non-lycid co-mimics:
Cerambycidae

Lycinae: Lycini

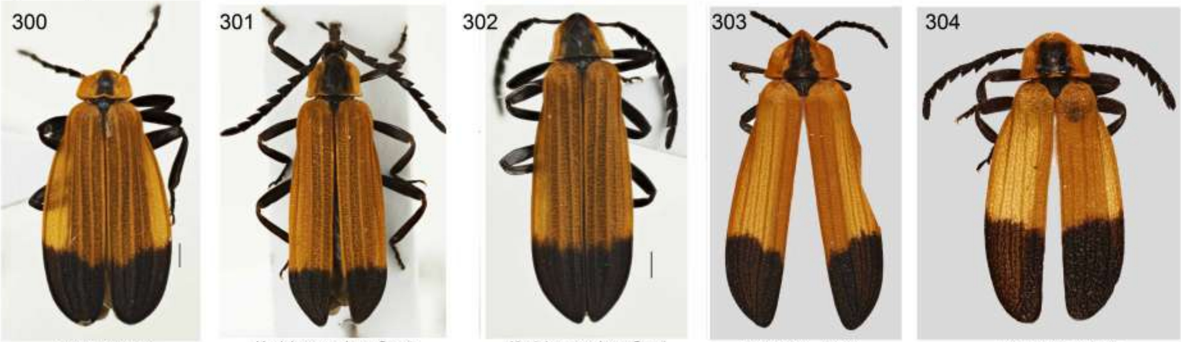

Lycus sp., Ivory Coast | *Haplolycus* sp. Ivory Coast | *Haplolycus* sp. Ivory Coast | *Haplolycus* sp., Namibia | *Haplolycus* sp., Tanzania

Lycinae: Lycini

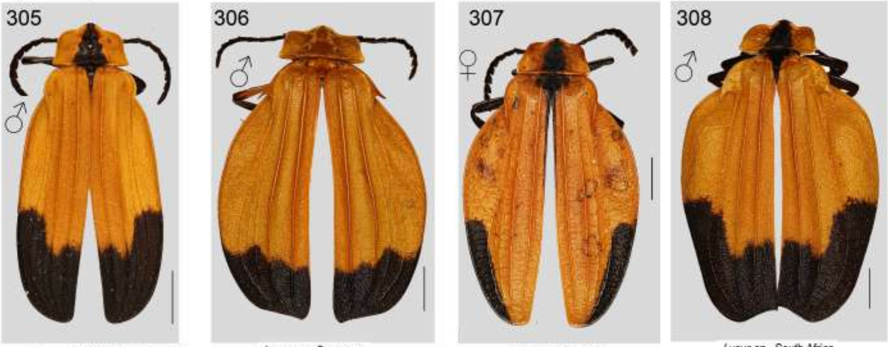

Lycus sp., South Africa | *Lycus* sp., Cameron | *Lycus* sp., Cameron | *Lycus* sp., South Africa

All graphics and text produced by the authors as they are listed under the title of this article (CC-BY open acces
li[illegible]ng horn beetle photographs taken by L. Dembicky.

Lycinae: Lycini

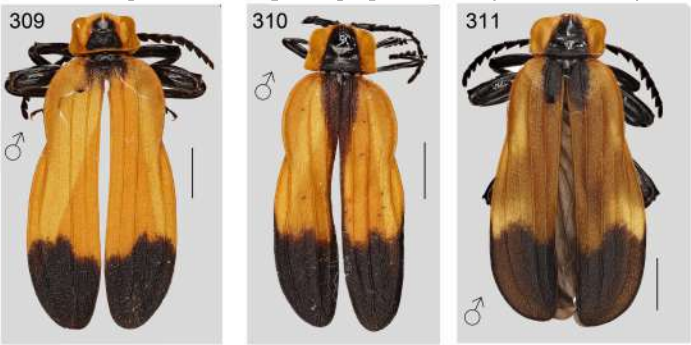

Lycus sp., Zambia | *Lycus* sp., Ethiopia | *Lycus* sp., South Africa

Lycinae: Lycini

hymeral thorns
as putative
antipredatory
adaptation

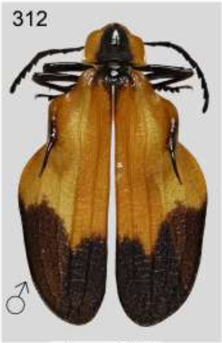

Lycus sp., Zambia

Lycinae: Lycini

Pattern: bi-coloured elytra
as above but patches form transverse black band

Body size: 15-22 mm

Distribution:
Sub-Saharan continental Africa

Non-lycid co-mimics:
Cerambycidae

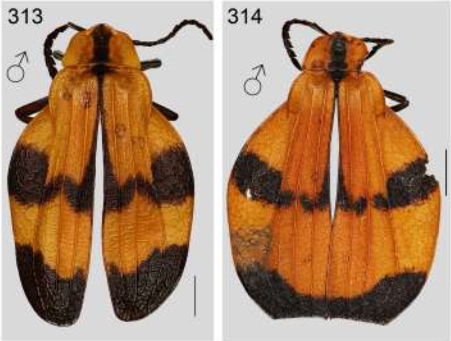

Lycus sp., Cameron | *Lycus* sp., Cameron

Sub-Saharan continetal Africa
S of the Arabian Peninsula

All Lycinae: Lycini

315

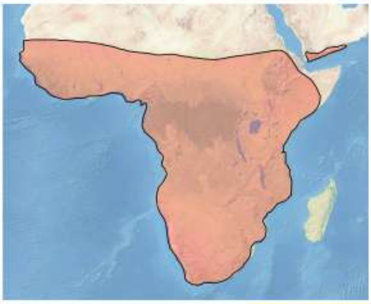

Pattern: bi-coloured elytra
with/without scutellar patch and
black elytral apex

Body size: 15-22 mm

Distribution:
The continental Sub-Saharan Africa and the southern part of the Arabian Peninsula

Non-lycid co-mimics:
Cerambycidae

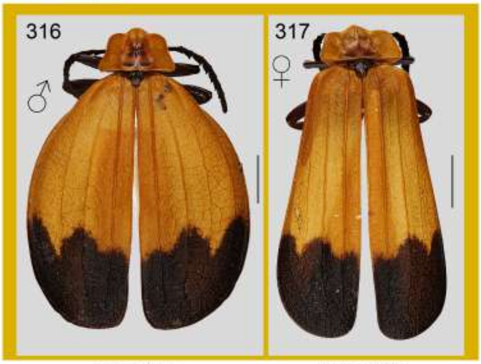

Lycus sp., Kenya   *Lycus* sp., Kenya

Sexual dimorphism
-males with dilated elytra
-females without modification
- multiple origins in the Lycini (Kusy et al., in press)

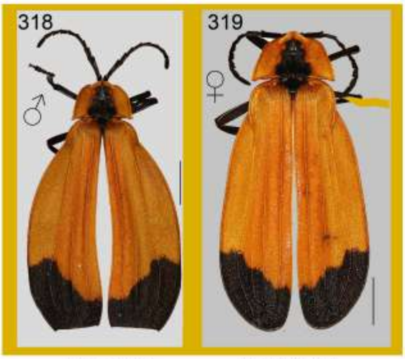

Lycus sp., Zambia   *Lycus* sp., Cameron

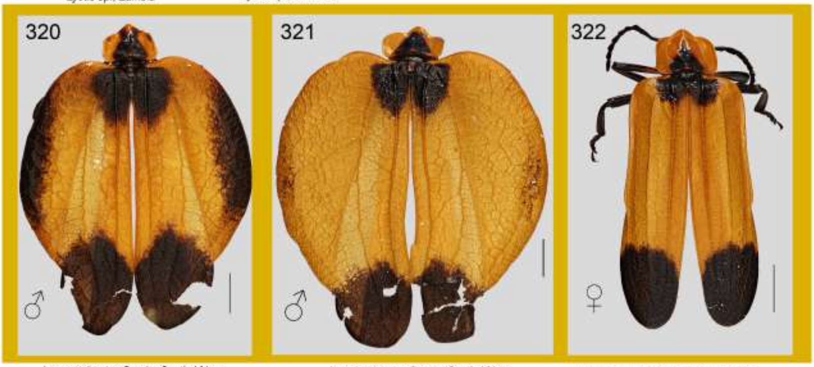

Lycus trabeatus Guerin, South Africa   *Lycus trabeatus* Guerin, South Africa   *Lycus trabeatus* Guerin, South Africa

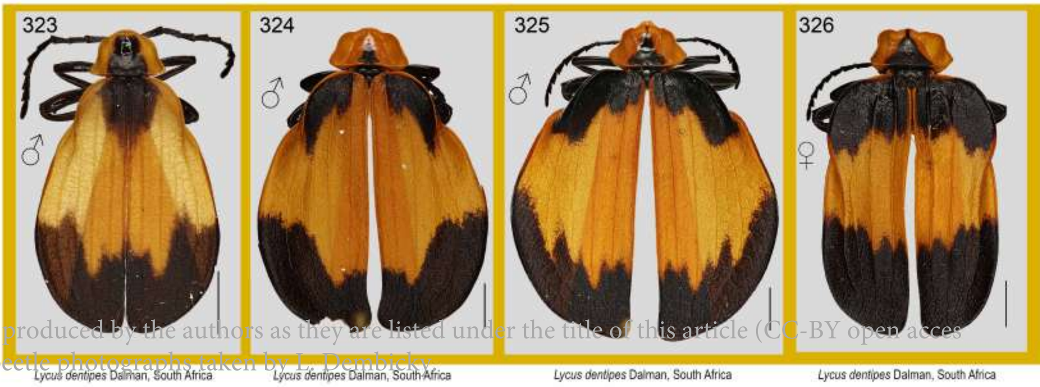

Lycus dentipes Dalman, South Africa   *Lycus dentipes* Dalman, South Africa   *Lycus dentipes* Dalman, South Africa   *Lycus dentipes* Dalman, South Africa

All graphics and text produced by the authors as they are listed under the title of this article (CC-BY open acces license). Long horn beetle photographs taken by L. Dembicky.

Pattern: stripped elytra

Body size: 12-15 mm

Distribution:
Sub-Saharan continental Africa

Non-lycid co-mimics:
Cerambycidae

Lycinae: Lycini

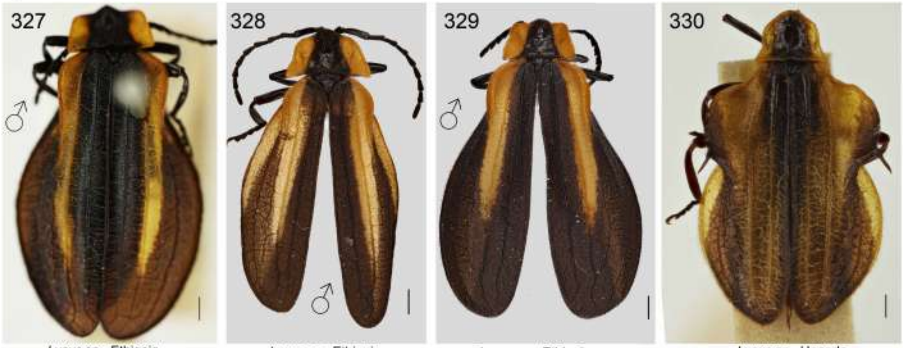

Lycus sp., Ethiopia — *Lycus* sp., Ethiopia — *Lycus* sp., Ethiopia — *Lycus* sp., Uganda

Pattern: bi-coloured elytra
both sexes with scutellar patch and black elytrl apex, some males with widened elytra

Body size: 1~13 mm

Distribution:
The eastern and southern part of continental Africa

Non-lycid co-mimics:
-

Lycinae: Lycini

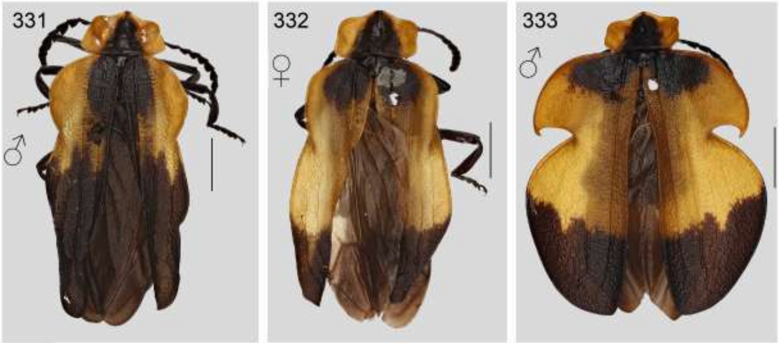

Lycus sp., Tanzania — *Lycus* sp., South Africa — *Lycus* sp., South Africa

Pattern: bi-coloured elytra
both sexes with scutellar patch and black elytrl apex, sometimes connected along the elytral suture

Body size: 10-12 mm

Distribution:
The eastern part of continental Africa

Non-lycid co-mimics:
-

Lycinae: Lycini

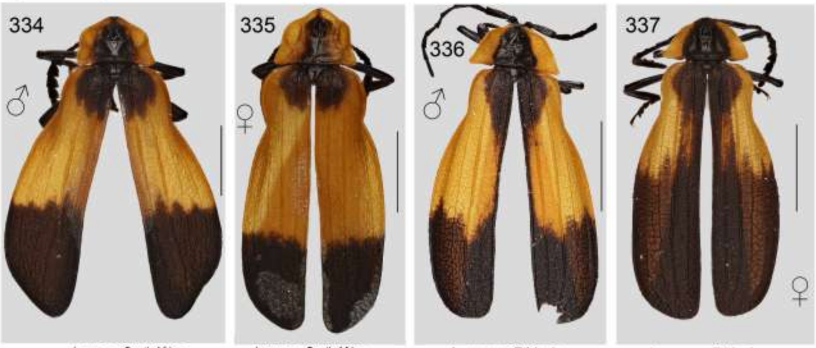

Lycus sp., South Africa — *Lycus* sp., South Africa — *Lycus* sp., Ethiopia — *Lycus* sp., Ethiopia

Pattern: bi-coloured elytra
the male with stripped elytra, female with scutellar patch and black elytrl apex

Body size: 12-15 mm

Distribution:
The eastern part of continental Africa

Non-lycid co-mimics:
-

sexual dimorphism

Lycinae: Lycini

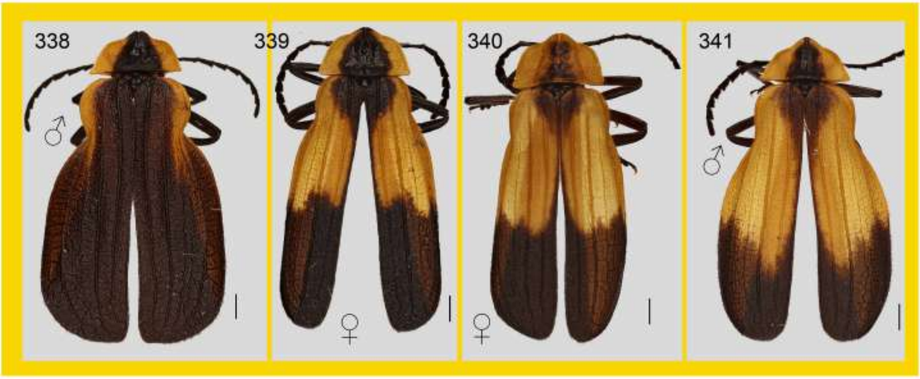

Lycinae: Lycini

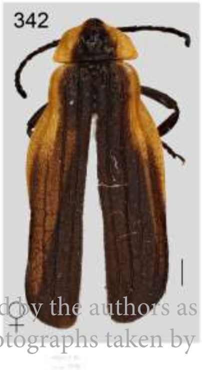

All graphics and text produced by the authors as they are listed under the title of this article (CC-BY open acces license). Long horn beetle photographs taken by L. Dembicky.

Sub-Saharan continetal Africa
S of the Arabian Peninsula

343

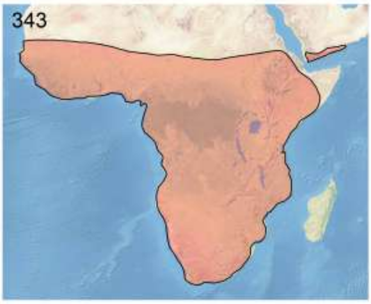

Pattern: Bi-coloured elytra
pronotum yellow to black
with light margins
Body size: ~ 8 mm

Distribution:
Sub-Saharan continental Africa
S of the Arabian Peninsula

Non_lycis co-mimics:
Cantharidae, Elateridae: Agrypninae: Drilini

Calochrominae: Calochromini

344

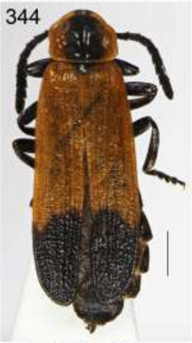

Micronychus sp., Namibia

345

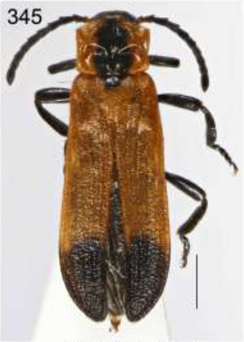

Micronychus sp., Namibia

Metriorrhynchinae: Metriorrhynchini: Cautirina

346

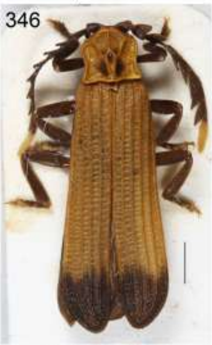

Cautires sp., Sierra Leone

347

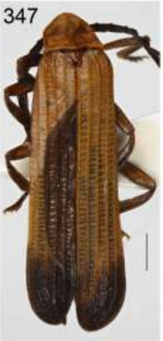

Cautires sp., Congo

Lycinae: Platerodini

348

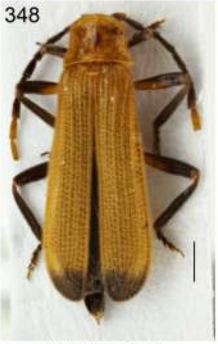

Plateros sp., Gabon

Metriorrhynchinae: Metriorrhynchini: Cautirina

349

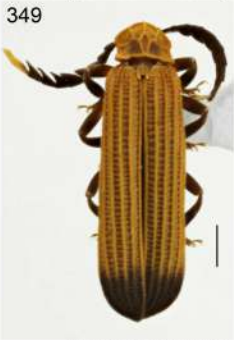

Cautires sp., Sierra Leone

350

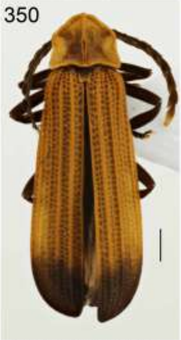

Cautires sp., Ivory Coast

351

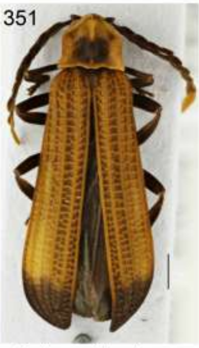

Cautires sp., Sierra Leone

Metriorrhynchinae: Metriorrhynchini: Cautirina

352

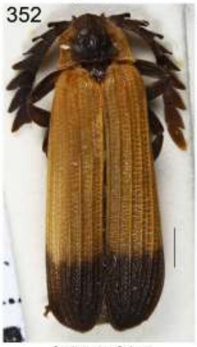

Cautires sp., Guinea

353

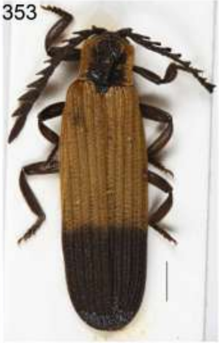

Cautires sp., Guinea

354

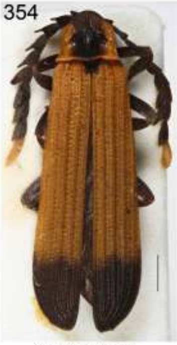

Cautires sp., Guinea

355

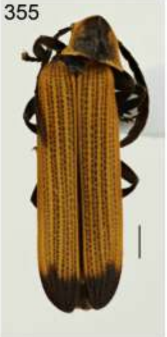

Cautires sp., Ivory Coast

356

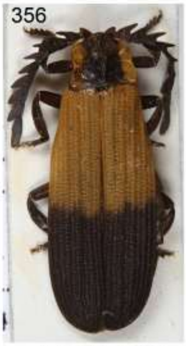

Cautires sp., Sierra Leone

357

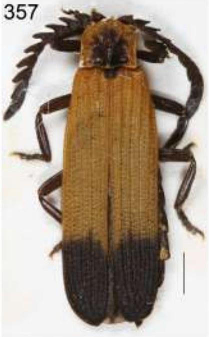

Cautires sp., Kenya

Metriorrhynchinae: Metriorrhynchini: Cautirina

358

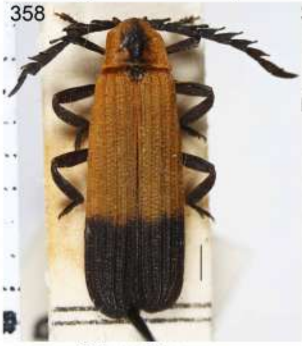

Cautires sp., Guinea

359

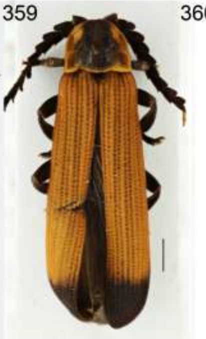

Cautires sp., Ivory Coast

360

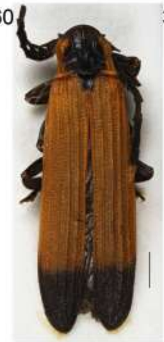

Cautires sp., Guinea

361

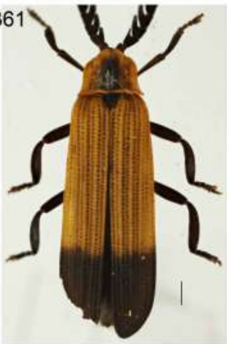

Cautires sp., Tanzania

Lycinae: Platerodini

362

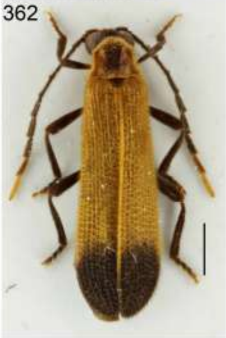

Plateros sp., Zambia

Pattern: Bi-coloured elytra
pronotum yellow
Body size: ~ 5 mm

Distribution:
Sub-Saharan continental Africa

Metriorrhynchinae: Metriorrhynchini: Cautirina

363

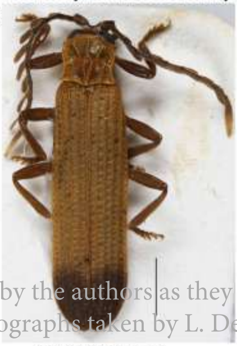

Cautires sp., Sierra Leone

All graphics and text produced by the authors as they are listed under the title of this article (CC-BY open acces license). Long horn beetle photographs taken by L. Dembicky.

Sub-Saharan continetal Africa

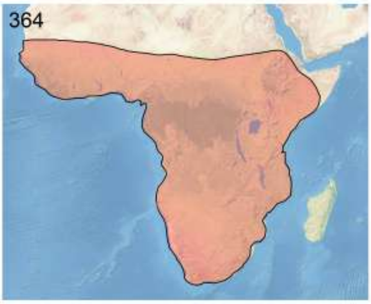

Pattern: Yellow elytra
pronotum black

Body size: ~ 6 mm

Distribution:
Sub-Saharan continental Africa

Non_lycis co-mimics:
Cantharidae

Lycinae: Platerodini

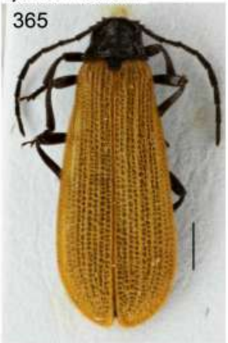

Plateros sp., Tanzania

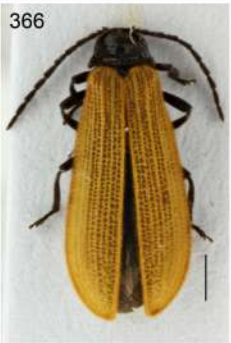

Plateros sp., Tanzania

Metriorrhynchinae: Metriorrhynchini: Cautirina

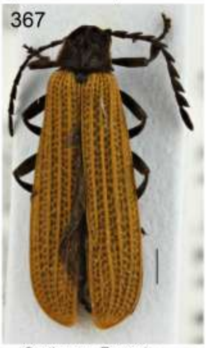

Cautires sp., Tanzania

Lycinae: Platerodini

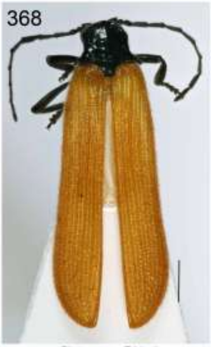

Plateros sp., Ethiopia

Pattern: Bi-coloured elytra
pronotum black

Body size: ~ 6 mm

Distribution:
Sub-Saharan continental Africa
S of the Arabian Peninsula

Non_lycis co-mimics:
Cantharidae, Elateridae:
Agrypninae: Drilini

Lycinae: Platerodini

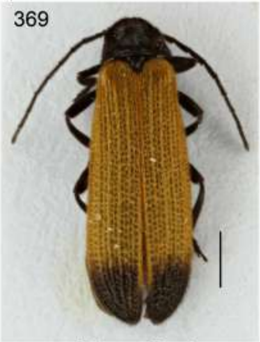

Plateros sp., Tanzania

Metriorrh.: Metriorrh.: Cautirina

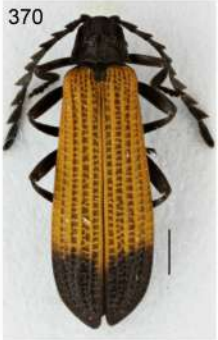

Cautires sp., Tanzania

Pattern: Uniform yellow

Body size: 5-8 mm

Distribution:
Sub-Saharan continental Africa

Lycinae: Platerodini

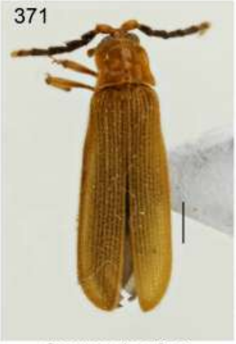

Plateros sp., Ivory Coast

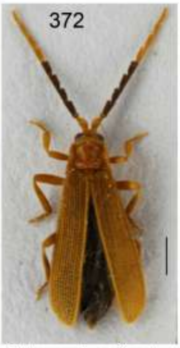

Plateros sp., Ivory Coast

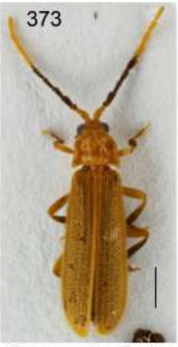

Plateros sp., Ivory Coast

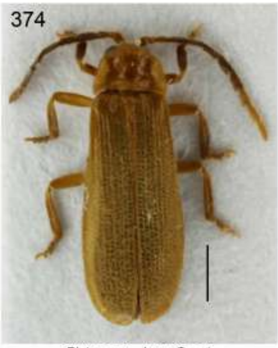

Plateros sp., Ivory Coast

Metriorrhynchinae: Metriorrhynchini: Cautirina

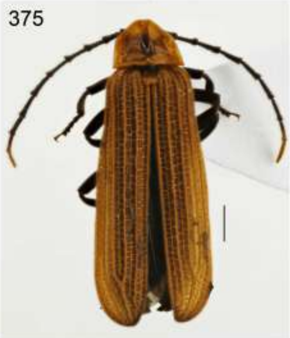

Cautires sp., Ivory Coast

Pattern: Uniform yellow

Body size: 3-5 mm

Distribution:
the central part
of the Sub-Saharan
continental Africa

Note: neotenic lineages, possibly a limited interaction with other Lycidae due to different biology

Dexorinae: Dexorini

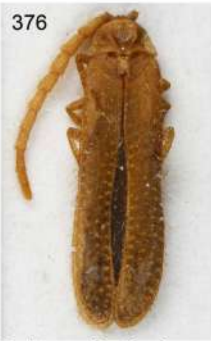

Dexoris tessmanni Boc. et Boc., Cameroon

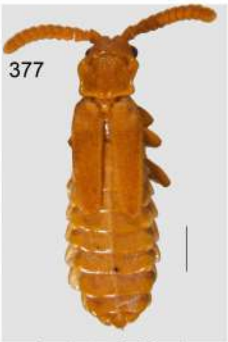

Dexoris apterus Boc., Tanzania

All graphics and text produced by the authors as they are listed under the title of this article (CC-BY open acces license). Long horn beetle photographs taken by L. Dembicky.

Sub-Saharan Africa (part)

Pattern: Bi-coloured elytra, black part over one third
pronotum yellow

Body size: ~ 8 mm

Distribution:
Sub-Saharan continental Africa (east and central)

Non_lycis co-mimics:
Cantharidae, Elateridae: Agrypninae: Drilini

Erotinae: Slipinskiina

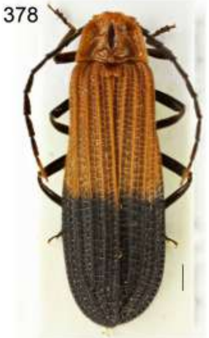

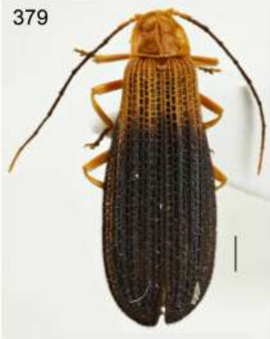

sp., Tanzania

Metriorrhynchinae: Metriorrhynchini: Cautirina

Pattern:
as previous,
smaller light
part of elytra

Body size:
~ 10 mm

Distribution:
Tanzania

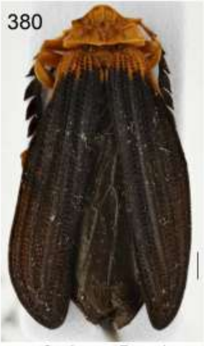

Cautires sp., Tanzania

Afrotropical Region

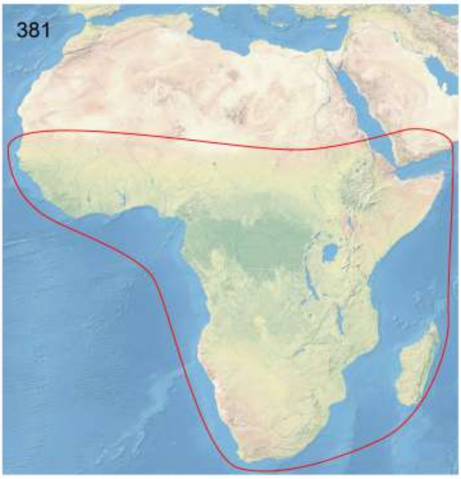

Madagascar

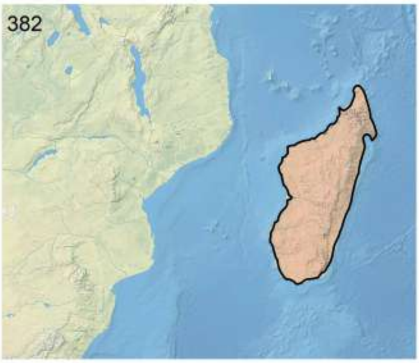

The phylogenetic structure of the fauna

Subfamily	Tribe /Subtribe	# spp. (woldwide)	# spp. (Madag.)
Dexorinae	Dexorini	15 spp.	
	Mimolibnetini	5 spp.	
Erotinae	Erotini	54 spp.	
	Dictyopterini	73 spp.	
	Taphini	31 spp.	
	Slipinskiini	46 spp.	
Calochrominae	Calochromini	289 spp.	
Ateliinae	Ateliini	45 spp.	
	Lyponiini	45 spp.	
	Macrolycini	69 spp.	
Lyropaeinae	Lyropaeini	43 spp.	
	Alyculini	5 spp.	
	Antennolycini	3 spp.	
	Platerodrilini	49 spp.	
Lycinae	Conderini	42 spp.	
	Eurrhacini	102 spp.	
	Thonalmini	11 spp.	
	Leptolycini	12 spp.	
	Platerodini	861 spp.	
	Calopterini	367 spp.	
	Lycini	413 spp.	
Metriorrhynchinae	Dihammatini	44 spp.	
	Lycoprogenthini	7 spp.	
	Libnetini	112 spp.	
	Dilophotini	81 spp.	
	Metriorrhynchini	1410 spp.	
	Metanoeina	40 spp.	
	Cautirina	750 spp.	40 spp.
	Metriorrhynchina	620 spp.	
Total		4234 spp.	40 spp.

The presence of net-winged beetle aposematic patterns in the region

Group	Colour type	Characteristics	+/-	Figures
Uniform coloration	black	pronotum an elytra uniformly black to dark brown	✓	384-386
	yellow	pronotum and elytra yellow to light brown		
	orange and red	pronotum and elytra brightly orange or cinnamon red		
	metallic (blue, green)	pronotum and elytra metalic, all shades of colours		
Bi-colored pronotum/ elytra	black/yellow	pronotum black (at most with bright margins), elytra yellow		
	black/red	pronotum black (at most with bright margins), elytra red		
	bright/black	pronotum brightly colored, elytra uniformly black	✓	387-390
	red/merallic	pronotum brightly red, elytra metallic blue		
Bi-colored elytra	yellow/black	elytra bi-colored. humeral part yellow, apical part dark coloured		
	red(orange)/black	elytra bi-colored. humeral part orange/red, apical part dark		
	black/bright	elytra bi-colored. humeral part black, apex yellow/orange/red		
Fasciate elytra	yellow/black	humeri and apex of elytra black, middle of elytron yellow		
	bright/black	humeri and apex of elytra brigt, middle of elytron black		
	yellow/metallic	most elytra black with blue metallic shine, middle yellow		
Striate elytra	bright/black	elytra brown/brightly coloured, suture or middle of elytron black		
Punctate el.	bright/black	elytra brown/brightly coloured, black patch in each elytron		
Tri-colored el.	all combinations of colors	elytron with three differently coloured parts		
Reticulate	bright/black	backgroud colour of elytra dark, costae large, brightly coloured		
Non-categorized aposematic patterns		see listed examples for further information		

All graphics and text produced by the authors as they are listed under the title of this article (CC-BY open acces license). Long horn beetle photographs taken by L. Dembicky.

Madagascar

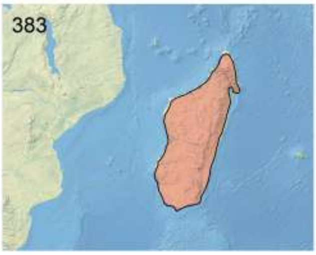

Phylogenetic structure of the fauna

Metriorrhynchinae: Metriorrhynchini: Metriorrhynchina

2 genera: *Cautires* Waterhouse ~30 spp.
Caenioxylobanus xxxxx 1 sp.

Cautires spp. represent the terminal monophylum (Sklenarova et al., 2013)

Aposematic patterns

a) uniform black
b) pronotum red or with red lateral margins, elytra uniform black

Metriorrhynchinae: Metriorrhynchini: Cautirina

Uniform black

Madagascar
Co-mimics:
Cautires,
Caenioxylobanus

Non-lycid co-mimics:
Cantharidae

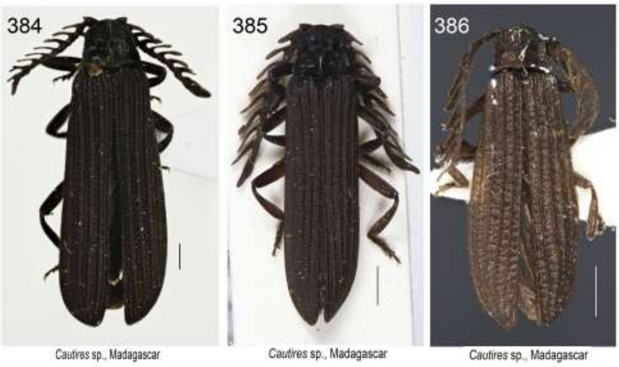

Cautires sp., Madagascar — *Cautires* sp., Madagascar — *Cautires* sp., Madagascar

Bicolored pronotum,
elytra black

Madagascar
Co-mimics:
Cautires,

Non-lycid co-mimics:
not recorded

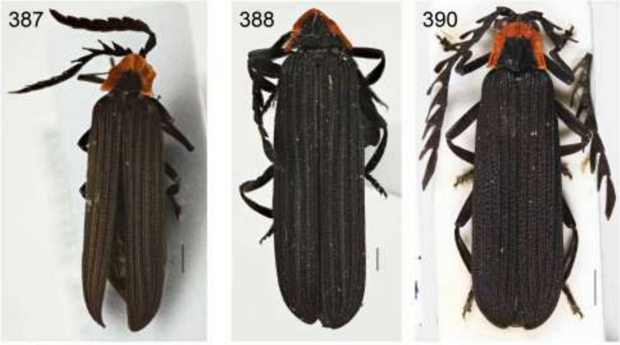

Cautires sp., Madagascar — *Cautires* sp., Madagascar — *Cautires* sp., Madagascar

All graphics and text produced by the authors as they are listed under the title of this article (CC-BY open acces license). Long horn beetle photographs taken by L. Dembicky.

Sub-Saharan continetal Africa

391

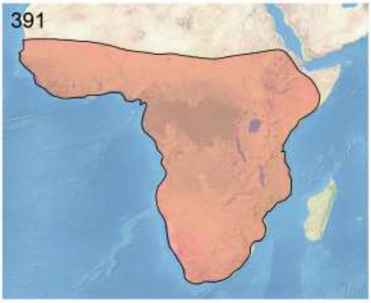

Cleridae

392

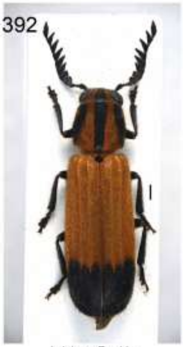

indet. sp. Zambia

Cantharidae

393

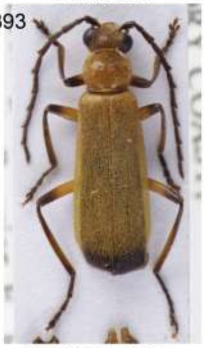

Silidius ifanensis, Ivory Coast

Chrysomelidae

394

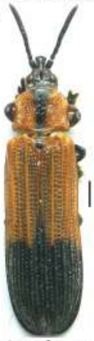

indet. sp. Cameroon

Cantharidae

395

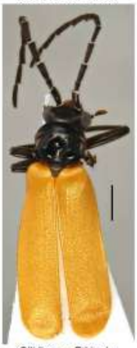

Silidius sp. Ethiopia

Cerambycidae

396

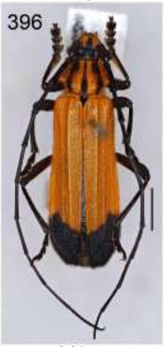

indet. sp.
Republic of South Africa

Cerambycidae

397

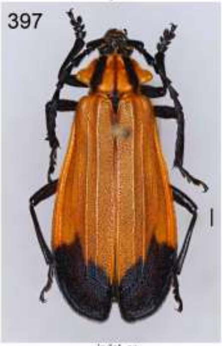

indet. sp.
Republic of South Africa

All graphics and text produced by the authors as they are listed under the title of this article (CC-BY open acces license). Long horn beetle photographs taken by L. Dembicky. The Hispa sp. photograph taken by A. Coache.

Palearctic Region

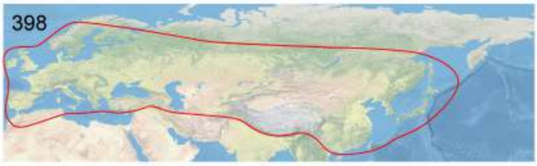

Eastern Asia , Sachalin, Japanese Islands,Taiwan and Hainan

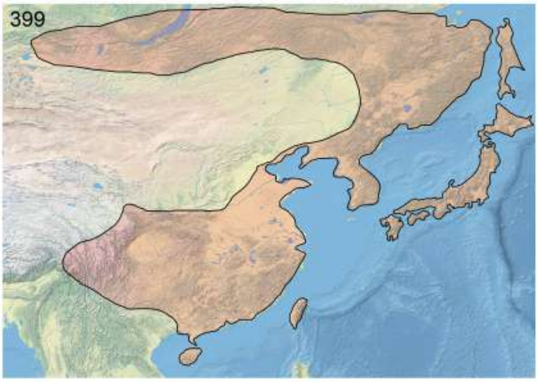

Phylogenetic structure of the fauna

Subfamily	Tribe /Subtribe	# spp. (woldwide)	# spp. (region)
Dexorinae	Dexorini	15 spp.	
	Mimolibnetini	5 spp.	
Erotinae	Erotini	54 spp.	25 spp.
	Dictyopterini	73 spp.	40 spp.
	Taphini	31 spp.	2 spp.
	Slipinskiini	46 spp.	
Calochrominae	Calochromini	289 spp.	
Ateliinae	Ateliini	45 spp.	
	Lyponiini	45 spp.	37 spp.
	Macrolycini	69 spp.	65 spp.
Lyropaeinae	Lyropaeini	43 spp.	1 spp.
	Alyculini	5 spp.	
	Antennolycini	3 spp.	
	Platerodrilini	49 spp.	2 spp.
Lycinae	Conderini	42 spp.	15 spp.
	Eurrhacini	102 spp.	
	Thonalmini	11 spp.	
	Leptolycini	12 spp.	
	Platerodini	861 spp.	~60 spp.
	Calopterini	367 spp.	
	Lycini	413 spp.	15 spp.
Metriorrhynchinae	Dihammatini	44 spp.	10 spp.
	Lycoprogenthini	7 spp.	1 sp.
	Libnetini	112 spp.	20 spp.
	Dilophotini	81 spp.	25 spp.
	Metriorrhynchini	1410 spp.	
	Metanoeina	40 spp.	10 spp.
	Cautirina	750 spp.	30 spp.
	Metriorrhynchina	620 spp.	3 spp.
Total		4234 spp.	~360 spp.

The presence of net-winged beetle aposematic patterns in the region

Group	Colour type	Characteristics	+/-	Figures
Uniform coloration	black	pronotum an elytra uniformly black to dark btown	✓	420, 421, 430-436
	yellow	pronotum and elytra yellow to light brown	✓	445
	orange and red	pronotum and elytra brightly orange or cinnamon red	✓	400-406 (bright), 407-417, 423 (dark red)
	metallic (blue, green)	pronotum and elytra metalic, all shades of colours		
Bi-colored pronotum/ elytra	black/yellow	pronotum back (at most with bright margins), elytra yellow	✓	441
	black/red	pronotum black (at most with bright margins), elytra red	✓	418, 438
	bright/black	pronotum brightly colored, elytra uniformly black	✓	444
	red/merallic	pronotum brightly red, elytra metallic blue		
Bi-colored elytra	yellow/black	elytra bi-colored. humeral part yellow, apical part dark coloured		
	red(orange)/black	elytra bi-colored. humeral part orange/red, apical part dark		
	black/bright	elytra bi-colored. humeral part black, apex yellow/orange/red		
Fasciate elytra	yellow/black	humeri and apex of elytra black, middle of elytron yellow		
	bright/black	humeri and apex of elytra brigt, middle of elytron black		
	yellow/metallic	most elytra black with blue metallic shine, middle yellow		
Striate elytra	bright/black	elytra brown/brightly coloured, suture or middle of elytron black	✓	450-452, 456
Punctate el.	bright/black	elytra brown/brightly coloured, black patch in each elytron		
Tri-colored el.	all combinations of colors	elytron with three differently coloured parts		
Reticulate	bright/black	backgroud colour of elytra dark, costae large, brightly coloured		
Non-categorized aposematic patterns		see listed examples for further information		

Figures S398–S484. The overview of the lycid fauna, coloration, and co-mimics: Palearctic region, Eastern Asia, Sachalin, Japanese Islands, and Hainan.

All graphics and text produced by the authors as they are listed under the title of this article (CC-BY open acces license). Long horn beetle photographs taken by L. Dembicky.

Pattern: uniform bright red

Body size: 5-8 mm

Distribution: Eastern Palearctic region, especially mountains of Yunan, Sichuan and some species in Japan

Further Lycidae co-mimics: none

Non-lycid co-mimics:
Cantharidae, Elateridae: Dendrometrini

Remark: widespread pattern known from the Palearctic and Nearctic regions.

Erotinae: Dictyopterini

400
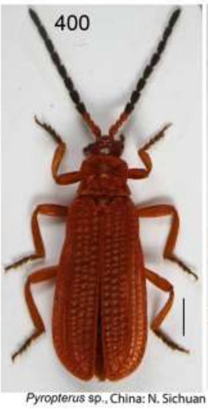
Pyropterus sp., China: N. Sichuan

401
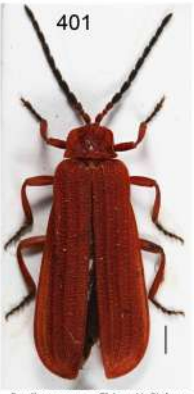
Benibotarus sp., China: N. Sichuan

402
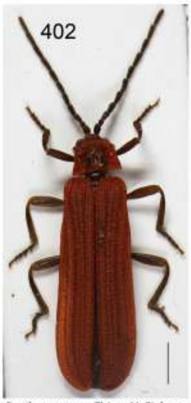
Benibotarus sp., China: N. Sichuan

Erotinae: Dictyopterini

403
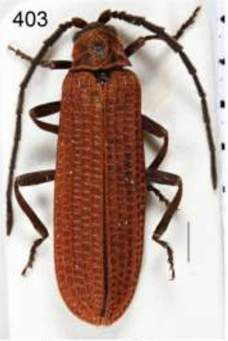
Pyropterus sp., Yunnan

404
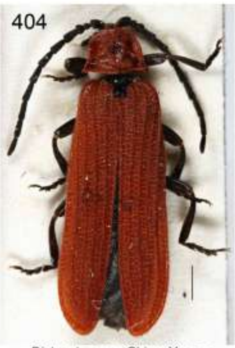
Dictyoptera sp., China: Yunnan

Ateliinae: Ateliini

405
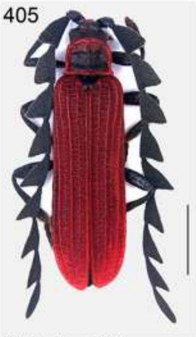
Atelius brevicornis Li , Yunnan

Pattern: uniform orange
Body size: 8-14 mm

Distribution: Himalayas and the eastern slopes of the Tibet Plateau

Further Lycidae co-mimics

Non-lycid co-mimics:
Cantharidae

Ateliinae: Macrolycini

406
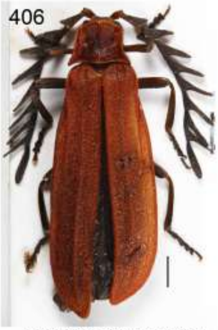
Macrolycus sp., China: N. Sichuan

Pattern: uniform dark red
Body size: 7-16 mm

Distribution: mountains of Sichuan, Yunnan and Gansu

Non-lycid co-mimics:
Cantharidae

Metriorrhynchinae: Dilophotini

407
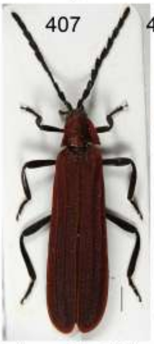
Dilophotes sp., China: N. Sichuan

408
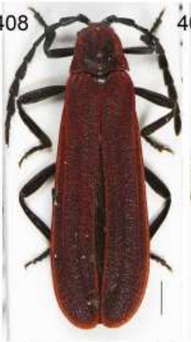
Dilophotes sp., China: N. Sichuai

Calochrominae: Calochromini

409
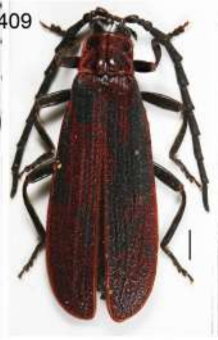
Parantis sp., China: N. Sichuan

410
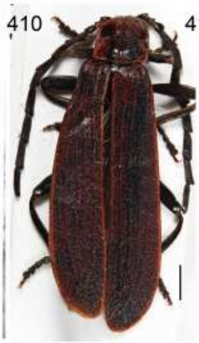
Parantis sp., China: N. Sichuan

M.:M.:Cautirina

411
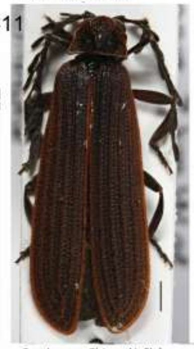
Cautires sp., China: N. Sichuan

412
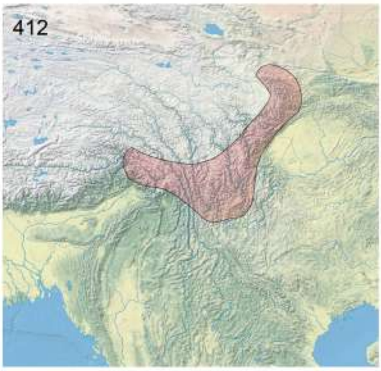

Ateliinae: Macrolycini

413
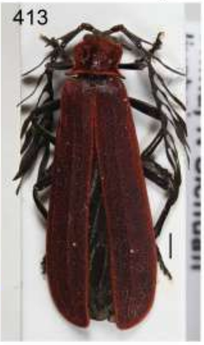
Macrolycus sp., China: N. Sichuan

414
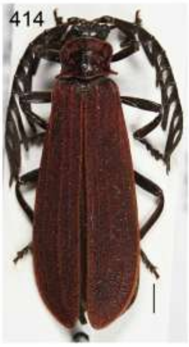
Macrolycus sp., China: N. Sichuan

Lycinae: Lycini

415
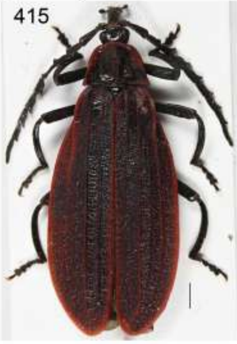
Lycostomus sp., China: N. Sichuan

M.:M.:Cautirina

416
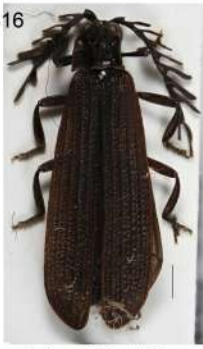
Cautires sp., China: N. Sichuan

All graphics and text produced by the authors as they are listed under the title of this article (CC-BY open acces license). Long horn beetle photographs taken by L. Dembicky.

417
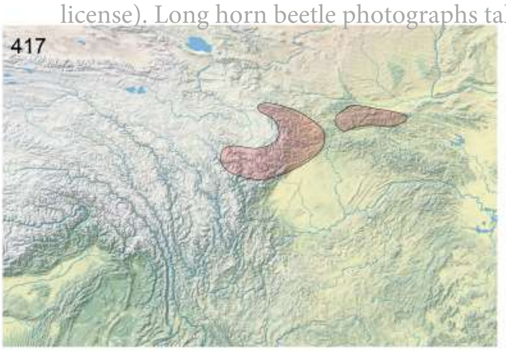

Pattern: black pronotum. red elytra with black elytral suture

Body size: 9-12 mm

Distribution: mountains of Shaanxi (Qinling mts), Gansu, northern Sichuan

Non-lycid co-mimics:
Cantharidae, Elateridae: Denticollini

Remark: a single species of Lycidae

Ateliinae: Lyponiini

418
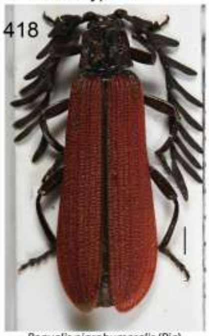
Ponyalis nigrohumeralis (Pic), China: N. Sichuan

Japan

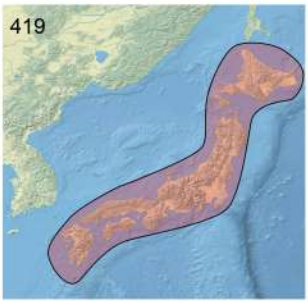

Pattern: dark red to black elytra;
the pronotum black or dark
red coloured, the pronotal margins
lighter coloured than the disc

Body size: 8-11 mm

Distribution: Japan

Non-lycid co-mimics:
Pyrochroidae

Remark. The medium-sized lycids
common on leaves under
the forest canopy, the dominant pattern
in the mid summer in Japan

Metriorrhynchinae: Metriorrhynchini: Cautirina

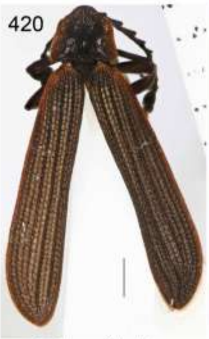

Cautires sp., China: Jiangsu

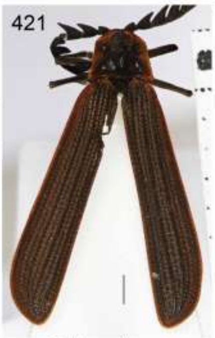

Cautires sp., Japan

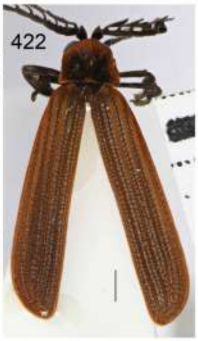

Cautires sp., Japan

Pattern: dark red elytra;
the pronotum black or dark
brown
Body size: 8-12 mm

Distribution: Japan

Non-lycid co-mimics:
Pyrochroidae, Cantharidae

Remark. The common lycids
sometimes visiting flowers. Several
species known from Japan.

Lycinae: Lycini

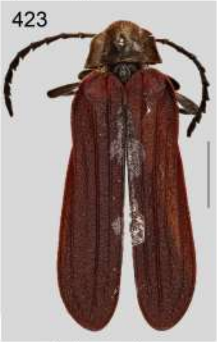

Lycostomus sp., Japan

Southern China

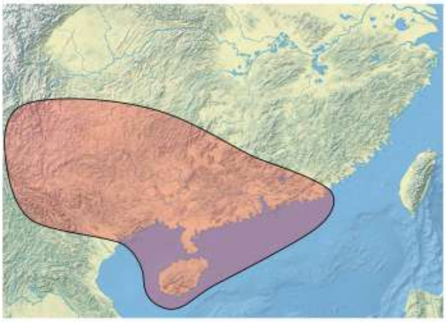

Pattern: dark red to cinnamon brown elytra;
the pronotum black with the pronotal margins
lighter coloured than the disc

Body size: 6-11 mm

Distribution: south eastern China

Non-lycid co-mimics: not recorded

Remark. The medium-sized lycids
common on leaves under
the forest canopy, the dominant pattern
in the mid summer season

Ateliinae: Ateliini

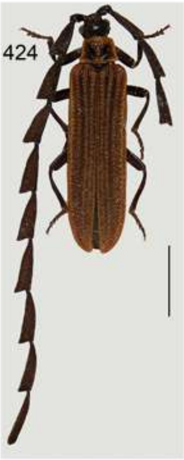

Atelius wittmeri Boc., China: Hainan

Metriorrh.:Metriorrh.: Cautirina

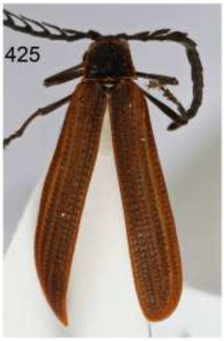

Cautires sp., China: Guangxi

A reddish form of the preceeding
pattern. *Benibotarus* belong to the
Dictyopterini whose species are
commonly brightly red coloured.
The similarly coloured *Benibotarus*
occurs also in Japan,

Erotinae: Dictyopterini

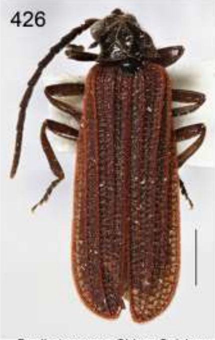

Benibotarus sp., China: Guizhou

Erotinae: Dictyopterini

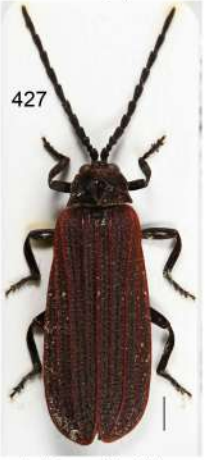

Benibotarus sp., China: Sichuan

A lowland pattern from southern
Yunnan. The shade resembles
the patterns known from northern
Laos. See the relative species of
Atelius from the high mountains
of Yunnan shown in the preceeding
table.

Ateliinae: Ateliini

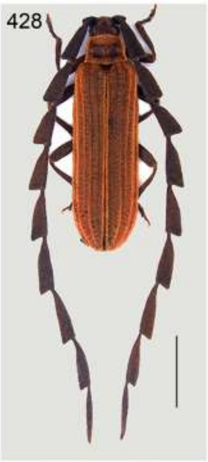

Atelius kadoorieorum Li, Yunnan

All graphics and text produced by the authors as they are listed under the title of this article (CC-BY open acces license). Long horn beetle photographs taken by L. Dembicky.

China, Korea, Russian Far East, Japan

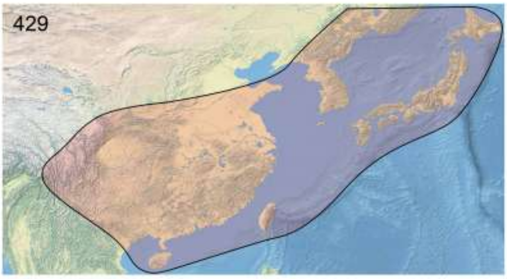

Pattern: uniform black

Body size: ~ 8 mm

Distribution: Japan, Korea, China

Non-lycid co-mimics:
Cantharidae

Remark. Small-bodied species common on leaves under canopy. A widespread pattern in the whole region

Lycinae: Platerodini

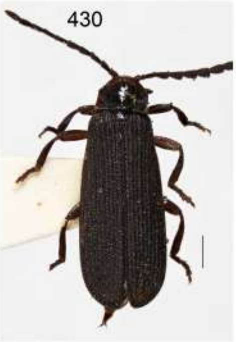

Plateros coracinus Kies., Japan

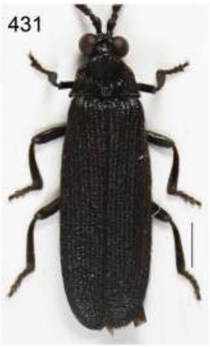

Plateros sp., China: Sichuan

Erotinae: Erotini

Metriorrhynchinae: Libnetini

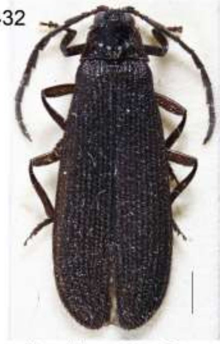

Plateros kleineanus Nakane, Taiwan

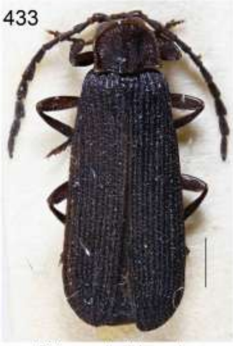

Plateros yaku Nakane, Japan

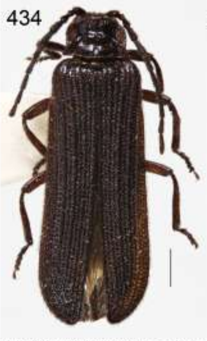

Eropterus taiwanus Mats., Taiwan

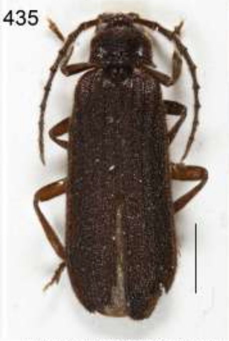

Libnetis fodingshanensis Boc., Shaanxi

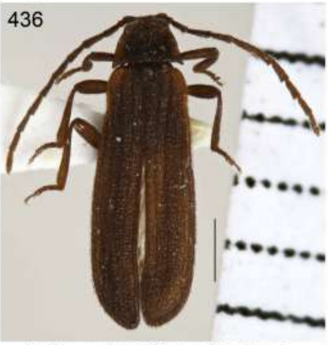

Libnetisia xunyanbanensis Bocakova, China, Shaanxi

All graphics and text produced by the authors as they are listed under the title of this article (CC-BY open acces license). Long horn beetle photographs taken by L. Dembicky.

Rjukju Islands, Taiwan

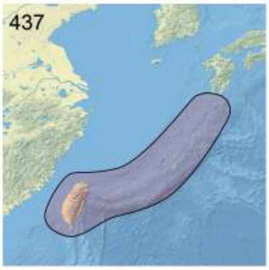

Pattern: red elytra; the pronotum black or with median patch

Body size: ~ 8 mm

Distribution: Rjukju Islands, Taiwan

Further Lycidae co-mimics:
Macrolycus spp.

Non-lycid co-mimics:
Cantharidae, Elateridae

Metriorrhynchinae: Metriorrhynchini: Cautirina

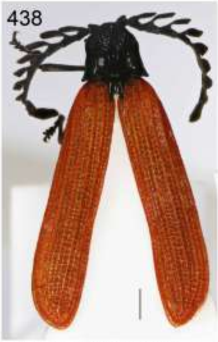

Cautires ishigakianus Nakane, Rjukju Isl.

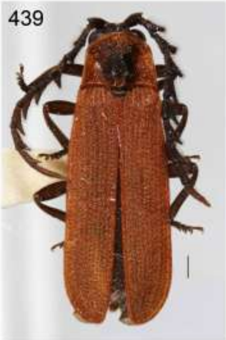

Plateros miwai Sato & Ohbayashi, Rjukju

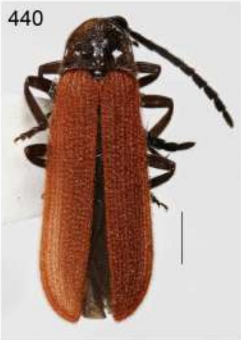

Plateros sp., Ishigaki

A light coloured form of the preceeding pattern

Metriorrh.: Mettriorrh.: Cautirina

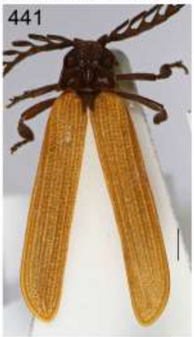

Cautires sp., Rjukju Isl. Okinawa

Pattern: dark elytra and the pronotum

Body size: ~ 8 mm

Distribution: Yunnan, Sichuan Guizhou

Non-lycid co-mimics:
Cantharidae

Lycinae: Platerodini

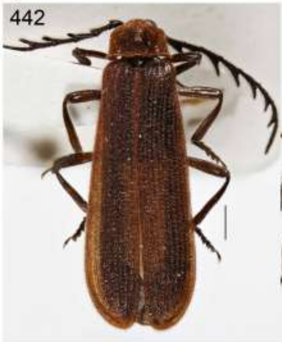

Plateros sp., Yunnan

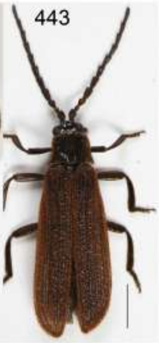

Plateros sp., Yunnan

Pattern: black elytra pronotum with a black median patch and light orange margins
Body size: ~ 8 mm

Distribution: Taiwan, southeastern China
Further Lycidae co-mimics

Non-lycid co-mimics:
Cantharidae, Elateridae

Remark. The pattern known also from northern Vietnam

Lycinae: Platerodini

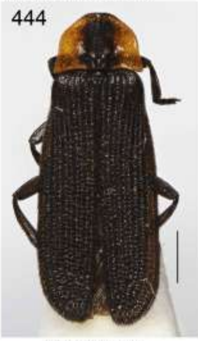

Plateros sp., Taiwan

Pattern: uellow elytra and the pronotum

Body size: ~ 5 mm

Distribution: Yunnan

Non-lycid co-mimics:
Cantharidae

Remark. A pattern known from the low elevations of Yunnan and northern Indo-Burma

Metriorrhynchinae: Libnetini

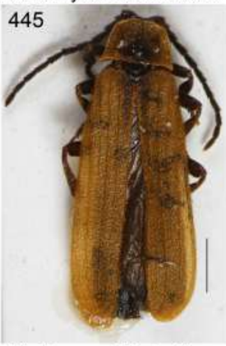

Libnetisia yunnanensis Bocakova, Yunnan

Lycinae: Lycini

Continental China, Taiwan island, Korea

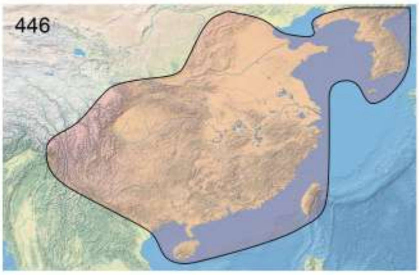

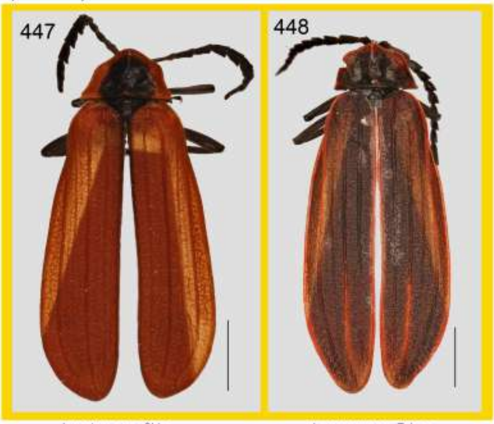

intraspecific polymorphism
geographically defined

Lycostomus sp., China

Lycospomus sp., Taiwan

Lycinae: Lycini

Continental China and Taiwan Island

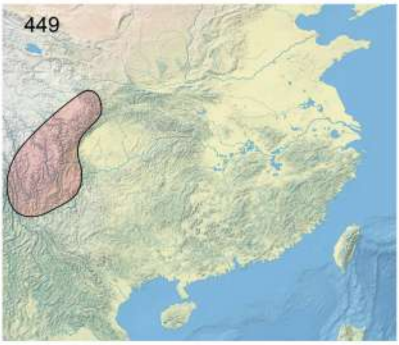

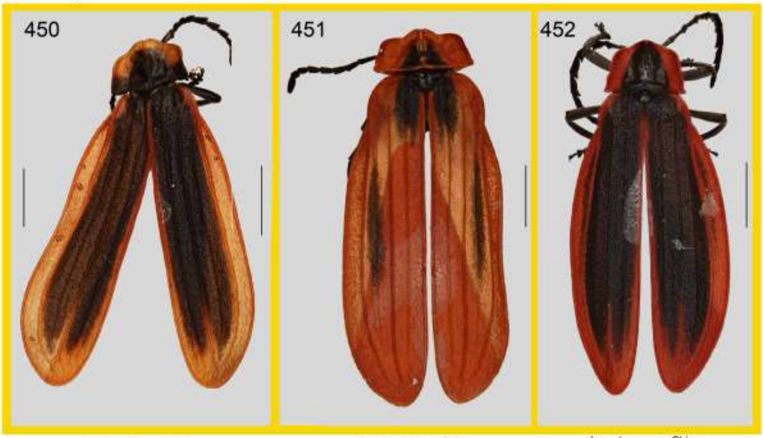

intraspecific polymorphism
altitudinally defined

Lycostomus sp., China

Lycostomus sp., China

Lycostomus sp., China

Eastern Himalayas and eastern slopes of theTibet Plateau (Yunnan and Sichuan)

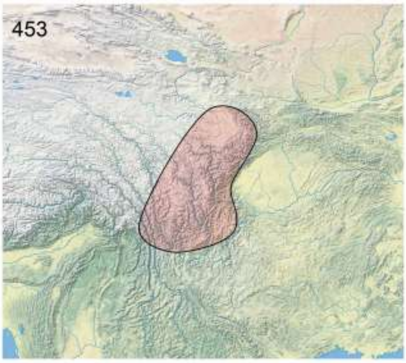

Lycinae: Lycini

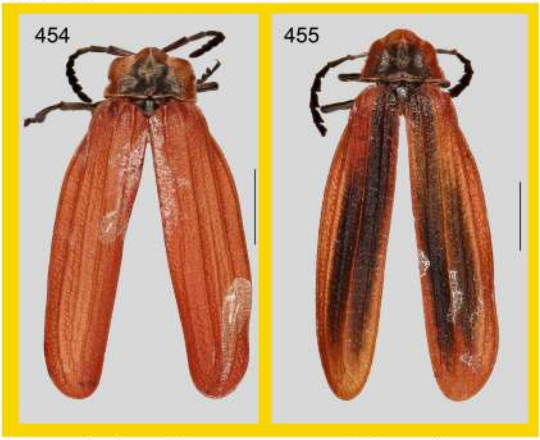

intraspecific polymorphism
altitudinally defined

Lycostomus sp., China

Lycostomus sp., China

All graphics and text produced by the authors as they are listed under the title of this article (CC-BY open acces license). Long horn beetle photographs taken by L. Dembicky.

Lycinae: Lycini

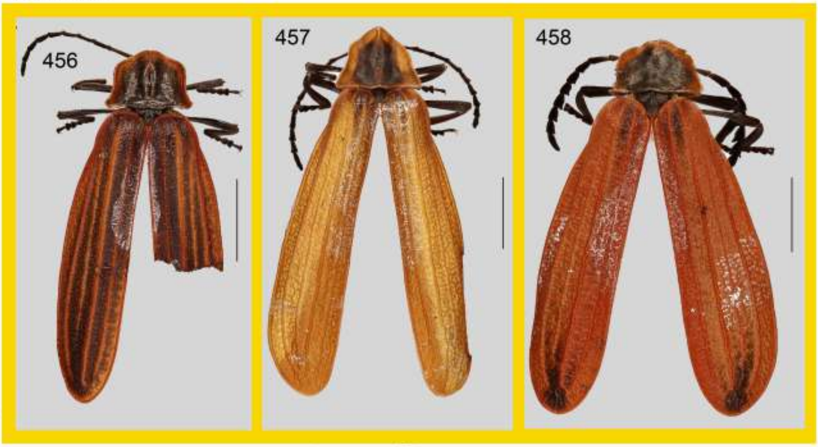

intraspecific polymorphism
geographically defined

Lycostomus sp., China

Lycostomus sp., China

Lycostomus sp., China

Palearctic Region

459

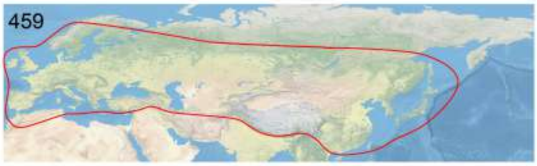

Himalayas

460

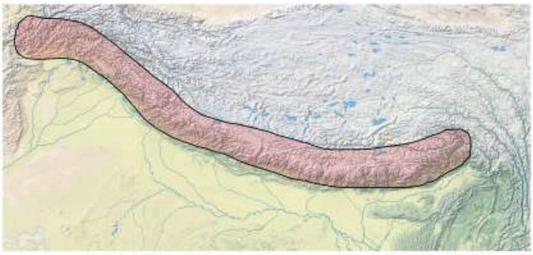

The phylogenetic structure of the Lycidae fauna

Subfamily	Tribe /Subtribe	# spp. (woldwide)	# spp. (region)
Dexorinae	Dexorini	15 spp.	
	Mimolibnetini	5 spp.	
Erotinae	Erotini	54 spp.	5 spp.
	Dictyopterini	73 spp.	10 spp.
	Taphini	31 spp.	
	Slipinskiini	46 spp.	
Calochrominae	Calochromini	289 spp.	15 spp.
Ateliinae	Ateliini	45 spp.	
	Lyponiini	45 spp.	2 spp.
	Macrolycini	69 spp.	2 spp.
Lyropaeinae	Lyropaeini	43 spp.	
	Alyculini	5 spp.	
	Antennolycini	3 spp.	
	Platerodrilini	49 spp.	2 spp.
Lycinae	Conderini	42 spp.	15 spp.
	Eurrhacini	102 spp.	
	Thonalmini	11 spp.	
	Leptolycini	12 spp.	
	Platerodini	861 spp.	30 spp.
	Calopterini	367 spp.	
	Lycini	413 spp.	10 spp.
Metriorrhynchinae	Dihammatini	44 spp.	5 spp.
	Lycoprogenthini	7 spp.	
	Libnetini	112 spp.	5 spp.
	Dilophotini	81 spp.	5 spp.
	Metriorrhynchini	1410 spp.	
	Metanoeina	40 spp.	
	Cautirina	750 spp.	10 spp.
	Metriorrhynchina	620 spp.	
Total		4234 spp.	~90 spp.

The presence of net-winged beetle aposematic patterns in the region

Group	Colour type	Characteristics	+/-	Figures
Uniform coloration	black	pronotum an elytra uniformly black to dark brown	✓	475, 476
	yellow	pronotum and elytra yellow to light brown		
	orange and red	pronotum and elytra brightly orange or cinnamon red	✓	*Helcophorus* spp., *Macrolycus* spp. (not shown)
	metallic (blue, green)	pronotum and elytra metalic, all shades of colours		
Bi-colored pronotum/ elytra	black/yellow	pronotum black (at most with bright margins), elytra yellow	✓	471
	black/red	pronotum black (at most with bright margins), elytra red		
	bright/black	pronotum brightly colored, elytra uniformly black	✓	474
	red/merallic	pronotum brightly red, elytra metallic blue		
Bi-colored elytra	yellow/black	elytra bi-colored. humeral part yellow, apical part dark coloured		
	red(orange)/black	elytra bi-colored. humeral part orange/red, apical part dark		
	black/bright	elytra bi-colored. humeral part black, apex yellow/orange/red		
Fasciate elytra	yellow/black	humeri and apex of elytra black, middle of elytron yellow		
	bright/black	humeri and apex of elytra brigt, middle of elytron black		
	yellow/metallic	most elytra black with blue metallic shine, middle yellow		
Striate elytra	bright/black	elytra brown/brightly coloured, suture or middle of elytron black	✓	463, 464, 466-468, 472, 473
Punctate el.	bright/black	elytra brown/brightly coloured, black patch in each elytron	✓	465
Tri-colored el.	all combinations of colors	elytron with three differently coloured parts		
Reticulate	bright/black	backgroud colour of elytra dark, costae large, brightly coloured		
Non-categorized aposematic patterns		see listed examples for further information		

All graphics and text produced by the authors as they are listed under the title of this article (CC-BY open acces license). Long horn beetle photographs taken by L. Dembicky.

Himalayas

461

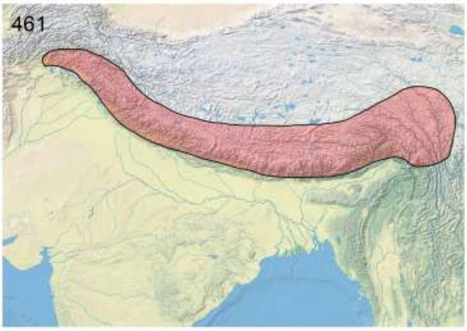

Calochrominae: Calochromini

Pattern: uniform cinnamon red

Body size: ~ 10 mm

Distribution: Himalayas

Non-lycid co-mimics:
Cantharidae

Remark. A pattern distributed mostly in Indo-Burma, uncommon in the Himalayas

462

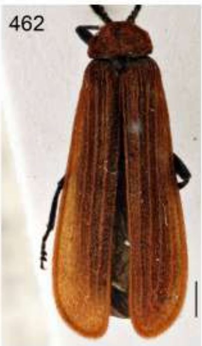

Micronychus vesticollis Gorham, Himalayas

Pattern: stripped red/black elytra, the pronotum black with brightly coloured lateral margins

Body size: ~ 10 mm

Distribution: Himalayas

Non-lycid co-mimics:
Cantharidae

Remark. A pattern rersembling Himalayan Lycini, distributed also in the eastern part of the Tibetan Plateau

Calochrominae: Calochromini

463

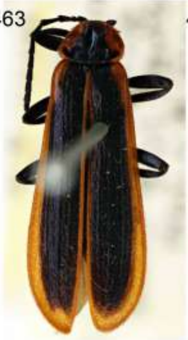

Micronychus sp., Yunnan

464

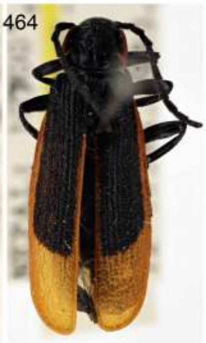

Micronychus sp., Himalayas

Pattern: red/black elytra with patches the pronotum black with brightly coloured lateral margins

Body size: ~ 10 mm

Distribution: Himalayas

Non-lycid co-mimics:
Cerambycidae

Remark. A pattern possibly derived from the preceeding one

Calochrominae: Calochromini

465

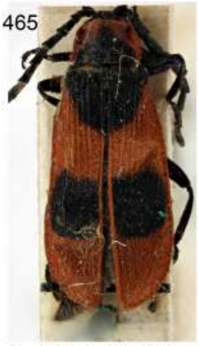

Parantis trimaculatus Kleine, Himalayas

Pattern: stripped red/black elytra, the pronotum black

Body size: ~ 7 mm

Distribution: Himalayas

Non-lycid co-mimics:
Cantharidae

Remark. Small-bodied lycids, the extent of patches variable seldom almost absent

Lycinae: Lycini

466

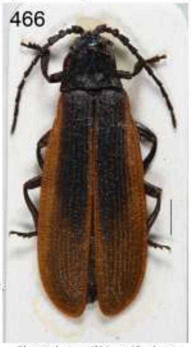

Plateros kumatai Nakane, Himalayas

467

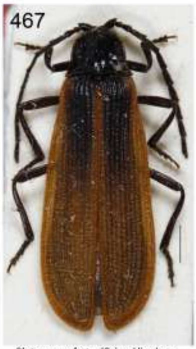

Plateros confertus Kleine, Himalayas

468

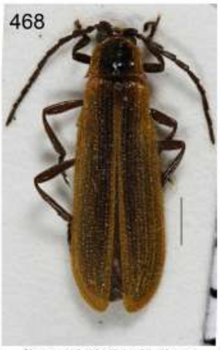

Plateros cimicoides Kleine, Himalayas

469

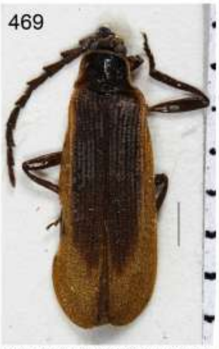

Plateros macrosuturalis Kasantsev, Himalayas

470

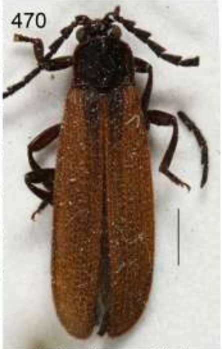

ateros demissus Kasantsev, Himalayas

A form with substantially reduced elytral patches

Lycinae: Lycini

471

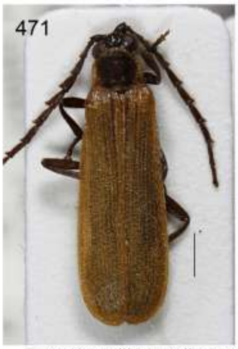

Plateros planatus Waterhouse, Himalayas

Pattern: stripped red/black elytra, the black area along the suture the pronotum black

Body size: ~ 7 mm

Distribution: Himalayas

Non-lycid co-mimics:
Cantharidae

Remark. Small-bodied lycids occurring togehter with thosethe displaying the preceeding pattern

Metriorrhynchinae: Libnetini

472

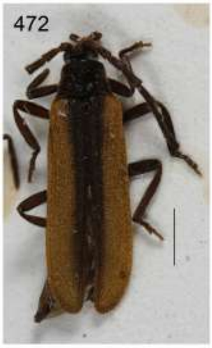

ibnetis piceovittatus Kasantsev, Himalayas

Lycinae: Platerodini

473

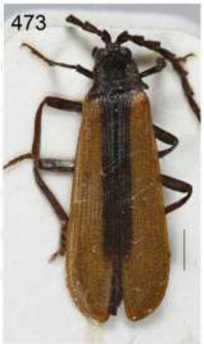

Plateros suturalis Pic, Himalayas

All graphics and text produced by the authors as they are listed under the title of this article (CC-BY open acces license). Long horn beetle photographs taken by L. Dembicky.

Pattern: black elytra, the pronotum black with red margins

Body size: ~ 7 mm

Distribution: Himalayas

Non-lycid co-mimics: not recorded

Remark. Small-bodied lycids, similar species known from Vietnam, southern China and Taiwan, rare in the Himalayas

Lycinae: Platerodini

474

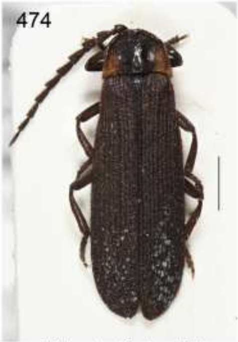

Plateros jageri Bocakova, Himalayas

Pattern: uniform black

Body size: ~ 7 mm

Distribution: Himalayas

Non-lycid co-mimics:
Cantharidae

Lycinae: Platerodini

475

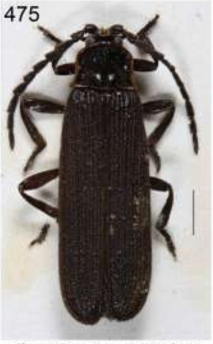

Plateros cinereus Kasantsev, Himalayas

Metriorrhynchinae: Dihammatini

476

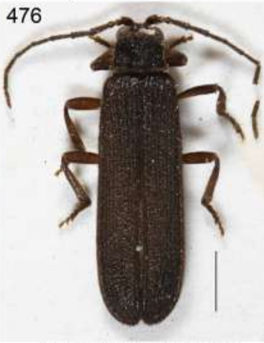

Dihammatus holzschuhi Bocakova, Nepal

Lycinae: Lycini

Pattern: pronotum with black patch and bright margins, red elytra, sometime striate

Body size: ~ 15 mm

Distribution: Himalayas

Non-lycid co-mimics: Cantharidae

477

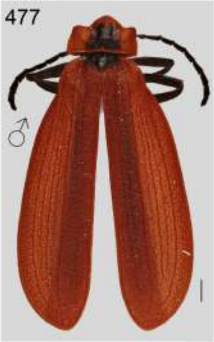

478

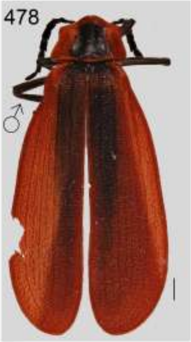

Southern China

479

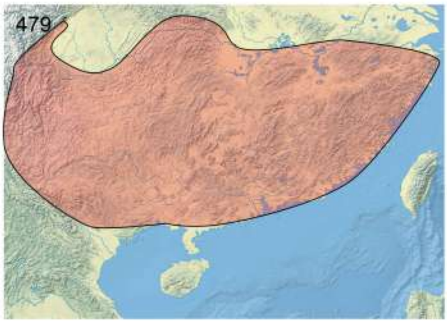

Cerambycidae

480

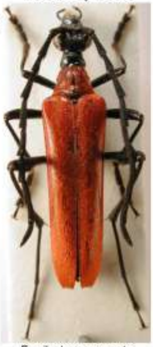

Emeileptura conspecta Holzschuh 1991 China, Sichuan

Cerambycidae

481

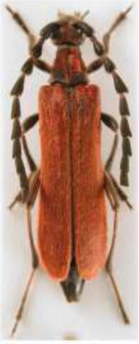

Formosopyrrhona longula Holzschuh 1999 China, Hubei

Cerambycidae

482

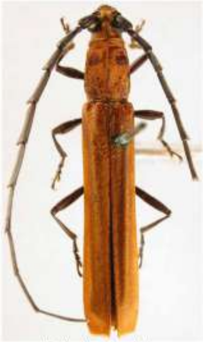

Erythresthes bowringi Pascoe 1863 Hong Kong

Cerambycidae

483

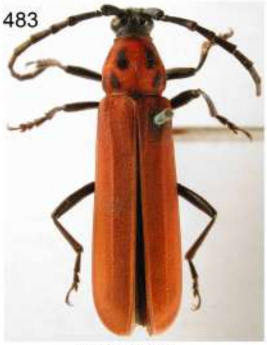

Erythrus congruus Pascoe 1863 Hong Kong

Cerambycidae

484

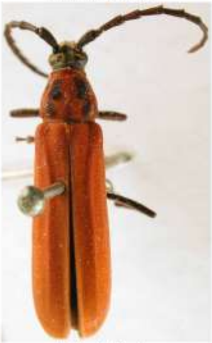

Erythrus fortunei White 1853 W China

All graphics and text produced by the authors as they are listed under the title of this article (CC-BY open acces license). Long horn beetle photographs taken by L. Dembicky.

Palearctic Region

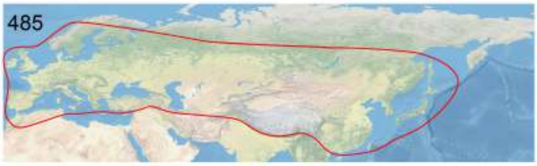

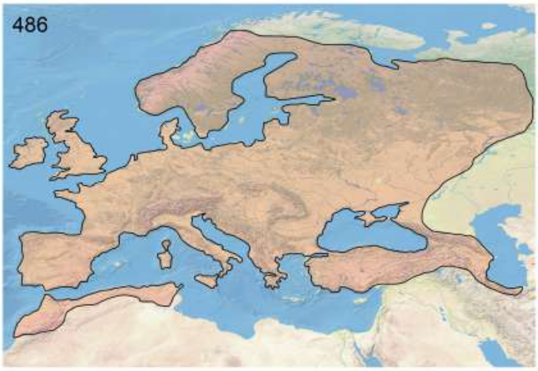

Phylogenetic structure of the fauna

Subfamily	Tribe /Subtribe	# spp. (woldwide)	# spp. (region)
Dexorinae	Dexorini	15 spp.	
	Mimolibnetini	5 spp.	
Erotinae	Erotini	54 spp.	6 spp.
	Dictyopterini	73 spp.	5 spp.
	Taphini	31 spp.	
	Slipinskiini	46 spp.	
Calochrominae	Calochromini	289 spp.	5 spp.
Ateliinae	Ateliini	45 spp.	
	Lyponiini	45 spp.	
	Macrolycini	69 spp.	
Lyropaeinae	Lyropaeini	43 spp.	
	Alyculini	5 spp.	
	Antennolycini	3 spp.	
	Platerodrilini	49 spp.	
Lycinae	Conderini	42 spp.	1 sp.
	Eurrhacini	102 spp.	
	Thonalmini	11 spp.	
	Leptolycini	12 spp.	
	Platerodini	861 spp.	
	Calopterini	367 spp.	
	Lycini	413 spp.	2 spp.
Metriorrhynchinae	Dihammatini	44 spp.	
	Lycoprogenthini	7 spp.	
	Libnetini	112 spp.	
	Dilophotini	81 spp.	
	Metriorrhynchini	1410 spp.	
	Metanoeina	40 spp.	
	Cautirina	750 spp.	
	Metriorrhynchina	620 spp.	
Total		4234 spp.	19 spp.

The presence of net-winged beetle aposematic patterns in the region

Group	Colour type	Characteristics	+/-	Examples
Uniform coloration	black	pronotum an elytra uniformly black to dark brown		
	yellow	pronotum and elytra yellow to light brown	✓	*Platycis schneideri* Kiesenwetter (Azerbaijan)
	orange and red	pronotum and elytra brightly orange or cinnamon red	✓	489-491, 494
	metallic (blue, green)	pronotum and elytra metalic, all shades of colours		
Bi-colored pronotum/ elytra	black/yellow	pronotum back (at most with bright margins), elytra yellow		
	black/red	pronotum black (at most with bright margins), elytra red	✓	492, *Pyropterus nigroruber*, *Benibotarus alternatus* (not shown)
	bright/black	pronotum brightly colored, elytra uniformly black		
	red/merallic	pronotum brightly red, elytra metallic blue		
Bi-colored elytra	yellow/black	elytra bi-colored. humeral part yellow, apical part dark coloured	✓	496, 498
	red(orange)/black	elytra bi-colored. humeral part orange/red, apical part dark		
	black/bright	elytra bi-colored. humeral part black, apex yellow/orange/red		
Fasciate elytra	yellow/black	humeri and apex of elytra black, middle of elytron yellow		
	bright/black	humeri and apex of elytra brigt, middle of elytron black		
	yellow/metallic	most elytra black with blue metallic shine, middle yellow		
Striate elytra	bright/black	elytra brown/brightly coloured, suture or middle of elytron black		
Punctate el.	bright/black	elytra brown/brightly coloured, black patch in each elytron		
Tri-colored el.	all combinations of colors	elytron with three differently coloured parts		
Reticulate	bright/black	backgroud colour of elytra dark, costae large, brightly coloured		
Non-categorized aposematic patterns		see listed examples for further information		

Figures S485–S498. The overview of the lycid fauna, coloration, and co-mimics: Palearctic region. Europe, the northern part of Africa, and Anatolia.

All graphics and text produced by the authors as they are listed under the title of this article (CC-BY open acces license). Long horn beetle photographs taken by L. Dembicky.

Palearctic region: Europe, Northern Africa, western Asia

487

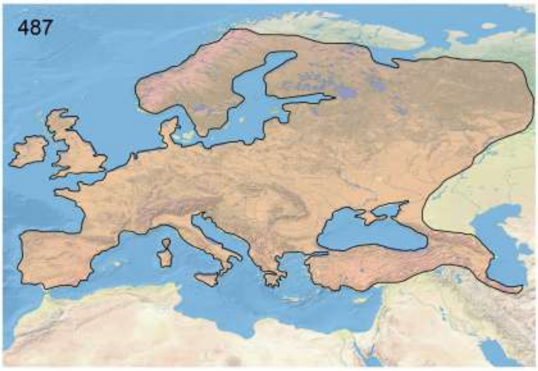

Pattern: Bicoloured: pronotum/elytra
pronotum with red margins

Body size: ~ 8 mm

Distribution: continental Europe

Non-lycis co-mimics: none

Remarks: flower visiting; the pattern shared with relatives from China (Motyka et al. 2017) and *L. anorachilus* Ragusa from Italy

Calochrominae: Calochromini

488

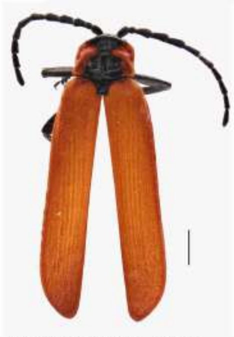

Lygistopterus sanguineus L., Slovakia

Patterns: Uniform red to bicoloured: pronotum/elytra intermediate forms have the pronotum with red margins

Body size: 5-7 mm

Distribution: continental Europe

Non-lycis co-mimics: none

Remarks: the pattern shared with relatives from eastern Paleactic and and Nearctic regions

Erotinae: Dictyopterini

489

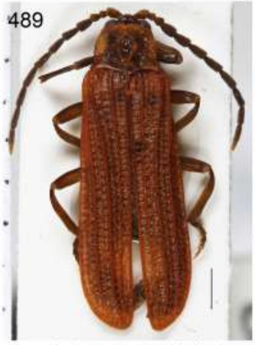

Benibotarus taygetanus Pic, Slovakia

490

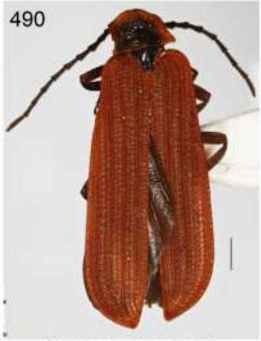

Dictyoptera aurora Herbst, Moravia

Erotini

491

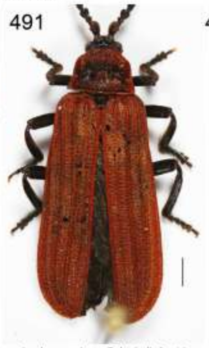

Lopheros rubens Gylenhall, Austria

492

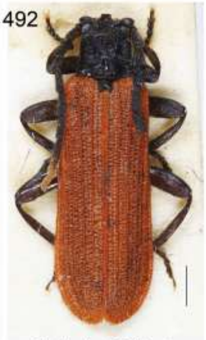

Platycis minutus F., Moravia

493

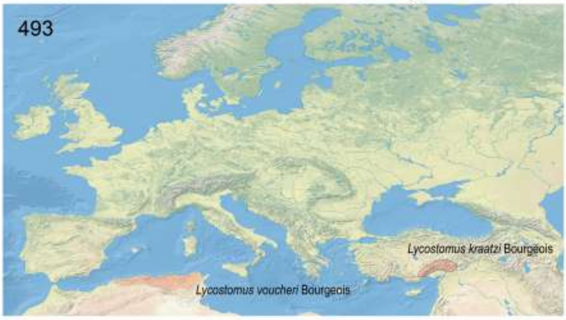

Patterns: Uniform red and
bicoloured: pronotum/elytra
pronotum with black patch

Body size: 13-16 mm

Distribution:
Northern Africa (*L. voucheri*, uniform orange) and southern Turkey (*L. kraatzi*)

Non-lycis co-mimics:none

Remarks: flower visiting; the pattern shared with relatives from eastern Palearctic region

Lycinae: Lycini

494

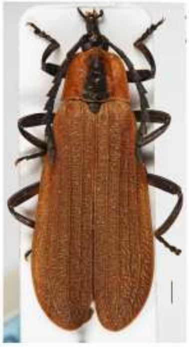

Lycostomus kraatzi Bourgeois., Turkey

495

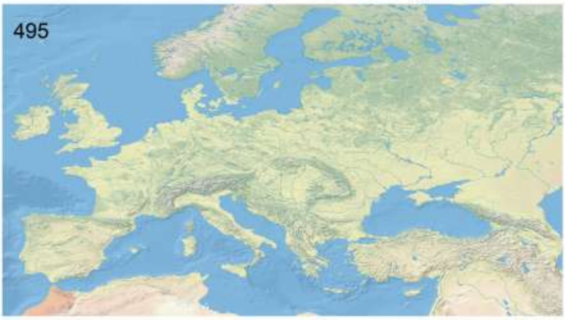

Pattern: Bicoloured: elytra
humeri yellow, apex black

Body size: ~ 5 mm

Distribution: Morroco

Non-lycis co-mimics: Elateridae: Agrypninae: Drilini

Remarks: the pattern shared with relatives from Sub-Saharan Arfica (Motyka et al. 2017)

Calochrominae: Calochromini

496

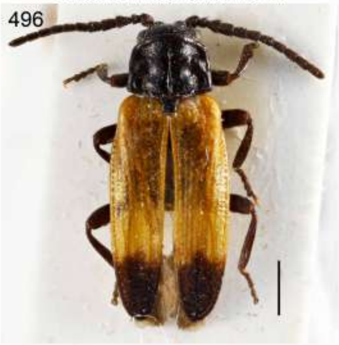

Lygistopterus caroli Bourgeois, Morocco

497

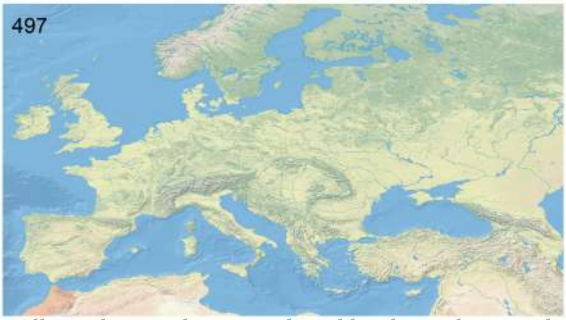

Pattern: Bicoloured: elytra
humeri orange, apex black

Body size: ~ 5 mm

Distribution: Morroco

Non-lycis co-mimics: none

Remarks: the pattern shared with relatives from Sub-Saharan Arfica (Motyka et al. 2017)

Calochrominae: Calochromini

498

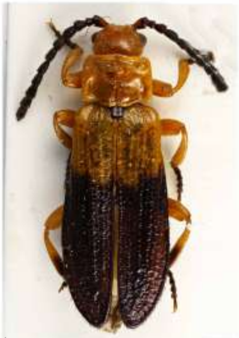

Lygistopterus alluaudi Bourgeois, Morocco, Alger

All graphics and text produced by the authors as they are listed under the title of this article (CC-BY open acces license). Long horn beetle photographs taken by L. Dembicky.

Mesoamerica

499

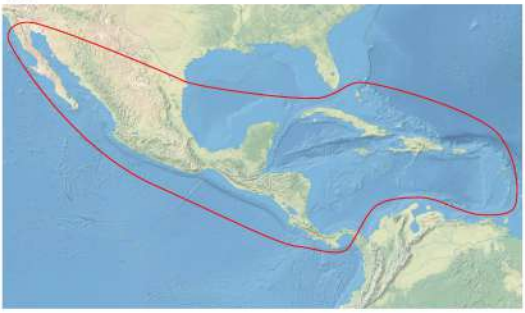

Continental Mesoamerica sensu Holt *et al.* (2013)

500

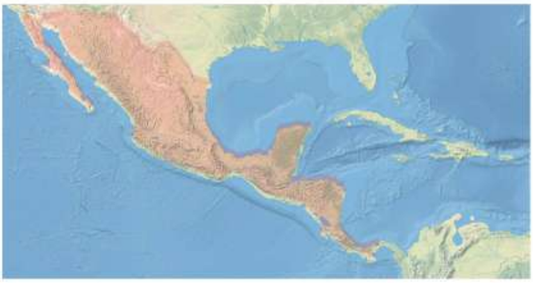

The phylogenetic structure of the Lycidae fauna

Subfamily	Tribe /Subtribe	# spp. (woldwide)	# spp. (region)
Dexorinae	Dexorini	15 spp.	
	Mimolibnetini	5 spp.	
Erotinae	Erotini	54 spp.	
	Dictyopterini	73 spp.	
	Taphini	31 spp.	
	Slipinskiini	46 spp.	
Calochrominae	Calochromini	289 spp.	~25 spp.
Ateliinae	Ateliini	45 spp.	
	Lyponiini	45 spp.	
	Macrolycini	69 spp.	
Lyropaeinae	Lyropaeini	43 spp.	
	Alyculini	5 spp.	
	Antennolycini	3 spp.	
	Platerodrilini	49 spp.	
Lycinae	Conderini	42 spp.	
	Eurrhacini	102 spp.	~10 spp.
	Thonalmini	11 spp.	
	Leptolycini	12 spp.	
	Platerodini	861 spp.	~70 spp.
	Calopterini	367 spp.	~65 spp.
	Lycini	413 spp.	~15 spp.
Metriorrhynchinae	Dihammatini	44 spp.	
	Lycoprogenthini	7 spp.	
	Libnetini	112 spp.	
	Dilophotini	81 spp.	
	Metriorrhynchini	1410 spp.	
	Metanoeina	40 spp.	
	Cautirina	750 spp.	
	Metriorrhynchina	620 spp.	
Total		4234 spp.	~185 spp.

The presence of net-winged beetle aposematic patterns in the region

Group	Colour type	Characteristics	+/-	Figures
Uniform coloration	black	pronotum an elytra uniformly black to brown		
	yellow	pronotum and elytra yellow to light brown	✓	501, 502
	orange and red	pronotum and elytra brightly orange or cinnamon red	✓	503
	metallic (blue, green)	pronotum and elytra metalic, all shades of colours		
Bi-colored pronotum/ elytra	black/yellow	pronotum black (at most with bright margins), elytra yellow		
	black/red	pronotum black (at most with bright margins), elytra red		
	bright/black	pronotum brightly colored, elytra uniformly black		
	red/merallic	pronotum brightly red, elytra metallic blue		
Bi-colored elytra	yellow/black	elytra bi-colored. humeral part yellow, apical part dark coloured	✓	513-515
	red(orange)/black	elytra bi-colored. humeral part orange/red, apical part dark	✓	527-532
	black/bright	elytra bi-colored. humeral part black, apex yellow/orange/red		
Fasciate elytra	yellow/black	humeri and apex of elytra black, middle of elytron yellow		
	bright/black	humeri and apex of elytra brigt, middle of elytron black	✓	512, 516-520
	yellow/metallic	most elytra black with blue metallic shine, middle yellow		
Striate elytra	bright/black	elytra brown/brightly coloured, suture or middle of elytron black		
Punctate el.	bright/black	elytra brown/brightly coloured, black patch in each elytron	✓	509
Tri-colored el.	all combinations of colors	elytron with three differently coloured parts		
Reticulate	bright/black	backgroud colour of elytra dark, costae large, brightly coloured		
Non-categorized aposematic patterns		see listed examples for further information		

Figures S499–S537. The overview of the lycid fauna, coloration, and co-mimics: Mesoamerican region, and the Caribbean.

All graphics and text produced by the authors as they are listed under the title of this article (CC-BY open acces license). Long horn beetle photographs taken by L. Dembicky.

Pattern: uniform yellow,
thepronotum with
a black patch

Body size: ~ 7 mm

Distribution:
Continental Mesoamerica

Non-lycid co-mimics:
Cantharidae

Lycinae: Platerodini

501

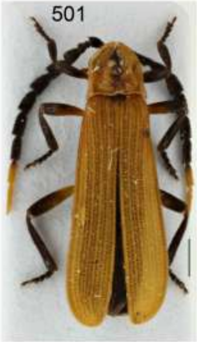

Plateros sp., Panama

502

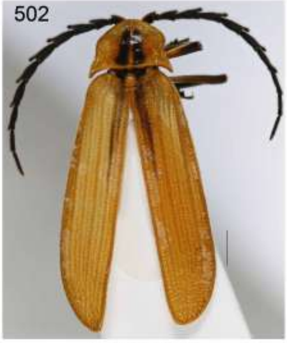

Plateros sp., Panama

Pattern: red elytra with black apices
and area at the scutellum,
the pronotum with black patch
and yellow margins
Body size: ~ 9 mm

Distribution: Costa Rica

Non-lycid co-mimics: *Ormetica* sp.
(Lepidoptera: Erebidae)

Remark: A unique endemic pattern

Lycinae: Platerodini

503

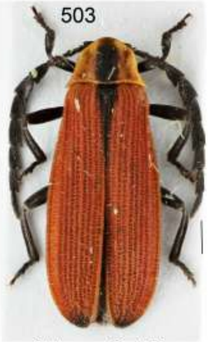

Plateros sp., Costa Rica

Pattern: red elytra with a black area at the
scutellum, the pronotum with a black patch

Body size: ~ 9 mm

Distribution: southern part of Mesoamerica

Non-lycid co-mimics:
Cantharidae

Remark: A pattern resembling Holoarctic Erotinae

Lycinae: Platerodini

504

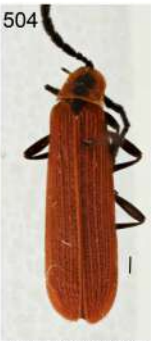

Plateros sp., Costa Rica

505

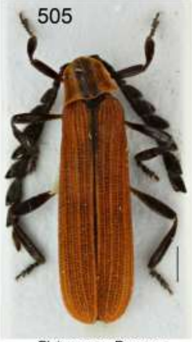

Plateros sp., Panama

506

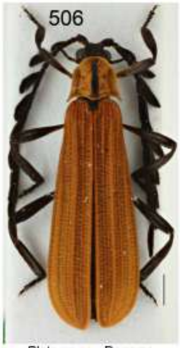

Plateros sp., Panama

Pattern: bicoloured elytra, a yellow humeral part and
black apices, the pronotum with a black patch

Body size: 5-9 mm

Distribution: continental Mesoamerica

Lycis and Non-lycid co-mimics:
Cantharidae

Remark: A widespread pattern

Lycinae: Calopterini

507

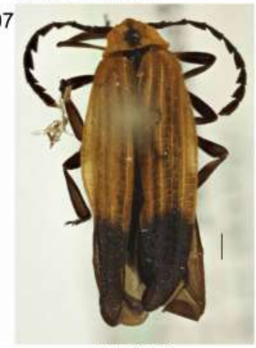

indet., Costa Rica

Lycinae: Calopterini

508

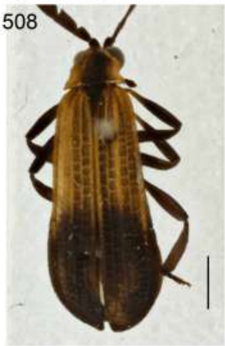

indet., Costa Rica

All graphics and text produced by the authors as they are listed under the title of this article (CC-BY open acces license). Long horn beetle photographs taken by L. Dembicky.

Pattern: bicoloured elytra with two black patches
the pronotum yellow a black median
patch
Body size: ~ 8 mm

Distribution: Mesoamerica

Non-lycid co-mimics: unknown

Remark: A unique rare pattern

509

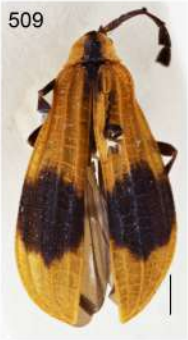

Calopteron beltii Gorham, Nicaragua

Pattern: bicoloured elytra, yellow humeral part;
the black suture and apices, the pronotum
with a black patch

Body size: ~7 mm

Distribution: the southern part of continental
Mesoamerica

Lycis and Non-lycid co-mimics: unknown

Lycinae: Calopterini

510

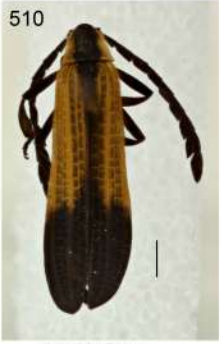

indet., Costa Rica

Continental Mesoamerica sensu Holt *et al.* (2013)

511

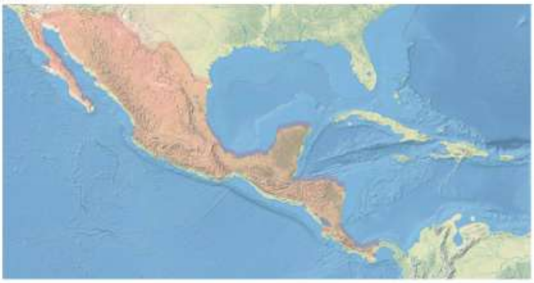

Pattern: bicoloured apically widemed elytra, with a black middle transverse band and black apices; the pronotum with a middle black patch

Body size: 11-15 mm

Distribution: continental Mesoamerica

Lycis and Non-lycid co-mimics: various moths

Remark: A widespread pattern known from most of Continental South America

Lycinae: Calopterini

512

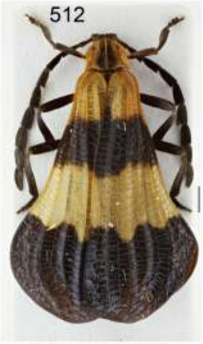

Calopteron sp., Panama

Pattern: bicoloured elytra, the apex black, humeral part yelloww; the pronotum yellow usually with a black patch

Body size: 11-15 mm

Distribution: continental Mesoamerica

Non-lycid co-mimics: Cantharidae, various moths

Remark: A very widespread pattetn known from the nearctic and Neotropical region.

Lycinae: Calopterini

513

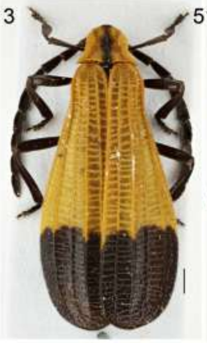

indet., Panama

514

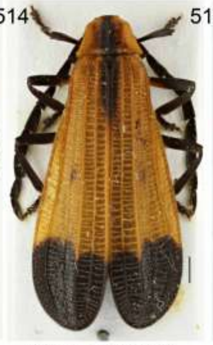

Calopteron sp., Panama

515

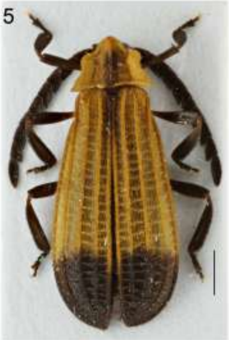

indet., Panama

Pattern: bicoloured elytra, with a black middle transverse band and black apices; the pronotum with a middle black patch

Body size: 11-15 mm

Distribution: continental Mesoamerica

Lycis and Non-lycid co-mimics: various moths

Remark: A widespread pattern known from most of Continental South America

Lycinae: Calopterini

516

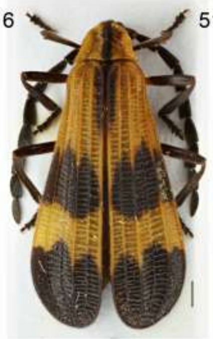

Calopteron sp., Panama

517

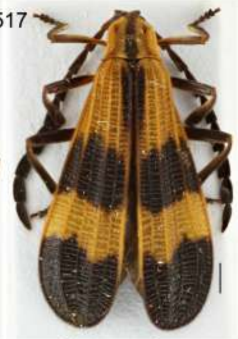

Calopteron sp., Panama

518

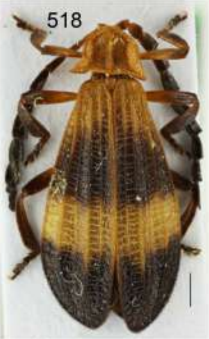

Calopteron sp., Panama

Lycinae: Calopterini

519

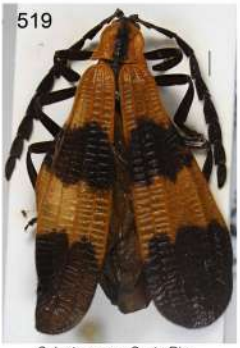

Calopteron sp., Costa Rica

520

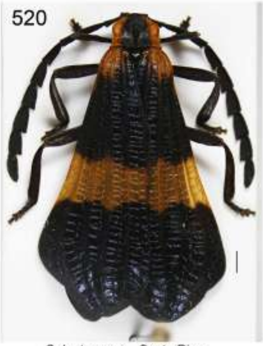

Calopteron sp., Costa Rica

Pattern: bicoloured elytra with two yellow humeral patches the pronotum yellow a black median patch

Body size: ~ 6 mm

Distribution: Mesoamerica

Non-lycid co-mimics: unknown

Remark: A unique rare pattern

Lycinae: Calopterini

521

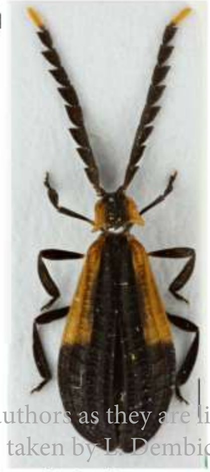

Caenia sp., Panama

All graphics and text produced by the authors as they are listed under the title of this article (CC-BY open acces license). Long horn beetle photographs taken by L. Dembicky.

Continental Mesoamerica sensu Holt *et al.* (2013)

522

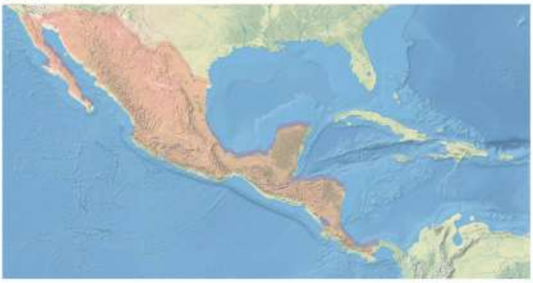

Pattern: bicoloured elytra, yellow with two black patches at the scutellum, the apex of elytra black, the pronotum yellow a black median patch

Body size: ~ 8 mm

Distribution: Mesoamerica

Non-lycid co-mimics: unknown

Remark: A pattern known also from sontinental South America

Lycinae: Platerodini

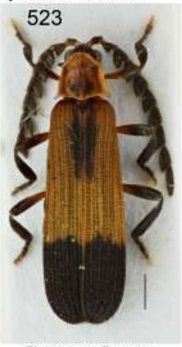

Plateros sp., Panama

Pattern: bicoloured elytra, black with yellow patches at humeri, the pronoutum with a black patch

Body size: ~ 5 mm

Distribution: Mesoamerica

Non-lycid co-mimics: unknown

Remark: A pattern known also from continental South America

Lycinae: Platerodini

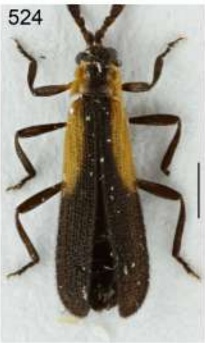

Plateros sp., Panama

Pattern: bicoloured elytra, black with yellow patches at humeri and a yellow band in the middle part, the pronoutum with a black patch

Body size: ~ 5 mm

Distribution: Mesoamerica

Non-lycid co-mimics: unknown

Remark: A pattern known also from continental South America

Lycinae: Platerodini

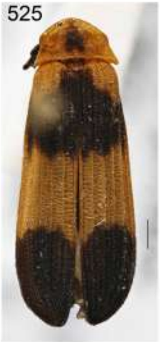

Plateros sp. , Mexico

Pattern: fark brown elytra, the pronoutum yellow with a black patch

Body size: ~ 5 mm

Distribution: Mesoamerica

Non-lycid co-mimics: unknown

Remark: A pattern known also from continental South America

Lycinae: Calopterini

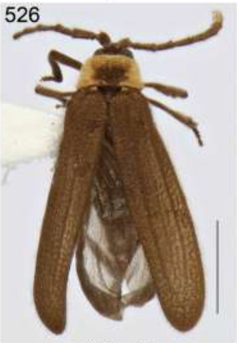

indet., Costa Rica

Pattern: black elytra, the pronoutum with a black patch

Body size: ~ 5 mm

Distribution: Mesoamerica

Non-lycid co-mimics: unknown

Remark: A pattern known also from continental South America and the Neotropical region

Lycinae: Platerodini

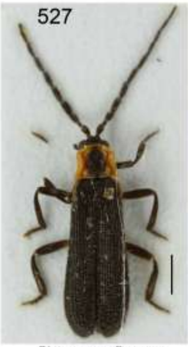

Plateros sp., Panama

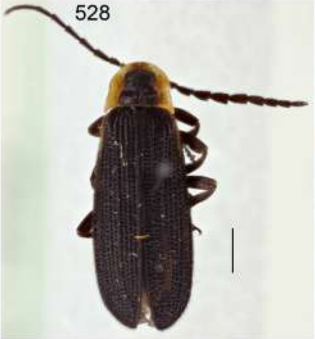

Plateros sp., Costa Rica

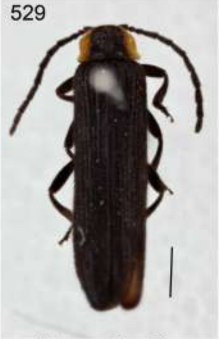

Plateros sp., Costa Rica

Lycinae: Platerodini

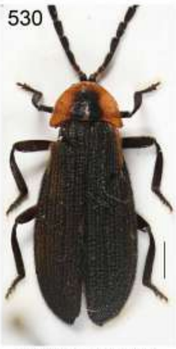

Plateros sp., Costa Rica

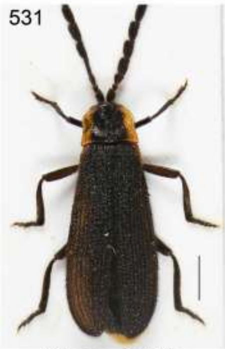

Plateros sp., Costa Rica

Pattern: black elytra, the pronoutum with a black patch

Body size: ~12 mm

Distribution: Mesoamerica

Non-lycid co-mimics: unknown

Remark: A pattern very similar to the preceeding, but it difers in the body size

Calochrominae: Calochromini

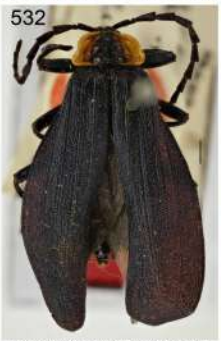

Macrolygistopterus caerulescens Gorham, Guatemala

All graphics and text produced by the authors as they are listed under the title of this article (CC-BY open acces license). Long horn beetle photographs taken by L. Dembicky.

Caribbean

533

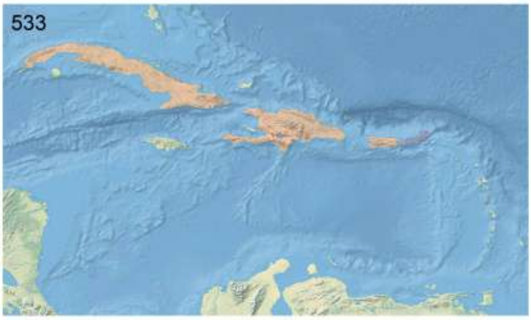

The Carribean Lycidae fauna

Unlike the continetal fauna, the Lycidae are represented in the Great Antilles, Virgin Islands and Lesser Antilles only by two endemic tribes: the widespread Thonalmini and by the Leptolycini restricted to the Greater Antilles and the Virgin Islands.

There are no Lycidae known from Jamaica.

Pattern: orange elytra and the blue elytral apices

Body size: ~6 mm
Distribution: the Great Antilles, Montserrat

Non-lycid co-mimics: unknown

Remark: A unique pattern

534

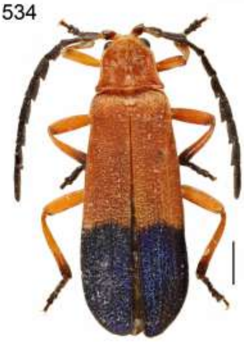

Pattern: dark brown elytra, with yellow patches at humeri, the pronoutum black

Body size: 2-3 mm
Distribution: the Great Antilles, Virgin Islands

Non-lycid co-mimics: unknown

Remark: extremely small bodied neotenic netwinged beetles,

Lycinae: Leptolycini

535

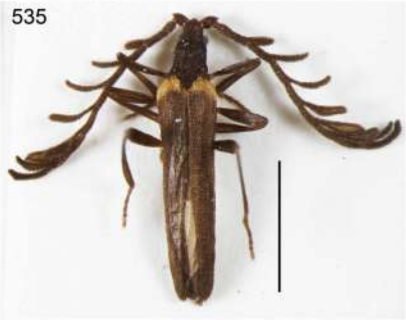

536

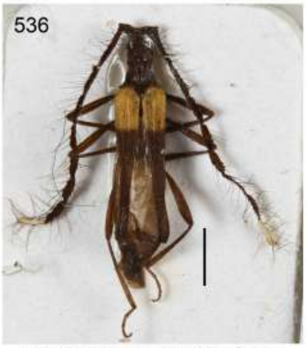

Leptolycus hetericornis Leng et Mutchler, Puerto Rico

Cerambycidae

537

Lycidae co-mimics:
Thonalmus spp.

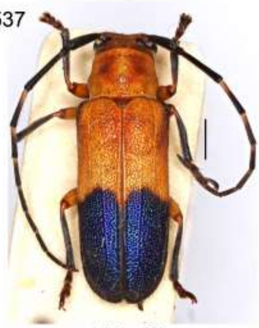

indet. sp. Cuba

All graphics and text produced by the authors as they are listed under the title of this article (CC-BY open acces license). Long horn beetle photographs taken by L. Dembicky.

South America

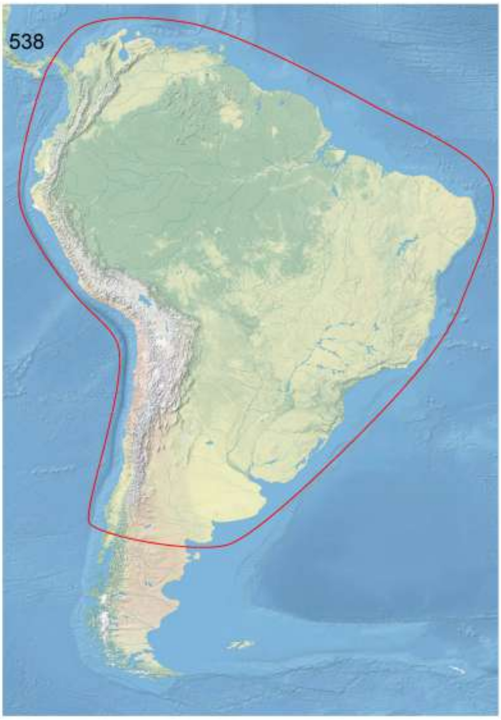

The phylogenetic structure of the Lycidae fauna

Subfamily	Tribe /Subtribe	# spp. (woldwide)	# spp. (region)
Dexorinae	Dexorini	15 spp.	
	Mimolibnetini	5 spp.	
Erotinae	Erotini	54 spp.	
	Dictyopterini	73 spp.	
	Taphini	31 spp.	
	Slipinskiini	46 spp.	
Calochrominae	Calochromini	289 spp.	~35 spp.
Ateliinae	Ateliini	45 spp.	
	Lyponiini	45 spp.	
	Macrolycini	69 spp.	
Lyropaeinae	Lyropaeini	43 spp.	
	Alyculini	5 spp.	
	Antennolycini	3 spp.	
	Platerodrilini	49 spp.	
Lycinae	Conderini	42 spp.	
	Eurrhacini	102 spp.	~90 spp.
	Thonalmini	11 spp.	
	Leptolycini	12 spp.	
	Platerodini	861 spp.	~80 spp.
	Calopterini	367 spp.	~290 spp.
	Lycini	413 spp.	10 spp.
Metriorrhynchinae	Dihammatini	44 spp.	
	Lycoprogenthini	7 spp.	
	Libnetini	112 spp.	
	Dilophotini	81 spp.	
	Metriorrhynchini	1410 spp.	
	Metanoeina	40 spp.	
	Cautirina	750 spp.	
	Metriorrhynchina	620 spp.	
Total		4234 spp.	~500 spp.

The presence of net-winged beetle aposematic patterns in the region

Group	Colour type	Characteristics	+/-	Figures
Uniform coloration	black	pronotum an elytra uniformly black to dark brown	✓	549, 550
	yellow	pronotum and elytra yellow to light brown	✓	540-542
	orange and red	pronotum and elytra brightly orange or cinnamon red		
	metallic (blue, green)	pronotum and elytra metalic, all shades of colours	✓	572
Bi-colored pronotum/ elytra	black/yellow	pronotum back (at most with bright margins), elytra yellow		
	black/red	pronotum black (at most with bright margins), elytra red		
	bright/black	pronotum brightly colored, elytra uniformly black	✓	548, 618, 632
	red/merallic	pronotum brightly red, elytra metallic blue	✓	630, 631
Bi-colored elytra	yellow/black	elytra bi-colored. humeral part yellow, apical part dark coloured	✓	544-547, 582, 583, 624-627
	red(orange)/black	elytra bi-colored. humeral part orange/red, apical part dark	✓	614-616
	black/bright	elytra bi-colored. humeral part black, apex yellow/orange/red	✓	558-563
Fasciate elytra	yellow/black	humeri and apex of elytra black, middle of elytron yellow	✓	564-570, 585-590
	bright/black	humeri and apex of elytra brigt, middle of elytron black	✓	562, 563, 591-594, 596-598
	yellow/metallic	most elytra black with blue metallic shine, middle yellow	✓	573
Striate elytra	bright/black	elytra brown/brightly coloured, suture or middle of elytron black	✓	557, 576, 600, 604
Punctate el.	bright/black	elytra brown/brightly coloured, black patch in each elytron	✓	552 (black patches), 577-580 (yellow patches)
Tri-colored el.	all combinations of colors	elytron with three differently coloured parts		
Reticulate	bright/black	backgroud colour of elytra dark, costae large, brightly coloured	✓	575
Non-categorized aposematic patterns		see listed examples for further information		

Figures S538–S653. The overview of the lycid fauna, coloration, and co-mimics: Neotropical region.

All graphics and text produced by the authors as they are listed under the title of this article (CC-BY open acces license). Long horn beetle photographs taken by L. Dembicky.

South America (except the southernmost region)

539
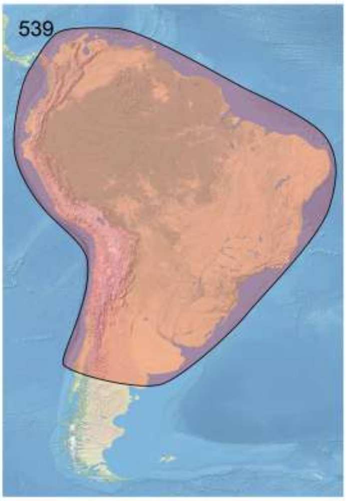

Pattern: uniform yellow elytra the pronotum with a black patch
Body size: ~ 8 mm

Distribution: continental South America

Further Lycidae co-mimics: *Calopteron* spp.

Non-lycid co-mimics:
Cantharidae

Lycinae: Calopterini

540
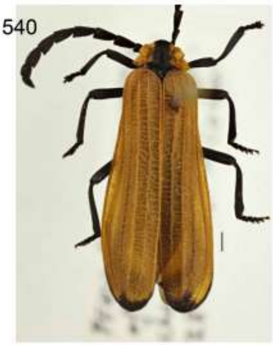
indet., Peru

541
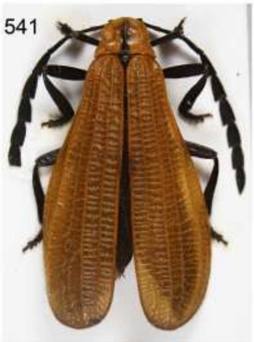
Idiopteron sp., Bolivia

Lycinae: Calopterini

542
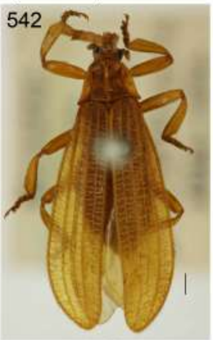
indet., Ecuador

Pattern: bicoloured elytra; yellow with paches at the suture and apex the pronotum yellow with a patch(s)

Body size: ~ 8 mm

Distribution: continental South America

Non-lycid co-mimics:
Cantharidae, Cerambycidae

Lycinae: Platerodini

543
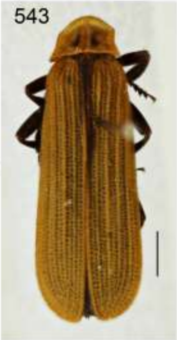
Plateros sp., Venezuela

544
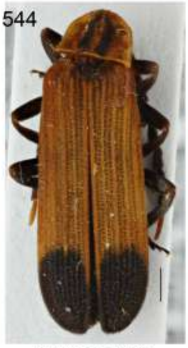
Plateros sp., Bolivia

Pattern: bicoloured elytra; yellow with paches at the suture and apex the pronotum yellow with a patch(s)

A form with two parallel sutural patches at the base of elytra

Lycinae: Platerodini

545
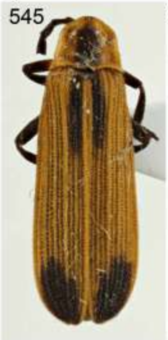
Plateros sp., Argentina

546
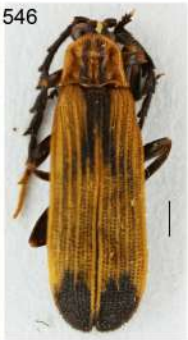
Plateros sp., Bolivia

Pattern: bicoloured elytra; yellow with paches at the suture and apex the pronotum yellow with a patch(s)

A form with a single scutellar patch

Lycinae: Platerodini

547
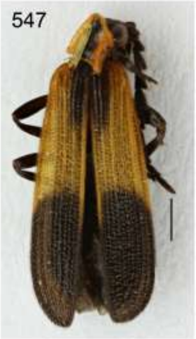
Plateros sp., Bolivia

Pattern: bicoloured elytra with a black scutellar patch and a reticulate structure of elytral cells; the pronotum black with light margins
Body size: ~ 8 mm

Distribution: the Eastern Andes

Non-lycid co-mimics: not recorded

Lycinae: Calopterini

548
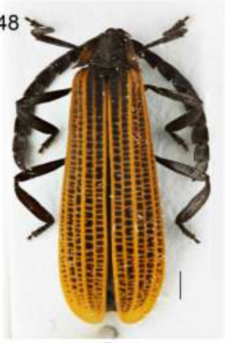
indet., Ecuador

Pattern: uniform black elytra and the pronotum

Body size: ~ 8 mm

Distribution: continental South America

Non-lycid co-mimics: not recorded

Lycinae: Platerodini

549
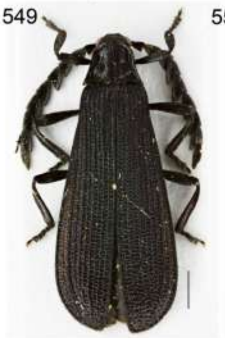
Plateros sp., Ecuador

550
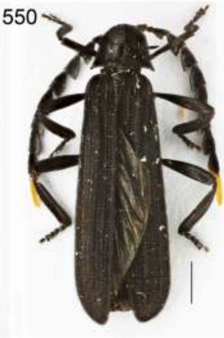
Eurrhacus sp., Ecuador

Pattern: bicoloured elytra; yellow with paches at the suture, the mid of the elytron and the apex; the pronotum yellow with a patch(s)

Body size: ~ 8 mm

Distribution: continental South America

Non-lycid co-mimics:
not recorded

Lycinae: Platerodini

551
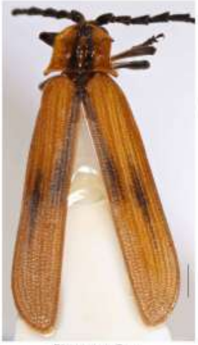
Plateros sp., Peru

Lycinae: Calopterini

552
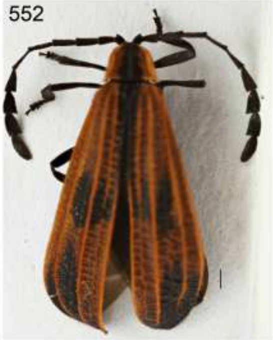
indet., Peru

All graphics and text produced by the authors as they are listed under the title of this article (CC-BY open acces license). Long horn beetle photographs taken by L. Dembicky.

Lycinae: Platerodini

Pattern: bicoloured elytra; black humeri and yellow to red apex the pronotum uniform black

Body size: ~ 8 mm

Distribution: continental South America

Non-lycid co-mimics: Cantharidae

553
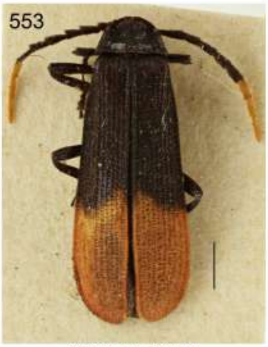
Plateros sp., Ecuador

554
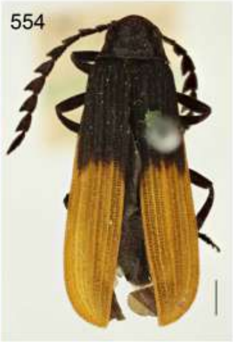
Plateros sp., Ecuador

555
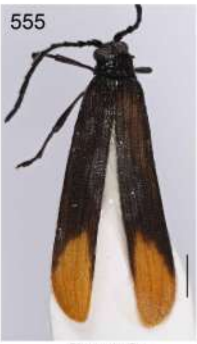
Plateros sp., Peru

556
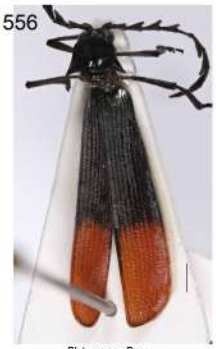
Plateros sp., Peru

Pattern: bicoloured elytra; black humeri and yellow to red apex with light coloured elytral costae and back interspaces; the pronotum uniform black

Body size: ~ 8 mm

Distribution: continental South America

Non-lycid co-mimics: not recorded

Lycinae: Calopterini

557
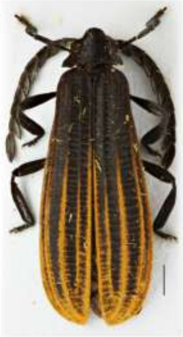
indet., Ecuador

Lycinae: Calopterini

558
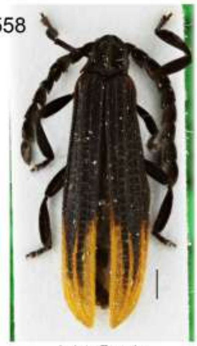
indet., Ecuador

Lycinae: Platerodini

559
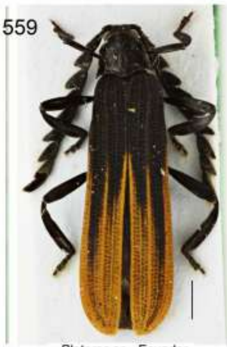
Plateros sp., Ecuador

Lycinae: Calopterini

560
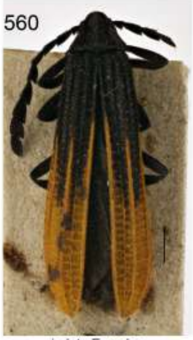
indet., Ecuador

Pattern: bicoloured elytra; black humeri and yellow to red apex with light coloured elytral costae and back interspaces; the pronotum uniform black

A form with black apex of the elytra

Lycinae: Calopterini

561
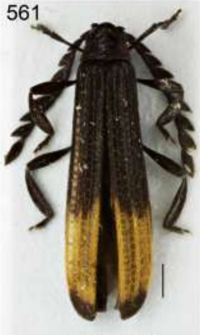
indet., Ecuador

Pattern: bicoloured elytra; reddish humeri and apex, with a black sutural patch widened posteriorly; the pronotum reddish with a median black patch

Body size: ~ 8 mm

Distribution: continental South America

Non-lycid co-mimics: not recorded

Lycinae: Platerodini

562
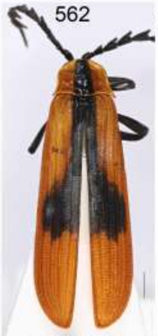
Plateros sp., Peru

563
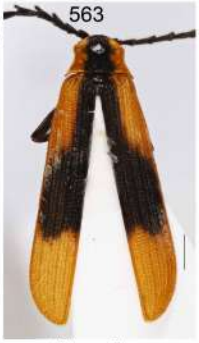
Plateros sp., Peru

Pattern: bicoloured elytra; reddish humeri and apex, with a black sutural patch widened posteriorly, the apex of elytra black; the pronotum reddish, with a median black patch

Body size: ~ 8 mm

Distribution: continental South America

Non-lycid co-mimics: not recorded

Remark: A very widespread pattern known from the whole Nearctic region.

564
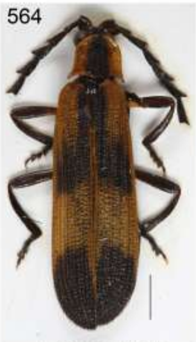
Eurrhacus sp., Boivia

565
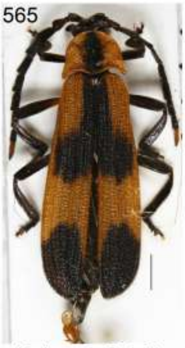
Eurrhacus sp., Costa Rica

566
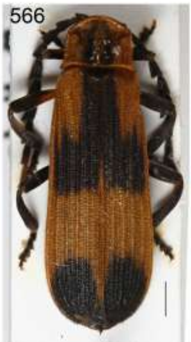
Eurrhacus sp., Bolivia

Lycinae: Platerodini

567
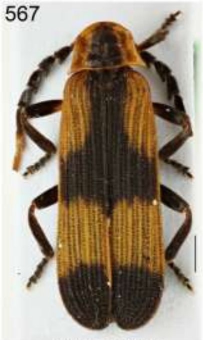
Plateros sp., Ecuador

All graphics and text produced by the authors as they are listed under the title of this article (CC-BY open acces license). Long horn beetle photographs taken by L. Dembicky.

Lycinae: Calopterini

568
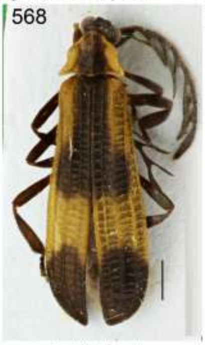
indet., Ecuador

Lycinae: Platerodini

569
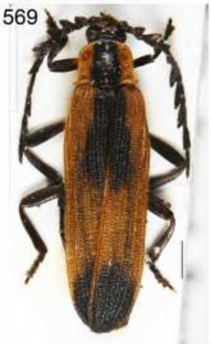
Plateros sp., Bolivia

Pattern: bicoloured elytra; reddish humeri and apex, with a black sutural patch widened posteriorly, the apex of elytra black; the pronotum reddish, with a median black patch

A red high-contras form of the previous pattern

Remark: The highly similar pattern with *Calochromus tricoloratus* Kleine from the Himalayas

570
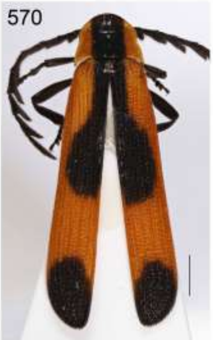
Plateros sp., Peru

The eastern Andes

571

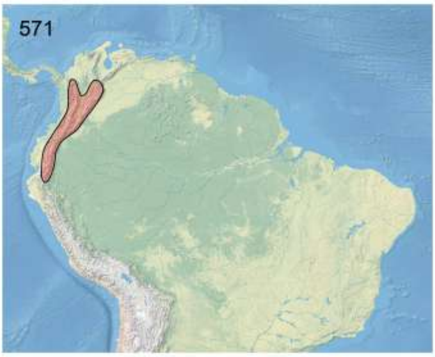

Lycinae: Calopterini

Pattern: uniform metallic blue elytra; the pronotum black
Body size: 12-15 mm

Distribution: the Eastern Andes

Non-lycid co-mimics: *Lycidola felix*, Fig. 652 Coleoptera: Cerambycidae)

Remark: The pattern known from high moountain, up to 4500 m a. s. l. Large-bodied lycids with dilated elytra

572

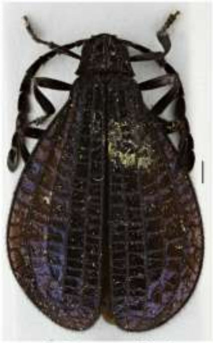

Calopteron sp., Ecuador

Lycinae: Calopterini

Pattern: metallic blue elytra with transverse yellow band; the pronotum black
Body size: 13-15 mm

Distribution: the Eastern Andes

Non-lycid co-mimics: *Correbia* sp. (Lepidoptera: Arctiidae) Cerambycidae - *Pteroplatus pulcher*, Fig. 653

573

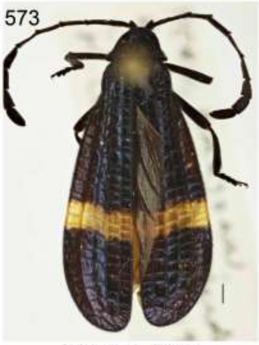

Calopteron sp., Ecuador

Lycinae: Calopterini

Pattern: black elytra with yellow apex and reticular structure; the pronotum black
Body size: 10-13 mm

Distribution: the Eastern Andes

Non-lycid co-mimics: not recorded

574

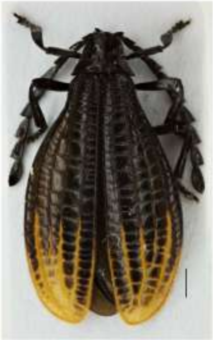

Calopteron sp., Ecuador

Lycinae: Calopterini

Pattern: black elytra a yellow reticular structure in the middle part; the pronotum black
Body size: 12-15 mm

Distribution: the Eastern Andes

Non-lycid co-mimics: not recorded

575

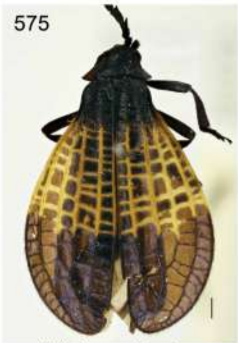

Calopteron sp., Ecuador

Lycinae: Calopterini

Pattern: whole elytra stripped; the pronotum black
Body size: 17 mm

Distribution: the Eastern Andes

Non-lycid co-mimics: not recorded

Remark: a rare pattern

576

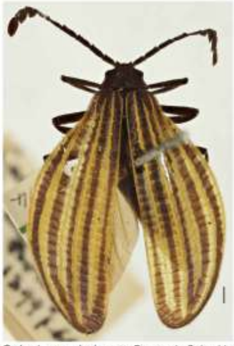

Calopteron gloriossus Bourgeois Colombia

Pattern: black elytra with a yellow patch in the middle part; the pronotum uniform black or with light coloured lateral margins
Body size: 6-12 mm

Distribution: continental South America

Non-lycid co-mimics: not recorded

Lycinae: Platerodini

577

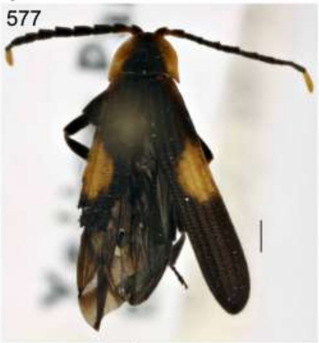

indet., Peru

Lycinae: Calopterini

578

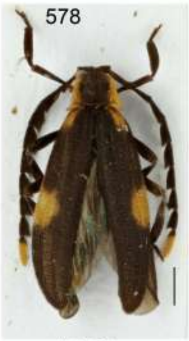

indet., Peru

579

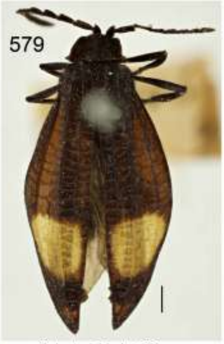

Calopterini indet., Peru

580

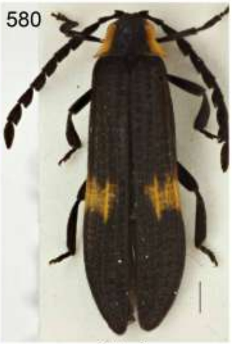

indet., Peru

All graphics and text produced by the authors as they are listed under the title of this article (CC-BY open acces license). Long horn beetle photographs taken by L. Dembicky.

Colombia

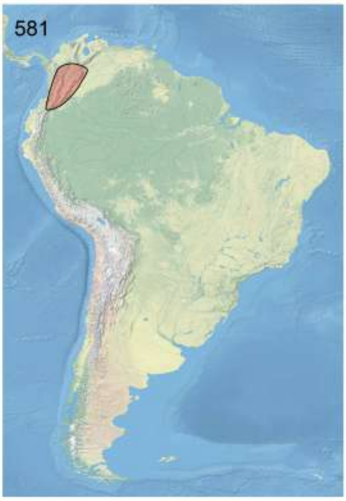

Pattern: bicoloured elytra with
a yellow humeral half and the
black apex; the pronotum with
a median black patch and yellow
margins
Body size: 6-10 mm

Distribution: continental
South America

Non-lycid co-mimics:
Cantharidae

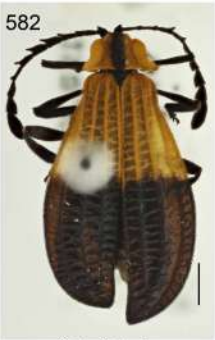

indet., Colombia

Pattern: bicoloured elytra with
a yellow humeral two thirds and the
black apex; the pronotum yellow
margins
Body size: ~20 mm

Distribution: the Eastern Andes

Non-lycid co-mimics: not recorded

Remark: an extremely large-bodied
net-winged beetle with very large
dilated elytra, a very rare pattern

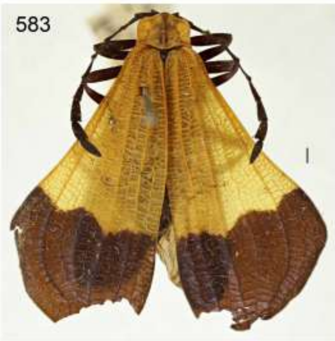

Calopteron sp., Colombia

Bolivia

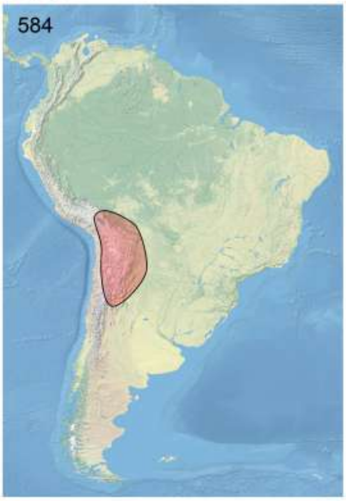

Pattern: bicoloured elytra with yellow humeri
and the yellow transverse band behind
the middle of elytra; the pronotum
yellow margins
Body size: 10-18 mm

Distribution: continental South America

Non-lycid co-mimics: Cantharidae,
Cerambycidae, various moths

Remark: large-bodied net-winged beetles with
dilated elytra, a dominant pattern in the
Neotropical region

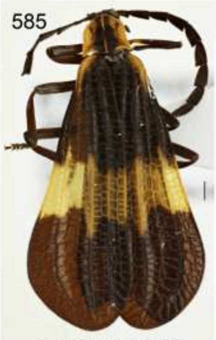

Calopteron sp., Argentina

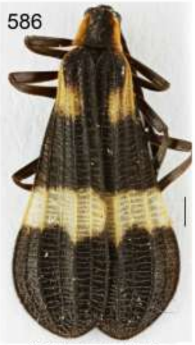

Calopteron sp., Bolivia

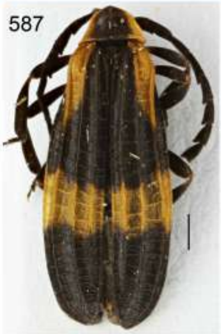

indet. Bolivia

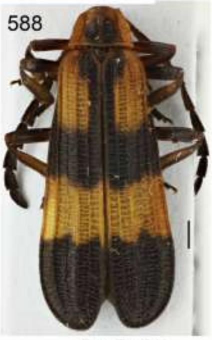

indet. Bolivia

Forms of the basic
banded pattern

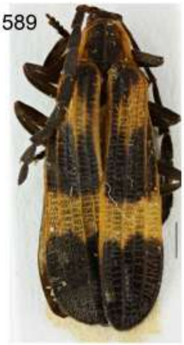

indet. Bolivia

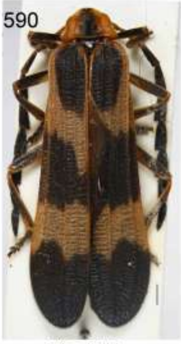

indet., Bolivia

All graphics and text produced by the authors as they are listed under the title of this article (CC-BY open acces license). Long horn beetle photographs taken by L. Dembicky.

Pattern: bicoloured elytra with
yellow humeri and the yellow
apex; the pronotum with a black
patch and yellow margins
Body size: 8-12 mm

Distribution: continental
South America

Non-lycid co-mimics: Cantharidae,
Cerambycidae, various moths
Remark: a pattern common in the
Neotropical region

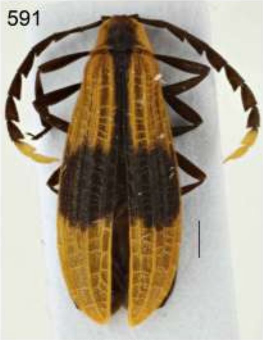

indet., Bolivia

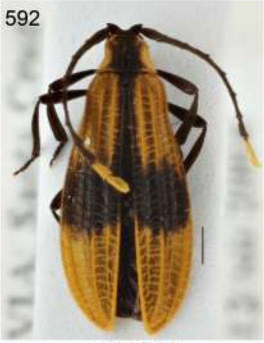

indet., Bolivia

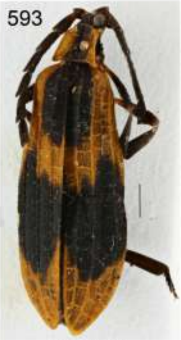

indet. Bolivia

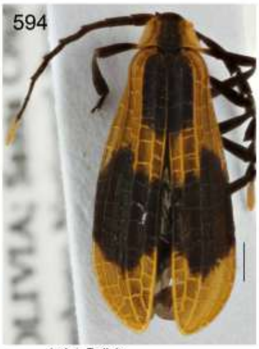

indet. Bolivia

595

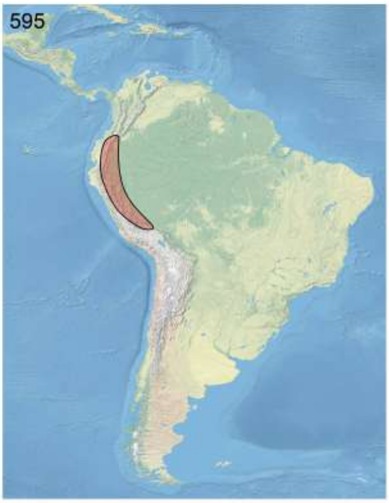

Pattern: bicoloured elytra with
a yellow humeral half and the
black apex; the pronotum with
a median black patch and yellow
margins
Body size: ~5 mm

Distribution: continental
South America

Non-lycid co-mimics:
Cantharidae

Lycinae: Eurrhacini

596

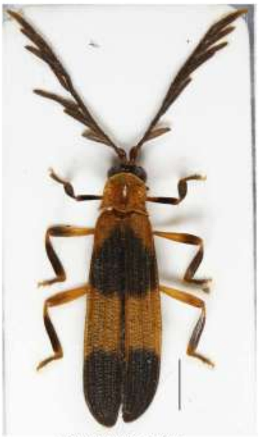

Eurrhacus sp., Peru

Lycinae: Calopterini

597

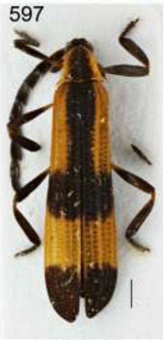

indet., Ecuador

598

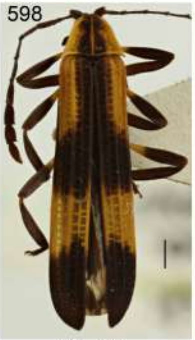

indet., Peru

599

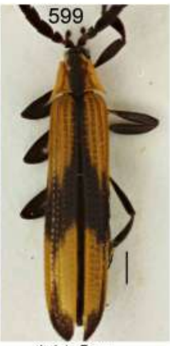

indet., Peru

600

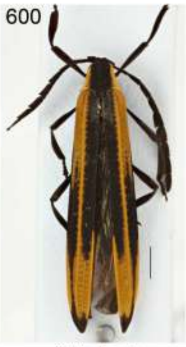

indet., Ecuador

601

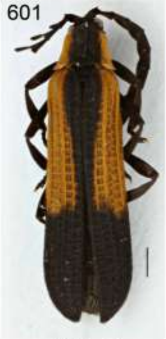

indet., Ecuador

Pattern: bicoloured elytra with
a yellow humeral patches, yellow trasverse
band and the black apex; the pronotum with
a median black patch and yellow margins
Body size: 6-11 mm

Distribution: continental
South America

Non-lycid co-mimics: not recorded

Remark: A groups of species with
a medium-sized and very slender body

Pattern: bicoloured elytra with
a scutellarl patch,and a black trasverse
band, the apex of elytra yellow; the pronotum
with a median black patch and yellow margins

Body size: ~8 mm

Distribution: continental
South America

Non-lycid co-mimics: not recorded

Remark: A species with a medium-sized and
slender body, sympatrically occurring with the
species belonging to the previous pattern

Lycinae: Calopterini

603

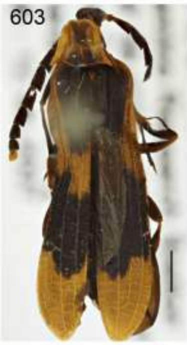

indet., Ecuador

Pattern: bicoloured elytra with long black and
yellow longitudinal stripes; the pronotum black

Body size: ~8 mm

Distribution: continental
South America

Non-lycid co-mimics: not recorded

Remark: A species with a medium-sized
and very slender body. A rare pattern

Lycinae: Platerodini

604

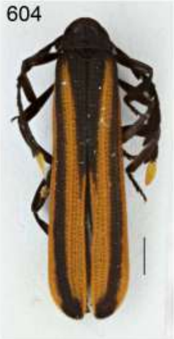

Plateros sp., Ecuador

Pattern: bicoloured elytra with
yellow humeri, yellow trasverse
band and the black apex; the pronotum with
a median black patch and yellow margins
or black
Body size: ~5 mm

Distribution: continental
South America

Non-lycid co-mimics: not recorded

Remark: A groups of species with
a small and wide body

Lycinae: Platerodini

605

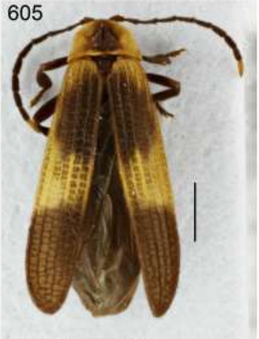

Plateros sp., Bolivia

Lycinae: Calopterini

606

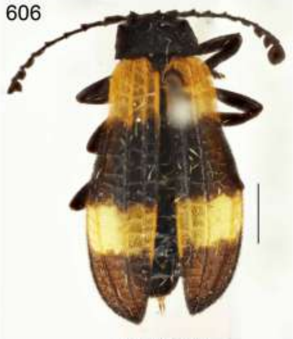

indet., Ecuador

All graphics and text produced by the authors as they are listed under the title of this article (CC-BY open acces license). Long horn beetle photographs taken by L. Dembicky.

Lycinae: Platerodini

Pattern: bicoloured elytra with a brown patch close to the middle part of elytra; the pronotum black or with light yellow margins
Body size: ~5 mm

Distribution: continental South America

Non-lycid co-mimics: not recorded

Remark: A groups of species with a small-sized and relatively robust body, a widespread but uncommon pattern in the Neotropical region

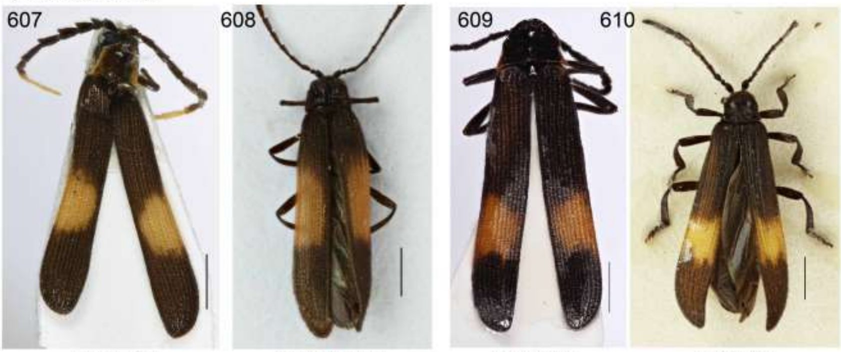

Plateros sp., Peru — *Plateros* sp., Ecuador — *Plateros* sp., Ecuador — *Plateros* sp., Peru

Lycinae: Platerodini

Pattern: bicoloured elytra with a red patch close to the middle part of elytra; the pronotum black
Body size: 6-10 mm

Distribution: the eastern Andes

Non-lycid co-mimics: not recorded

Remark: A groups of species with a small-sized and relatively robust body, a pattern currently known only from Ecuador

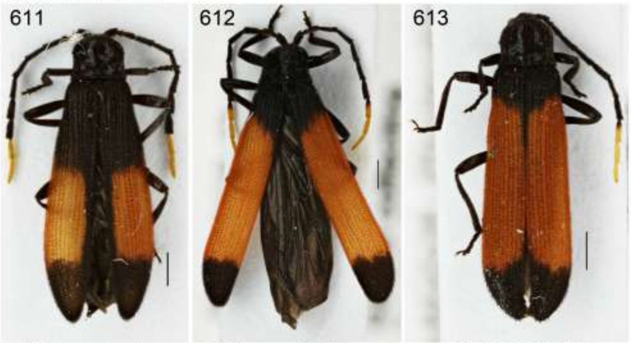

Plateros sp., Ecuador — *Plateros* sp., Ecuador — *Plateros* sp., Ecuador

Lycinae: Platerodini

Pattern: bicoloured elytra with a black scutellar patch and the black apex of elytra; the pronotum with a black patch and light margins
Body size: ~5 mm

Distribution: continental South America

Non-lycid co-mimics: not recorded

Remark: A groups of species with a small-sized and relatively robust body, a widespread pattern

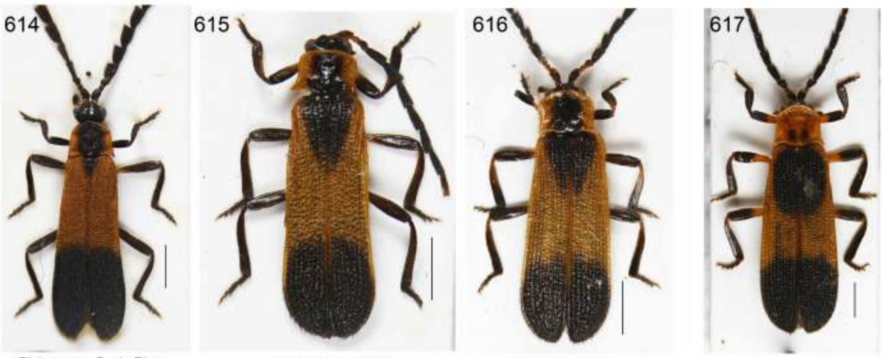

Plateros sp., Costa Rica — *Plateros* sp., Bolivia — *Plateros* sp., Bolivia — *Plateros* sp., Peru

Lycinae: Platerodini

Pattern: black elytra, the pronoutum with a black patch

Body size: ~ 5 mm

Distribution: Mesoamerica and South America
Non-lycid co-mimics: unknown

Remark: A pattern known also from the Nearctic region (SE USA), southern Chia, Vietnam and Taiwan

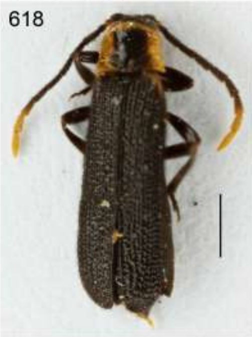

Plateros sp., Bolivia

Lycinae: Platerodini

Pattern: bicoloured elytra with yellow humeri; the pronotum black with light margins
Body size: ~5 mm

Distribution: continental South America

Non-lycid co-mimics: Chrysomelidae

Remark: A groups of species with a small-sized and relatively robust body

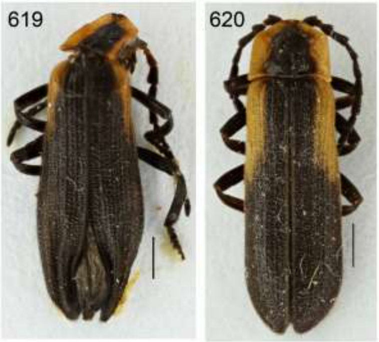

Plateros sp., Bolivia — *Plateros* sp., Bolivia

Lycinae: Platerodini

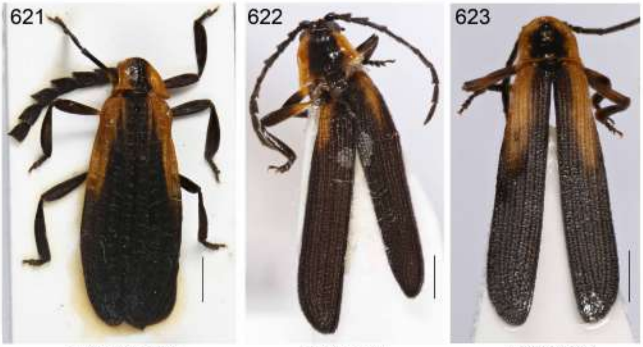

indet., Costa Rica — *Plateros* sp., Peru — *Plateros* sp., Peru

All graphics and text produced by the authors as they are listed under the title of this article (CC-BY open acces license). Long horn beetle photographs taken by L. Dembicky.

Pattern: bicoloured elytra with a black apical part; the pronotum black patch and light margins or uniform yellow

Body size: 12-17 mm

Distribution: continental South America

Non-lycid co-mimics: Cantharidae, Cerambycidae

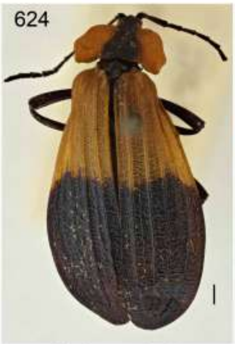

Rhyncheros sp., Ecuador

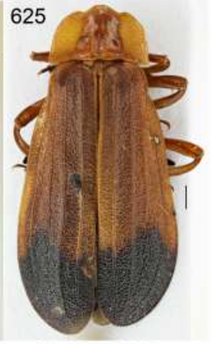

Rhyncheros sp., Bolivia

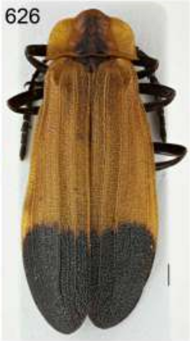

Rhyncheros sp. Peru

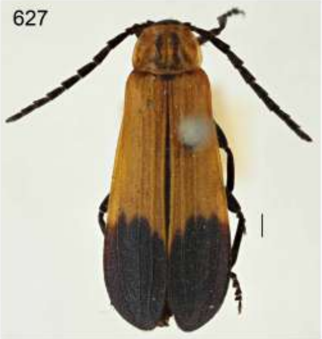

Macrolygistopterus missiorum Berg, Argentina

Pattern: bicoloured elytra with a black humeral patch, sometimes also black apically; the pronotum black patch and light margins or uniform yellow

Body size: 12-17 mm

Distribution: continental South America

Non-lycid co-mimics: Cantharidae, Cerambycidae

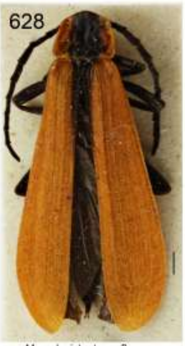

Macrolygistopterus flameus Gorham, Panama

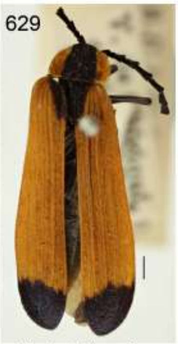

Macrolygistopterus aletus Gorham, Panama

Pattern: bicoloured elytra with red humeri and a blue metallic apical part; the pronotum with black patch and light margins or uniform black

Body size: 12-17 mm

Distribution: continental South America

Non-lycid co-mimics: not recorded

Remark: A rare pattern in high elevations

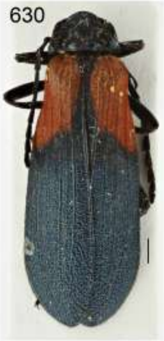

Macrolygistopterus sp., Ecuador

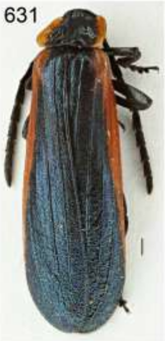

Macrolygistopterus sp., Ecuador

Pattern: black elytra and the red pronotum

Body size: 14 mm

Distribution: Colombia

Non-lycid co-mimics: not recorded

Remark: A rare pattern

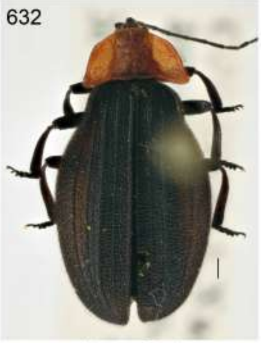

Celiasis sp., Colombia

All graphics and text produced by the authors as they are listed under the title of this article (CC-BY open acces license). Long horn beetle photographs taken by L. Dembicky.

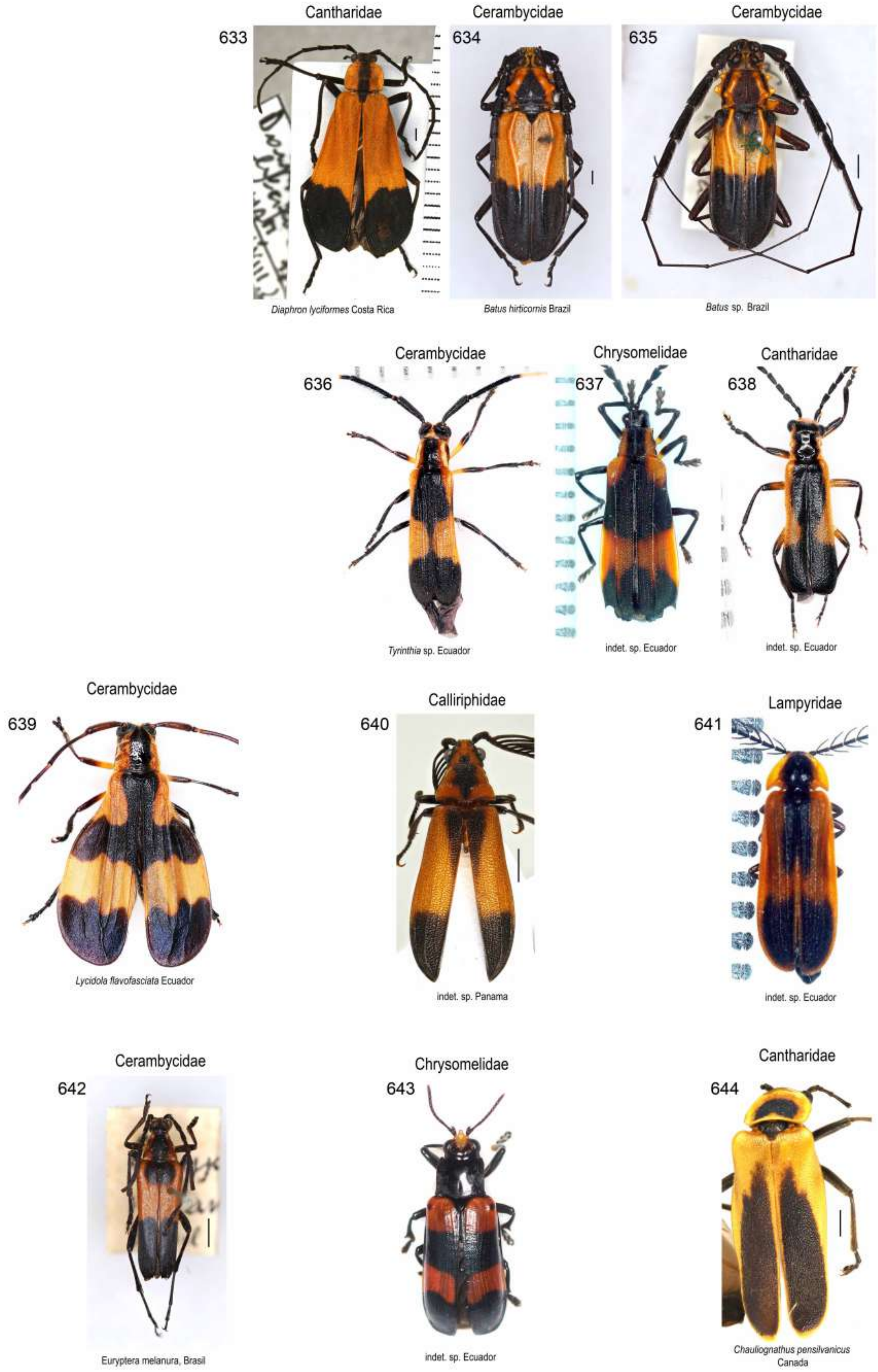

All graphics and text produced by the authors as they are listed under the title of this article (CC-BY open acces license). Long horn beetle photographs taken by L. Dembicky. The photographs of the co-mimics taken by J. McClarin.

Cantharidae

645

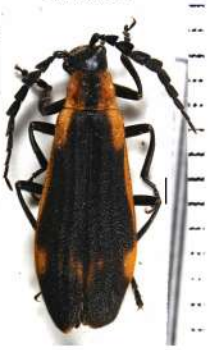

indet. sp. Ecuador

Cantharidae

646

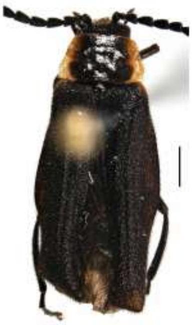

indet. sp. Ecuador

Elateridae

647

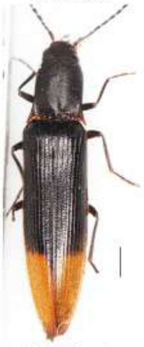

indet. sp. Ecuador

Cleridae

648

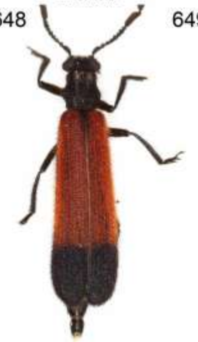

indet. sp. Ecuador

Cantharidae

649

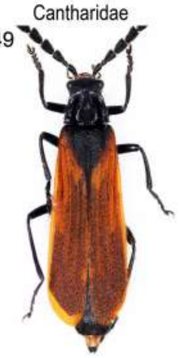

indet. sp. Ecuador

Lycidae co-mimics:
Rhyncheros sanguineus
(Gorham)

Cantharidae

650

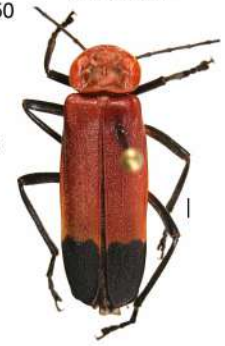

Chauliognathus lecontei, Champion
USA: Arizona

Cerambycidae

651

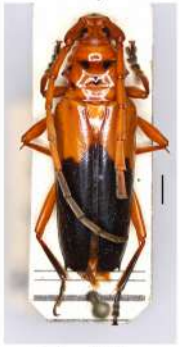

indet. sp. Brazil

Cerambycidae

652

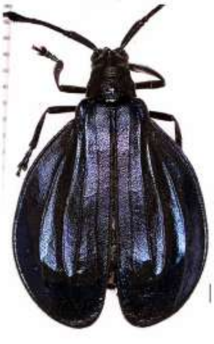

Lycidola felix Ecuador

Cerambycidae

653

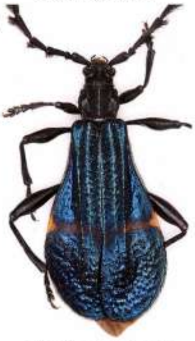

Pteroplatus pulcher Ecuador

All graphics and text produced by the authors as they are listed under the title of this article (CC-BY open acces license). Long horn beetle photographs taken by L. Dembicky. The photographs of the co-mimics taken by J. McClarin.

Northern America

654

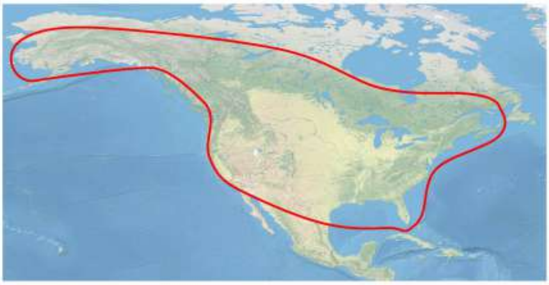

The phylogenetic structure of the Lycidae fauna

Subfamily	Tribe /Subtribe	# spp. (woldwide)	# spp. (region)
Dexorinae	Dexorini	15 spp.	
	Mimolibnetini	5 spp.	
Erotinae	Erotini	54 spp.	8 spp.
	Dictyopterini	73 spp.	5 spp.
	Taphini	31 spp.	
	Slipinskiini	46 spp.	
Calochrominae	Calochromini	289 spp.	13 spp.
Ateliinae	Ateliini	45 spp.	
	Lyponiini	45 spp.	
	Macrolycini	69 spp.	
Lyropaeinae	Lyropaeini	43 spp.	
	Alyculini	5 spp.	
	Antennolycini	3 spp.	
	Platerodrilini	49 spp.	
Lycinae	Conderini	42 spp.	
	Eurrhacini	102 spp.	
	Thonalmini	11 spp.	
	Leptolycini	12 spp.	
	Platerodini	861 spp.	33 spp.
	Calopterini	367 spp.	12 spp.
	Lycini	413 spp.	12 spp.
Metriorrhynchinae	Dihammatini	44 spp.	
	Lycoprogenthini	7 spp.	
	Libnetini	112 spp.	
	Dilophotini	81 spp.	
	Metriorrhynchini	1410 spp.	
	Metanoeina	40 spp.	
	Cautirina	750 spp.	
	Metriorrhynchina	620 spp.	
Total		4234 spp.	83 spp.

The presence of net-winged beetle aposematic patterns in the region

Group	Colour type	Characteristics	+/-	Figures
Uniform coloration	black	pronotum an elytra uniformly black to dark brown	✓	*Plateros* spp. (not shown)
	yellow	pronotum and elytra yellow to light brown	✓	658
	orange and red	pronotum and elytra brightly orange or cinnamon red	✓	e.g. *Dictyoptera simplicipes, D. aurora* (not shown)
	metallic (blue, green)	pronotum and elytra metalic, all shades of colours		
Bi-colored pronotum/ elytra	black/yellow	pronotum black (at most with bright margins), elytra yellow		
	black/red	pronotum black (at most with bright margins), elytra red	✓	*Dictyoptera aurora* (some individuals, not shown)
	bright/black	pronotum brightly colored, elytra uniformly black	✓	660, *Plateros* spp. (not shown)
	red/merallic	pronotum brightly red, elytra metallic blue	✓	*Calopteron* sp. (not shown)
Bi-colored elytra	yellow/black	elytra bi-colored. humeral part yellow, apical part dark coloured	✓	456
	red(orange)/black	elytra bi-colored. humeral part orange/red, apical part dark	✓	*Lygistopterus slevini* (not shown)
	black/bright	elytra bi-colored. humeral part black, apex yellow/orange/red		
Fasciate elytra	yellow/black	humeri and apex of elytra black, middle of elytron yellow	✓	*Calopteron reticulatum* (not shown)
	bright/black	humeri and apex of elytra brigt, middle of elytron black		
	yellow/metallic	most elytra black with blue metallic shine, middle yellow		
Striate elytra	bright/black	elytra brown/brightly coloured, suture or middle of elytron black		
Punctate el.	bright/black	elytra brown/brightly coloured, black patch in each elytron		
Tri-colored el.	all combinations of colors	elytron with three differently coloured parts		
Reticulate	bright/black	backgroud colour of elytra dark, costae large, brightly coloured		
Non-categorized aposematic patterns		see listed examples for further information		

Figures S654–S660. The overview of the lycid fauna, coloration, and co-mimics: Nearctic region.

All graphics and text produced by the authors as they are listed under the title of this article (CC-BY open acces license). Long horn beetle photographs taken by L. Dembicky.

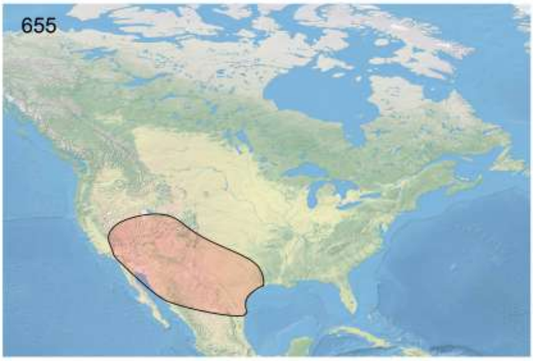

Pattern: uniform orange to red elytra with apical patch; the pronotum sometimes uniform bright

Body size: 12-16 mm

Distribution: the southwest USA

Non-lycid co-mimics: Cantharidae, Cerambycidae

Remark. A widespread pattern of large bodied lycids, *Neolycus* commonly form aggregations on flowers and fly in open situations. The Nearctic species have been placed in Lycus due to their superficial similarity (Kusy *et al.* in press)

Lycinae: Lycini

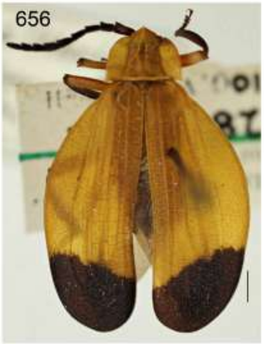

Neolycus arizonensis, USA

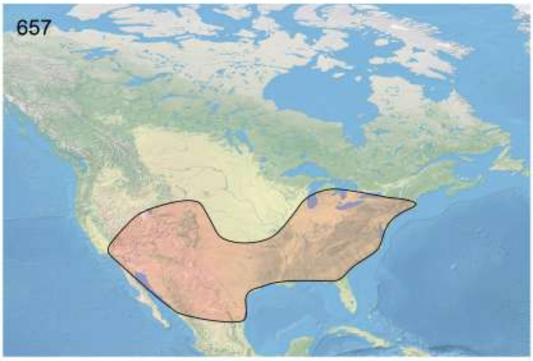

Pattern: uniform orange elytra and the pronotum

Body size: 10-14 mm

Distribution: the southwest USA

Non-lycid co-mimics: Cantharidae, Cerambycidae

Remark. A widespread pattern of large bodied lycids, *Rhyncheros* commonly form aggregations on flowers and fly in open situations
Some species of Rhyncheros have apical part of the elytra black

Lycinae: Lycini

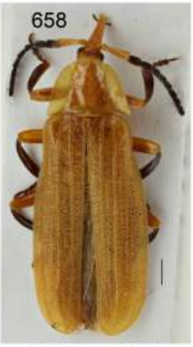

Rhyncheros sp., USA

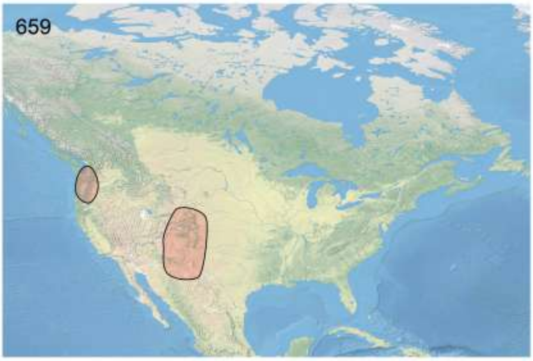

Pattern: uniform black and slightly blue metallic elytra and the uniform red pronotum

Body size: 10-12 mm

Distribution: the southwest USA

Non-lycid co-mimics: not recorded

Remark. A rare aposematic pattern, currently without known co-mimics.
A similar combination of bright pronotum and black elytra was recorded in Lycini in Colombia (see South American patterns below) and in the Dictyopterini in Japan (Nakane 1969).

Calochrominae: Calochromini

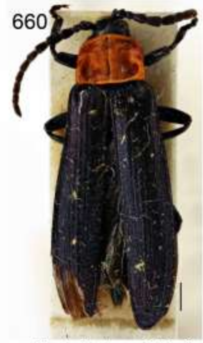

Macrolygistopterus ruficolis LeConte
USA (SW)

Futherr patterns known from North America:

Calopteron spp. fasciate elytra Distribution: The eastern and southern regions of the USA, southern Canada

Plateros spp., *Macrolygistopterus* spp. black elytra, the pronotum with brightly coloured apical margins. Distribution: the souteast of the USA

Dictyoptera spp., Uniform red coloured elytra and the pronotum. Distribution: humid forest regions of the USA and southern Canada

Macrolygistopterus rubripennis LeConte red coloured alytra and the black pronotum. Distribution: Colorado, Arizona, New Mexico

All graphics and text produced by the authors as they are listed under the title of this article (CC-BY open acces license). Long horn beetle photographs taken by L. Dembicky.

Australian region: Wallacea

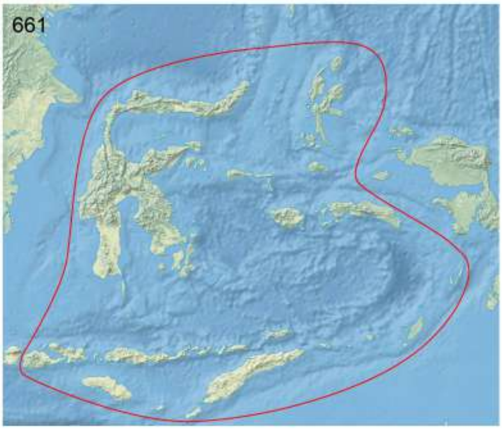

Sulawesi

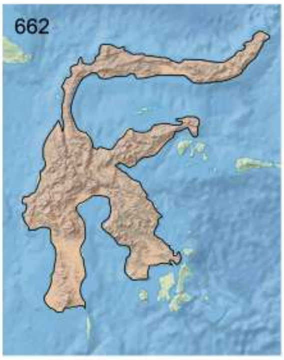

The phylogenetic structure of the Lycidae fauna

Subfamily	Tribe /Subtribe	# spp. (woldwide)	# spp.(Sulawesi)
Dexorinae	Dexorini	15 spp.	
	Mimolibnetini	5 spp.	
Erotinae	Erotini	54 spp.	
	Dictyopterini	73 spp.	
	Taphini	31 spp.	1 sp.
	Slipinskiini	46 spp.	
Calochrominae	Calochromini	289 spp.	3 spp.
Ateliinae	Ateliini	45 spp.	
	Lyponiini	45 spp.	
	Macrolycini	69 spp.	
Lyropaeinae	Lyropaeini	43 spp.	5 spp.
	Alyculini	5 spp.	
	Antennolycini	3 spp.	
	Platerodrilini	49 spp.	
Lycinae	Conderini	42 spp.	
	Eurrhacini	102 spp.	
	Thonalmini	11 spp.	
	Leptolycini	12 spp.	
	Platerodini	861 spp.	23 spp.
	Calopterini	367 spp.	
	Lycini	413 spp.	3 spp.
Metriorrhynchinae	Dihammatini	44 spp.	
	Lycoprogenthini	7 spp.	
	Libnetini	112 spp.	
	Dilophotini	81 spp.	
	Metriorrhynchini	1410 spp.	
	Metanoeina	40 spp.	
	Cautirina	750 spp.	5 spp.
	Metriorrhynchina	620 spp.	80 spp.
Total		4234 spp.	120 spp.

The presence of net-winged beetle aposematic patterns in the region

Group	Colour type	Characteristics	+/-	Examples
Uniform coloration	black	pronotum an elytra uniformly black to dark btown	✓	666-668
	yellow	pronotum and elytra yellow to light brown	✓	674, 675
	orange and red	pronotum and elytra brightly orange or cinnamon red	✓	677
	metallic (blue, green)	pronotum and elytra metalic, all shades of colours		
Bi-colored pronotum/ elytra	black/yellow	pronotum back (at most with bright margins), elytra yellow		
	black/red	pronotum black (at most with bright margins), elytra red		
	bright/black	pronotum brightly colored, elytra uniformly black		
	red/merallic	pronotum brightly red, elytra metallic blue	✓	663-665
Bi-colored elytra	yellow/black	elytra bi-colored. humeral part yellow, apical part dark coloured	✓	*Leptotrichalus* spp., *Lobatang* sp. (not shown)
	red(orange)/black	elytra bi-colored. humeral part orange/red, apical part dark	✓	*Coloberos* sp. (not shown)
	black/bright	elytra bi-colored. humeral part black, apex yellow/orange/red		
Fasciate elytra	yellow/black	humeri and apex of elytra black, middle of elytron yellow	✓	669-673
	bright/black	humeri and apex of elytra brigt, middle of elytron black		
	yellow/metallic	most elytra black with blue metallic shine, middle yellow		
Striate elytra	bright/black	elytra brown/brightly coloured, suture or middle of elytron black		
Punctate el.	bright/black	elytra brown/brightly coloured, black patch in each elytron		
Tri-colored el.	all combinations of colors	elytron with three differently coloured parts		
Reticulate	bright/black	backgroud colour of elytra dark, costae large, brightly coloured		
Non-categorized aposematic patterns		see listed examples for further information		

Figures S661–S655. The overview of the lycid fauna, coloration, and co-mimics: Australian region, Wallacea.

All graphics and text produced by the authors as they are listed under the title of this article (CC-BY open acces license). Long horn beetle photographs taken by L. Dembicky.

Pattern: red pronotum, metallic elytra

Body size: 8-20 mm

Distribution:
Sulawesi

Lycidae-comimics:
Plateros sp., *Calochromus* sp.

Non-lycid co-mimics: Eucnemidae

M.:M.:Metriorrhynchina

663

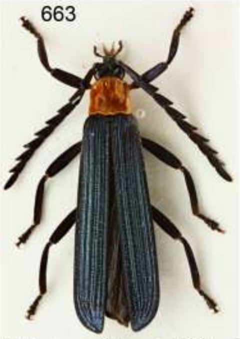

Metriorrhyncus cf. thoracicus F., Sulawesi

Calochromine: Calochromini

664

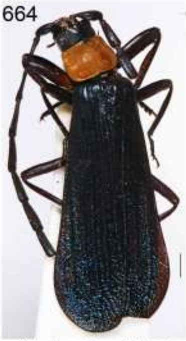

Calochromus toxopei, Sulawesi

665

broxylus

Metriorrhynchinae: Metriorrhynchini: Metriorrhynchina

Pattern: uniform black

Body size: ~ 6 mm

Distribution:
Sulawesi

Lycidae-co-mimics:
Plateros spp., *Sulabanus* spp.

Non-lycid co-mimics:
Cantharidae

666

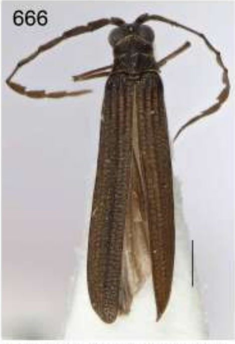

Wakarumbia linearis Dvor. et Boc., Sulawesi

667

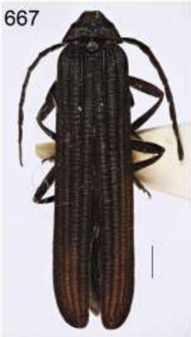

Sulabanus sp., Sulawesi

668

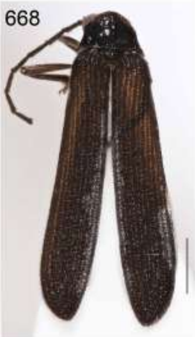

Plateros sp., Sulawesi

Metriorrhynchinae: Metriorrhynchini: Metriorrhynchina

Pattern: fasciate elytra

Body size: ~6 mm

Distribution:
Sulawesi

Lycidae-co-mimics:
Plateros spp., *Sulabanus* spp.

Non-lycid co-mimics:
Cantharidae

669

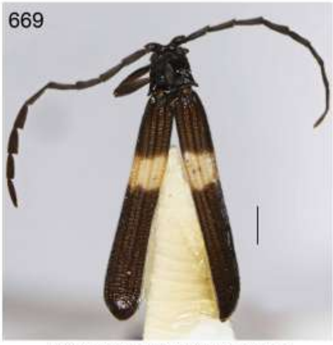

Wakarumbia pendolensis Dvor. et Boc., Sulawesi

670

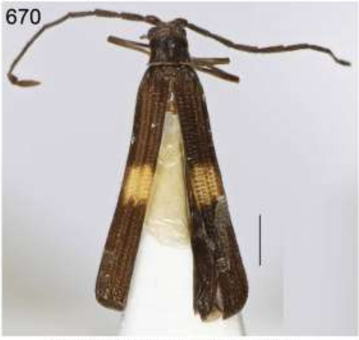

Wakarumbia mamasensis Dvor. et Boc., Sulawesi

671

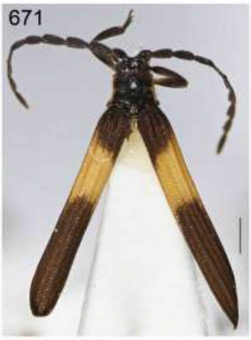

Wakarumbia pseudofasciata Dvor. et Boc., Sulawesi

672

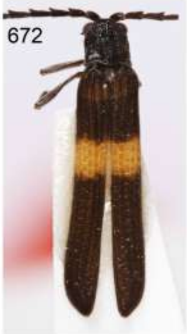

Sulabanus robustus Dvor. et Boc., Sulawesi

673

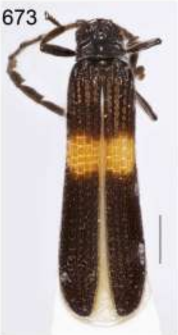

S. similis Dvor. et Boc., Sulawesi

Pattern: uniform yellow

Body size: ~6 mm

Distribution:
Sulawesi

Lycidae-co-mimics:
Plateros sp., *Xylobanus* sp.

Non-lycid co-mimics:
Cantharidae

Metr.:Metr.: Metriorrhynchina

674

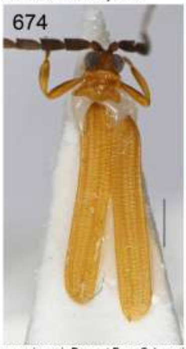

Wakarumbia amporiwensis Dvor. et Boc., Sulawesi

Lycinae: Lycini

675

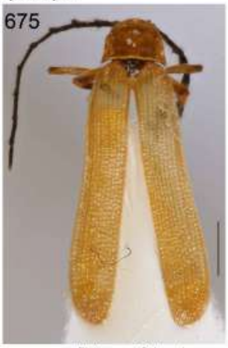

Plateros sp., Sulawesi

Pattern: light brown

Body size: ~4 mm

Distribution:
Sulawesi

Lycidae-co-mimics:
none

Non-lycid co-mimics:
-

Metr.:Metr.: Metriorrhynchina

676

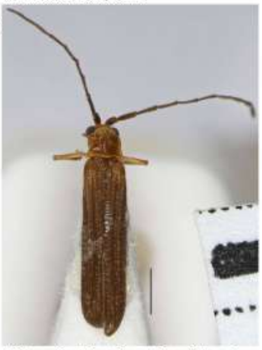

Wakarumbia obstinata Dvor. et Boc., Sulawesi

Pattern: uniform yellow with a black patch in the pronotum and light margins

Body size: 18-23 mm

Distribution:
Sulawesi

Further Lycidae co-mimics: none

Non-lycid co-mimics:
Remark: flower visiting, forms aggregations,

Lycinae: Lycini

677

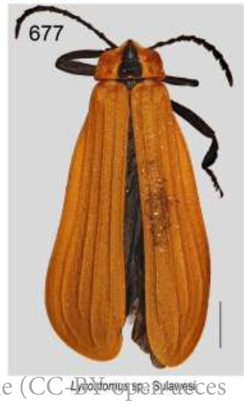

Lycodomus sp., Sulawesi

All graphics and text produced by the authors as they are listed under the title of this article (CC-BY open access license). Long horn beetle photographs taken by L. Dembicky.

678

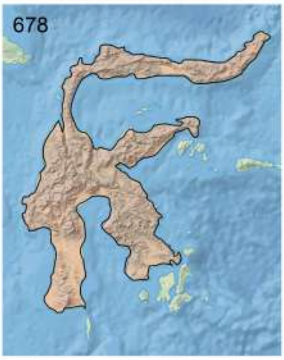

Cantharidae

679

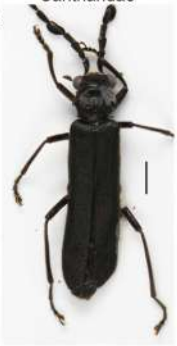

indet., Sulawesi

Eucnemidae

680

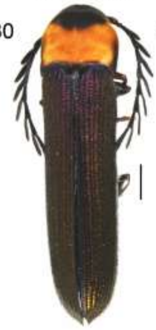

indet., Sulawesi

Elateridae

681

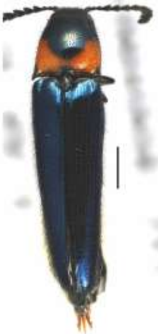

indet., Sulawesi

Chrysomelidae

682

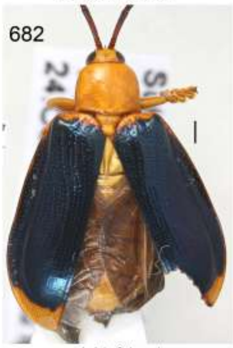

indet., Sulawesi

Cleridae

683

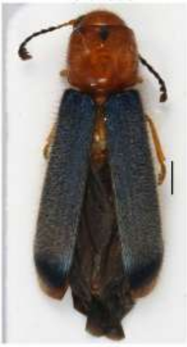

indet., Sulawesi

Chrysomelidae

684

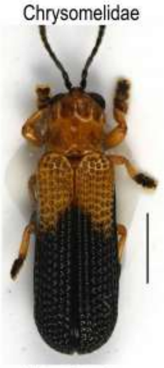

indet. sp. Sulawesi

Eucnemidae

685

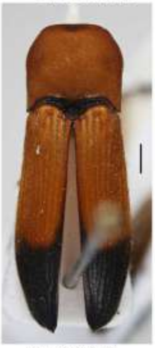

indet. sp. Sulawesi

All graphics and text produced by the authors as they are listed under the title of this article (CC-BY open acces license). Long horn beetle photographs taken by L. Dembicky.

Australian Region

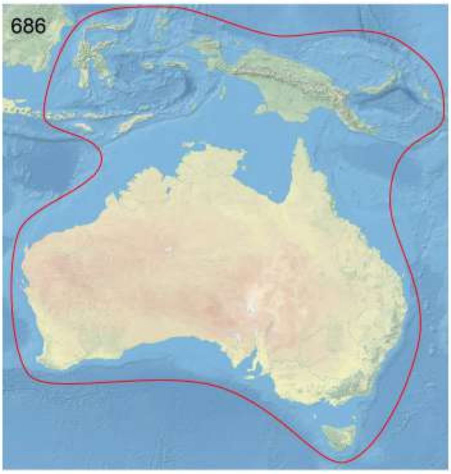

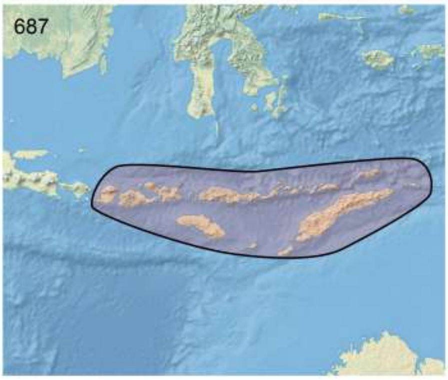

The phylogenetic structure of the Lycidae fauna

Subfamily	Tribe /Subtribe	# spp. (woldwide)	# spp. (region)
Dexorinae	Dexorini	15 spp.	
	Mimolibnetini	5 spp.	
Erotinae	Erotini	54 spp.	
	Dictyopterini	73 spp.	1 sp.
	Taphini	31 spp.	
	Slipinskiini	46 spp.	
Calochrominae	Calochromini	289 spp.	5 spp.
Ateliinae	Ateliini	45 spp.	
	Lyponiini	45 spp.	
	Macrolycini	69 spp.	
Lyropaeinae	Lyropaeini	43 spp.	1 sp.
	Alyculini	5 spp.	
	Antennolycini	3 spp.	
	Platerodrilini	49 spp.	
Lycinae	Conderini	42 spp.	1 sp.
	Eurrhacini	102 spp.	
	Thonalmini	11 spp.	
	Leptolycini	12 spp.	
	Platerodini	861 spp.	~20 spp.
	Calopterini	367 spp.	
	Lycini	413 spp.	
Metriorrhynchinae	Dihammatini	44 spp.	
	Lycoprogenthini	7 spp.	
	Libnetini	112 spp.	
	Dilophotini	81 spp.	
	Metriorrhynchini	1410 spp.	
	Metanoeina	40 spp.	
	Cautirina	750 spp.	3 spp.
	Metriorrhynchina	620 spp.	~30 spp.
Total		4234 spp.	~60 spp.

The presence of net-winged beetle aposematic patterns in the region

Group	Colour type	Characteristics	+/-	Figures
Uniform coloration	black	pronotum an elytra uniformly black to dark btown		
	yellow	pronotum and elytra yellow to light brown	✓	692
	orange and red	pronotum and elytra brightly orange or cinnamon red		
	metallic (blue, green)	pronotum and elytra metalic, all shades of colours		
Bi-colored pronotum/ elytra	black/yellow	pronotum back (at most with bright margins), elytra yellow		
	black/red	pronotum black (at most with bright margins), elytra red		
	bright/black	pronotum brightly colored, elytra uniformly black	✓	689-691
	red/merallic	pronotum brightly red, elytra metallic blue		
Bi-colored elytra	yellow/black	elytra bi-colored. humeral part yellow, apical part dark coloured	✓	*Leptotrichalus* spp., *Trichalus communis* (not shown)
	red(orange)/black	elytra bi-colored. humeral part orange/red, apical part dark		
	black/bright	elytra bi-colored. humeral part black, apex yellow/orange/red		
Fasciate elytra	yellow/black	humeri and apex of elytra black, middle of elytron yellow		
	bright/black	humeri and apex of elytra brigt, middle of elytron black		
	yellow/metallic	most elytra black with blue metallic shine, middle yellow		
Striate elytra	bright/black	elytra brown/brightly coloured, suture or middle of elytron black		
Punctate el.	bright/black	elytra brown/brightly coloured, black patch in each elytron		
Tri-colored el.	all combinations of colors	elytron with three differently coloured parts		
Reticulate	bright/black	backgroud colour of elytra dark, costae large, brightly coloured		
Non-categorized aposematic patterns		see listed examples for further information	✓	688

All graphics and text produced by the authors as they are listed under the title of this article (CC-BY open acces license). Long horn beetle photographs taken by L. Dembicky.

Pattern: bi-coloured elytra
black humeri, testaceous apex

Body size: ~ 6 mm

Distribution:
Lombok

Lycidae co-mimics:
none

Non-lycid co-mimics:
-

Lyropaeinae: Platerodrilini

688

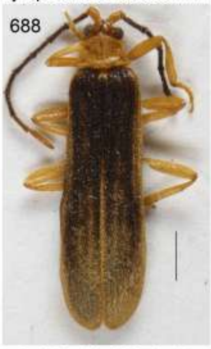

Pendola lombokiensis Boc., Lombok

Pattern: brown pronotum and
black elytra

Body size: 4-6 mm

Distribution:
Lombok, Sumbawa, Sumba
(a pattern widespread in Papua)

Non-lycid co-mimics:
-

Lycinae: Platerodini

689

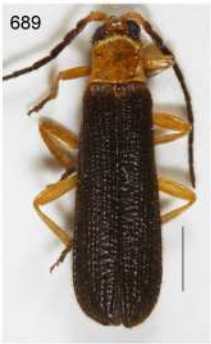

Plateros sp., Lombok

690

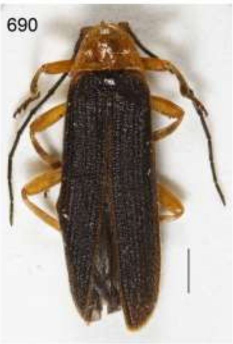

Plateros sp., Lombok

691

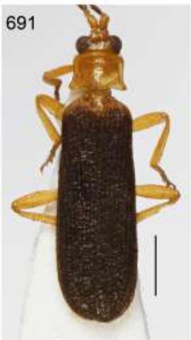

Plateros sp., Sumba

Metriorrhynchinae: Metriorrhynchini: Cautirina

Pattern: bicoloured testaceous and black
elytra; the pronotum black or dark
coloured, pronotal margins light coloured

Body size: 8-10 mm

Distribution: the Lesser Sundas

Non-lycid co-mimics:
Cantharidae

Remark. The medium-bodied lycids resembling
net-winged beetle common in eastern Java

692

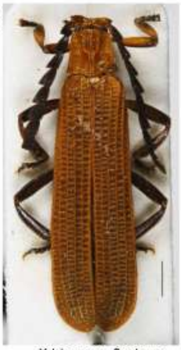

Xylobanus sp., Sumbawa

Remark. Further pattern are represented by yellow/black *Leptotrichalus* spp. (Metriorrhynchina) and *Trichalus* sp. (*T. communis* Waterhouse and relatives).
These species have yellow pronotum and basal 3/4 of elytra; the apex of elytra is dark coloured.

All graphics and text produced by the authors as they are listed under the title of this article (CC-BY open acces license). Long horn beetle photographs taken by L. Dembicky.

Australian Region

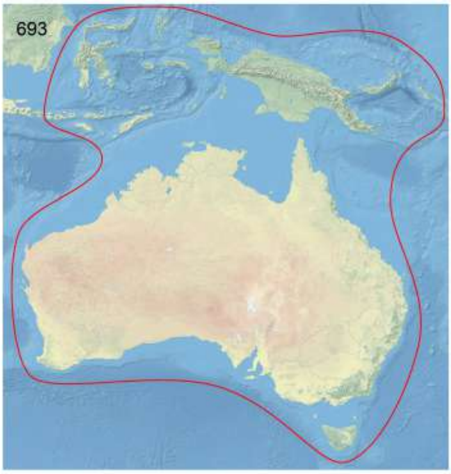

New Guinea and adjacent islands

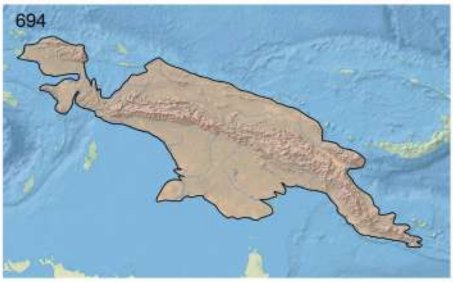

Phylogenetic structure of the fauna

Subfamily	Tribe /Subtribe	# spp. (woldwide)	# spp. (region)
Dexorinae	Dexorini	15 spp.	
	Mimolibnetini	5 spp.	
Erotinae	Erotini	54 spp.	
	Dictyopterini	73 spp.	
	Taphini	31 spp.	1 sp.
	Slipinskiini	46 spp.	
Calochrominae	Calochromini	289 spp.	10 spp.
Ateliinae	Ateliini	45 spp.	
	Lyponiini	45 spp.	
	Macrolycini	69 spp.	
Lyropaeinae	Lyropaeini	43 spp.	
	Alyculini	5 spp.	
	Antennolycini	3 spp.	
	Platerodrilini	49 spp.	
Lycinae	Conderini	42 spp.	
	Eurrhacini	102 spp.	
	Thonalmini	11 spp.	
	Leptolycini	12 spp.	
	Platerodini	861 spp.	50 spp.
	Calopterini	367 spp.	
	Lycini	413 spp.	
Metriorrhynchinae	Dihammatini	44 spp.	
	Lycoprogenthini	7 spp.	
	Libnetini	112 spp.	
	Dilophotini	81 spp.	
	Metriorrhynchini	1410 spp.	
	Metanoeina	40 spp.	
	Cautirina	750 spp.	
	Metriorrhynchina	620 spp.	~280 spp.*
Total		4234 spp.	~340 spp.

The presence of net-winged beetle aposematic patterns in the region

Group	Colour type	Characteristics	+/-	Examples
Uniform coloration	Black	pronotum an elytra uniformly black to dark brown	✓	696-705, 791, 793, 794
	Yellow	pronotum and elytra yellow to light brown	✓	712-718
	Orange and Red	pronotum and elytra brightly orange or cinnamon red	✓	787
	Metallic (blue, green)	pronotum and elytra metalic, all shades of colours	✓	802-804
Bi-colored pronotum/ elytra	black/yellow	pronotum black (at most with bright margins), elytra yellow	✓	812
	black/red	pronotum black (at most with bright margins), elytra red		
	bright/black	pronotum brightly colored, elytra uniformly black	✓	724-734
	red/merallic	pronotum brightly red, elytra metallic blue		
Bi-colored elytra	yellow/black	elytra bi-colored. humeral part yellow, apical part dark coloured	✓	720-735, 737-744, 750-761, 765-774
	red(orange)/black	elytra bi-colored. humeral part orange/red, apical part dark	✓	746-748, 762, 763
	black/bright	elytra bi-colored. humeral part black, apex yellow/orange/red	✓	777, 780-786
Fasciate elytra	yellow/black	humeri and apex of elytra black, middle of elytron yellow	✓	706-708
	bright/black	humeri and apex of elytra brigt, middle of elytron black	✓	814
	yellow/metallic	most elytra black with blue metallic shine, middle yellow		
Striate elytra	bright/black	elytra brown/brightly coloured, suture or middle of elytron black		
Punctate el.	bright/black	elytra brown/brightly coloured, black patch in each elytron	✓	797
Tri-colored el.	all combinations of colors	elytron with three differently coloured parts	✓	*Cladophorus psittacinus* (not shown)
Reticulate	bright/black	backgroud colour of elytra dark, costae large, brightly coloured		
Non-categorized aposematic patterns		see listed examples for further information	✓	709, 710, 776, 778, 790, 800, 806-809, 811

*Most New Guinean speciesw have not yet been described and about a thousand of species is available in collections

Figures S656–S844. The overview of the lycid fauna, coloration, and co-mimics: Australian region, Lesser Sunda Islands, New Guinea.

All graphics and text produced by the authors as they are listed under the title of this article (CC-BY open acces license). Long horn beetle photographs taken by L. Dembicky.

New Guinea and adjacent islands

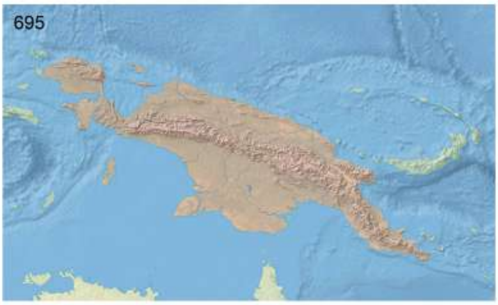

Metriorrhynchinae: Metriorrhynchini: Metriorrhynchina

Pattern: uniform black

Body size: 4.5-12 mm

Distribution: New Guinea

Non-lycid co-mimics:
Cantharidae

Remark. Uniform black pattern is common in the whole region and contains small- to large bodied species. Usually, small bodied species dominate on leaves of low fores canopy.

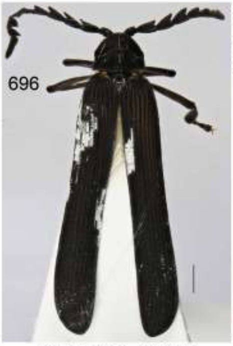

Metriorrhynchus sp., New Guinea

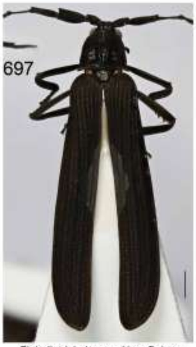

Flabellotrichalus sp., New Guinea

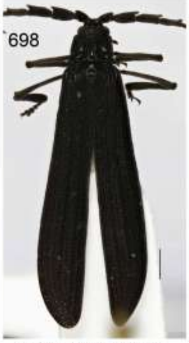

Diatrichalus sp., New Guinea

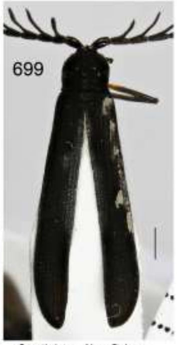

Carathrix sp., New Guinea

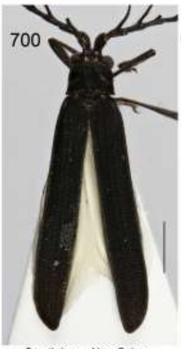

Carathrix sp., New Guinea

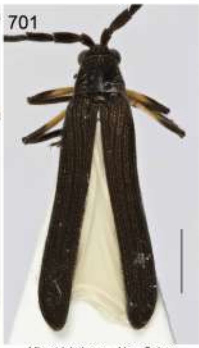

Microtrichalus sp,, New Guinea

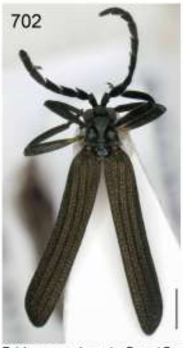

Eniclases pseudoapertus Boc. et Boc. New Guinea

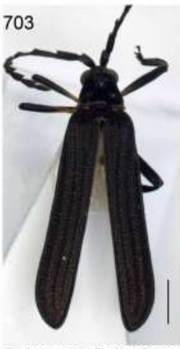

Eniclases apertus Pic, New Guinea

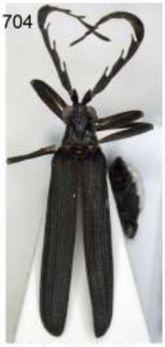

Eniclases niger Boc. et Boc., New Guinea

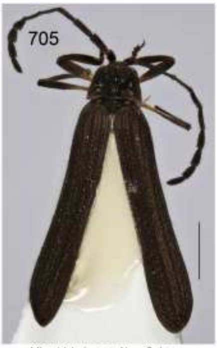

Microtrichalus sp,, New Guinea

Pattern: black pronotum, black elytra with a bright transverse patch

Body size: 8-12 mm

Distribution: New Guinea

Further Lycidae co-mimics:
Metriorrhynchus spp.

Non-lycid co-mimics:
Cantharidae

Remark: rare pattern with variable conspicuousness of the patch

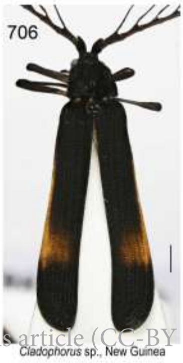

Cladophorus sp., New Guinea

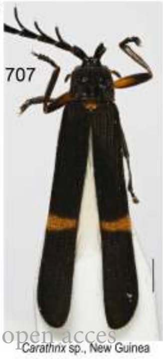

Carathrix sp., New Guinea

All graphics and text produced by the authors as they are listed under the title of this article (CC-BY open acces license). Long horn beetle photographs taken by L. Dembicky.

Pattern: black pronotum, black elytra with a transwerse patch

Body size: ~10 mm

Remark: as previous, but with different shape and position of the patch

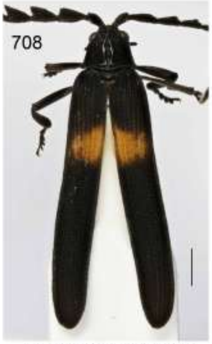

Metriorrhynchus sp., New Guinea

Pattern: black pronotum, black elytra with a sutural patch

Body size: ~10 mm

Remark: as previous, but with different shape and position of the patch

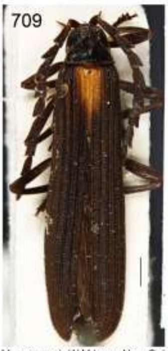

M. papuensis W.M.Leay, New Guinea

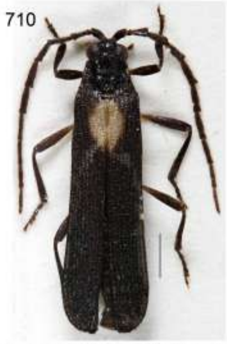

Plateros serratus Bocakova, New Guinea

New Guinea and adjacent islands

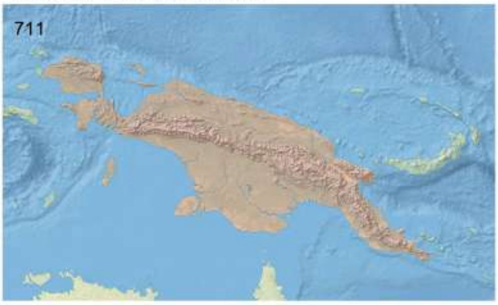

Pattern: uniform yellow
Body size: 6-20 mm

Distribution: New Guinea

Non-lycid co-mimics:
Cantharidae

Remark: Common in the whole area, includes medium- to large-bodied forms

Metriorrhynchinae: Metriorrhynchini: Metriorrhynchina

Large-bodied *Metriorrhynchus* spp. most common in lowlands, commonly flying outside forest canopy. Some species with metallic abdomen, conspicuous when the beetle flies.

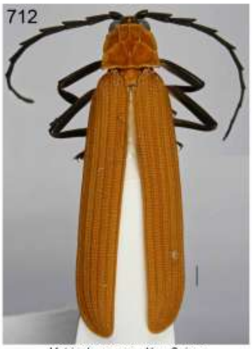

Metriorrhyncus sp., New Guinea

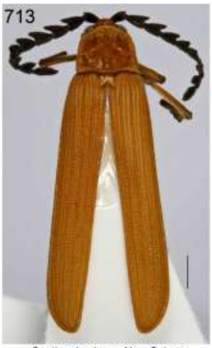

Cautirominmis sp., New Guinea

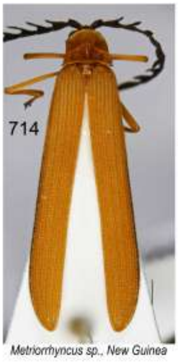

Metriorrhyncus sp., New Guinea

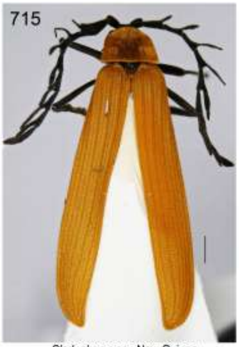

Cladophorus sp., New Guinea

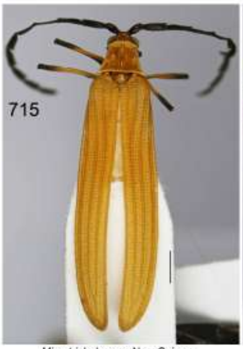

Microtrichalus sp., New Guinea

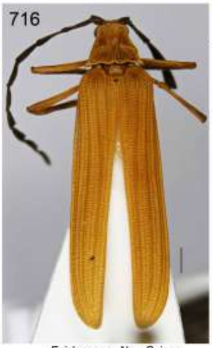

Eniclases sp., New Guinea

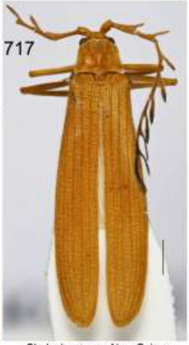

Cladophorus sp., New Guinea

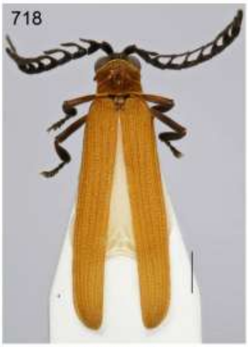

indet., New Guinea

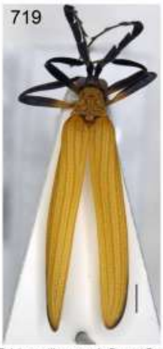

Eniclases tikapurensis Boc. et Boc.
New Guinea

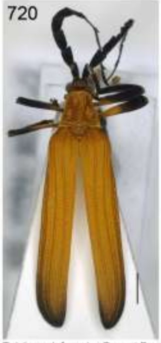

Eniclases infuscatus Boc. et Boc.
New Guinea

Wallacea: Ceram

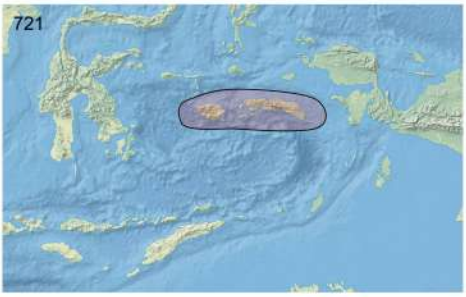

Pattern: bicoloured elytra with a black apical part; the pronotum orange

Body size: ~ 14 mm

Distribution: Ceram

Non-lycid co-mimics:
Cantharidae, Cerambycidae, Chrysomelidae

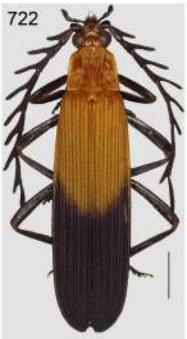

Metriorrhynchus doleschali Redtenb.
Moluccas

All graphics and text produced by the authors as they are listed under the title of this article (CC-BY open acces license). Long horn beetle photographs taken by L. Dembicky.

New Guinea and adjacent islands

723
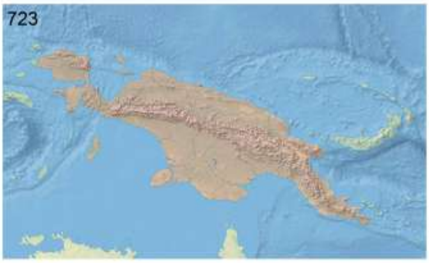

Pattern: bright pronotum, black elytra

Body size: 4-10 mm

Distribution: New Guinea

Further Lycidae co-mimics: *Metriorrhynchus* spp.

Non-lycid co-mimics:
Cantharidae, Lampyridae

Remark: very common pattern, especially in lowland habitats

Metriorrhynchinae: Metriorrhynchini: Metriorrhynchina

724
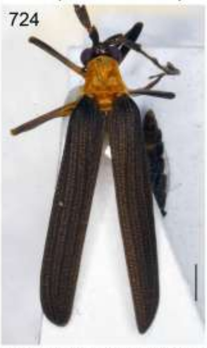
Eniclases niger Boc. et Boc., New Guinea

725
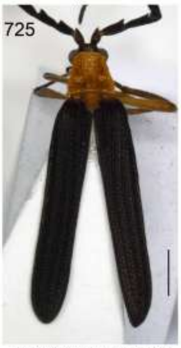
Eniclases brancuccii Boc. et Boc., New Guinea

726
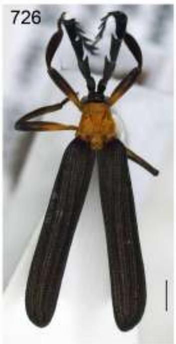
Eniclases elelimensis Boc. et Boc. New Guinea

727
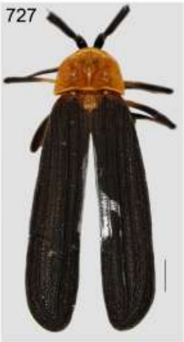
Synchonnus testaceithorax Pic, New Guinea

Lycinae: Platerodini

727
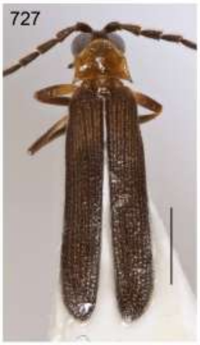
Plateros sp. New Guinea

728
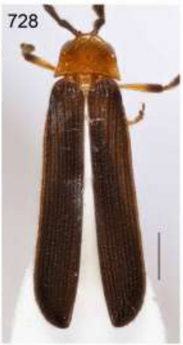
Plateros sp. New Guinea

729
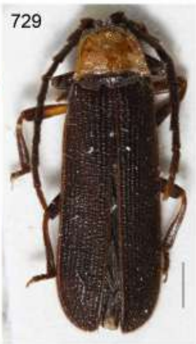
Plateros dimidiatus Bocakova, New Guinea

730
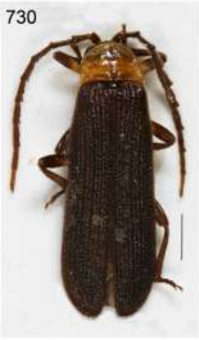
Plateros infusus Bocakova, New Guinea

731
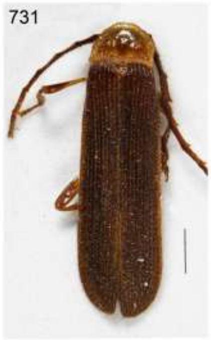
Plateros spinosus Bocakova, New Guinea

Pattern: bright pronotum, black elytra

Body size: 4-10 mm

Distribution: New Guinea

Remark: an example of transitional forms between uniform dark coloured and bright coloured pronotum, various types of pronotal colouration occur syntopically and within a single aggreagtion

Metriorrhynchinae: Metriorrhynchini: Metriorrhynchina

732
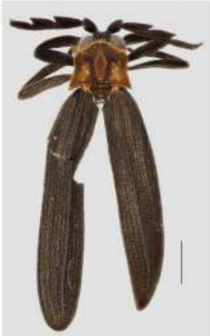
Synchonnus [illegible] Kusy, New Guinea

733
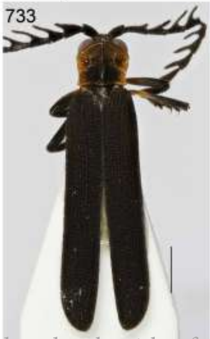
Metriorrhynchus sp., New Guinea

Lycinae: Platerodini

734
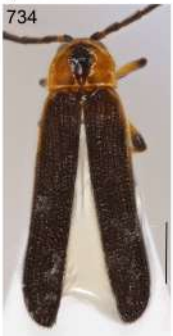
Plateros sp. New Guinea

All graphics and text produced by the authors as they are listed under the title of this article (CC-BY open acces license). Long horn beetle photographs taken by L. Dembicky.

Pattern: bright pronotum, bright humeri, a black posterior part of elytra

Body size: 8-15 mm

Distribution: New Guinea, lowlands

Non-lycid co-mimics:
Cantharidae, Lampyridae

Remark: Calochromus is rare in New Guinea and only distantly resembles other net-winged beetles in the islands. Calochromus flies outside canopy and visits flowers, its interaction with other net-winged beetles is limited.

Calochrominae: Calochromini

735
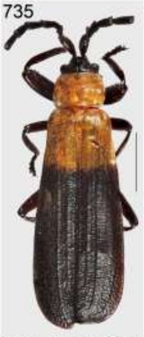
Calochromus xxx, New Guinea

New Guinea and adjacent islands

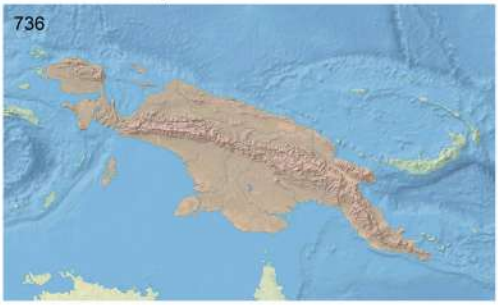

Metriorrhynchinae: Metriorrhynchini: Metriorrhynchina

Pattern: bicoloured elytra
pronotum and elytral humeri yellow

Body size: ~ 8 mm

Distribution: New Guinea

Non-lycid co-mimics: Cantharidae, Chrysomelidae

Remark: common and widespread pattern known also from the Moluccas.

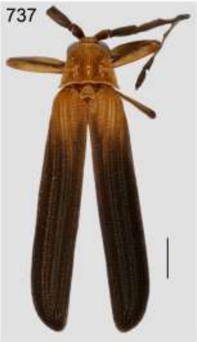

Microtrichalus sp., New Guinea

Metriorrhynchinae: Metriorrhynchini: Metriorrhynchina

Pattern: bicoloured elytra
pronotum and elytral humeri yellow

Remark: the examples of a variable extent the dark coloured apical part of the elytra, similarly, the contrast between the bright and dark parts of the elytra is variable

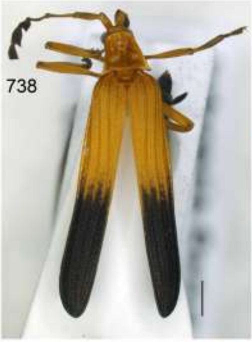

Eniclases bicolor Boc. et Boc., New Guinea

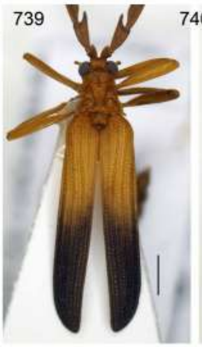

Eniclases similis Boc et. Boc., New Guinea

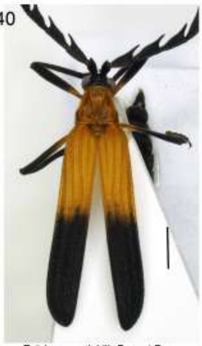

Eniclases variabilis Boc. et Boc., New Guinea

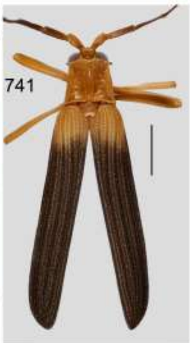

Microtrichalus sp., New Guinea

Metriorrhynchinae: Metriorrhynchini: Metriorrhynchina

Pattern: bicoloured elytra
pronotum and elytral humeri yellow

Remark: the characteristic dark coloured elytral suture evolved in unrelated species, here Eniclases and Microtrichalus, known also in some small-bodied Metriorrhynchus. The individuals with this pattern occur in a single aggreagtion with similar net-winged beetles shown above (Bocek et al., 2019).

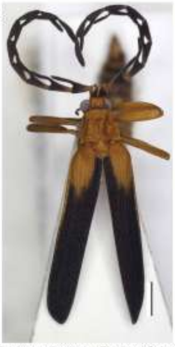

Eniclases divaricatus Pic, New Guinea

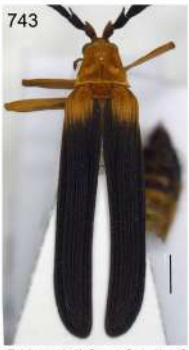

Eniclases similis Boc et. Boc., New Gu

Lycinae: Platerodini

Pattern: bicoloured elytra
pronotum and elytral humeri yellow

Remark: A pattern highly similar with numerous Metriorrhyn-china, which differ in the body size.

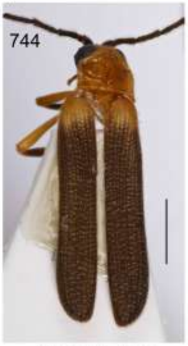

Plateros sp. New Guinea

Metriorrhynchinae: Metriorrhynchini: Metriorrhynchina

Pattern: bicoloured elytra
pronotum and elytral humeri brightly coloured

Body size: ~ 8 mm

Distribution: New Guinea

Non-lycid co-mimics: Cantharidae, Chrysomelidae

Remark: Pattern in the principle similar with the previous ones, but differs in the very high contrast between the bright and dark parts; of elytra. less common, but widespread pattern similar aposematic signal identified in unrelated Oriental *Cautires* (Cautirina).

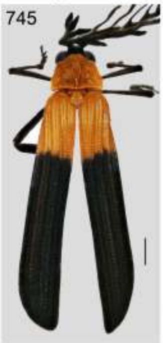

Flabellotrichalus sp., New Guinea

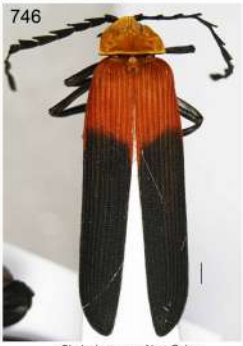

Cladophorus sp., New Guinea

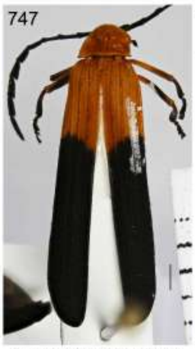

Metriorrhynchus sp., New Guinea

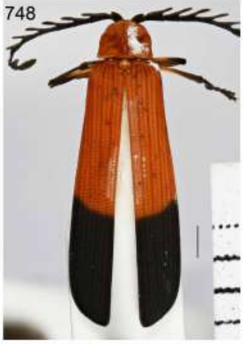

Ditua sp., New Guinea

All graphics and text produced by the authors as they are listed under the title of this article (CC-BY open acces license). Long horn beetle photographs taken by L. Dembicky.

New Guinea and adjacent islands

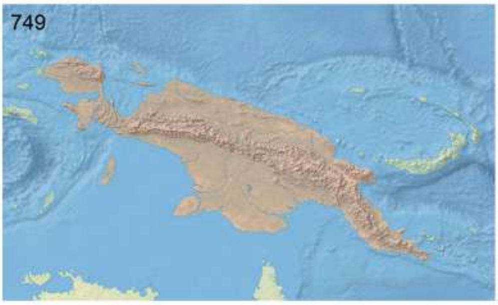

Pattern: bicoloured elytra with bright humeri, pronotum black

Body size: ~ 8 mm

Distribution: New Guinea

Non-lycid co-mimics:

Metriorrhynchinae: Metriorrhynchini: Metriorrhynchina

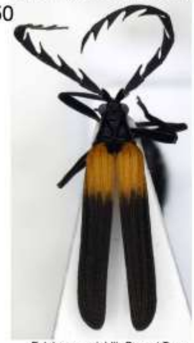

Eniclases variabilis Boc. et Boc., New Guinea

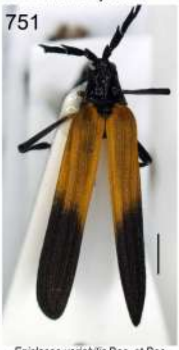

Eniclases variabilis Boc. et Boc., New Guinea

Pattern: bicoloured elytra with bright humeri, pronotum black

Remark: As previous, but small-bodied species from a different subfamily

Lycinae: Platerodini

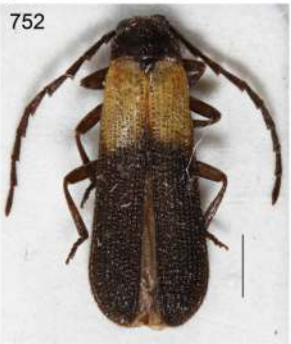

Plateros wauensis Bocakova, New Guinea

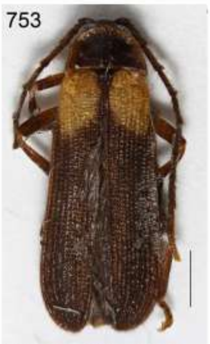

Plateros flavohumeralis Bocakova, New Guinea

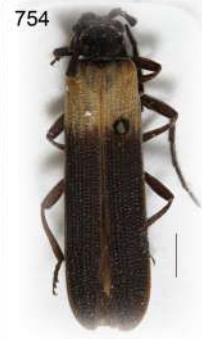

Plateros sedlaceki Bocakova, New Guinea

Pattern: bicoloured elytra with bright humeri, pronotum black

Body size: 6-14 mm

Distribution: New Guinea

Remark: An example of a very limited extent of the brightly coloured humeri

Metriorrhynchinae: Metriorrhynchini: Metriorrhynchina

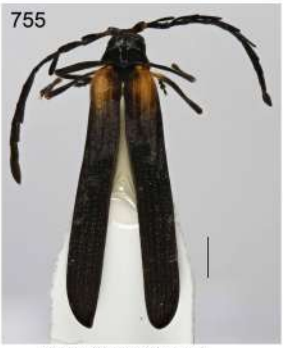

Metriorrhynchus sp., New Guinea

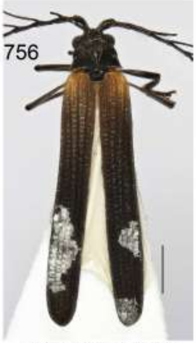

Metriorrhynchus sp., New Guinea

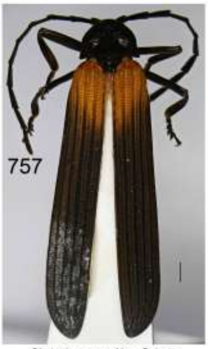

Cladophorus sp., New Guinea

Pattern: bicoloured elytra with bright humeri, pronotum black

Body size: 8-15 mm

Distribution: New Guinea

Remark: The pattern similar in colour arrangement, but characteristic in the high contrast and large body

Metriorrhynchinae: Metriorrhynchini: Metriorrhynchina

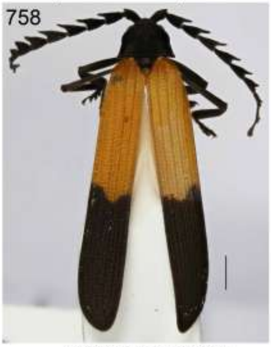

Metriorrhynchus sp., New Guinea

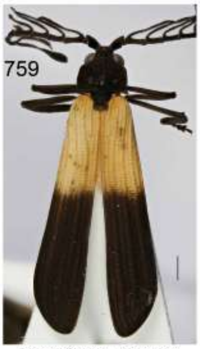

Flabellotrichalus sp., New Guinea

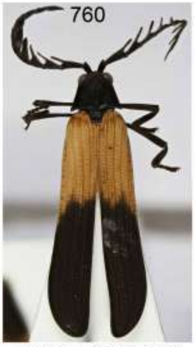

Metriorrhynchus sp., New Guinea

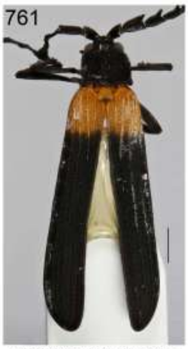

Flabellotrichalus sp., New Guinea

All graphics and text produced by the authors as they are listed under the title of this article (CC-BY open acces license). Long horn beetle photographs taken by L. Dembicky.

Pattern: bicoloured elytra with cinnabarin red humeri, pronotum black

Body size: 8-15 mm

Distribution: New Guinea

Remark: The pattern similar in colour arrangement, but characteristic in very bright humeri, the extremely high contrast and large body

Metriorrhynchinae: Metriorrhynchini: Metriorrhynchina

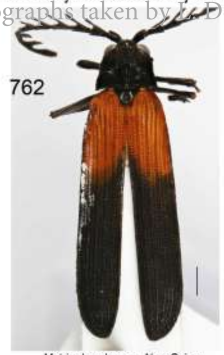

Metriorrhynchus sp., New Guinea

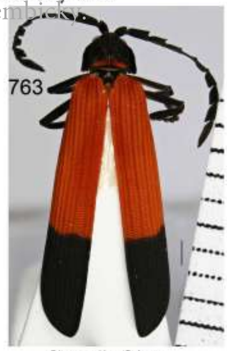

Ditua sp., New Guinea

Metriorrhynchinae: Metriorrhynchini: Metriorrhynchina

New Guinea and adjacent islands

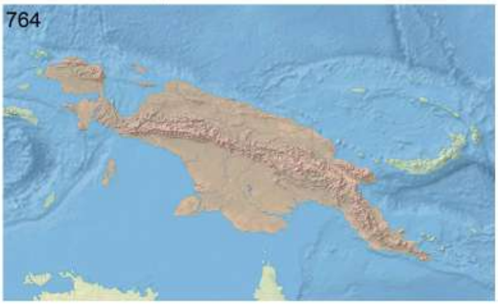

Pattern: bicoloured elytra, pronotum black, humeri testaceous; high contrast between light and dark area

Body size: 4-10 mm

Distribution: New Guinea

Non-lycid co-mimics: Cantharidae,

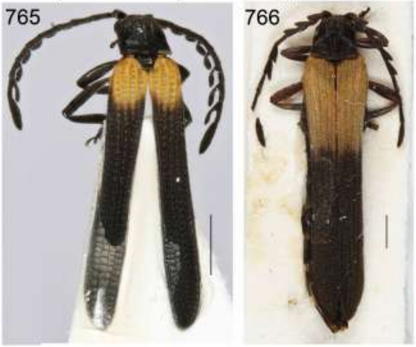

Metriorrhynchus sp., New Guinea

Metriorrhynchinae: Metriorrhynchini: Metriorrhynchina

New Guinea and adjacent islands

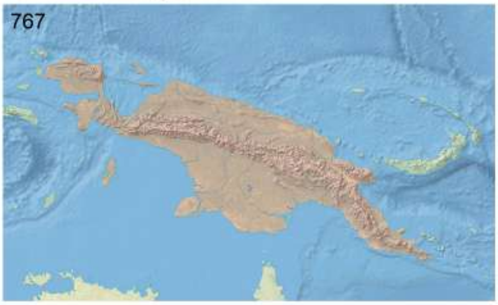

Pattern: bicoloured elytra, pronotum black, humeri testaceous; low contrast between light and dark area

Body size: 4-10 mm

Distribution: New Guinea

Non-lycid co-mimics: Cantharidae,

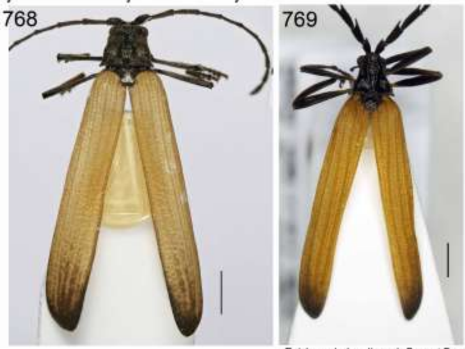

Eniclases bokondinensis Boc. et Boc., New Guinea

Metriorrhynchinae: Metriorrhynchini: Metriorrhynchina

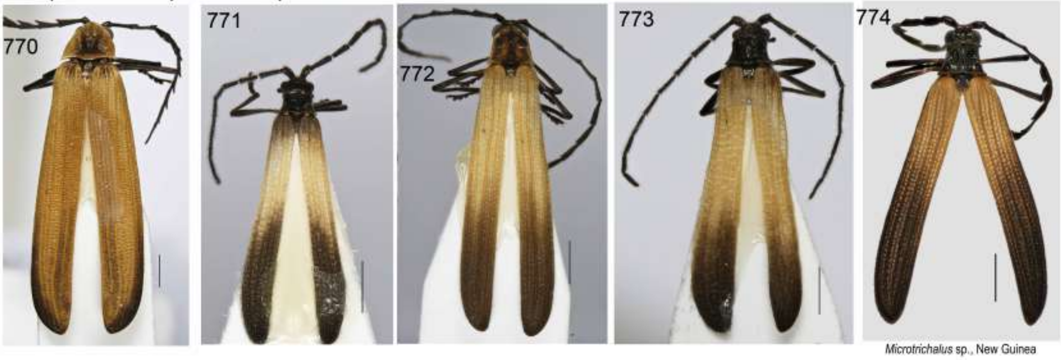

Microtrichalus sp., New Guinea

New Guinean high mountains (cca over 2000 m a. s. l.)

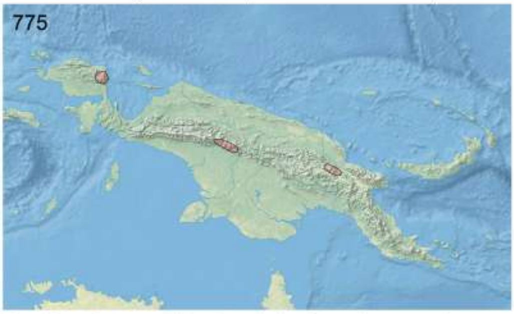

Note: Due to inaccessibility, data missing from other mountain ranges

Pattern: bicoloured elytra; pronotum black, humeral part of the pronotum white, apex black

Body size: ~ 7 mm

Distribution: high mountain of New Guinea

Further Lycidae co-mimics: Metriorrhynchus spp.

Non-lycid co-mimics: Cantharidae

Metriorrhynchinae: Metriorrhynchini: Metriorrhynchina

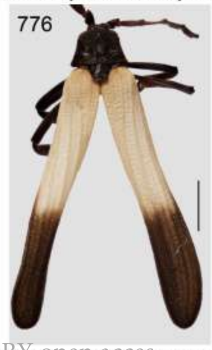

Flabellotrichalus horaki Bocek, New Guinea

All graphics and text produced by the authors as they are listed under the title of this article (CC-BY open access license). Long horn beetle photographs taken by L. Dembicky.

Metriorrhynchinae: Metriorrhynchini: Metriorrhynchina

Pattern: bicoloured elytra; pronotum black, humeral part of the pronotum black, apex white

Body size: ~ 7 mm

Distribution: high mountain of New Guinea

Further Lycidae co-mimics: *Metriorrhynchus* spp.

Non-lycid co-mimics: Cantharidae

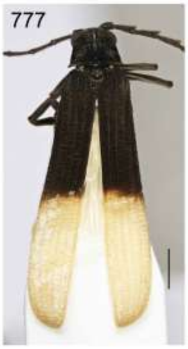

Metriorrhynchinae: Metriorrhynchini: Metriorrhynchina

Pattern: bicoloured elytra; pronotum yellow, humeral part of the pronotum yellow, apex white, elytral margins infuscate

Body size: ~ 8 mm

Distribution: high mountain of New Guinea

Further Lycidae co-mimics: none

Non-lycid co-mimics: none

Remark: known a single species

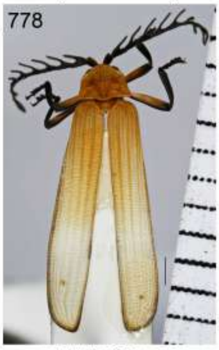

indet., New Guinea

New Guinea

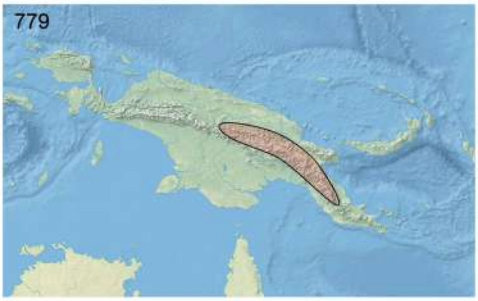

Pattern: bicoloured elytra,
apex testaceous
pronotum black

Body size: 15-18 mm

Distribution: mountains of eastern New Guinea

Further Lycidae co-mimics none

Non-lycid co-mimics: none

Metriorrhynchinae: Metriorrhynchini: Metriorrhynchina

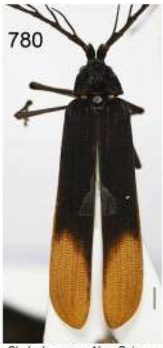

Cladophorus sp., New Guinea

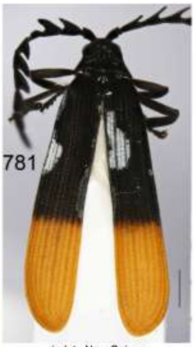

indet., New Guinea

Metriorrhynchinae: Metriorrhynchini: Metriorrhynchina

Pattern: bicoloured elytra,
apex dark reddish brown;
pronotum yellow

Body size: 15-18 mm

Distribution: mountains of eastern New Guinea

Non-lycid co-mimics: none

Remark: a rare pattern

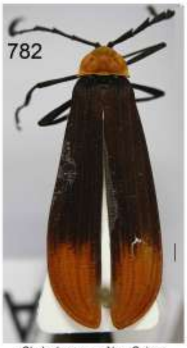

Cladophorus sp., New Guinea

pronotum silver

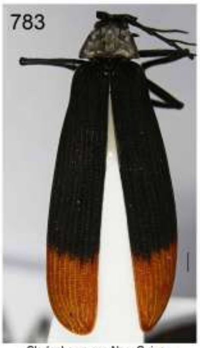

Cladophorus sp., New Guinea

pronotum black

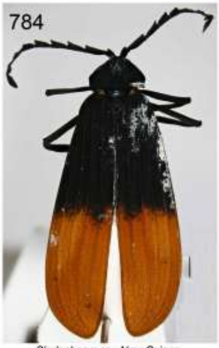

Cladophorus sp., New Guinea

Metriorrhynchinae: Metriorrhynchini: Metriorrhynchina

Pattern: bicoloured elytra,
humeri black, apex red.
pronotum as elytra

Body size: 15-18 mm

Distribution: mountains of eastern New Guinea

Further Lycidae co-mimics:none

Non-lycid co-mimics: none

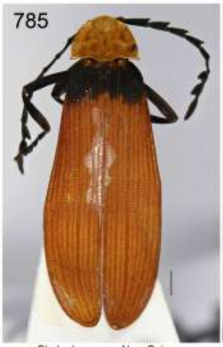

Cladophorus sp., New Guinea

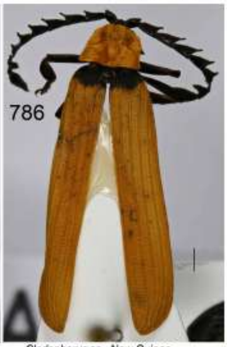

Cladophorus sp., New Guinea

Metriorrhynchinae: Metriorrhynchini: Metriorrhynchina

Pattern: uniform red elytra,
pronotum yellow

Body size: ~ 14 mm

Distribution: mountains of eastern New Guinea

Further Lycidae co-mimics none

Non-lycid co-mimics: none

Remark: a similar colour pattern is known in a single species of *Cautires*

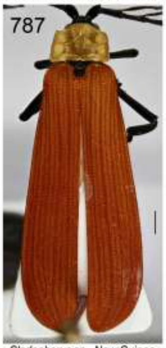

Cladophorus sp., New Guinea

Metriorrhynchinae: Metriorrhynchini: Metriorrhynchina

Pattern: bicoloured elytra,
apex infuscate or black
pronotum coloured as elytra

Body size: 15-18 mm

Distribution: New Guinea

Further Lycidae co-mimics: none

Non-lycid co-mimics:
Cantharidae (*Chauliognathus*)

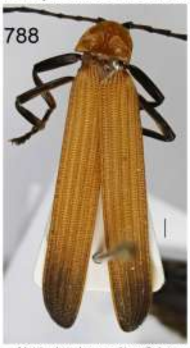

Metriorrhynchus sp., New Guinea

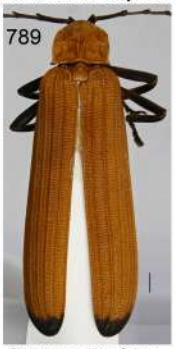

Cladophorus sp., New Guinea

Metriorrhynchinae: Metriorrhynchini: Metriorrhynchina

Pattern: uniform dark red elytra,
apex infuscate, with light
coloured costae
pronotum red

Body size: ~17 mm

Distribution: mountains of eastern New Guinea

Further Lycidae co-mimics: none

Non-lycid co-mimics: none

Remark: a single species with a unique pattern

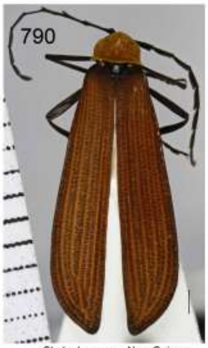

Cladophorus sp., New Guinea

Metriorrhynchinae: Metriorrhynchini: Metriorrhynchina

Pattern: pronotum and elytra
black

Body size: 17 mm

Distribution: mountains of eastern New Guinea

Non-lycid co-mimics: none

Remark: Although uniform black as other net-winged beetle in the area. it differs in the body shape and size

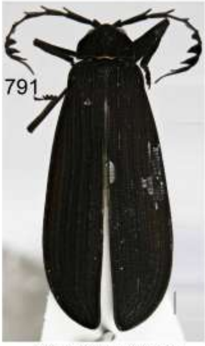

Cladophorus sp., New Guinea

All graphics and text produced by the authors as they are listed under the title of this article (CC-BY open acces license). Long horn beetle photographs taken by L. Dembicky.

New Guinea and adjacent island

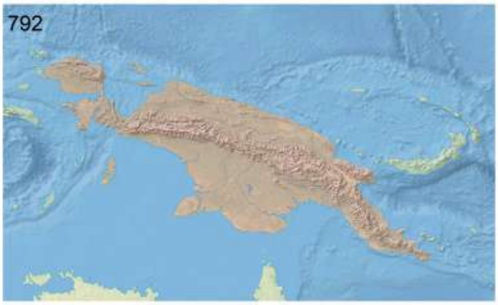

Pattern: uniform broen elytra;
pronotum similarly coloured
or with a black patch

Body size: 4-7 mm

Distribution: New Guinea

Further Lycidae co-mimics: none
Non-lycid co-mimics: Cantharidae
Remark: small bodied forms

Lycinae: Platerodini

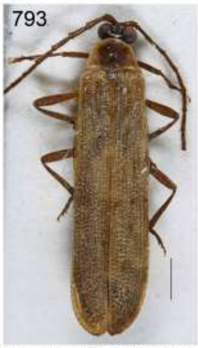

Plateros montanus Bocakova, New Guinea

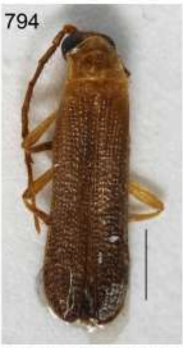

Plateros usitatus Bocakova, New Guinea

Pattern: bicoloured elytra with a
substatntial part testaceous,
apex black; pronotum black

Body size: ~ 7 mm

Distribution: New Guinea

Further Lycidae co-mimics: none
Non-lycid co-mimics: Cantharidae

Lycinae: Platerodini

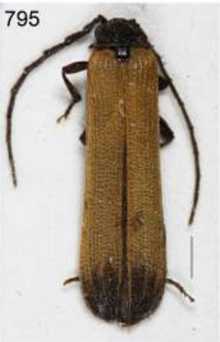

Plateros sedlaceki Bocakova, New Guinea

Pattern: bicoloured elytra with a
humeral part testaceous,
apex black, transition gradual;
pronotum black or brown

Body size: 5-7 mm

Distribution: New Guinea

Further Lycidae co-mimics: none

Non-lycid co-mimics: Cantharidae

Lycinae: Platerodini

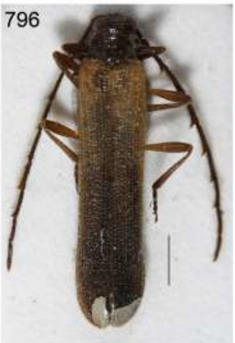

Plateros prominens Bocakova, New Guinea

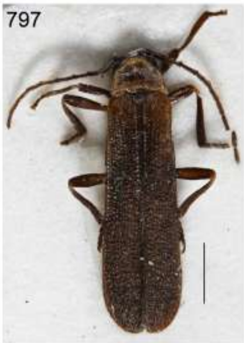

Plateros guineensis Pic, New Guinea

Pattern: bicoloured elytra with humeri and anterior lateral
margins and suture orange; pronotum orange
with a black patch

Body size: ~ 8 mm

Distribution: New Guinea

Further Lycidae co-mimics: none
Non-lycid co-mimics: none
Remark: a characteristic pattern occurring with
a low frequency in lowland habitats

Metri.:Metr.: Metriorrhynchina

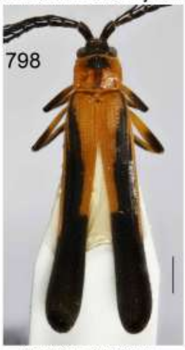

Carathrix sp., New Guinea

Pattern: bicoloured elytra with humeri and
apex blact, the middle part orange;
pronotum black black and orange

Body size: ~ 8 mm

Distribution: New Guinea

Further Lycidae co-mimics: none
Non-lycid co-mimics: none
Remark: a characteristic pattern
occurring in a low frequency in
lowland habitats

Metri.:Metr.: Metriorrhynchina

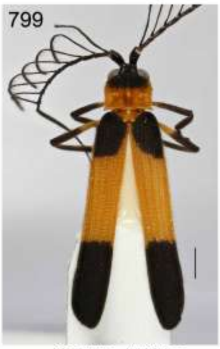

Carathrix sp., New Guinea

Pattern: bicoloured elytra with humeri and anterior lateral
margins black; pronotum black

Body size: ~ 10 mm

Distribution: Eastern New Guinea

Further Lycidae co-mimics: none
Non-lycid co-mimics: none
Remark: a characteristic pattern represented by a single
common species

Metri.:Metr.: Metriorrhynchina

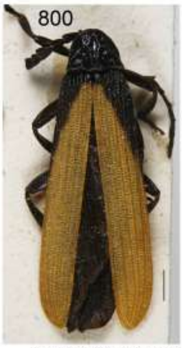

M. marginatus Kln., New Giunea

Pattern: uniform metallic blue, gree or bronze upper part
of the body
Body size: 6-9 mm
Distribution: New Guinea
Further Lycidae co-mimics: none
Non-lycid co-mimics: Cantharidae
Remark: metallically coloured *Diatrichalus* and several
other Lycidae occur in a low frequency from lowlands to
high mountains

All graphics and text produced by the authors as they are listed under the title of the article (CC-BY open acces license). Long horn beetle photographs taken by E. Dembicky.

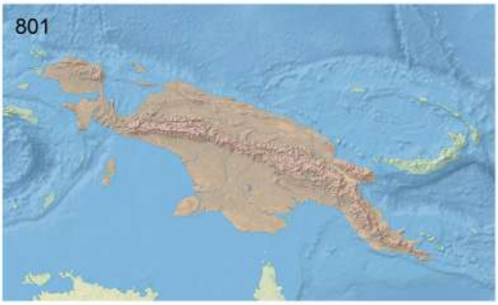

Metri.:Metr.: Metriorrhynchina

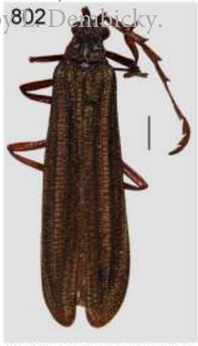

Diatrichalus aeneus Boc., New Guinea

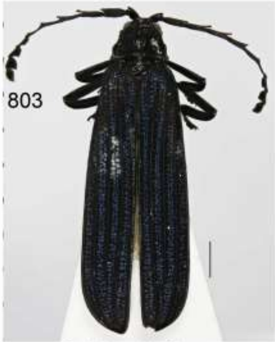

Diatrichalus sp., New Guinea

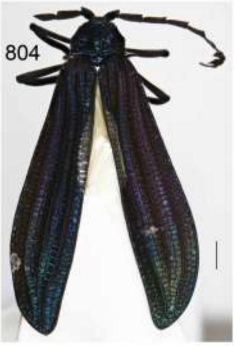

Diatrichalus sp., New Guinea

New Guinea

mountains of
the eastern
part of the island

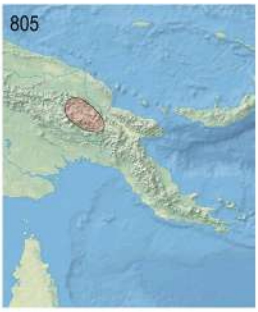

Metriorrhynchinae: Metriorrhynchini: Metriorrhynchinae

Pattern: uniform green elytra (the variable shade),
pronotum reddish

Body size: 11-13 mm

Distribution: mountains of eastern New Guinea

Non-lycid co-mimics: none

Remark: a rare pattern with modification
shown below

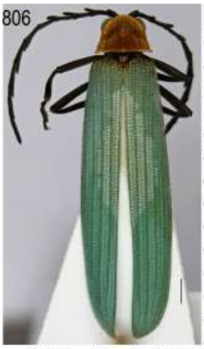

Metriorrhynchus sp., New Guinea

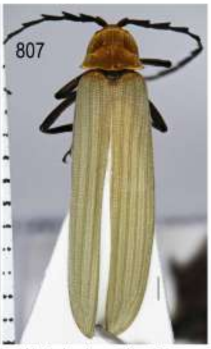

Metriorrhynchus sp., New Guinea

Metriorrhynchinae: Metriorrhynchini: Metriorrhynchinae

Pattern: uniform blue elytra (the varibale shade),
pronotum reddish

Body size: 11-13 mm

Distribution: mountains of eastern New Guinea

Non-lycid co-mimics: none

Remark: a rare pattern with modification
shown above

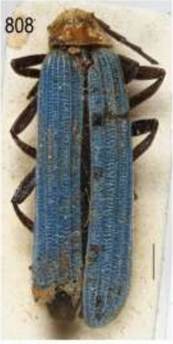

M. tricolor Kleine, New Guinea

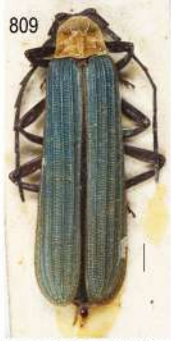

M. smaragdinus Kleine, New Guinea

Metriorrhynchinae: Metriorrhynchini: Metriorrhynchinae

Pattern: uniform green elytra
(the variable shade),
pronotum black

Body size: ~10 mm

Distribution: mountains of eastern
New Guinea

Non-lycid co-mimics: none

Remark: a rare pattern with
modifications shown above

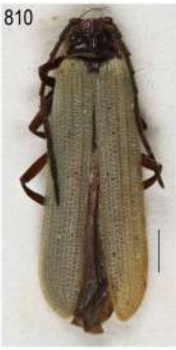

Metriorrhynchus sp., New Guinea

Metriorrhynchinae: Metriorrhynchini: Metriorrhynchinae

Pattern: uniform green elytra
pronotum light green with black
patch and with light margins

Body size: ~ 17 mm

Distribution: mountains of eastern New Guinea

Further Lycidae co-mimics: none
Non-lycid co-mimics: none

Remark: a single species
with the green colouration

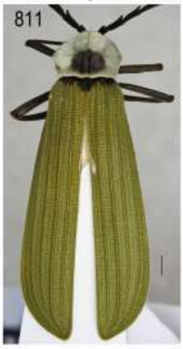

Porrostoma sp., New Guinea

Metriorrhynchinae: Metriorrhynchini: Metriorrhynchinae

Pattern: uniform reddish elytra
pronotum black and/or with light
silver pubescence

Body size: ~ 8 mm

Distribution: mountains of eastern New Guinea

Further Lycidae co-mimics: *Diatrichalus* sp.
Non-lycid co-mimics: none

Remark: uncommon pattern; a part
of aggregations in mountains

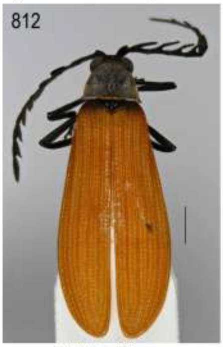

indet., New Guinea

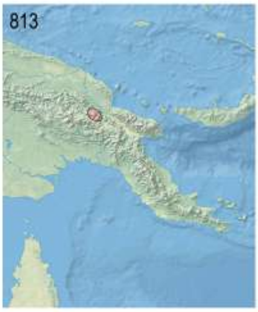

Metriorrhynchinae: Metriorrhynchini: Metriorrhynchinae

Pattern: black pronotum reddish elytra
with light black transverse band

Body size: 8-12 mm

Distribution: mountains of eastern New Guinea

Further Lycidae co-mimics: none
Non-lycid co-mimics: none

Remark: a pattern represented by a single
very common species in the mountain
of Mt. Wihelm (east New Gionea)

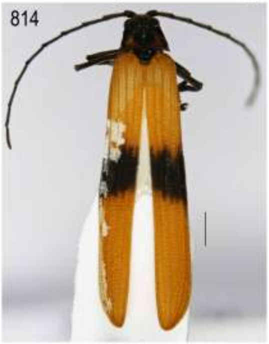

Metriorrhynchus sp., New Guinea

All graphics and text produced by the authors as they are listed under the title of this article (CC-BY open acces license). Long horn beetle photographs taken by L. Dembicky.

New Guinea and adjacent island

815

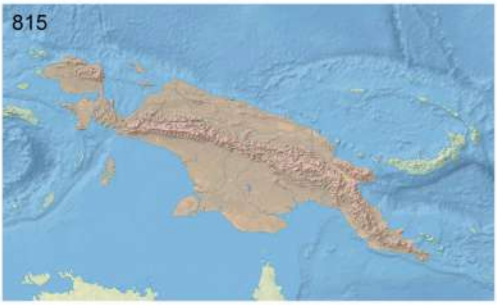

Elateridae

816

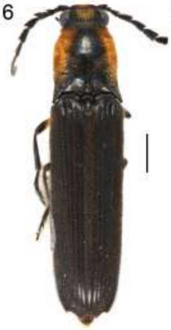

indet.,W. Papua, Fak Fak

Cleridae

817

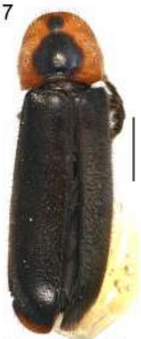

indet.,W. Papua, Fak Fak

Cantharidae

818

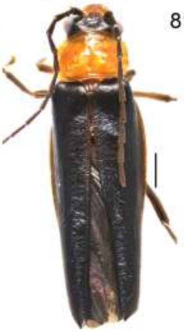

indet., Papua, Kobakma

Chrysomelidae

819

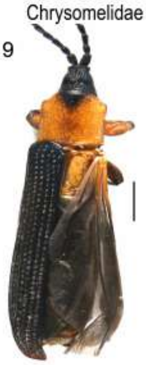

indet., W. Papua: Bokondini

Cantharidae

820

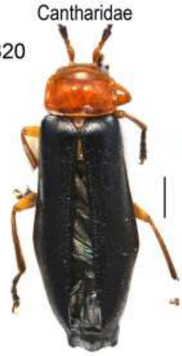

indet., W. Papua, Fak Fak

Oedemeridae

821

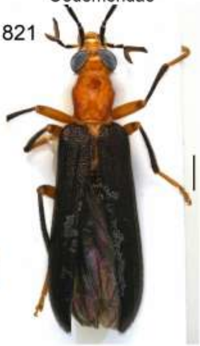

indet., W. Papua, Fak Fak

Elateridae

822

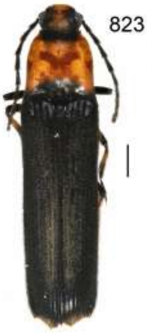

indet., W. Papua, Fak Fak

Elateridae

823

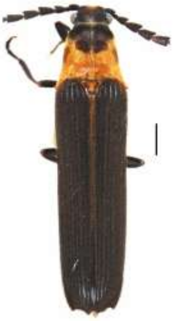

indet., W. Papua, Kobakma

Eucnemidae

824

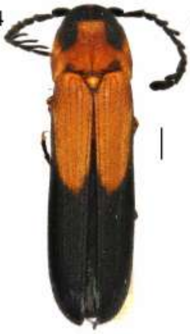

indet., W. Papua, Fak Fak

Elateridae

825

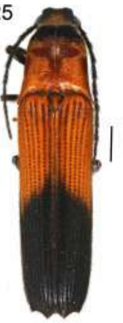

indet., W. Papua, Fak Fak

Cleridae

826

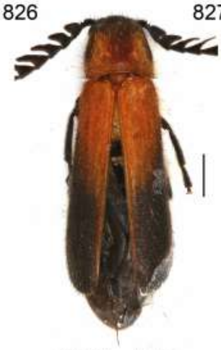

Cantharidae

827

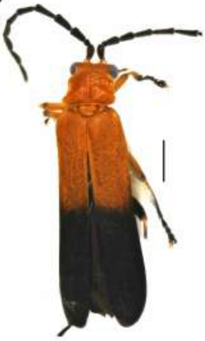

indet., W. Papua, Fak Fak

Cantharidae

828

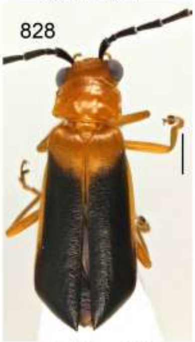

indet., W. Papua, Fak Fak

Oedemeridae

829

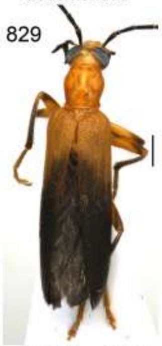

indet., W. Papua, Fak Fak

All graphics and text produced by the authors as they are listed under the title of this article (CC-BY open acces license). Long horn beetle photographs taken by L. Dembicky.

Oedemeridae

830

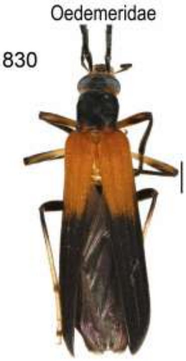

indet., W. Papua, Fak Fak

Chrysomelidae

831

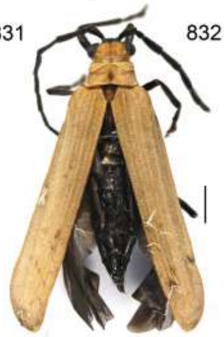

indet., Papua, Wamena

Oedemeridae

832

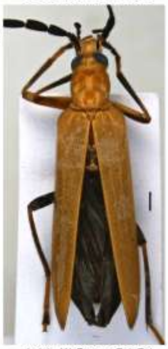

indet., W. Papua, Fak Fak

Oedemeridae

833

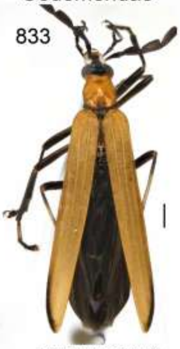

indet., Papua, Bokondini

Erotylidae: Languriinae

834

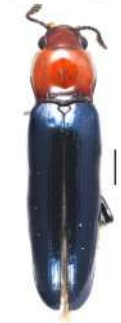

indet., W. Papua, Fak Fak

835

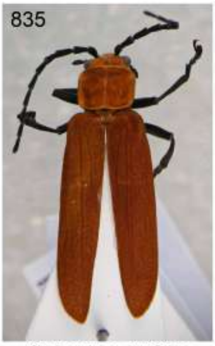

Chauliognathus sp., New Guinea

836

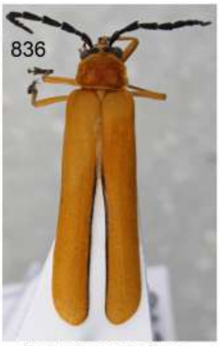

Chauliognathus sp., New Guinea

Chrysomelidae

837

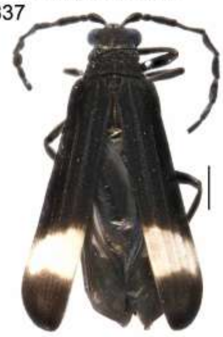

indet. W. Papua: Bokondini

Chrysomelidae

838

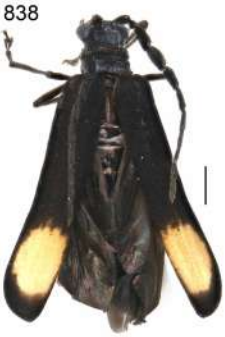

indet. W. Papua: Bokondini

Chrysomelidae

839

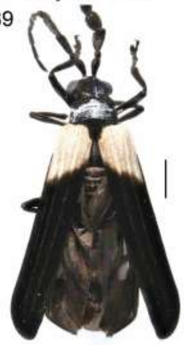

indet. W, Papua: Bokondini

Chrysomelidae

840

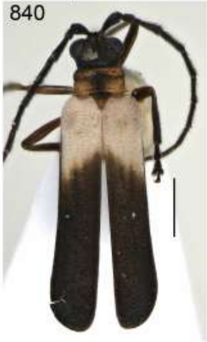

indet. W. Papua: Wamena

Chrysomelidae

841

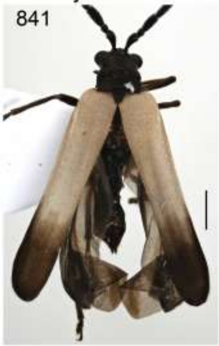

indet. W. Papua: Wamena

Chrysomelidae

842

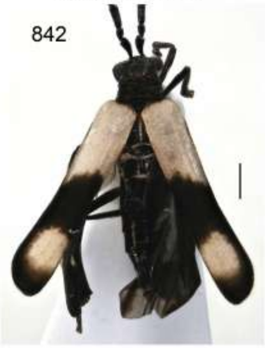

indet. W, Papua: Bokondini

Cantharidae

843

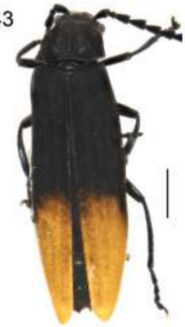

indet. W, Papua: Bokondini

Cantharidae

844

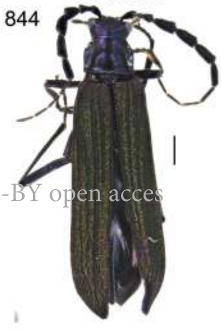

indet. W, Papua: Bokondini

All graphics and text produced by the authors as they are listed under the title of this article (CC-BY open acces license). Long horn beetle photographs taken by L. Dembicky.

Australian Region

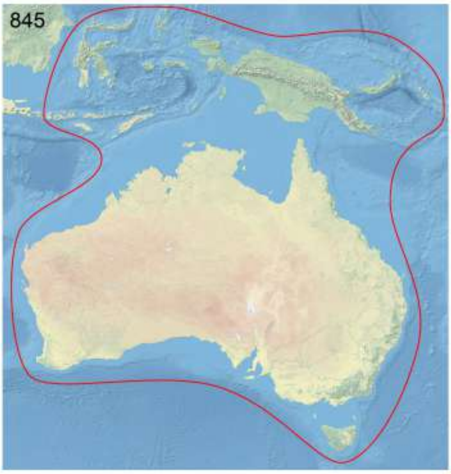

Continental Australia and Tasmania

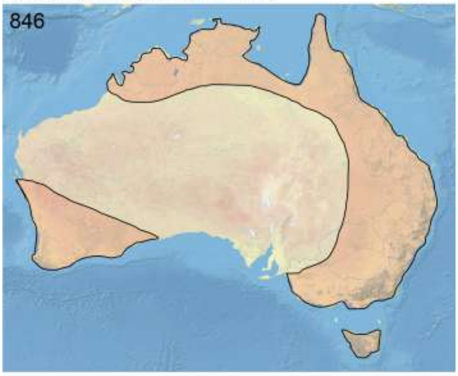

Phylogenetic structure of the fauna

Subfamily	Tribe /Subtribe	# spp. (woldwide)	# spp. (region)
Dexorinae	Dexorini	15 spp.	
	Mimolibnetini	5 spp.	
Erotinae	Erotini	54 spp.	
	Dictyopterini	73 spp.	
	Taphini	31 spp.	2 spp.
	Slipinskiini	46 spp.	
Calochrominae	Calochromini	289 spp.	17 spp.
Ateliinae	Ateliini	45 spp.	
	Lyponiini	45 spp.	
	Macrolycini	69 spp.	
Lyropaeinae	Lyropaeini	43 spp.	
	Alyculini	5 spp.	
	Antennolycini	3 spp.	
	Platerodrilini	49 spp.	
Lycinae	Conderini	42 spp.	
	Eurrhacini	102 spp.	
	Thonalmini	11 spp.	
	Leptolycini	12 spp.	
	Platerodini	861 spp.	3 spp.
	Calopterini	367 spp.	
	Lycini	413 spp.	
Metriorrhynchinae	Dihammatini	44 spp.	
	Lycoprogenthini	7 spp.	
	Libnetini	112 spp.	
	Dilophotini	81 spp.	
	Metriorrhynchini	1410 spp.	
	Metanoeina	40 spp.	
	Cautirina	750 spp.	
	Metriorrhynchina	620 spp.	193 spp.
Total		4234 spp.	215 spp.

The presence of net-winged beetle aposematic patterns in the region

Group	Colour type	Characteristics	+/-	Examples
Uniform coloration	black	pronotum an elytra uniformly black to dark btown		
	yellow	pronotum and elytra yellow to light brown		
	orange and red	pronotum and elytra brightly orange or cinnamon red	✓	879
	metallic (blue, green)	pronotum and elytra metalic, all shades of colours		
Bi-colored pronotum/ elytra	black/yellow	pronotum back (at most with bright margins), elytra yellow		
	black/red	pronotum black (at most with bright margins), elytra red	✓	870-874
	bright/black	pronotum brightly colored, elytra uniformly black	✓	876, 877
	red/merallic	pronotum brightly red, elytra metallic blue		
Bi-colored elytra	yellow/black	elytra bi-colored. humeral part yellow, apical part dark coloured	✓	846-852, 862-866
	red(orange)/black	elytra bi-colored. humeral part orange/red, apical part dark		
	black/bright	elytra bi-colored. humeral part black, apex yellow/orange/red	✓	881
Fasciate elytra	yellow/black	humeri and apex of elytra black, middle of elytron yellow		
	bright/black	humeri and apex of elytra brigt, middle of elytron black		
	yellow/metallic	most elytra black with blue metallic shine, middle yellow		
Striate elytra	bright/black	elytra brown/brightly coloured, suture or middle of elytron black	✓	855, 856, 858, 860, 867, 868
Punctate el.	bright/black	elytra brown/brightly coloured, black patch in each elytron	✓	854
Tri-colored el.	all combinations of colors	elytron with three differently coloured parts		
Reticulate	bright/black	backgroud colour of elytra dark, costae large, brightly coloured		
Non-categorized aposematic patterns		see listed examples for further information		

Figures S845–S914. The overview of the lycid fauna, coloration, and co-mimics: Australian region, Australia, and Tasmania.

All graphics and text produced by the authors as they are listed under the title of this article (CC-BY open acces license). Long horn beetle photographs taken by L. Dembicky.

Australia: Queensland, Northern Teritory, Western Australia

All Metriorrhynchinae: Metriorrhynchini: metriorrhyncha if not stated otherwise

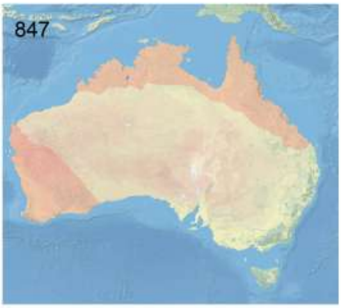

Pattern: bicoloured elytra - yellow/black
pronotum yellow or with a median black patch
Body size: 7-10 mm

Distribution: northern continental Australia

Further Lycidae co-mimics: small-bodied *Porrostoma*

Non-lycid co-mimics: Cantharidae, Belidae
Remark: the pattern widespread in the Lesser Sundas, Java, and southern New Guinea

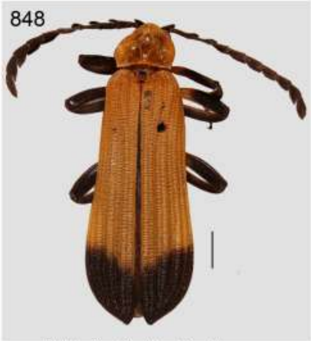

Trichalus flavopictus Waterh., Australia

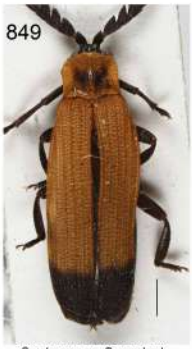

Synchonnus sp., Queensland

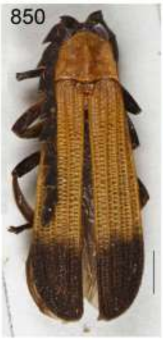

Trichalus sp., Queensland

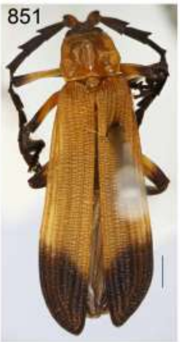

Lobatang sp., N.T., Australia

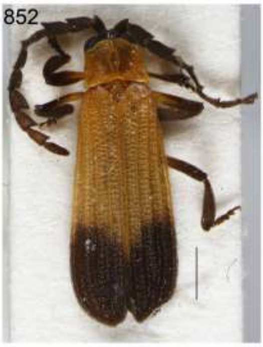

Lobatang sp., N.T., Australia

Australia: Southern Queensland, Northern New South Wales

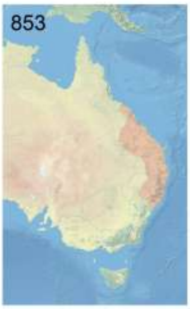

Pattern: stripped elytra - yellow/black
pronotum black or with a median black patch
Body size: 7-11 mm

Distribution: Eastern Australia

Non-lycid co-mimics: none

Remark: the pattern endemic to eastern Australia;small-bodied version of the patternrepresented by *Metriorrhynchus* (Fig..xxx)

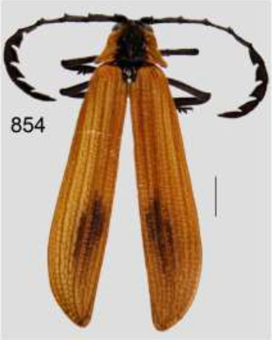

Synchonnus crypticus Kusy, Queensland

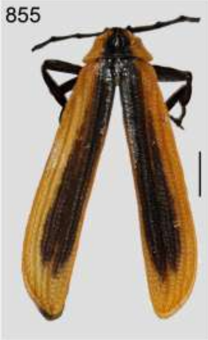

Synchonnus crypticus Kusy, Queensland

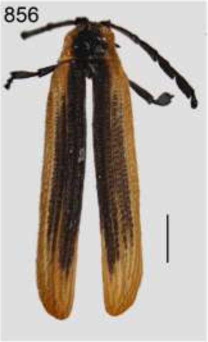

Synchonnus dubenovae Kusy, Queensland

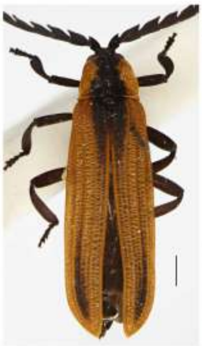

Trichalus sp., New South Wales

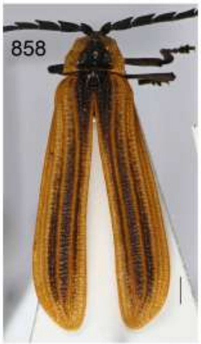

Trichalus sp., New South Wales

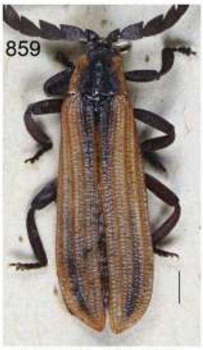

Synchonnus sp., N.S.W., Australia

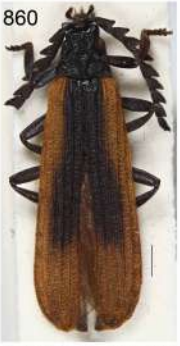

Porrostoma sp., Australia, N.S.W.

All graphics and text produced by the authors as they are listed under the title of this article (CC-BY open acces license). Long horn beetle photographs taken by L. Dembicky.

Australia: Queensland, Southern New South Wales

Calochrominae: Calochromini

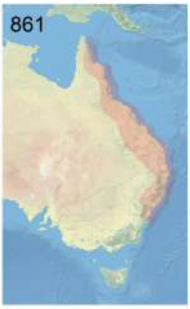

Pattern: bicoloured elytra - yellow/black; pronotum bllack
Body size: 6-8 mm, *Calochomus* 4–6 mm

Distribution: Eastern Australia

Non-lycid co-mimics:

Remark: *Calochromus* flower visiting; the pattern known from New Guinea; small-bodied version of the pattern represented by *Porrosotma* sp. (Fig..xxx)

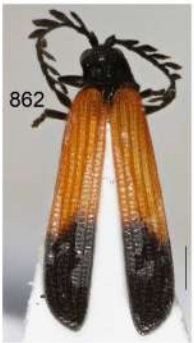

indet., Queensland

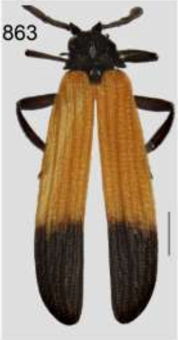

Synchonnus monteithi Kusy, Queensland

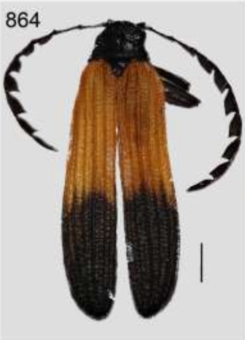

Synchonnus clientulus Waterh., Queensland

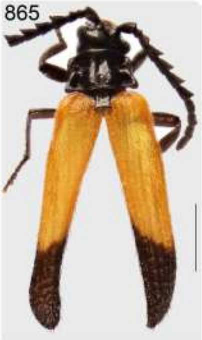

Escalonia amabilis (xxx) Australia

Pattern: bicoloured elytra - yellow/ black; pronotum black
Body size: 12-15 mm

Distribution: Eastern Australia

Non-lycid co-mimics:

Remark: the pattern endemic common in New Guinea

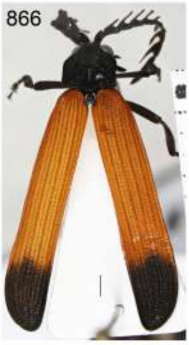

Porrostoma sp., Queensland

Pattern: stripped elytra - yellow/ black; pronotum with a median black patch
Body size: 12-17 mm

Distribution: Eastern Australia

Non-lycid co-mimics:

Remark: flower visiting spp, form aggregations; the pattern endemic to eastern Australia; large-bodied version of the pattern represented by *Enylus* (Fig..xxx)

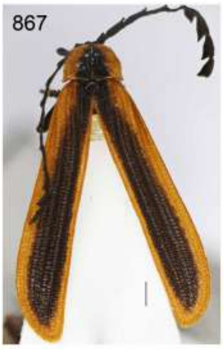

Trichalus sp., New South Wales

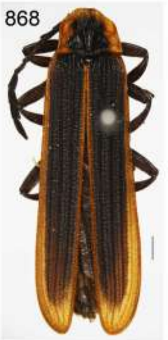

Metriorrhynchus eremita, Australia

Australia: Southern Queensland, New South Wales, Victoria

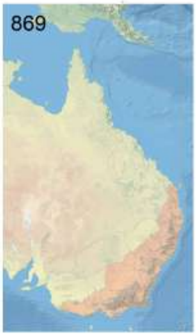

Pattern: uniform red elytra, black pronotum

Body size: 10-17 mm

Distribution: south-eastern Australia

Non-lycid co-mimics: Cantharidae, Belidae, Cerambycidae

Remark: flower visiting spp, form aggregations; the pattern dominant in SE Australia;

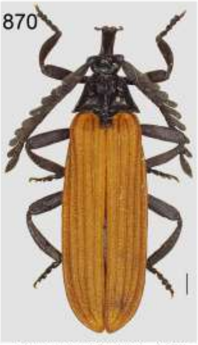

Porrostoma rhipidium Macl., N.S.W.

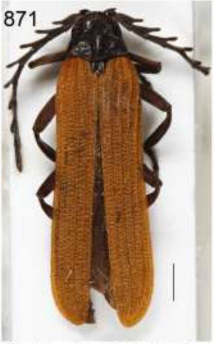

Porrostoma rufipennis F., Australia, Vic.

Pattern: uniform red elytra, black pronotum with red margins

Body size: 10-17 mm

Distribution: south-eastern Australia

Non-lycid co-mimics: Cantharidae, Belidae, Cerambycidae

Remark: flower visiting spp, form aggregations; the pattern dominant in SE Australia;

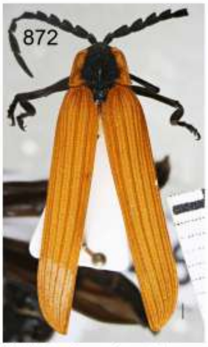

Metriorrhynchus sp., Queensland

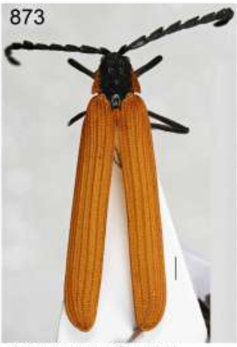

Metriorrhynchus sp., Queensland

Pattern: uniform red elytra, black pronotum

Body size: ~8 mm

Distribution: eastern Australia

Non-lycid co-mimics: Cantharidae

Remark: small-bodied version of the patternrepresented by *Porrostoma* (Fig..xxx)

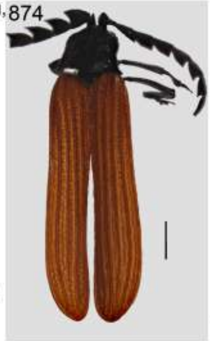

Synchonnus maseki Kusy, Queensland

Australia: Queensland

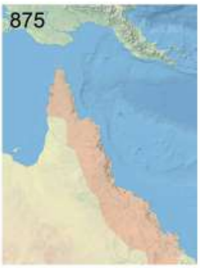

Pattern: dicoloured; red pronotum, black elytra

Body size: ~7 mm

Distribution: north-eastern Australia

Non-lycid co-mimics:

Remark: the common pattern in New Guinea

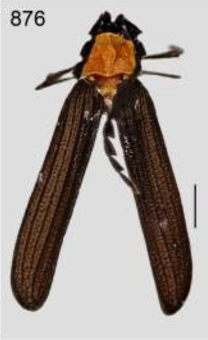

Synchonnus chilvertonensis Kusy, Queensland

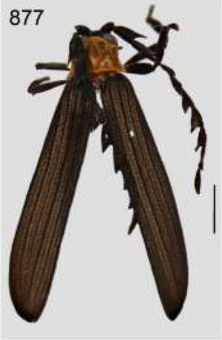

Synchonnus flavonotatum Kusy, Queensland

Pattern: bicoloured; red pronotum, black elytra with bright humeri

Body size: ~7 mm

Distribution: north-eastern Australia

Non-lycid co-mimics:

Remark: the common pattern in New Guinea

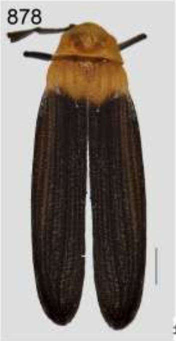

Synchonnus slipinskii Kusy, Queensland

Pattern: uniform orange-red

Body size: 10-12 mm

Distribution: north-eastern Australia

Non-lycid co-mimics: Cantharidae

Remark: the common pattern in New Guinea

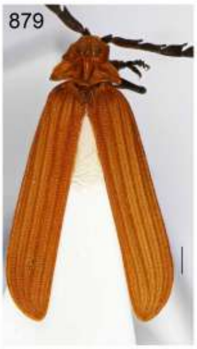

Porrostoma sp., N. Queensland

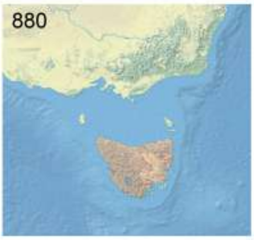

Pattern: bicoloured elytra with yellow/red apex

Body size: 8-9 mm

Distribution: Tasmania

Non-lycid co-mimics: Cantharidae

Remark: the pattern endemic to Tasmania

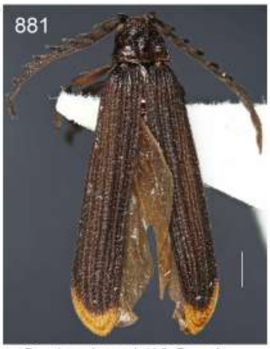

Porrostomae haemorrhoidalis, Tasmania

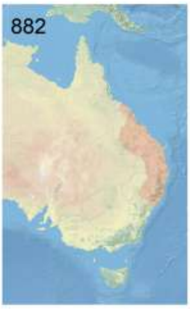

Oedemeridae

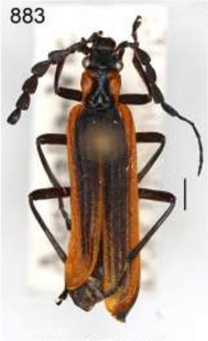

indet sp. New South Wales

Oedemeridae

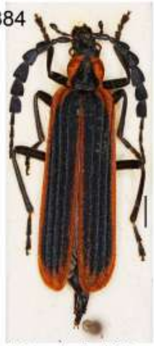

indet sp. New South Wales

Oedemeridae

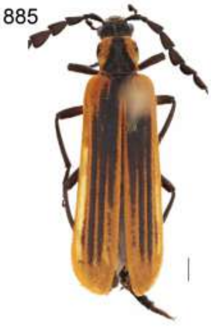

Pseudolycus sp., New South Wales

Pyrochroidae

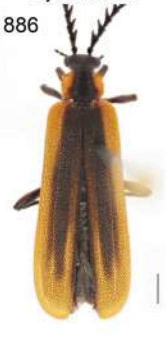

Morpholycus sp., Queensland

Pyrochroidae

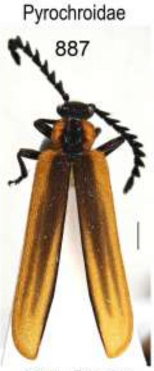

indet sp., Queensland

Pyrochroidae

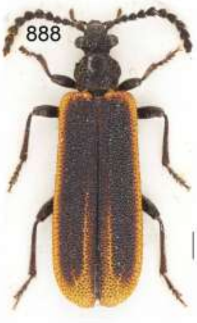

Morpholycus sp., New South Wales

Cerambycidae

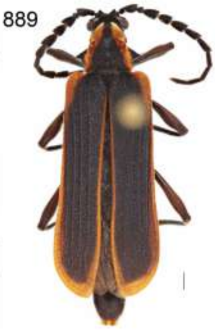

indet sp. New South Wales

Belidae

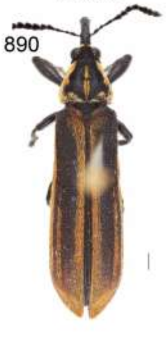

Rhinotia sp., New South Wales

Queensland, New South Wales

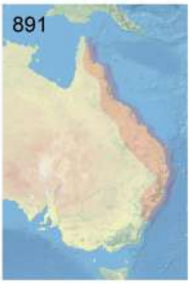

Pyrochroidae

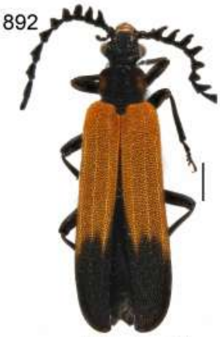

indet sp., Queensland

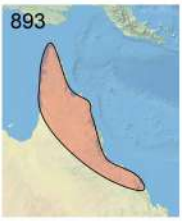

Bright orange/black pattern widespread in New Guinea, co-mimics in the Lycidae Cape York fauna

Cantharidae

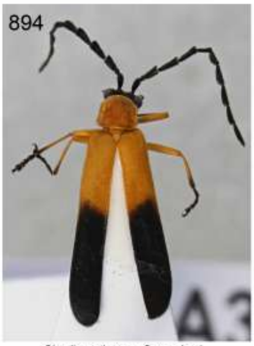

Chauliognathus sp., Queensland

Pyrochroidae

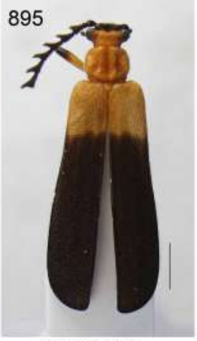

indet., Queensland

All graphics and text produced by the authors as they are listed under the title of this article (CC-BY open acces license). Long horn beetle photographs taken by L. Dembicky.

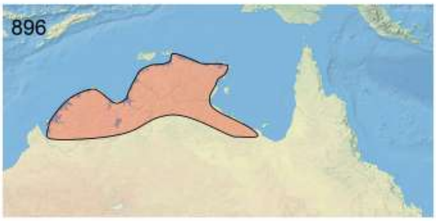

Cantharidae

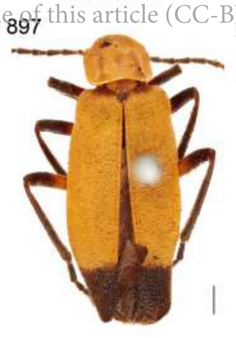

Chauliognathus sp., Northern Territory

Belidae

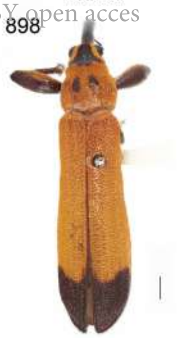

Rhinotia sp., Northern Territory

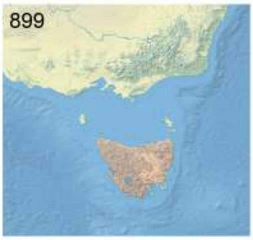

Oedemeridae

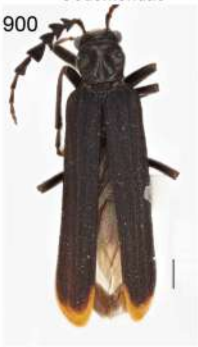

Pseudolycus sp., Tasmania

Oedemeridae

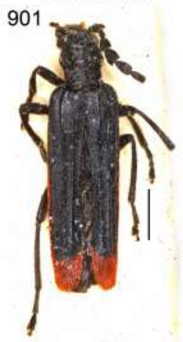

indet sp., New South Wales

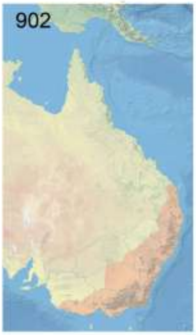

Cleridae

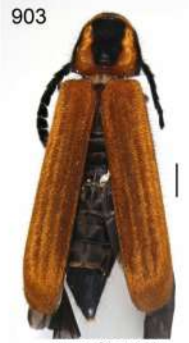

indet sp. Queensland

Buprestidae

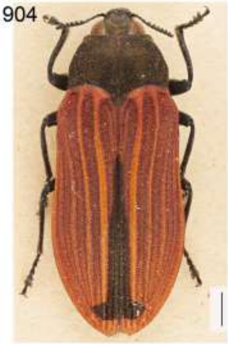

indet sp. Victoria

Oedemeridae

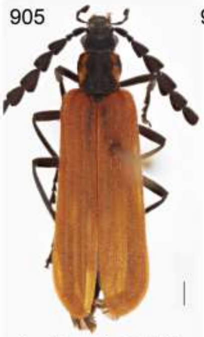

Pseudolycus sp. New South Wales

Oedemeridae

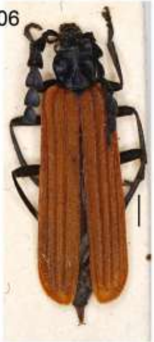

indet sp. New South Wales

Pyrochroidae

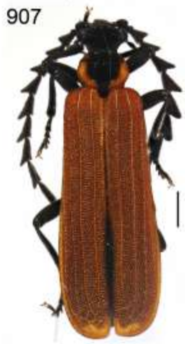

indet sp. Queensland

Pyrochroidae

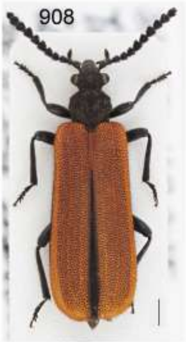

Morpholycus sp.
Australian Capital Territory

Cerambycidae

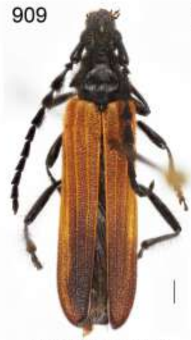

indet sp. Western Australia

Cerambycidae

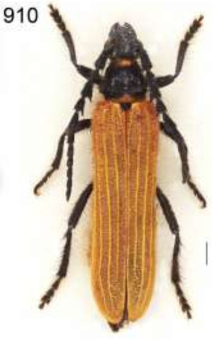

indet sp. Australian Capital Territory

Cerambycidae

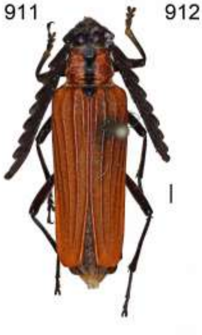

Distichocera maculicollis New South Wales

Belidae

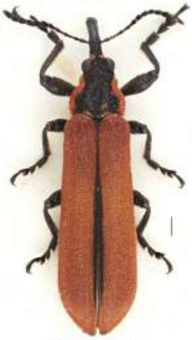

Rhinotia sp. Victoria

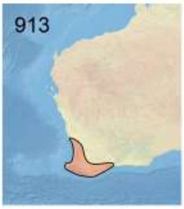

Belidae

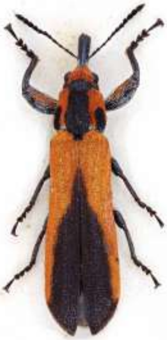

indet sp. Western Australia

All graphics and text produced by the authors as they are listed under the title of this article (CC-BY open access license). Long horn beetle photographs taken by L. Dembicky.

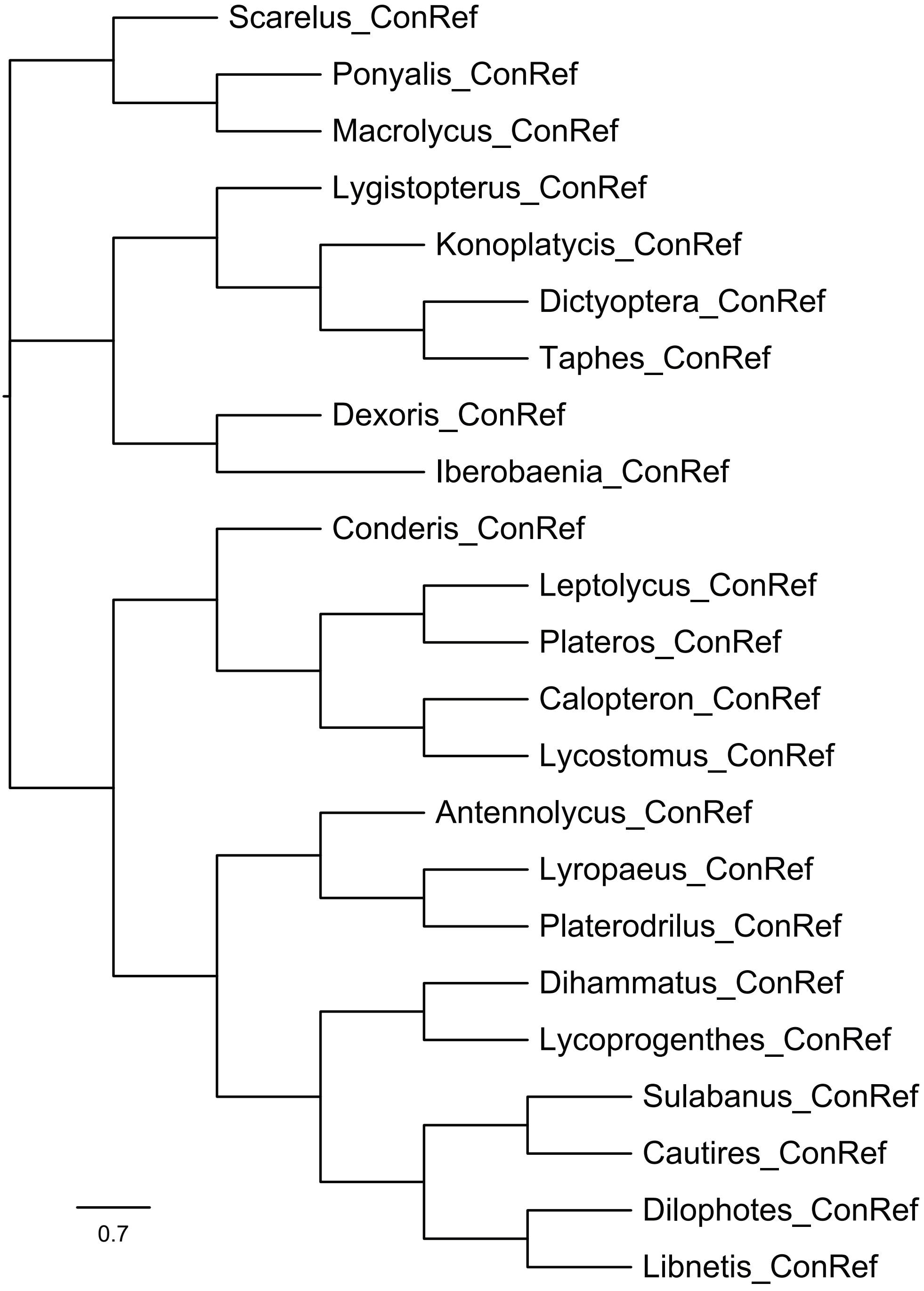

Figure S915. The guiding phylogenomic topology for the maximum likelihood estimation.

All graphics and text produced by the authors as they are listed under the title of this article (CC-BY open acces license). Long horn beetle photographs taken by L. Dembicky.

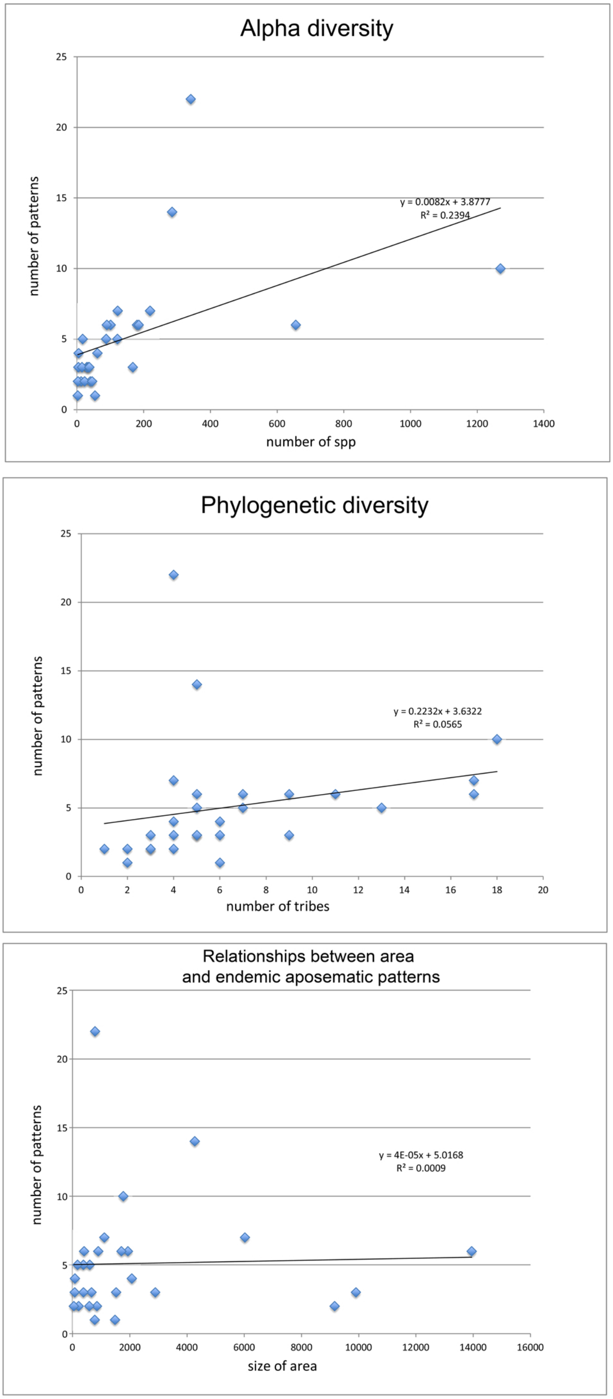

Figure S916. Alpha diversity, phylogenetic diversity, area size and the numbers of endemic aposematic patterns.

All graphics and text produced by the authors as they are listed under the title of this article (CC-BY open acces license). Long horn beetle photographs taken by L. Dembicky.

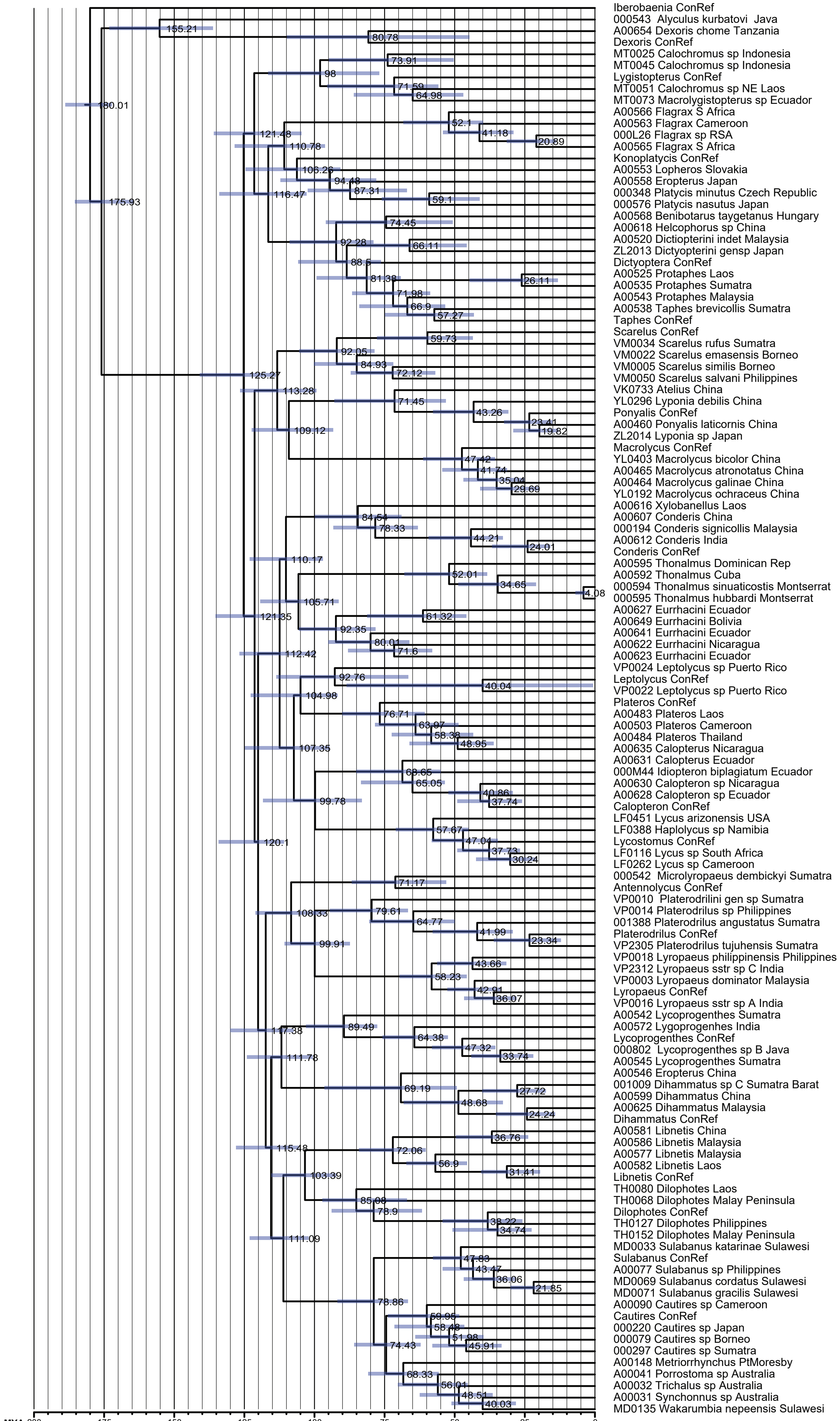

Figure S917. Time-calibrated, maximum clade credibility tree computed using BEAST.

All graphics and text produced by the authors as they are listed under the title of this article (CC-BY open acces license). Long horn beetle photographs taken by L. Dembicky.

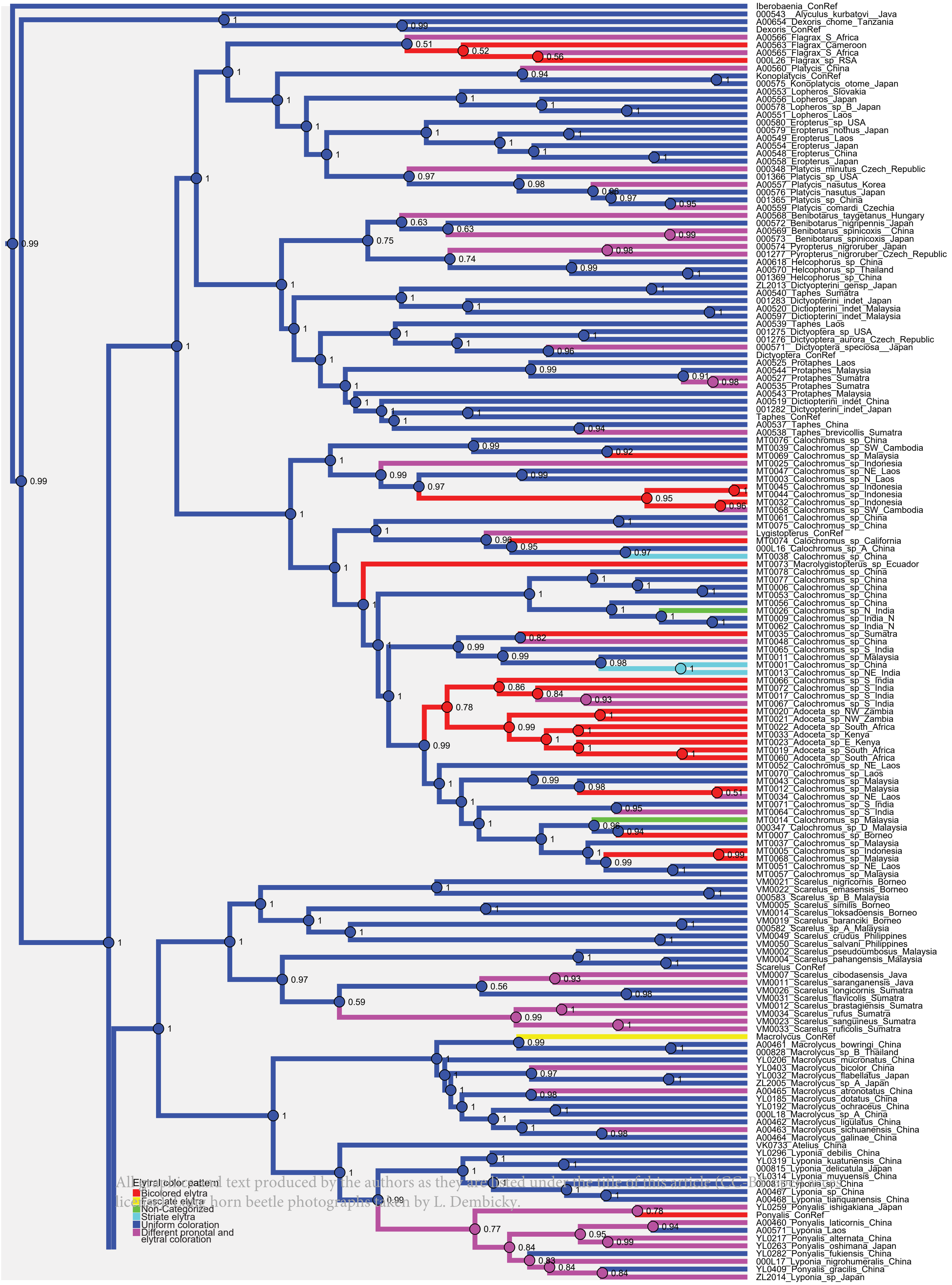

All graphics and text produced by the authors as they are listed under the title of this article (CC-BY licence) [illegible] horn beetle photographs taken by L. Dembicky.

Figure S918. The reconstruction of the ancestral pattern coloration using BEAST and constrained topology from IQ-Tree analysis. The figure continues on the next page.

All graphics and text produced by the authors as they are listed under the title of this article (CC-BY open acces
license). Long horn beetle photographs taken by L. Dembicky.

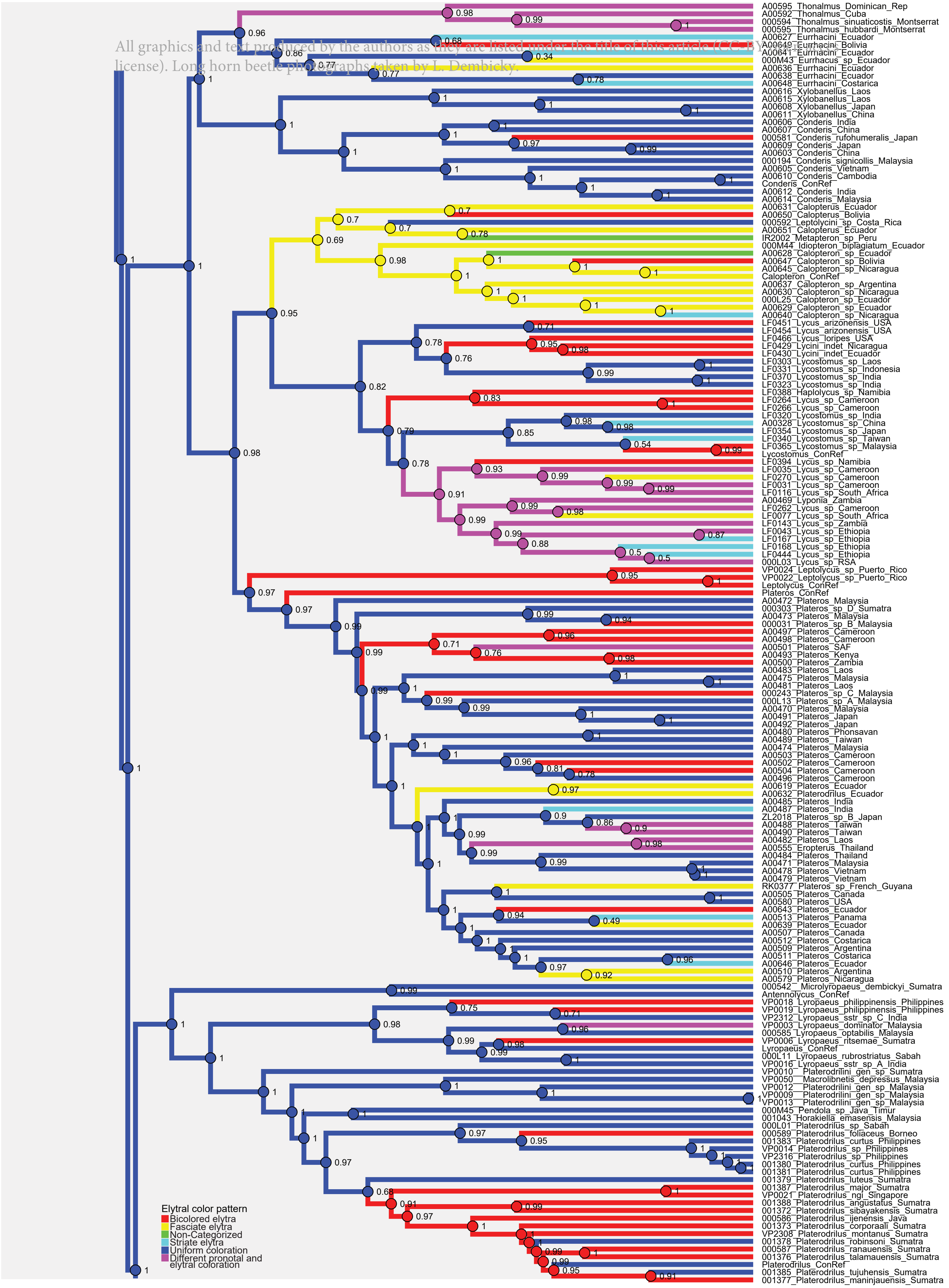

Figure S918. The reconstruction of the ancestral pattern coloration using BEAST and constrained topology from IQ-Tree analysis. The figure continues on the next page.

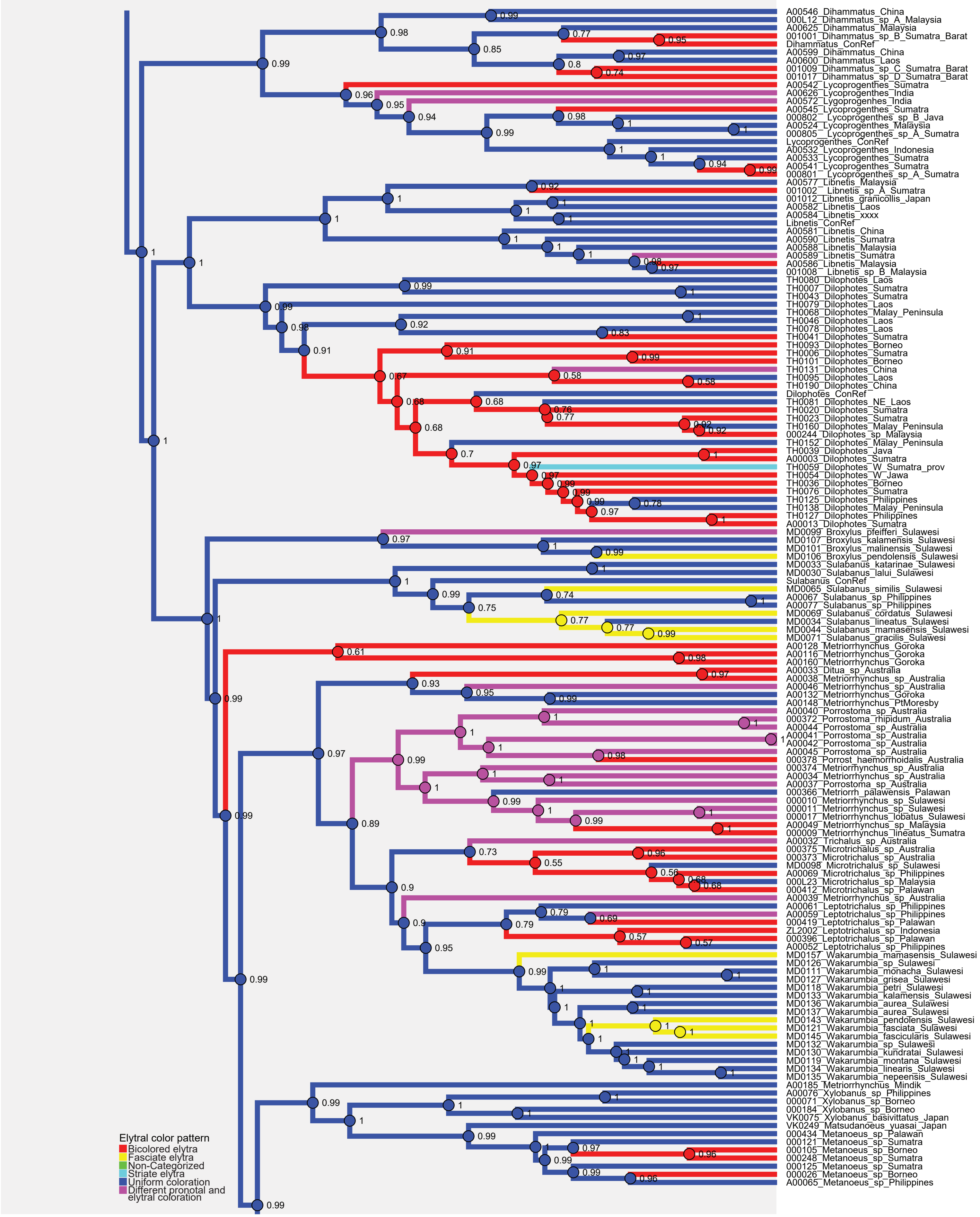

All graphics and text produced by the authors as they are listed under the title of this article (CC-BY open acces license). Long horn beetle photographs taken by L. Dembicky.

Figure S918. The reconstruction of the ancestral pattern coloration using BEAST and constrained topology from IQ-Tree analysis. The figure continues on the next page.

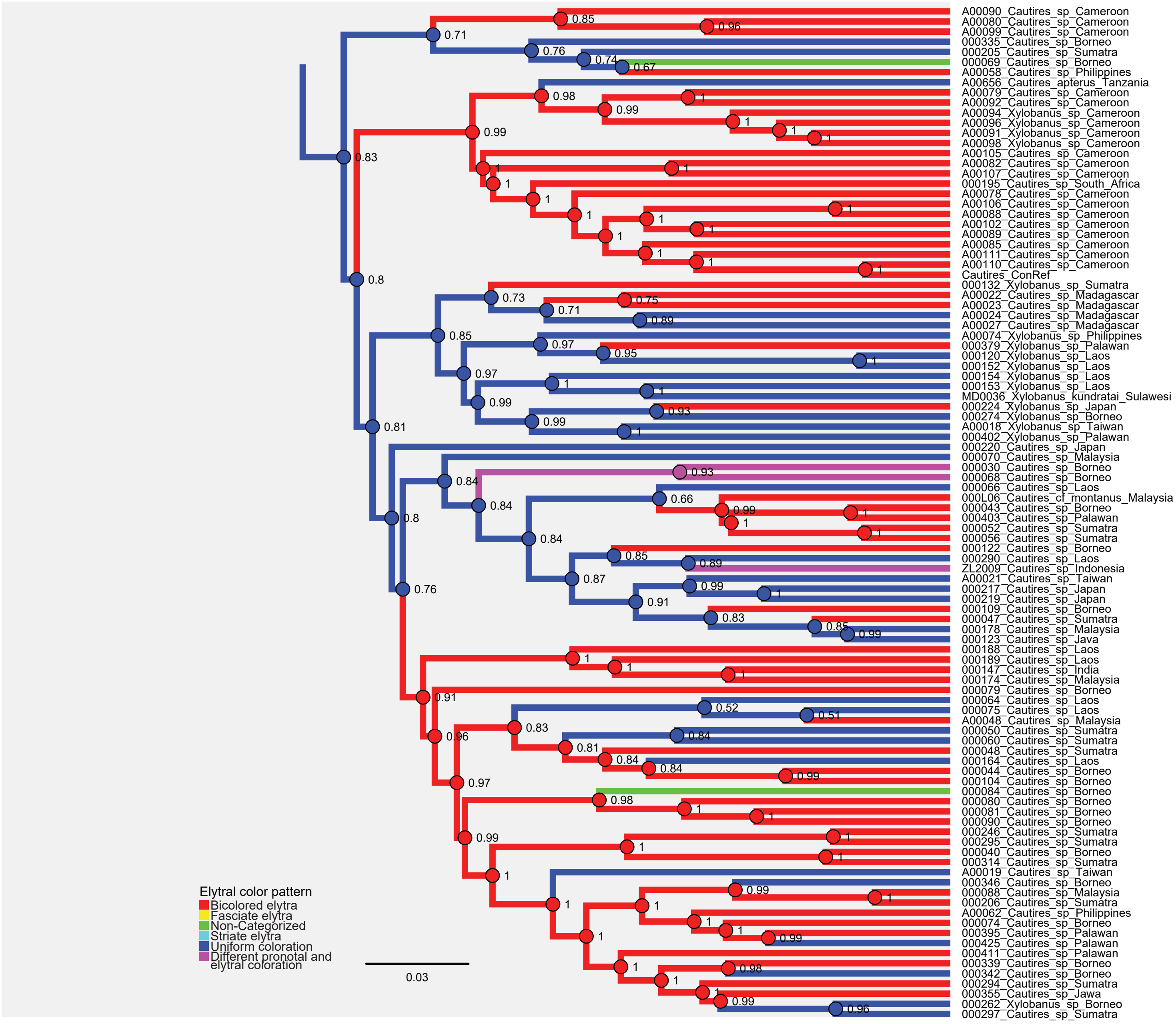

Figure S918. The reconstruction of the ancestral pattern coloration using BEAST and constrained topology from IQ-Tree analysis. The figure continues on the next page.

All graphics and text produced by the authors as they are listed under the title of this article (CC-BY open acces license). Long horn beetle photographs taken by L. Dembicky.

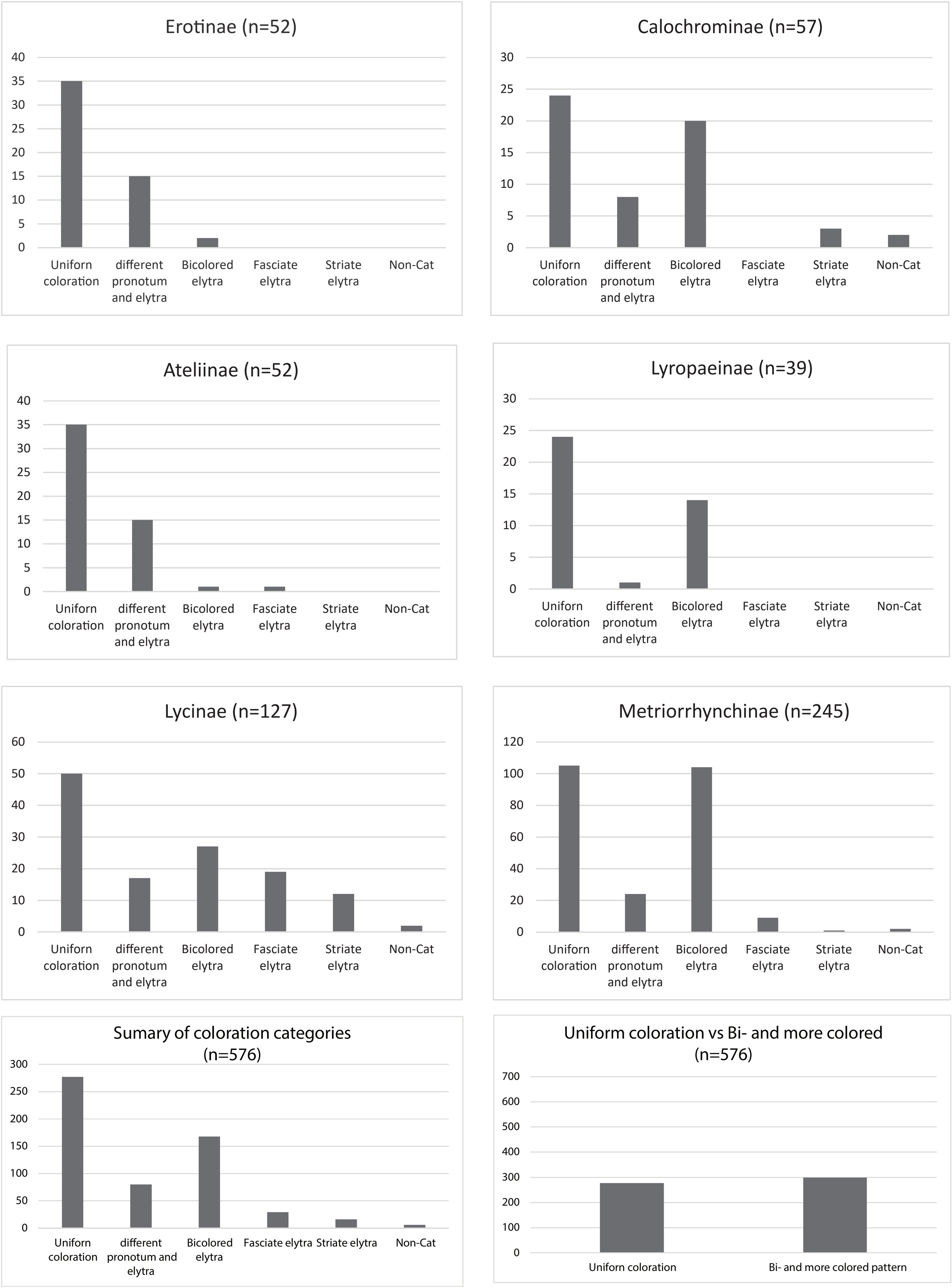

Figure S919. Colour patterns recorded in lycid subfamilies.

All graphics and text produced by the authors as they are listed under the title of this article (CC-BY open acces license). Long horn beetle photographs taken by L. Dembicky.

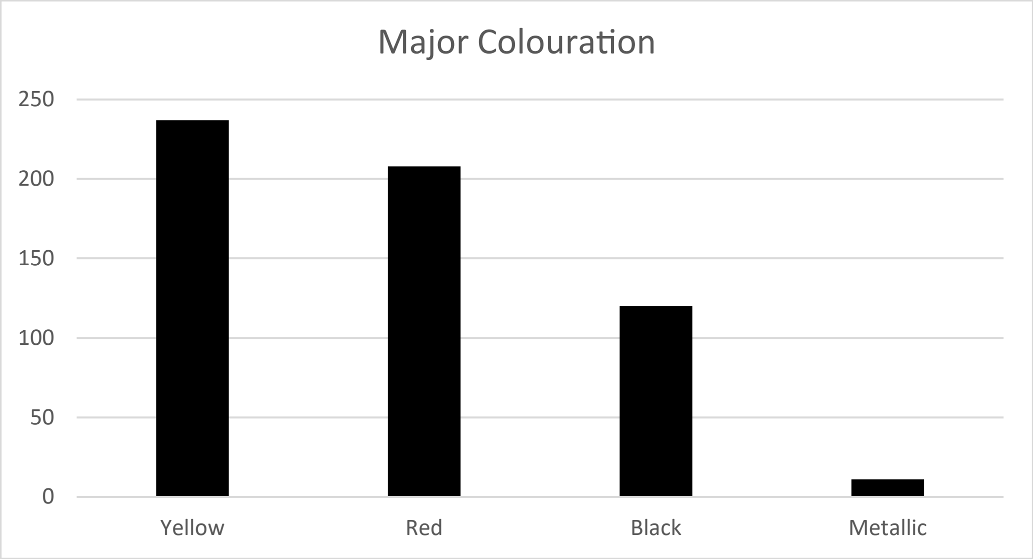

Figure S920. The dominant dorsal colouration in net-winged beetles.

All graphics and text produced by the authors as they are listed under the title of this article (CC-BY open acces license). Long horn beetle photographs taken by L. Dembicky.

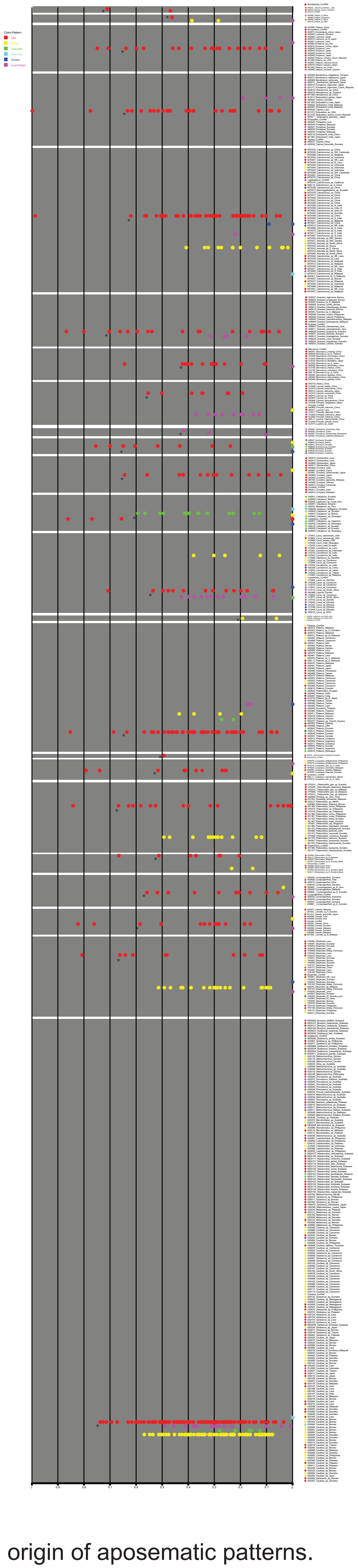

Figure S921. The dating of the origin of aposematic patterns.

All graphics and text produced by the authors as they are listed under the title of this article (CC-BY open acces license). Long horn beetle photographs taken by L. Dembicky.
